# FAF1 and FAF2 enhance unfolding by p97-UFD1-NPL4 complex enabling rational design of p97 activators

**DOI:** 10.1038/s44318-026-00894-x

**Published:** 2026-08-15

**Authors:** Pritha Dasgupta, Ian R Kelsall, Gaurav Anand, Anna Pérez-Ràfols, Thomas A Jowitt, Axel Knebel, Robert Gourlay, Glenn R Masson, Yogesh Kulathu

**Affiliations:** 1 MRC Protein Phosphorylation and Ubiquitylation Unit, Faculty of Life Sciences, University of Dundee, Dundee, DD1 5EH UK; 2 Wellcome Trust Centre for Extracellular Matrix Research, Faculty of Biology Medicine and Health, University of Manchester, Manchester, UK; 3 Division of Cancer Research, School of Medicine, University of Dundee, Dundee, DD1 9SY UK

**Keywords:** Post-translational Modifications & Proteolysis

## Abstract

VCP/p97 is an AAA+ ATPase that, together with its cofactors UFD1-NPL4 (p97-UN), unfolds ubiquitylated substrates to maintain cellular homeostasis. The human p97-UN complex associates with additional cofactors, but how these cofactors modulate p97-UN activity is not fully understood. Here, we screen cofactors and identify FAF2 to potently enhance substrate unfolding by p97-UN. Using biochemical and structural approaches, we show how FAF2 engages p97-UN and polyubiquitin to promote unfolding. We define a conserved activation motif in FAF2 that contacts both UFD1 and the ubiquitin proximal to the initiator, thereby stabilizing and supporting the unfolding of the initiator ubiquitin in a UFD1-dependent manner. We leverage the features of the FAF2 activation motif to engineer de novo proteins that potently enhance unfolding, providing a rational strategy to boost p97 activity. Our findings reveal how cofactors can provide additional adaptive control, fine-tuning human p97 activity to unfold challenging substrates and those modified with short ubiquitin chains.

## Synopsis

Efficient unfolding by the ATPase p97/VCP is essential for protein quality control, yet human p97 is far less efficient than its yeast counterpart for reasons that have remained unclear. This work investigates the cofactor and the mechanism enabling efficient unfolding by the human enzyme.

- A cofactor screen identifies FAF2 as the strongest activator of substrate unfolding by the human p97-UFD1-NPL4 complex.
- FAF2 binds the p97-UFD1-NPL4 complex in a UFD1-dependent manner through its conserved activation motif.
- The FAF2 activation motif simultaneously engages the UFD1 UT3 domain, the substrate-proximal and initiator ubiquitin, thereby accelerating the rate-limiting initiator unfolding step.
- These mechanistic insights allow computational *de-novo* design of mini-proteins that potently activate human p97.

## Abstract (web summary)

A cofactor screen identifies FAF2 as the strongest activator of substrate unfolding by the human p97-UFD1-NPL4 complex.

## Graphical abstract

**Figure:**
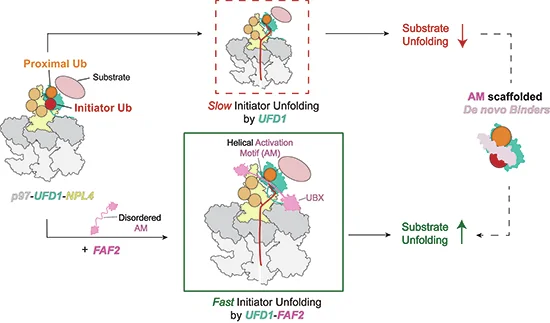

## Introduction

Efficient proteasomal degradation often requires well-folded proteins to be unfolded and extracted from their native environment. This process is carried out by VCP/p97, an evolutionary conserved AAA+ ATPase, which functions upstream of the proteasome as a segregase and unfoldase (Beskow et al, 2009; Olszewski et al, 2019). By extracting proteins from membranes, chromatin, and multiprotein complexes, p97 facilitates their subsequent delivery to the proteasome for degradation (Ye et al, 2017; van den Boom and Meyer, 2018). Accordingly, p97-dependent unfolding is essential in several quality control pathways, including endoplasmic reticulum-associated degradation (ERAD), ribosome-associated quality control, and several nuclear processes (Maric et al, 2014; Brandman et al, 2012; Stein et al, 2014; Ahlstedt et al, 2022). The importance of p97 function is underscored by autosomal dominant mutations that disrupt protein homoeostasis and cause multiple neurodegenerative disorders, including multisystem proteinopathy (MSP), amyotrophic lateral sclerosis (ALS), and vacuolar tauopathy (Darwich et al, 2020; Watts et al, 2004; Johnson et al, 2010; Saracino et al, 2018).

p97 functions as a homo-hexamer, with each protomer comprising an N-terminal domain (N) and two tandem ATPase domains (D1 and D2) arranged in stacked rings. The D1 and D2 rings form a central pore through which substrate polypeptides are threaded and mechanically unfolded by ATP hydrolysis-driven conformational changes (Twomey et al, 2019; Cooney et al, 2024). Substrate recognition is a key regulatory step and is commonly mediated by K48-linked polyubiquitin chains (Richly et al, 2005; Blythe et al, 2017). This ubiquitin-dependent selectivity is achieved through several adaptor complexes that bind to p97 and recruit specific substrates in different subcellular compartments, thereby functionalizing p97 for multiple cellular processes. Of these, UFD1-NPL4 (UN) is a core adaptor complex that contains multiple ubiquitin binding modules, preferentially binding K48-linked chains and recruiting ubiquitylated substrates to p97 (Meyer et al, 2000). Both UFD1 and NPL4 bind to the N-terminal domain of p97 via SHP motifs (SHP1 and SHP2) and a ubiquitin regulatory X-like (UBX-L) domain, respectively, with NPL4 arranged in a characteristic tower-like structure above the D1 ATPase ring (Twomey et al, 2019; Pan et al, 2021a, 2021b). The NPL4 C-terminal domain (CTD) and Mpr1/Pad1 N-terminal (MPN) tower engage distal ubiquitins of substrates modified with K48-linked chains, while the UFD1 UT3 domain binds to the proximal ubiquitin located closer to the substrate (Park et al, 2005; Sato et al, 2019; Twomey et al, 2019; Williams et al, 2023). This multi-site cooperative binding destabilises one ubiquitin in the chain, the initiator, which becomes captured in the NPL4 groove and unfolds in an ATP-independent manner, positioning its N-terminus for ATP-dependent translocation through the central pore (Bodnar & Rapoport, 2017; Twomey et al, 2019; Pan et al, 2021a; Ji et al, 2022). This substrate engagement mechanism is fundamentally distinct from the proteasome, which initiates unfolding through recognition of unstructured regions in the substrate protein itself (Bard et al, 2019).

Beyond the canonical adaptors UN, p97 associates with a diverse array of adaptors which modulate its function and direct it to specific cellular pathways (Buchberger et al, 2015; Hänzelmann and Schindelin, 2017). Many of these cofactors are shown to function in concert with UN (Hänzelmann et al, 2011; Fujisawa et al, 2022; Kochenova et al, 2022). While the UN is essential for substrate recruitment and unfolding, the contribution of additional cofactors to p97 function remains incompletely understood. Strikingly, this regulatory cofactor network is far more expanded in humans. While yeast Cdc48 functions with roughly 15 cofactors, human p97 interacts with a much broader set of more than 30 cofactors, highlighting increased functional diversification and context-specific regulation. Consistently, comparative studies between yeast Cdc48-Ufd1-Npl4 (Cdc48-UN) and mammalian p97-UFD1-NPL4 (p97-UN) revealed intriguing functional differences. In contrast to yeast Cdc48, mammalian p97-UN requires substantially longer or more complex heterotypic ubiquitin chains to achieve efficient substrate unfolding in reconstituted systems (Blythe et al, 2017; Fujisawa et al, 2022). Even atypical homotypic chains other than K48-linked and branched chains have been reported to target substrates for p97-mediated unfolding (Castañeda et al, 2016; Heidelberger et al, 2018; Lange et al, 2024; Suryo Rahmanto et al, 2023). Studies on canonical K48-linked chains revealed that both yeast and human complexes share highly conserved architecture and ubiquitin-binding mechanisms (Twomey et al, 2019; Bodnar and Rapoport, 2017; Pan et al, 2021a), suggesting that any differences in function are unlikely to arise from the core machinery alone. This raises the possibility that mammalian p97 relies on additional regulatory factors, although the identity and mechanisms of such factors remain underdefined.

To address how cofactors impact human p97 unfolding activity, we employ in vitro reconstitution and a focused biochemical screen to identify human p97 cofactors that enhance unfoldase activity of human p97 in the presence of UN. Of the adaptors tested, Fas-associated factor 2 (FAF2/UBXD8), a UBX domain adaptor protein, shows the strongest effect on unfolding by p97-UN. Through biochemical and structural analyses, we define the molecular features that underpin FAF2-mediated stimulation. Based on these insights, we employ computational protein design to engineer de novo mini-proteins that mimic FAF2 function within novel protein scaffolds to potently activate p97. Our work reveals how the unfoldase activity of p97 can be enhanced by additional cofactors, an adaptive feature we believe is important to unfold misfolded proteins and extract proteins from membranes and tightly bound multiprotein complexes.

## Results

### Human p97-UFD1-NPL4 exhibits reduced unfoldase activity

**Figure EV1:**
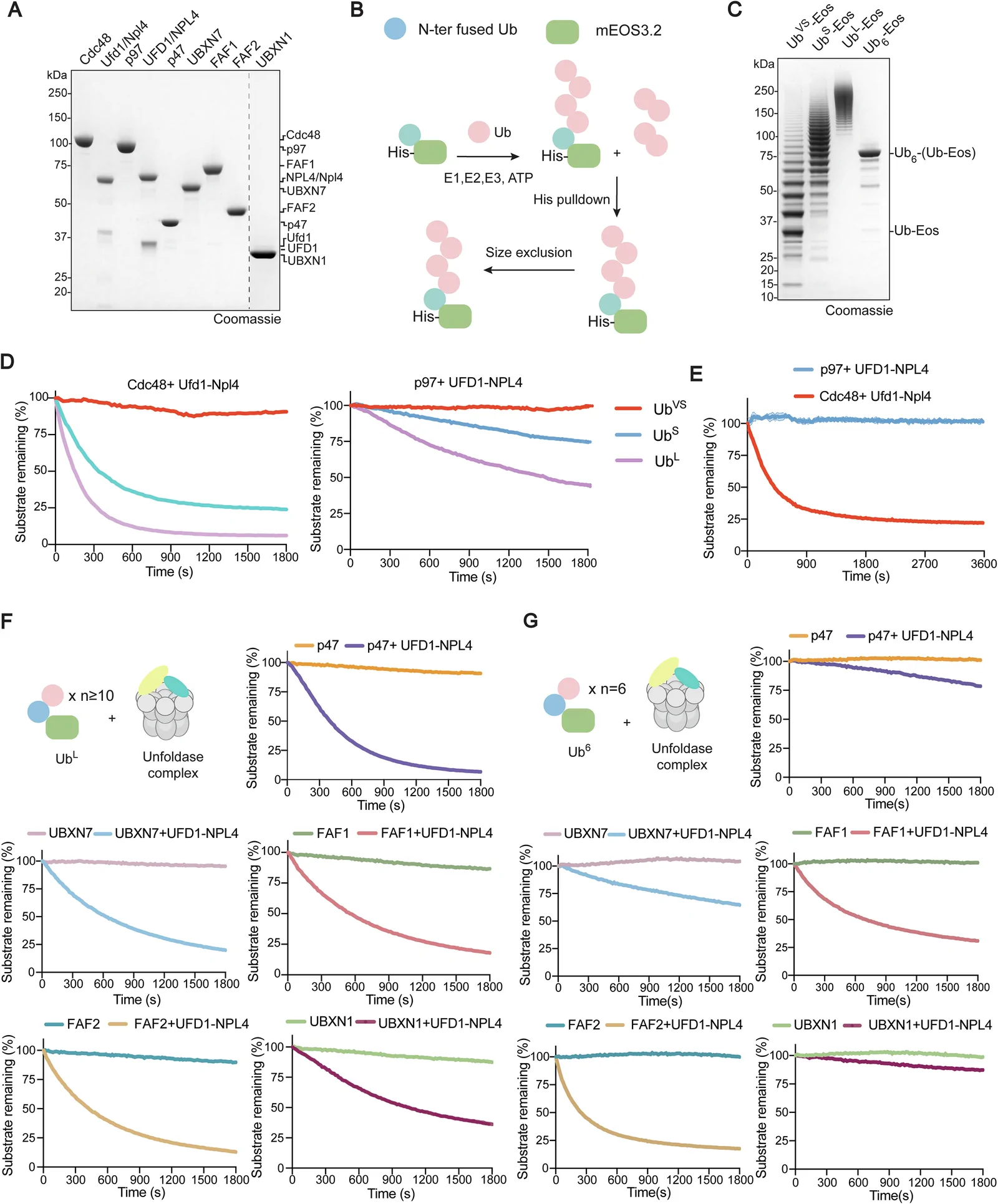
Differential modulation of human p97 activity by UBA-UBX cofactors requires UFD1-NPL4. (**A**) Coomassie-stained SDS-PAGE showing purified human p97/yeast Cdc48, yeast Ufd1-Npl4 complex, human UFD1-NPL4 complex and UBA-UBX cofactor proteins used in this study. (**B**) Schematic representing the overview of the method employed to prepare ubiquitylated reporter Eos substrates. (**C**) Coomassie-stained SDS-PAGE of K48-linked ubiquitylated Eos substrates of various lengths used in the unfolding assays. (**D**) Unfolding of Ub^VS^, Ub^S^, and Ub^L^ substrates by yeast Cdc48-Ufd1-Npl4 (left) and human p97-UFD1-NPL4 (right). (**E**) Comparison of unfolding of Ub_6_-Eos by human p97-UFD1-NPL4 and yeast Cdc48-Ufd1-Npl4 unfoldase complex. (**F**) Ub^L^ substrate unfolding by human p97 with UBA-UBX cofactors (p47, UBXN7, FAF1, FAF2 or UBXN1), alone or combined with UFD1-NPL4, showing cofactor-specific modulation of activity. (**G**) Substrate unfolding kinetics of Ub_6_-Eos substrate by human p97 in the presence of UBA-UBX cofactors, either alone or in combination with UFD1-NPL4, demonstrating cofactor-specific effects on unfolding depend on ubiquitin chain length on the client. For panels (**D**–**G**), fluorescence intensity values were normalised to values at time 0 and substrate-only controls. Data shown as mean ± SD (*n* ≥ 3 technical replicates for (**D**, **E**), *n* = 3 independent experiments, each with three technical replicates for (**F**) and *n* = 2 independent experiments, each with three technical replicates for (**G**). Source data are available online for this figure.

**Figure 1:**
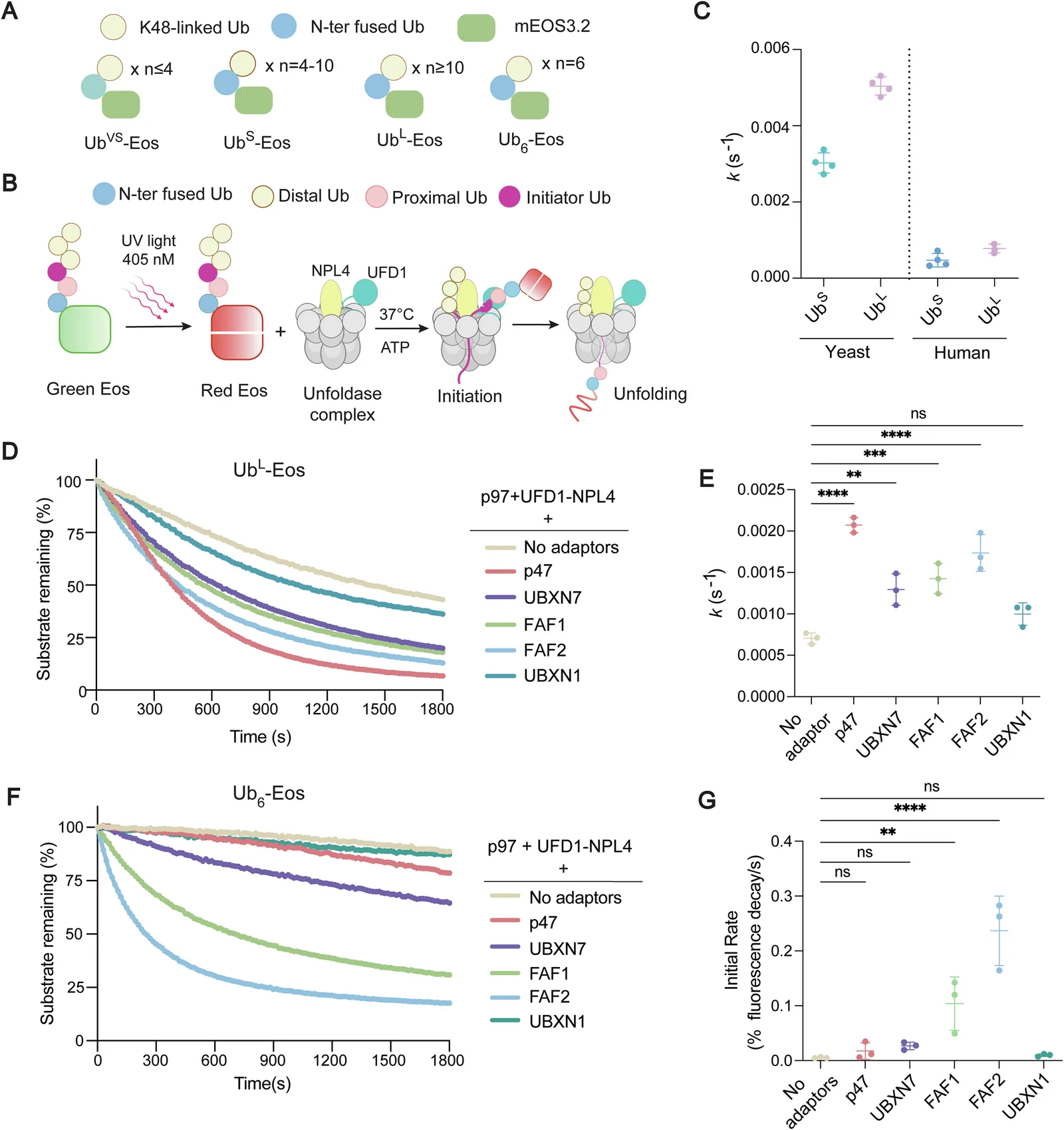
UBA-UBX cofactors enhance unfolding of ubiquitylated substrates by the human p97-UFD1-NPL4 complex. (**A**) Schematic describing the architecture of the ubiquitylated substrate used for the in vitro unfolding assay. Substrate modified with K48-linked ubiquitin chains of ≤4 ubiquitin (Ub^VS^), 4–10 ubiquitin (Ub^S^), and very long chains of ≥10 ubiquitin (Ub^L^). Ub_6_-Eos denotes Ub-Eos substrate modified with a hexameric Ub chain. (**B**) Schematic representing the principles of substrate unfolding assay with reconstituted p97-cofactor assemblies. (**C**) Comparison of the rate of unfolding (*k*) of Ub^S^ and Ub^L^ substrate by yeast Cdc48-Ufd1-Npl4 (left) and human p97-UFD1-NPL4 (right). Data represent the mean ± SD for *n* ≥ 3 technical replicates. (**D**) Single-turnover unfolding assay comparing unfolding of Ub^L^-Eos by p97-UN complex alone or upon addition of indicated UBA-UBX adaptors. Data representative of n = 3 independent experiments, each performed with three technical replicates. (**E**) Comparison of the rate of unfolding *k (*sec^−1^) for various p97-cofactor assemblies towards Ub^L^ substrate. Data represent the mean ± SD for *n* = 3 independent experiments, each with three technical replicates. Statistical significance was calculated by one-way ANOVA followed by multiple-comparison testing (no adaptor vs. p47, *p* < 0.0001; no adaptor vs. UBXN7, *p* = 0.0028; no adaptor vs. FAF1, *p* = 0.0005; no adaptor vs. FAF2, *p* < 0.0001; no adaptor vs. UBXN1, *p* = 0.1475). (**F**) Screening of the same UBA-UBX cofactors towards Ub_6_-Eos substrate. The unfolding traces are representative of *n* = 2 independent experiments, each with three technical replicates. (**G**) Comparison of the initial rate (first 2 min) of unfolding for various p97-cofactor assemblies towards Ub_6_-Eos substrate. Data represent the mean ± SD for *n* = 3 independent experiments, each with three technical replicates. Statistical significance was calculated by one-way ANOVA followed by multiple-comparison testing (no adaptor vs. p47, *p* = 0.9975; no adaptor vs. UBXN7, *p* = 0.9123; no adaptor vs. FAF1, *p* = 0.0011; no adaptor vs. FAF2, *p* < 0.0001; no adaptor vs. UBXN1, *p* > 0.9999). The fluorescence decay is represented by normalising the fluorescence intensity of each time point to time 0 and to the substrate-only controls. In panels (**C**, **E**), the unfolding rate *k* (s^−1^) was determined by fitting the unfolding trace to a single exponential decay. In panel (**G**), the initial rate of unfolding was calculated from the linear fitting of the first 2 min of the unfolding reaction because of poor fitting of a few conditions into an exponential decay function. Statistical significance is indicated as ns not significant (*p* > 0.05), *p ≤ 0.05, **p ≤ 0.01, ***p ≤  0.001, ****p ≤ 0.0001. Source data are available online for this figure.

Yeast p97 (Cdc48) has been well characterised and widely used for biochemical and structural studies (Twomey et al, 2019; Bodnar & Rapoport, 2017). In contrast, the regulation and activity of human p97 are not fully understood. To directly compare yeast and human systems, we performed in vitro unfolding assays with the yeast and human unfoldase complex (Fig. EV1A), using the model substrate monomeric Eos fluorescent protein version 3.2 with N-ter fused ubiquitin (Ub-mEos3.2), hereafter referred to as Ub-Eos (Blythe et al, 2017). This substrate mimics ubiquitin fusion degradation (UFD) pathway substrates such as Ub(G76V)-GFP, which has been established as a bona fide p97 substrate in cells (Beskow et al, 2009; Johnson et al, 1995; Wójcik et al, 2006; Dantuma et al, 2000; Chou et al, 2011). Moreover, the Eos undergoes backbone cleavage upon UV irradiation, with a consequent shift from green to red fluorescence. Following unfolding, the two Eos fragments cannot refold, leading to an irreversible loss in fluorescent signal. We modified the Ub-Eos with single K48-linked polyubiquitin chains of various lengths: long (Ub^L^, ≥10 ubiquitin), short (Ub^S^, 4–10 ubiquitin), and very short (Ub^VS^, ≤4 ubiquitin) (Figs. 1A and EV1B,C). Following photoconversion, the ubiquitylated Ub-Eos substrates were incubated with reconstituted yeast Cdc48-UN complexes in the presence of ATP, resulting in a steady-state decrease in fluorescence that directly reports on substrate unfolding kinetics under single-turnover conditions (Fig. 1B). Human p97 is known to contain multiple post-translational modification sites that are known to influence its adaptor interactions, localisation, and activity (Zhao et al, 2007; Hänzelmann and Schindelin, 2017). To retain these modifications, the human complex was reconstituted with p97 hexamers purified using a baculovirus expression system (Fig. EV1A). In comparison to the yeast Cdc48 complex, we observed a pronounced inefficiency in unfolding by human p97 purified from insect cells. Ub^VS^, mEOS substrate modified with fewer than 4 Ub molecules, was not unfolded by either yeast or human p97, implying that this specific chain length is below the functional threshold. While the yeast complex efficiently unfolded both short- and long-chain substrates (Ub^S^ and Ub^L^), with unfolding rates of *k* = 0.003 s⁻¹ and *k* = 0.005 s⁻¹, respectively, human p97 displayed markedly reduced activity. Unfolding of Ub^L^ by human p97 (*k* = 0.0008 s⁻¹) was ~6-fold slower than that of the yeast complex, whereas Ub^S^ was largely resistant to unfolding (Figs. 1C and EV1D). A similar kinetic difference was observed using Ub_6_-Eos, a substrate bearing a defined K48-linked hexamer, further highlighting the slower activity of human p97 (Fig. EV1E). Importantly, this unfolding deficit was observed across all ubiquitin chain lengths tested, indicating that the reduced activity of human p97-UN is a general feature of the complex rather than a consequence of insufficient substrate ubiquitylation, as previously suggested (Fujisawa et al, 2022) and raises the possibility that human p97 relies on additional factors beyond UN to achieve efficient substrate unfolding.

### Additional cofactors differentially modulate the unfolding activity of human p97

Proteomics analyses and in vitro studies revealed multiple UBX domain-containing p97 adaptors that interact with the UN complex (Raman et al, 2015; Hänzelmann et al, 2011). Notably, a subset of these adaptors has been shown to lower the ubiquitin chain length threshold required for human p97-dependent substrate processing (Fujisawa et al, 2022). Based on these observations, we tested whether UBX proteins that can simultaneously engage ubiquitylated clients influence substrate unfolding by the p97-UN complex. We focused on five p97 adaptors—p47/NSFL1C, UBXN7/UBXD7, FAF1/UBXD12, FAF2/UBXD8 and UBXN1/SAKS1, which all contain a UBX domain and ubiquitin-associated (UBA) domain for binding p97 and ubiquitin, respectively. We assessed the ability of these adaptors to stimulate Ub^L^ substrate unfolding in vitro. All adaptors were expressed as full-length proteins, except FAF2, where we deleted the membrane-tethering hairpin (HP) motif to facilitate soluble expression in *E. coli*. We observed a significant enhancement of unfolding activity in the presence of p47, UBXN7, FAF1 and FAF2, whereas UBXN1 showed no detectable effect (Fig. 1D). Kinetic analysis revealed p47 as the most potent activator among the adaptors tested (Fig. 1E). Importantly, UN was indispensable, as none of these adaptors by themselves can accelerate the unfolding of heavily ubiquitylated substrate independently (Fig. EV1F). These results indicate that p97, together with UN, forms the core unfoldase module, while the additional adaptor proteins function cooperatively to modulate or amplify its activity, thereby regulating substrate unfolding.

Model p97 substrates used for in vitro studies are typically modified with very long ubiquitin chains, and it is unclear whether such long ubiquitin chains are made in cells and how prevalent they are. Quantitative proteomics revealed that the composition of K48-linked polyUb in yeast consists of 2 to 7 ubiquitin moieties per chain at steady state (Tsuchiya et al, 2018). Hence, to mimic the predominant pool of p97 clients available in vivo, we generated Ub-Eos modified with chemically defined K48-linked Ub_6_ (Ub_6_-Eos; Fig. EV1C), to allow us to assess how a physiologically relevant Ub signal is decoded by the unfoldase complex. The canonical UN adaptor was unable to unfold the short-chain modified substrate, in agreement with the higher ubiquitin threshold of the human p97-UN complex (Fujisawa et al, 2022). We next analysed the impact of UBA-UBX adaptors on unfolding of the Ub_6_-Eos substrate under identical conditions. While p47 potentiated unfolding of the Ub^L^ substrate, it failed to support efficient unfolding of Ub_6_-Eos, suggesting that p47-mediated stimulation requires a minimum ubiquitin chain length threshold. Of the adaptors screened, only FAF1 and FAF2 supported efficient unfolding of short-chain substrate (Fig. 1G). In comparison, UBXN7 supported only moderate unfolding (Figs. 1F,G and EV1G). In summary, these results reveal that adaptor-mediated activation of p97 function varies widely among UBA-UBX cofactors and is strongly influenced by the length of the polyUb chain on the client proteins.

### FAF2 enhances unfolding of ubiquitylated substrates

**Figure 2:**
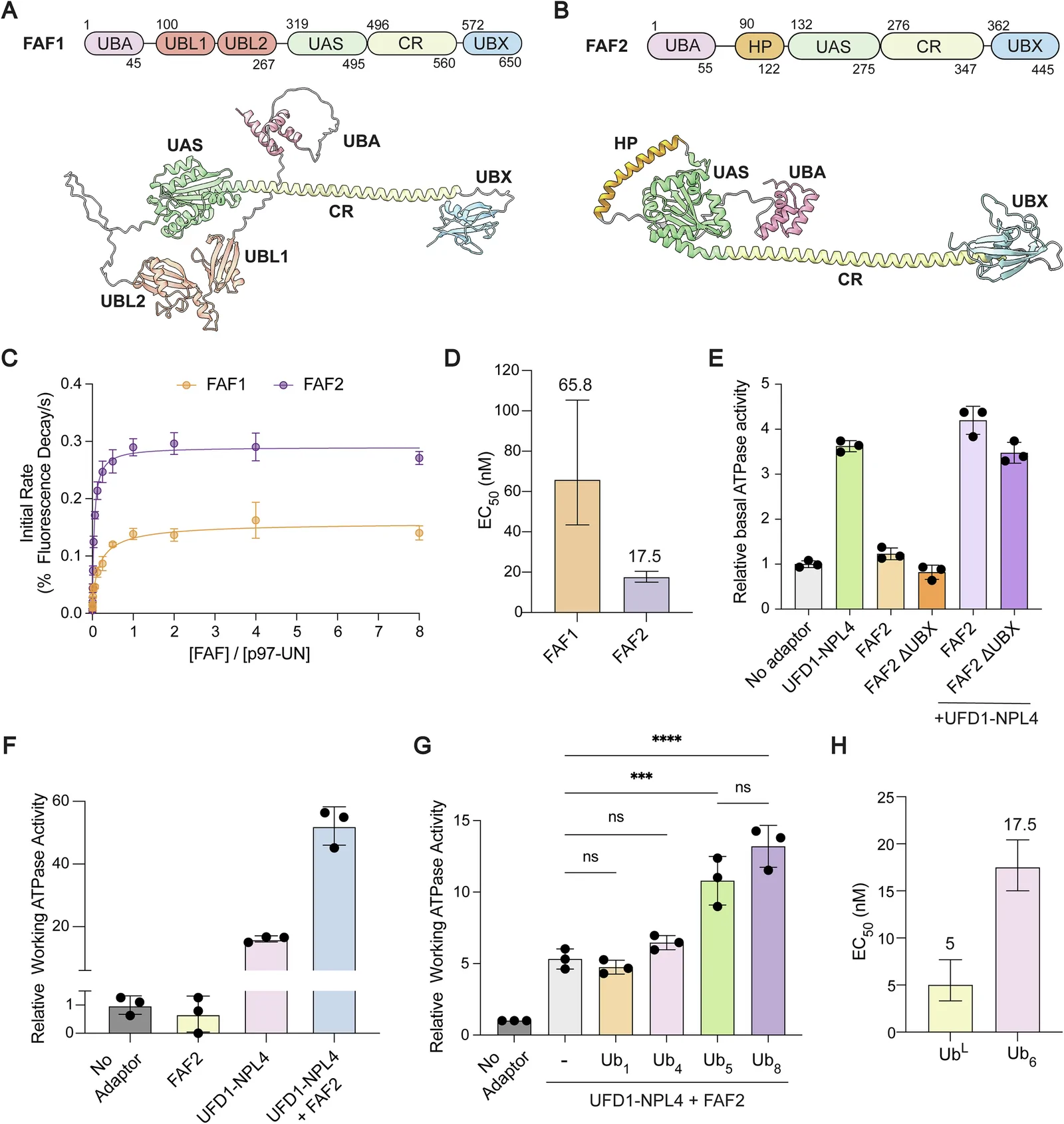
Enhanced unfolding of ubiquitylated substrate by FAF2. (**A**) Domain organisation (top) and AF3 structure (bottom) of full-length FAF1, highlighting the UBA, UBL1, UBL2, UAS, CR and UBX domains. (**B**) Domain organisation (top) and AF3 structure (bottom) of full-length FAF2, highlighting the UBA, Hairpin (HP) motif, UAS, CR and UBX domains. (**C**) Initial unfolding rate (first 2 min) of Ub_6_-Eos unfolding as a function of the molar ratio of FAF1/2 and p97-UN complex. p97 (400 nM) and UN (400 nM) concentrations were kept constant. Data shown as mean ± SD for* n* = 4 technical replicates. (**D**) Comparison of EC_50_ values of FAF1 and FAF2 for enhancing the unfolding rate of Ub_6_-Eos substrate by p97-UFD1-NPL4 complex. EC_50_ values were calculated from data in (**C**). Error bars denote 95% CI. (**E**) Relative ATPase activity of p97 without or with the indicated cofactors. Basal ATP hydrolysis was measured and normalised to p97 alone (no adaptor). Data represent the mean ± SD for *n* = 3 technical replicates. (**F**) Relative ATPase activity of p97 with indicated cofactors in the presence of Ub^L^ substrate. Working ATP hydrolysis was measured and normalised to p97 alone. Data represent the mean ± SD for *n* = 3 technical replicates. (**G**) Relative ATPase activity of p97 with indicated cofactors in the presence of monoubiquitin and free K48-linked polyUb chains of various lengths. Working ATP hydrolysis was measured and normalised to p97 alone. Data represent mean ± SD for *n* = 3 independent experiments, each with 3 technical replicates. Statistical significance was calculated by one-way ANOVA followed by multiple-comparison testing (No Ub vs. Ub, *p* = 0.9866; No Ub vs. Ub_4_, *p* = 0.8022; No Ub vs. Ub_5_, *p* = 0.0007; No Ub vs. Ub_8_, *p* < 0.0001; Ub_5_ vs. Ub_8_, *p* = 0.1615). (**H**) Comparison of EC_50_ values of FAF2 for enhancing the unfolding rate of Ub^L^ and Ub_6_-Eos substrate by the p97-UFD1-NPL4 complex. EC_50_ values were calculated from the data shown in Figs. 2C and EV5C. Error bars denote 95% CI. Statistical significance is indicated as ns not significant (*p* > 0.05), *p ≤ 0.05, **p ≤ 0.01, ***p ≤ 0.001, ****p ≤ 0.0001. Source data are available online for this figure.

Having identified FAF1 and FAF2 as the adaptors that most efficiently potentiate unfolding of substrates modified with both short and long ubiquitin chains, we focused on these two proteins to define the most active p97–adaptor assemblies. FAF2 is a membrane-associated p97 adaptor that plays critical roles in ER-associated degradation, lipid droplet turnover, mitochondrial and peroxisomal quality control pathways (Ganji et al, 2023; Koyano et al, 2024; Olzmann et al, 2013; Zheng et al, 2022). AlphaFold3 (AF3) predictions of FAF2 structure reveal that it contains an N-terminal UBA domain (residues ~1–55), a short hydrophobic hairpin (HP) motif (~90–122) which anchors FAF2 to the membrane, a conserved thioredoxin-like UAS domain (~132–275), a long helical central region (CR) (~275–347), and a C-terminal ubiquitin-regulatory X (UBX) domain (~362–445) (Fig. 2B). DALI searches using either the UAS domain together with the helical region or the helical region alone did not yield any meaningful structural homologues. FAF1 shares a similar overall domain architecture, but instead of a membrane-tethering domain, it harbours two additional ubiquitin-like domains (UBL1 and UBL2) at the corresponding position (Fig. 2A).

Using K48-linked Ub_6_-Eos as a model substrate, we found that stoichiometric amounts of either adaptor (one FAF1 or FAF2 per p97-UN complex) were sufficient to achieve maximal unfolding rates, with no further enhancement at higher FAF1/FAF2: p97-UN ratios (Fig. 2C). Notably, FAF2 showed a substantially higher maximal unfolding rate and displayed a lower EC_50_ (17.5 nM) compared to FAF1 (65.8 nM) (Fig. 2C,D). To understand the determinants underlying this effect, we tested whether FAF2 enhances unfolding by modulating ATP hydrolysis activity of p97. While UN significantly enhanced the basal ATPase activity of p97, as measured by an endpoint phosphate release assay, the addition of FAF2 to p97 by itself or to the p97-UN complex did not further increase ATP hydrolysis (Fig. 2E), indicating that FAF2 does not alter the intrinsic motor capacity of p97. Notably, FAF2 markedly increased ATP hydrolysis by the p97-UN complex in the presence of substrate (Fig. 2F). These observations are in agreement with the kinetic analysis of the ATPase activity reported recently (Huo et al, 2025), further supporting the idea that FAF2 selectively enhances substrate-engaged p97-UN activity.

**Figure EV2:**
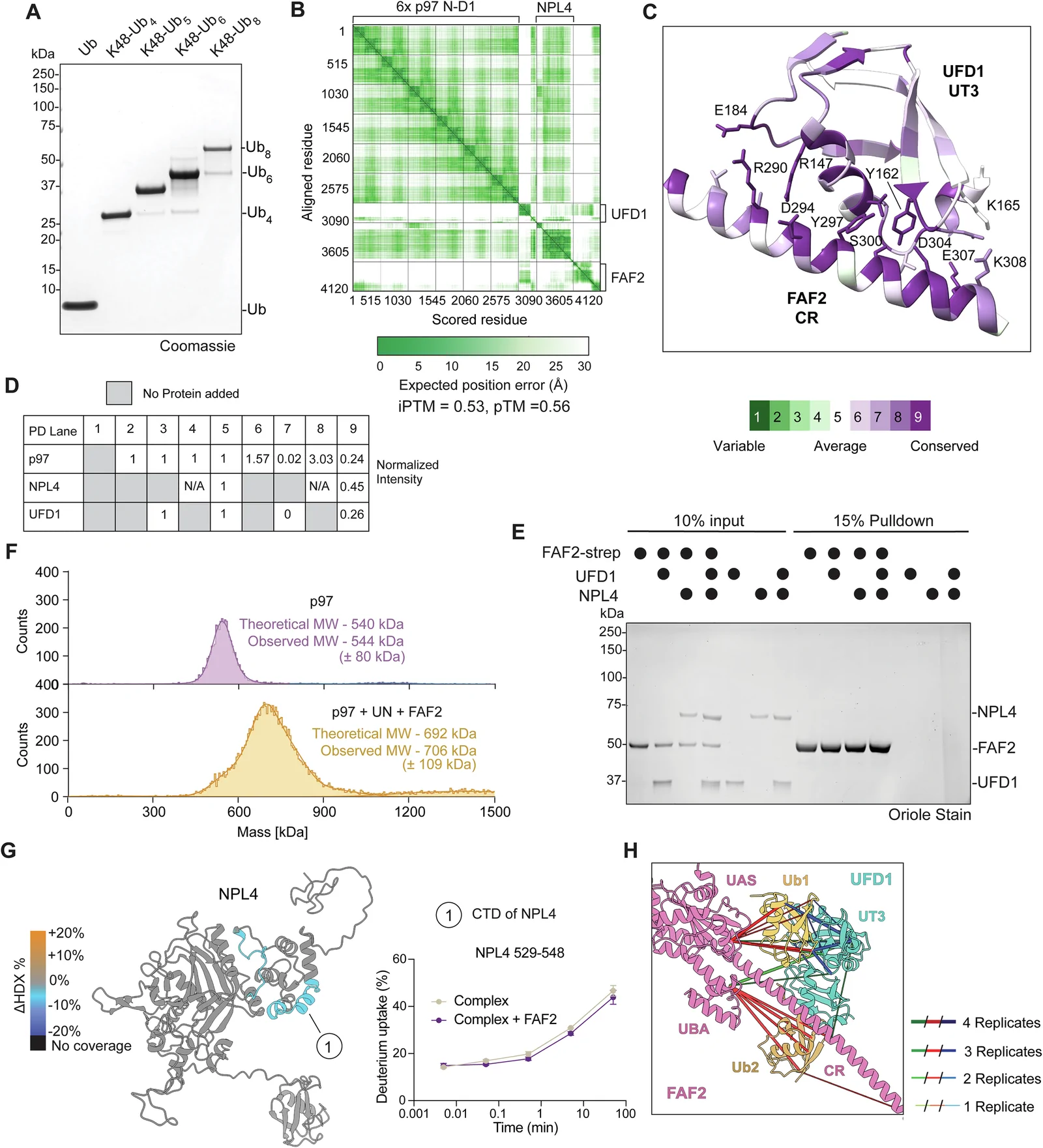
Analysis of p97: UN: FAF2 complex formation. (**A**) Coomassie-stained SDS-PAGE of K48-linked ubiquitin chains used for substrate-stimulated ATPase activity assay. (**B**) PAE plot of the represented AF3 model of p97(ND1)-NPL4-UFD1 complex. (**C**) Conservation analysis of FAF2 and UFD1 generated by ConSurf from the sequences of 150 eukaryotic species. Conservation of each residue is represented by a score between 1 and 9, with 1 being the least conserved and 9 being the most conserved. ConSurf analysis is then mapped onto the AF3 prediction of the p97-UFD1-FAF2 complex, and the UFD1-FAF2 interface is highlighted. (**D**) Quantification of pulldown samples in Fig. 3C. Values are represented by normalising the intensity of each protein under a specific condition to that in the condition where FAF2 was present. (**E**) Strep pull-down of Twin Strep-tagged FAF2 was performed with UFD1, NPL4, or the UFD1-NPL4 complex, followed by Oriole staining. (**F**) Mass photometry analysis of cross-linked p97-UFD1-NPL4-FAF2 complex. Conditions include 5 μM p97 hexamer alone (purple), 5 μM p97 hexamer + 10 μM UFD1-NPL4 + 10 μM FAF2 (orange), in the presence of 600 μM DSSO crosslinker. Histograms are representative of *n* = 3 independent experiments. MW, molecular weight. (**G**) ΔHDX mapping of NPL4 bound to p97 and UFD1 upon FAF2 binding (left). Regions with reduced solvent exchange are shown in blue, increased exchange in orange, no coverage in black, and no significant change (−5% to +5%) in grey. Deuterium uptake kinetics of the peptide in the NPL4 CTD domain showing FAF2-dependent protection (right). Data represent mean ± SD (*n* = 3 independent replicates). (**H**) Experimental crosslinks mapped onto the AlphaFold3 model of the p97-UFD1-NPL4-FAF2-polyUb complex. Cross-linked residue pairs are shown as connecting lines (colouring is the same as Fig. 3F), supporting the predicted complex architecture. Source data are available online for this figure.

We next asked how ubiquitin chain length on the substrate influences FAF2-mediated activation of the p97-UN complex and whether a chain length threshold exists. We therefore measured phosphate release upon addition of free K48-linked Ub chains of varying lengths to the p97-UN-FAF2 complex (Fig. EV2A). Mono-Ub and tetra-Ub (Ub_4_) substrates did not increase ATP hydrolysis, whereas a penta-Ub (Ub_5_) chain significantly enhanced ATP hydrolysis. Extending the chain to octa-Ub (Ub_8_) only caused a minor increase in phosphate release over what was observed for Ub_5_, indicating that a penta-Ub chain is sufficient for stimulation of ATPase activity (Fig. 2G). In line with this observation, FAF2 promoted unfolding of both short (Ub_6_-Eos) and long (Ub^L^-Eos) chain modified substrates, albeit with a modest difference in potency, displaying a lower EC_50_ for the longer chain (5 nM) compared to the shorter chain (17.5 nM) (Fig. 2H). Collectively, these data show that FAF2-dependent activation of p97-UN is gated by a minimum ubiquitin chain length of five K48-linked ubiquitin, consistent with previous observations for replisome disassembly (Fujisawa et al, 2022).

### FAF2 forms a stable complex with p97 in a UFD1-dependent manner

**Figure 3:**
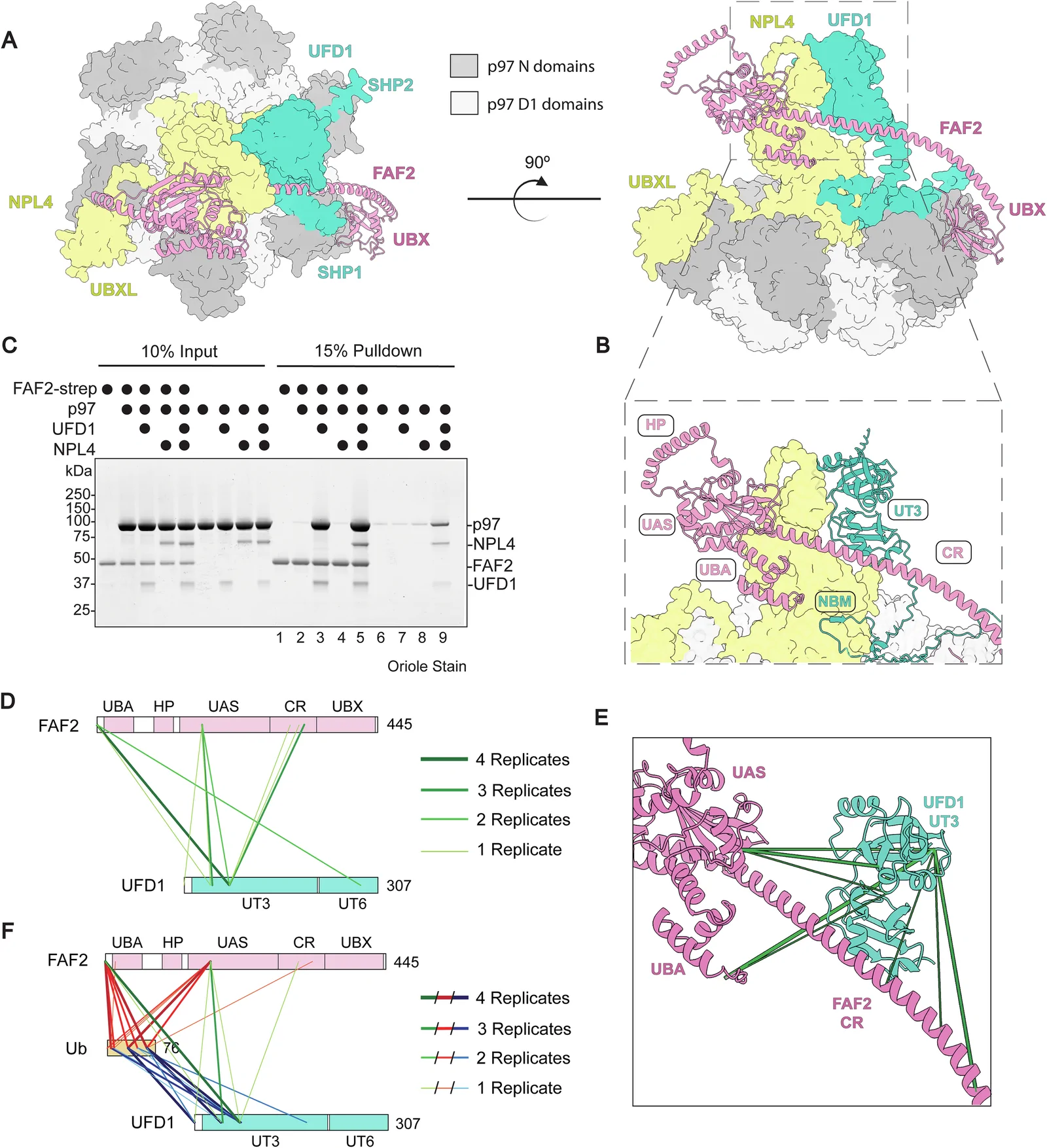
FAF2 engages the p97-UFD1-NPL4 complex through multivalent interactions. (**A**) AF3-predicted structure of the p97-ND1 hexamer bound to the UFD1-NPL4 heterodimer and FAF2. p97 N domains are shown in dark grey and D1 domains in light grey, UFD1 is shown in teal, NPL4 in yellow, and FAF2 in pink. Left, top view of the complex highlighting occupancy of p97 N domains by UBX domains and SHP motifs of cofactor proteins. Right, side view of the complex illustrating the extended architecture of FAF2 bridging the p97 N domain and the UFD1-NPL4 complex. (**B**) Close-up view of the FAF2 C-terminal region bound to UFD1-NPL4, highlighting interactions mediated by the CR of FAF2 and the UT3 domain of UFD1, while the UT6 domain interacts with NPL4. (**C**) Strep pull-down of C-term Twin Strep-tagged FAF2 performed with p97, individual UFD1 or NPL4, or the p97 and UFD1-NPL4 complex together, followed by Oriole staining, showing that FAF2 binding requires both p97 and UFD1. (**D**) Crosslinking of p97: UFD1-NPL4: FAF2 ternary complex with DSSO crosslinker. All detected crosslinks between the components are shown for *n* = 4 replicates. The colour and width of the crosslinks indicate the reproducibility across different replicates. (**E**) Crosslinks from (**D**) were mapped onto the AF3 model of the p97: UFD1-NPL4: FAF2 ternary complex. The FAF2 and UFD1 structures are highlighted to illustrate the crosslinks between them. (**F**) Crosslinking of p97: UFD1-NPL4: FAF2 ternary complex in the presence of polyUb substrate (free K48-linked Ub_6_-Ub_8_ chains) with DSSO crosslinker. All detected crosslinks between the components are shown for *n* = 4 replicates. The colour and width of the crosslinks indicate the reproducibility across different replicates. Source data are available online for this figure.

To uncover how FAF2 forms a complex with p97, we used AF3 to model the p97-UN-FAF2 complex, incorporating the p97 ND1 hexamer, UFD1, NPL4, and FAF2. All predicted models show a consistent interaction pattern in which three p97 protomers engage adaptors bound to the N domain through the UBX-L domain of NPL4, the UBX domain of FAF2, and the SHP1 and SHP2 motifs of UFD1 (Figs. 3A and EV2B). Of note, the FAF2-UBX domain is predicted to bind the N domain of the same p97 protomer where the SHP1 motif of UFD1 engages, whereas the SHP2 motif of UFD1 interacts with the adjacent p97 protomer. The N domains are modelled in the ‘up’ conformation, resembling the state when ATP is bound to the D1 domain. The models position the NPL4 tower on top of the p97 hexamer, with UFD1 located adjacent to it through interactions with the MPN domain of NPL4. FAF2 is positioned on the same side of the hexamer, where the CR bridges the p97 N domain to the UFD1-NPL4 complex (Fig. 3A). Notably, the FAF2 CR contacts the UT3 region of UFD1 but makes no direct contact with NPL4, and the interacting residues are evolutionarily conserved (Fig. EV2C). The UBA, HP and UAS domains of FAF2 do not engage the p97-UN complex in the absence of substrate (Fig. 3B).

To experimentally test the verity of the prediction, we performed in vitro pulldown assays using Twin Strep-tagged FAF2 and assayed FAF2 binding to p97 by itself or in the presence of additional components. This revealed that FAF2 pulled down only minimal amounts of p97 when incubated alone or with NPL4 (Figs. 3C, lanes 2 and 4 and EV2D). In contrast, the presence of UFD1 markedly increased p97 binding to FAF2, whereas the presence of the UN complex did not further enhance the interaction (Figs. 3C, lanes 3 and 5 and EV2D). These results indicate that UFD1 is both necessary and sufficient to mediate FAF2 recruitment to the p97 complex, consistent with the CR of FAF2 engaging the UT3 region of UFD1 in the structural model. Importantly, under the same conditions, no interaction between FAF2 and UFD1 was detected in the absence of p97, as the pulldown of FAF2 was unable to co-purify UFD1 when p97 was not added (Fig. EV2E), suggesting that a multivalent interaction network stabilises FAF2 binding to the assembled p97-UN complex. These results were further confirmed by mass photometry, which revealed the formation of a 1:1:1 complex of hexameric p97 with UFD1, NPL4 and FAF2 (Fig. EV2F).

To get more insights into the molecular interactions that enable complex formation between FAF2 and p97-UN, we analysed p97-UN-FAF2 complexes by cross-linking mass spectrometry (XL-MS). In line with structural models, multiple crosslinks are detected between the N domain of p97 and all the other components, including crosslinks to the UT6 domain of UFD1 and the UBX-L and ZF-NPL4 domains of NPL4 (Fig. 3D,E; Appendix Fig. S1). The D1 domain of p97 also makes contacts with the UT6 domain of UFD1 and the MPN domain of NPL4. The UT6 domain of UFD1 makes multiple crosslinks to the MPN domain of NPL4. The UT3 domain of UFD1 makes interactions with the MPN domain of NPL4 and the UAS domain of FAF2. Crosslinks are detected between the CR of FAF2 and the UT3 domain of UFD1 primarily in the absence of ubiquitin but seem to be reduced in the presence of ubiquitin, suggesting conformational rearrangements upon substrate engagement. Of note, very few peptides were detected in the CR region, likely due to the amino acid composition in this stretch, which limits resolution in this region. The crosslinks between the rest of the components do not appear to alter significantly in the absence or presence of ubiquitin. When present, ubiquitin forms extensive crosslinks with the MPN domain of NPL4, the UT3 domain of UFD1, and the UAS and CR of FAF2, suggesting it engages multiple domains of adaptors to coordinate substrate engagement (Fig. 3F; Appendix Fig. S1).

**Figure 4:**
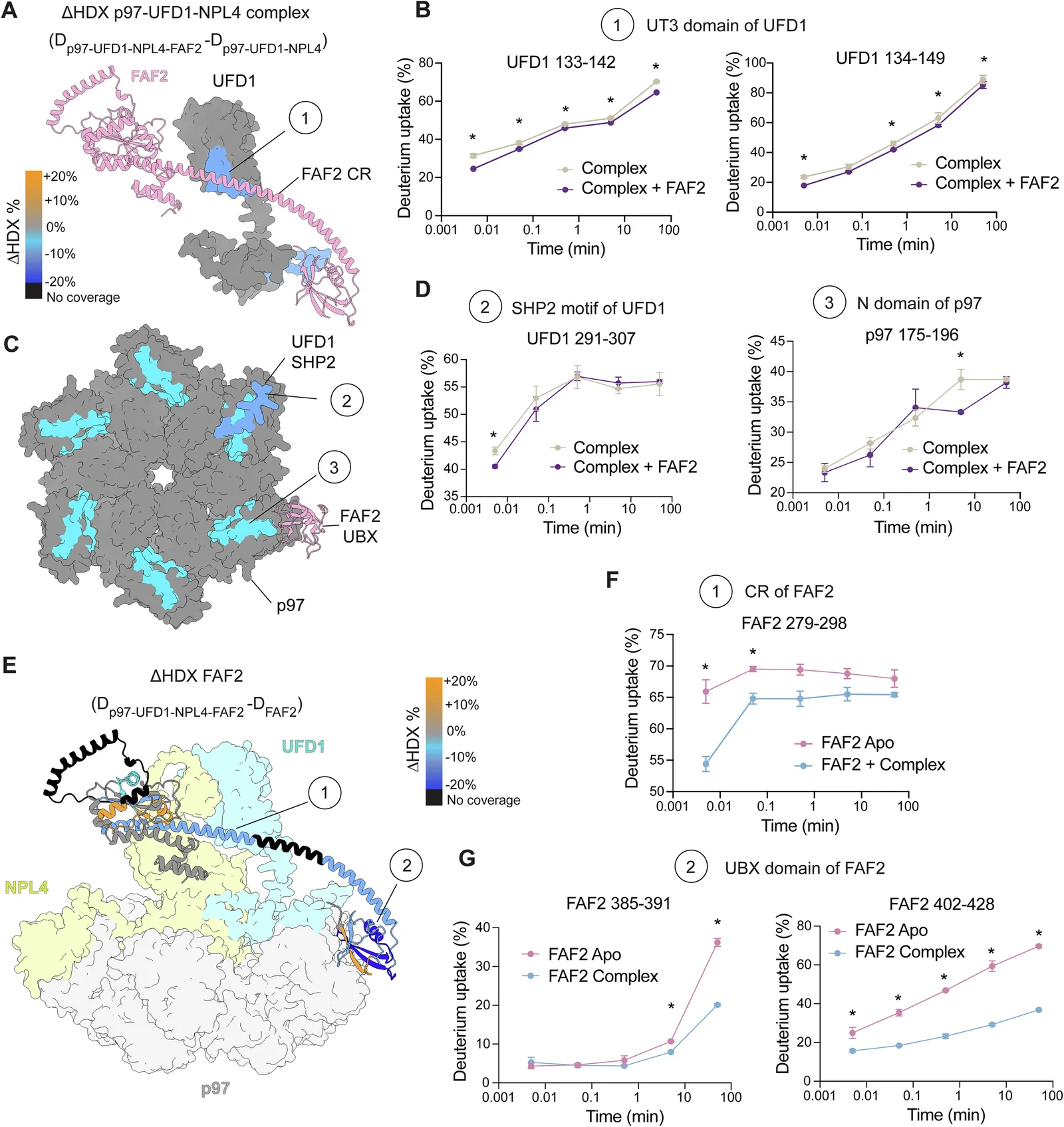
Hydrogen-deuterium exchange (HDX-MS) analysis of FAF2 interactions with the p97-UFD1-NPL4 complex. (**A**) HDX-MS was performed with p97:UFD1-NPL4 complex in the absence or presence of FAF2 at different time points. Regions in UFD1 which exhibited a reduction of solvent exchange upon FAF2 binding are highlighted. Regions with reduced solvent exchange are shown in blue, increased exchange in orange, no coverage in black, and no significant change (−5% to +5%) in grey. (**B**) Deuterium uptake kinetics of selected peptides in the UFD1 UT3 domain showing FAF2-dependent changes. Data represent mean ± SD (*n* = 3 independent replicates). Statistical significance between conditions at each labelling time point was calculated by Student’s *t*-test. For peptide 133–142 0.3 s, *p* = 0.000394; 3 s, *p* = 0.003231; 30 s, *p* = 0.002706; 300 s, *p* = 0.000679; 3000 s, *p* = 0.000045. For peptide 134–149 0.3 s, *p* = 0.000605; 3 s, *p* = 0.031355; 30 s, *p* = 0.009327; 300 s, *p* = 0.084807; 3000 s, *p* = 0.140234. (**C**) ΔHDX mapping of p97 and the UFD1 C-terminus upon FAF2 binding using the same colour scheme as in (**A**). (**D**) Deuterium uptake kinetics of selected peptides within the UFD1 SHP2 motif and corresponding binding sites in the p97 N domains. Data represented as mean ± SD (*n* = 3 independent replicates). Statistical significance was calculated by Student’s *t*-test. (UFD1 peptide 291–307 0.3 s, *p* = 0.003086; p97 peptide 175–196 300 s, *p* = 0.005476). (**E**) HDX-MS analysis of FAF2 in the apo state or bound to the p97:UFD1-NPL4 complex, displayed using the colour scheme described in (**A**). (**F**, **G**) Differential deuterium uptake of selected FAF2 peptides in the CR and UBX domain upon complex formation. Data represent mean ± SD (*n* = 3 independent replicates). Statistical significance was calculated by Student’s *t*-test. (Peptide 279–298 0.3 s, *p* = 0.000857; 3 s, *p* = 0.000005; Peptide 385–391 300 s, *p* = 0.000275; 3000 s, *p* = 0.000013; Peptide 402–428 0.3 s, *p* = 0.005802; 3 s, *p* = 0.000076; 30 s, *p* = 0.000005; 300 s, *p* = 0.000051; 3000 s, *p* ≤ 0.000001. * represents >5% and >0.5 Da difference and passes a student *t*-test with *p* = 0.05 (or lower). Source data are available online for this figure.

Hydrogen deuterium exchange (HDX-MS) analysis uncovered additional insights into the dynamic behaviour of the p97-UN-FAF2 complex. We generated a comprehensive peptide map of the p97-UN complex and assessed how FAF2 binding alters solvent exchange across individual components of the complex (Appendix Table S1). Notably, FAF2 association resulted in a pronounced reduction of solvent exchange within the UT3 domain of UFD1 (residues 133–149), consistent with direct engagement of FAF2 at this site and in strong agreement with the AF3 prediction (Fig. 4A,B). Interestingly, FAF2 binding also led to reduced solvent exchange within the UFD1 SHP2 motif (Fig. 4C,D). AF3 predicts that UFD1-SHP1 and FAF2-UBX bind distinct sites on the same p97 N domain, whereas the SHP2 motif interacts with a neighbouring N domain (Fig. 3A). The reduced solvent exchange observed in this region suggests that either it engages the same p97 N domain as FAF2 or FAF2 binding induces conformational rearrangements to stabilise UFD1 on p97 via the SHP2 motif. Similarly, corresponding regions of the p97 N domain, where both the SHP2 motif of UFD1 and the UBX domain of FAF2 bind, also exhibited a reduction in solvent exchange. In addition, regions in the CTD of NPL4 also showed minor protection from solvent exchange in the presence of FAF2 (Fig. EV2G). One limitation of this analysis is that changes in p97-derived peptides occurring on one protomer of p97 were diluted by sixfold due to stoichiometric effects, likely underestimating the effect of FAF2 binding on p97 conformation.

Notably, HDX-MS analysis also revealed a key aspect of FAF2 secondary structure. While AF3 predicted the central region between the UAS and UBX domain of FAF2 (residues 275–348) to be a long α-helix, HDX-MS analysis of FAF2 in the apo state shows a very rapid solvent exchange for peptides in this region even at very early time points (0.3 s), a feature typical of an intrinsically disordered region (Fig. 4E). Remarkably, when FAF2 binds the p97-UN complex, deuterium uptake in the peptides of this region decreases markedly, indicating that this region adopts a more ordered secondary structure, likely consistent with the α-helical structure predicted by AF3 (Fig. 4E,F). Additionally, peptides in the FAF2 CR, which bind the UT3 domain of UFD1, and FAF2-UBX, which engages the p97 N domain, showed decreased solvent exchange, further validating the structural model (Fig. 4G). However, amino acid composition within the UFD1 binding interface did not allow full coverage of this region (Appendix Table S1). Nevertheless, the differential solvent exchange properties of detected peptides clearly demonstrate how FAF2 induces coordinated stabilisation across UFD1 and p97 N domains through multivalent interactions.

### FAF2-UFD1 interactions are essential for substrate unfolding

**Figure 5:**
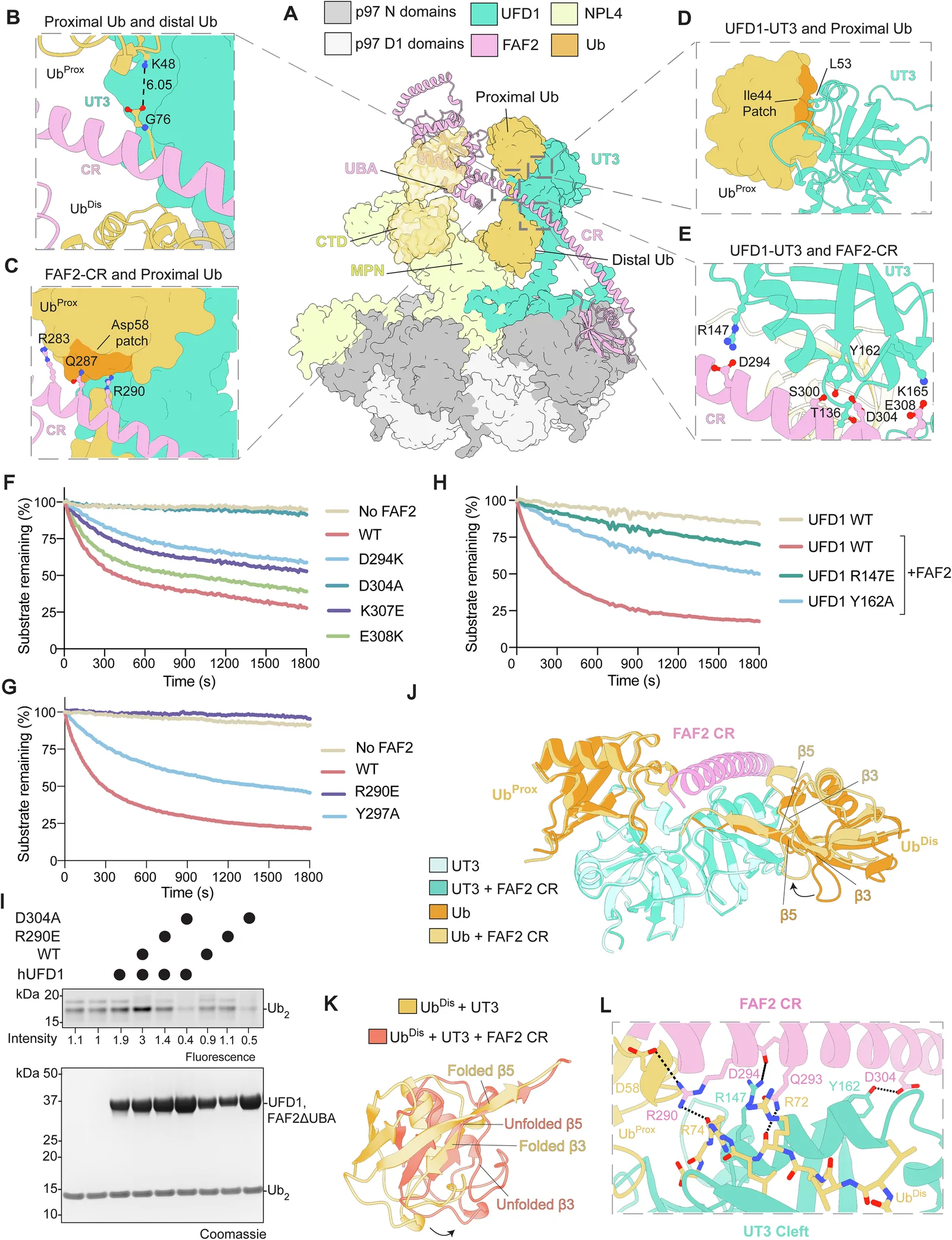
Coordinated ubiquitin engagement and initiator unfolding by FAF2 and UFD1 enables p97-mediated substrate unfolding. (**A**) AF3 model of p97 ND1 hexamer, UFD1-NPL4, FAF2 and ten ubiquitin molecules. The model shows binding of four ubiquitins across various ubiquitin-binding domains of FAF2, UFD1 and NPL4. (**B**) Inset showing two ubiquitin molecules bound to UFD1 and FAF2 in a K48-linked conformation. (**C**) Enlarged view of the FAF2-proximal ubiquitin, illustrating the FAF2(CR)-Asp58 patch interaction. (**D**) The UFD1-proximal Ub interface shows engagement with the Ile44 patch of ubiquitin. (**E**) UFD1 UT3 and FAF2 CR interface remain mostly unaltered in the presence of ubiquitin. The residues mediating interactions are shown as ball and stick. (**F**,** G**) Analysis of unfolding activity of FAF2 mutants predicted by AF3 to disrupt UFD1 binding, measured in the presence of UFD1-NPL4. (**H**) Unfolding activity of UFD1 mutants predicted by AF3 to impair interaction with FAF2 binding, measured in the presence of FAF2. For (**F**–**H**) traces were normalised to fluorescence intensity at time 0 and substrate-only controls. Data were shown for *n* = 4 technical replicates. (**I**) Unfolding of K48-linked I3C di-ubiquitin was monitored by labelling of the cysteine residue of unfolded ubiquitin with DyLight800 maleimide. Top: Fluorescence scan of the samples showing labelling efficiency in the presence of the indicated proteins. Bottom: Coomassie Blue staining of the corresponding samples showing the proteins included in each reaction mixture. (**J**) Structural alignment of AF3 predicted complex of 2xUb, UFD1-UT3 in the absence and presence of FAF2 CR (275–320). (**K**) Structural alignment of distal ubiquitin shows conformational changes in the presence of FAF2 CR. (**L**) Analysis of interactions between partially unfolded distal Ub and UFD1-UT3 and FAF2 CR. Source data are available online for this figure.

**Figure EV3:**
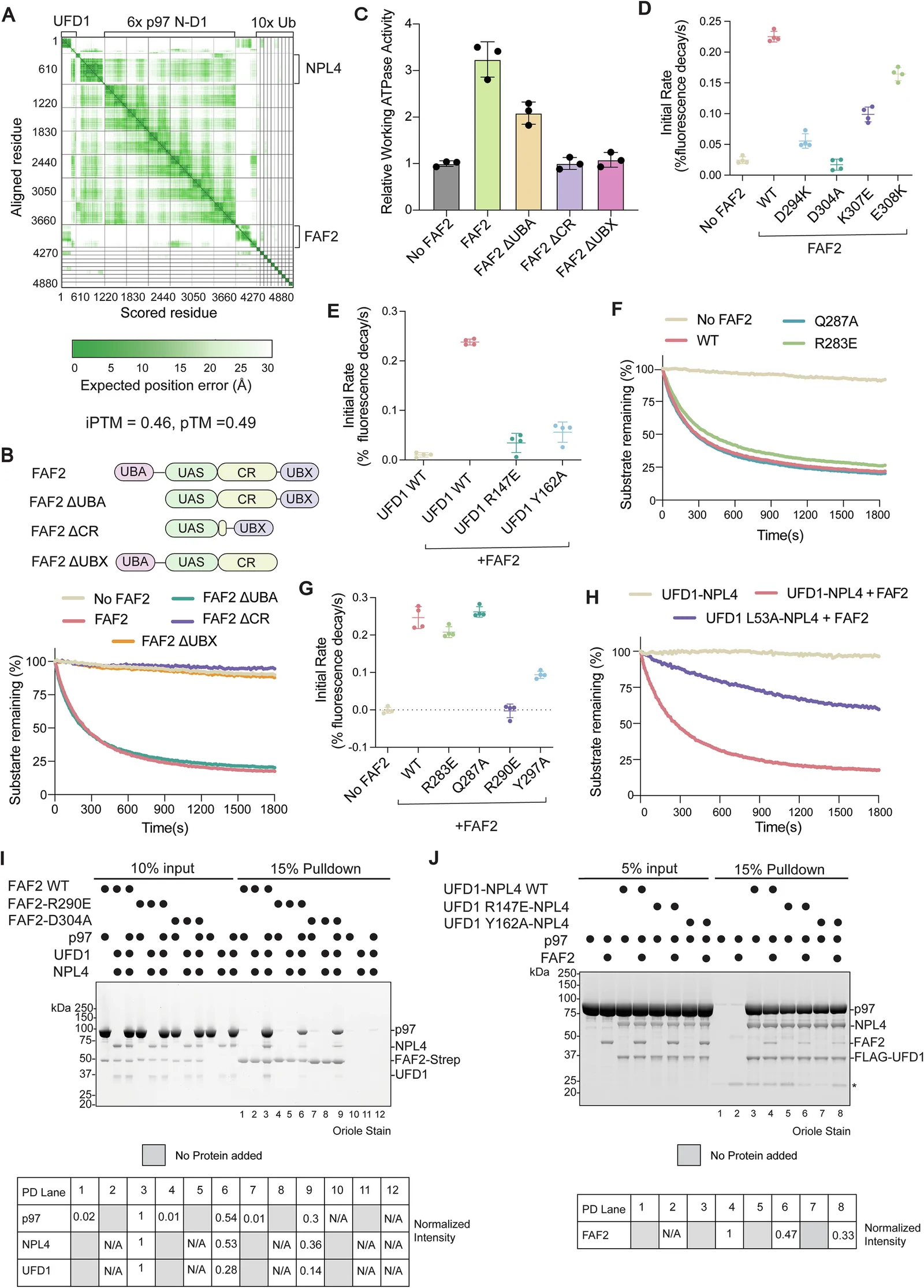
Analysis of FAF2, UFD1 and ubiquitin interactions. (**A**) PAE plot for the AF3 model of p97(ND1)-UFD1-NPL4-FAF2 with ten ubiquitin molecules. (**B**) Top: Schematic representation of FAF2 truncation constructs used. Bottom: Unfolding of Ub_6_-Eos by p97-UFD1-NPL4 in the presence of WT FAF2 or FAF2 truncation variants. (**C**) ATPase activity of p97 in the presence of the indicated FAF2 truncations and Ub^L^ substrate, normalised to ATPase activity of p97 in the presence of the UFD1-NPL4 complex and substrate. Data represent the mean ± SD (*n* = 3 technical replicates) and are extended from the data shown in Fig. 2F. (**D**) Comparison of substrate unfolding kinetics for FAF2 mutants predicted to impair UFD1 interactions. (**E**) Comparison of substrate unfolding kinetics for UFD1 mutants predicted to impair FAF2 interactions. (**F**,** G**) Comparison of substrate unfolding kinetics for FAF2 mutants predicted to impair UFD1 and ubiquitin interactions. (**H**) Unfolding activity of FAF2 mutant predicted to abrogate interaction with proximal Ub, measured in the presence of UFD1-NPL4. (**I**) Streptavidin pulldown assays assessing interactions between FAF2 (WT and indicated mutants) and the p97-UFD1-NPL4 complex. Intensities of each protein were normalised to the amount of bait (FAF2) recovered in each pulldown and to the amount pulled down with WT FAF2. (**J**) FLAG pulldown assays assessing binding of wild-type or mutant Flag-tagged UFD1-NPL4 complexes to p97 and FAF2. The asterisk (*) indicates non-specific bands, likely resulting from the light chain of the antibody. Intensities of FAF2 were normalised to the amount pulled down with WT UFD1-NPL4. In (**B**, **F**, **H**) traces were normalised to fluorescence intensity at time 0 and substrate-only controls. For (**B**, **D**–**H**), data were shown as mean ± SD for *n* = 4 technical replicates. Source data are available online for this figure.

To gain insights into how FAF2 activates the unfolding of substrates carrying both short and long Ub chains, we performed AF3 predictions of the p97-UN-FAF2 complex together with 10 ubiquitin moieties (Figs. 5A and EV3A). This revealed multi-site interactions between polyUb and the p97 complex. In line with our XL-MS results (Fig. EV2H; Appendix Fig. S1), ubiquitin interactions with the MPN and CTD domain of NPL4, the UBA domain and CR of FAF2 and the UT3 domain of UFD1 are consistently recapitulated in four out of the five models generated (Fig. 5A). Intriguingly, two ubiquitin moieties simultaneously engaged by the UFD1 UT3 domain are positioned such that the K48 linkage of the substrate-proximal ubiquitin points towards the C-terminus of the adjacent distal ubiquitin molecule (Fig. 5B). The UT3 domain interacts with the Ile44 patch of the proximal Ub in a manner similar to that characterised for yeast Ufd1 (Williams et al, 2023) (Fig. 5D). The distal Ub binds UT3 and is positioned at the entrance of the NPL4 groove, roughly where the initiator ubiquitin would be placed and inserted, as observed in the yeast complex (Twomey et al, 2019). Importantly, the FAF2 CR sits atop this UFD1 UT3-diUb complex, making contacts mostly with the Asp58 patch of the proximal Ub and the UT3 domain (Fig. 5C,E). The FAF2 CR also contacts the K48 linkage between the proximal and distal Ub (Fig. 5B,C), thus likely stabilising the UT3-diUb interactions. The interactions between CR and UT3 remain largely unaltered in the presence of ubiquitin in the structural model. Based on the structural model and the observation that FAF2 reduces the Ub chain length threshold for substrate unfolding, we predict that ubiquitin interactions with the FAF2 UBA domain and/or the CR would be important for this function. To map the region of FAF2 critical for stimulating p97 activity, we tested three key truncations of FAF2–ΔUBA (1–137), ΔCR (296–349) and ΔUBX (362–445) in an unfolding assay (Fig. EV3B). Deletion of the UBX domain completely abolished unfolding activity, confirming that engagement with p97 via the UBX domain is essential for its function. In contrast, removal of the UBA domain had no effect on the rate and efficiency of substrate processing, indicating that direct ubiquitin binding via this domain to FAF2 is not required for its stimulatory role. Importantly, deletion of a part of the CR also fully abolished substrate unfolding activity, indicating that this region is indispensable, along with the UBX domain, for FAF2 function (Fig. EV3B). Furthermore, a similar dependence on the UBX and CR domains was also reflected in substrate-induced ATP hydrolysis by the p97-UN-FAF2 complex (Fig. EV3C).

FAF2 and UFD1 UT3 domain interactions are mediated primarily by polar electrostatic and hydrogen-bonding interactions between D294, Y297, S300, D304, K307 and E308 in FAF2 and R147, T136, Y162 and K165 in UFD1, as suggested by the AF3 model and supported by the HDX-MS analysis (Fig. 5E). These interactions are also largely conserved in the FAF1-p97-UN complex, suggesting a conserved mode of interaction between FAF1 and FAF2 (Appendix Fig. S2). To disrupt the FAF2 CR-UFD1 UT3 interaction, we introduced point mutations in FAF2 and UFD1 guided by conservation analysis across 150 eukaryotic species (Fig. EV2C). Among the mutations tested, the D304A mutant resulted in a complete loss of unfolding activity, whereas D294E, Y297A, K307E and E308K partially reduced unfolding (Figs. 5F,G and EV3D). Complementary mutations on the UFD1 UT3 domain (R147E and Y162A), which interact with D294 and E304 of FAF2, similarly impaired unfolding activation (Figs. 5H and EV3E). Consistent with these functional defects, the FAF2 D304A mutant markedly reduced binding to the p97-UN complex (Fig. EV3I). Likewise, mutation of R147 and Y162, the residues in UFD1 that mediate contacts with D294 and D304 of FAF2, partially disrupted FAF2 binding by the p97-UN complex (Fig. EV3J).

**Figure EV4:**
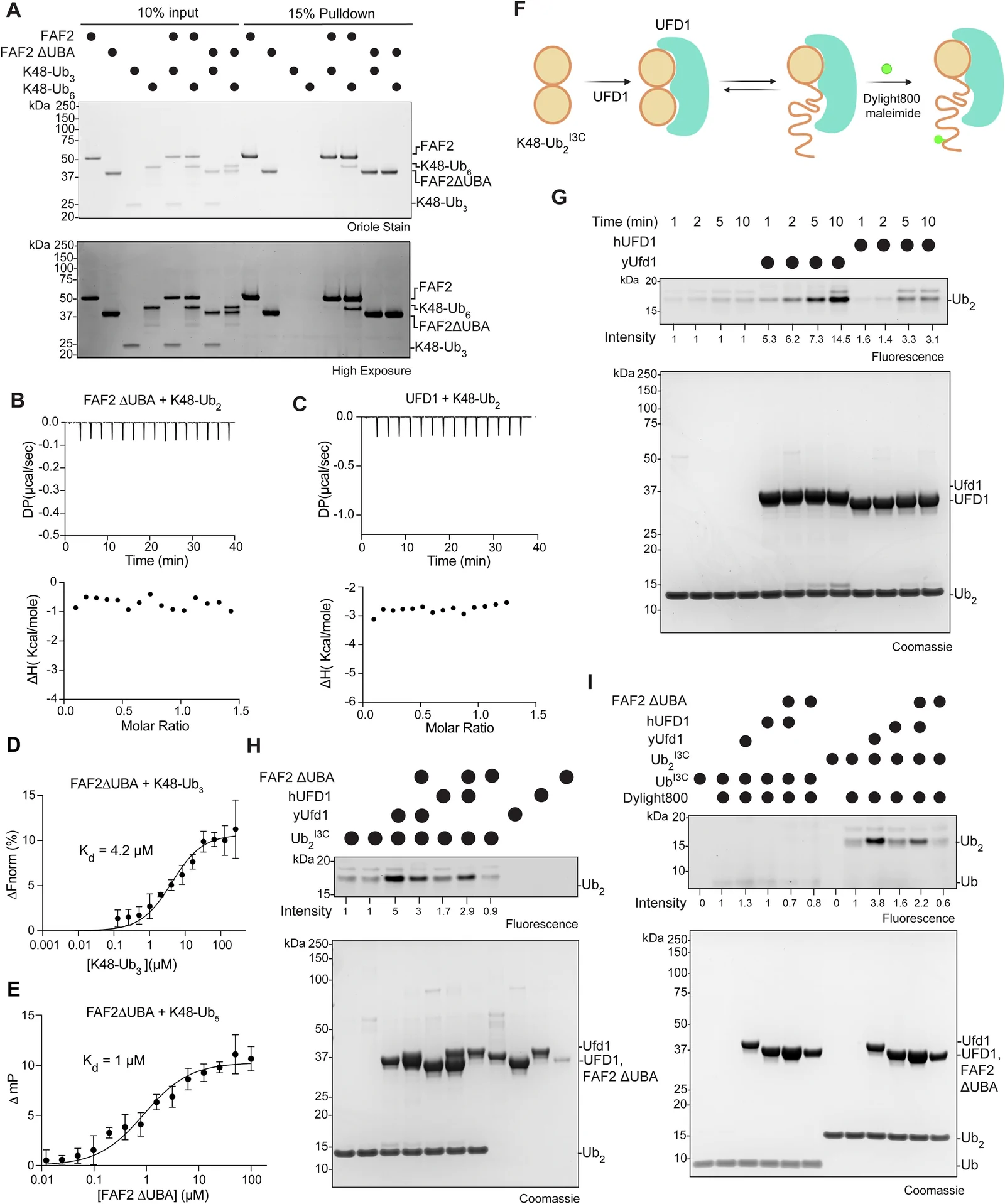
Analysis of ubiquitin binding and unfolding by UFD1 and FAF2. (**A**) Pulldown assays testing interactions between strep-tagged FAF2 and K48-linked ubiquitin chains (Ub_3_ and Ub_6_) in the absence of the UBA domain. Full-length FAF2 was included as a control. (**B**) Isothermal titration calorimetry (ITC) analysis of FAF2 ΔUBA binding to K48-linked diUb. Representative thermograms (top) and binding isotherms (bottom) are shown. (**C**) Isothermal titration calorimetry (ITC) analysis of UFD1 binding to K48-linked di-Ub. Representative thermograms (top) and binding isotherms (bottom) are shown. (**D**) Analysis of binding between FAF2 ΔUBA-Twin strep and K48-linked tri-Ub using microscale thermophoresis. FAF2 ΔUBA-Twin strep was labelled with NHS-647 dye and mixed with different concentrations of K48-Ub_3_. Data from 65 independent runs were averaged and fitted into a one-site specific binding function in Prism to calculate the dissociation constant (*K*_d_). (**E**) Analysis of binding between FAF2 ΔUBA-Twin strep and K48-linked penta-Ub using fluorescence polarisation. K48-Ub_5_ was labelled with BODIPY-maleimide dye and mixed with varying concentrations of FAF2 ΔUBA. Data were shown for *n* = 3 technical replicates. Differences in polarisation (ΔmP) was calculated by subtracting the mP value of the only tracer (K48-Ub_5_) control from that of each condition. Data were fitted into a one-site specific binding function in Prism to calculate the dissociation constant (*K*_d_). (**F**) Schematic of the dye accessibility assay. UFD1-bound K48-linked I3C di-ubiquitin undergoes unfolding and exposure of the cysteine residue, which is covalently labelled with DyLight800 maleimide, providing a cumulative readout of ubiquitin unfolding over time. (**G**) Time-course of K48-linked I3C di-ubiquitin unfolding measured by DyLight800 maleimide labelling of the exposed cysteine residue in the presence of yeast Ufd1 or human UFD1. Fluorescence scan (top) and Coomassie staining (bottom) of the corresponding samples are shown, with relative band intensities indicated. (**H**) As in (**G**), testing the ability of FAF2ΔUBA to enhance unfolding of K48-linked I3C di-ubiquitin by yeast and human UFD1. Fluorescence scan (top) and Coomassie staining (bottom) of the corresponding samples are shown. (**I**) Comparing unfolding of K48-linked I3C di-ubiquitin versus I3C monoubiquitin in the presence of FAF2ΔUBA and UFD1 homologues. Fluorescence scan (top) and Coomassie staining (bottom) of the corresponding samples are shown. Source data are available online for this figure.

Additionally, mutation of FAF2 R290, which contacts the K48 linkage in the AF3 model, fully abolished the unfolding stimulation, whereas others showed no significant effect (Figs. 5G and EV3F,G). Unexpectedly, the R290E mutation also abrogates the interaction of FAF2 with the p97-UN complex in the absence of ubiquitin (Fig. EV3I). This finding raises the possibility that R290 in FAF2 adopts a slightly different conformation upon ubiquitin binding, enabling simultaneous recognition of UFD1 and ubiquitin by the UT3 domain. Mutation of UFD1 L53 to alanine, which engages the proximal ubiquitin in our model, strongly reduced the unfolding activity, supporting the predicted positioning of ubiquitin in the complex (Figs. 5D and EV3H). We next analysed the ubiquitin binding properties of FAF2, which revealed that the UBA domain is the main binder. When deleted, FAF2 ΔUBA does not show any detectable binding to either Ub_3_ or Ub_6_ in pulldowns or show any detectable binding to Ub_2_ by ITC (Fig. EV4A,B). Similarly, UFD1 also does not show any detectable binding to K48-linked Ub_2_ (Fig. EV4C). However, when binding to longer chains was measured by microscale thermophoresis (MST) or fluorescence polarisation, FAF2 ΔUBA bound Ub_3_ and Ub_5_ with dissociation constants of 4.2μM and 1μM, respectively (Fig. EV4D,E). Collectively, these observations demonstrate that the CR-mediated FAF2-UFD1 interactions, together with FAF2-ubiquitin interactions, are critical for promoting efficient substrate unfolding.

### FAF2 activation motif supports initiator unfolding by UFD1

A recent preprint demonstrated that yeast Ufd1 directly catalyses unfolding of the initiator ubiquitin in an ATP-independent manner, where the UT3 domain of Ufd1 simultaneously engages K48-linked di-ubiquitin via its conserved ridge and cleft sites (preprint: Wang et al, 2026). This interaction actively unfolds the distal/initiator ubiquitin moiety by capturing its C-terminal β5 strand within the UT3 cleft and disrupting the highly stable β-grasp fold of ubiquitin for subsequent insertion into the NPL4 groove, providing an explanation to how substrate unfolding is initiated. To test whether human UFD1 also similarly catalyses initiator ubiquitin unfolding, we employed the dye accessibility assay (preprint: Wang et al, 2026) in which an I3C mutation within ubiquitin serves as a reporter of its folding state. In the folded state, the I3C residue is buried and inaccessible, but upon unfolding becomes exposed and covalently labelled by a fluorescent maleimide dye, allowing direct quantification of unfolding by fluorescence scanning (Fig. EV4F). Surprisingly, human UFD1 showed markedly reduced unfolding of initiator ubiquitin in this assay compared to its yeast counterpart, and even with extended reaction times, the unfolding efficiency remained negligible (Fig. EV4G), likely accounting for the differences in substrate unfolding activity between human and yeast p97-UN complexes.

Since the cooperative di-Ub engagement by yeast Ufd1 reported by Wang et al is recapitulated in our AF3-predicted complex of p97-UN, polyUb, and the FAF2 CR (Fig. 5A), we wondered if FAF2 can influence initiator ubiquitin unfolding by human UFD1. Indeed, the addition of FAF2 in this reaction stimulated ubiquitin unfolding, indicating that FAF2 compensates for this limitation of the human complex (Fig. 5I). This unexpected observation prompted us to run AF3 predictions of human UFD1-UT3 (1-196) in complex with two Ub molecules either in the presence or absence of FAF2 CR (275–320). Intriguingly, when FAF2 CR was included, the distal or initiator Ub shows a markedly unfolded conformation where β-augmentation between Ub β5 and the UT3 cleft results in destabilisation of Ub, as evidenced by significant structural rearrangement of β3 (Fig. 5K; Appendix Fig. S3). We suggest that this partially unfolded state is stabilised by interactions between UFD1 and FAF2 CR (Fig. 5J,L).

In line with these predictions, the UBA domain of FAF2, which is the main Ub binding module of FAF2 (Fig. EV4A) was not important for initiator unfolding, whereas the CR is critical as mutation of R290, which contacts both D58 of the proximal Ub and R74 of the unfolded initiator Ub, disrupts both initiator ubiquitin unfolding and substrate unfolding by p97-UN complex (Fig. 5G,I). Moreover, FAF2 CR did not enhance unfolding of di-ubiquitin by yeast Ufd1 (Fig. EV4H). As with yeast Ufd1, ubiquitin unfolding by the human UFD1-FAF2 complex is tightly coupled to engagement via both the UT3 ridge and cleft sites. Thus, the human UFD1-FAF2 complex is unable to unfold mono-Ub like yeast Ufd1 (Fig. EV4I).

Based on our structural and biochemical data, we identify residues 286–312 of FAF2 as the minimal UFD1- and ubiquitin-interacting region required for efficient initiator unfolding and refer to this segment as the activation motif (AM). Collectively, our findings suggest that FAF2, through its AM, compensates for the intrinsic limitation of human UFD1 in catalysing initiator ubiquitin unfolding. By simultaneously contacting K48-linked di-Ub and UFD1-UT3, FAF2 AM stabilises a productive UT3 conformation that enables efficient β5 capture and destabilisation of the β grasp fold of the initiator ubiquitin to promote unfolding. This cooperative mechanism of ATP-independent initiator unfolding provides a molecular explanation for how FAF2 enhances substrate unfolding by the p97-UN complex.

### FAF2 activation motif scaffolding guides de novo design of p97 activators

Loss-of-function variants of p97, such as D395G and D592N, show reduced ATPase activity and defects in clearance of aggregates (Ju et al, 2008; Weihl et al, 2006). These observations suggest that activating p97 could facilitate the clearance of aggregation-prone proteins in cells, offering a potential therapeutic strategy for proteinopathies. Building on the principle that the FAF2 AM is essential for UFD1 binding and accelerating the unfolding reaction, we explored the idea of designing activators of the p97-UN complex through a de novo protein design approach.

**Figure 6:**
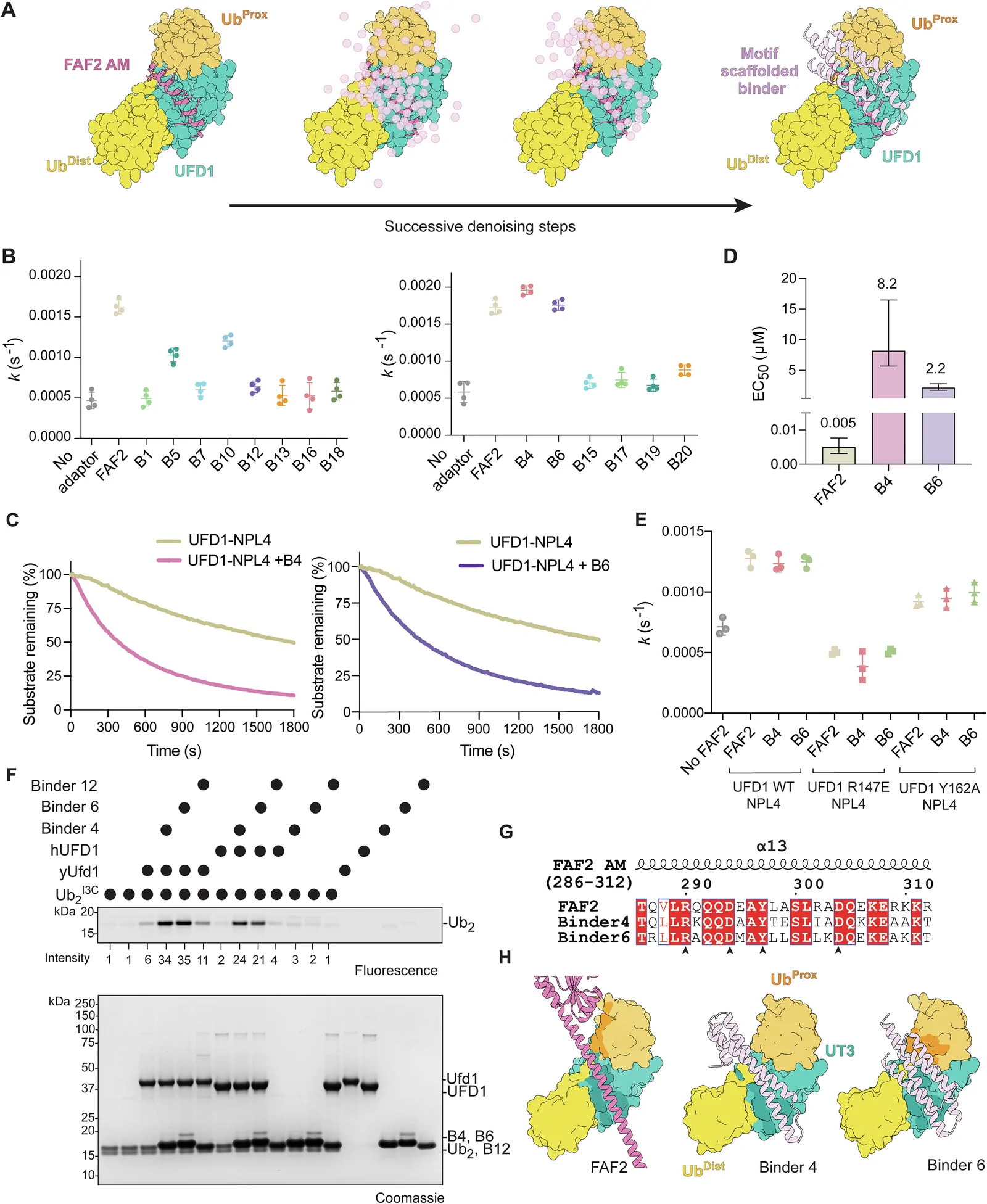
Targeting UFD1 with de novo binders enhances p97-mediated substrate unfolding activity. (**A**) De novo UFD1 binders were designed by fixing residues within the FAF2 AM (residues 286–312, dark pink) in the UFD1 (teal)-di-ubiquitin (orange) complex and building a scaffold around it (light pink) via RFdiffusion, transforming noisy residue frames into a stable backbone engaging the target. (**B**) Screening of FAF2 AM-scaffolded binders for enhancement of p97-mediated substrate unfolding in a UFD1-NPL4-dependent manner. Unfolding of a Ub^L^ substrate was measured in the presence of p97-UFD1-NPL4 and individual binders, with FAF2 as a positive reference. Rate was calculated by single exponential fitting of the trace. Data represent mean ± SD from *n* = 4 technical replicates. (**C**) Single-turnover unfolding traces of Ub^L^ in the absence or presence of representative binders show potent activation of the unfoldase complex. Data represent mean ± SD from *n* = 4 technical replicates. (**D**) Comparison of EC₅₀ values for enhancement of p97-mediated substrate unfolding by FAF2 and selected scaffolded binders, calculated from plotting the unfolding rates from data in Fig. EV5C as a function of FAF2 or binder concentration. Data represent mean ± SD from *n* = 3 technical replicates for FAF2 and *n* = 4 technical replicates for binders. Error bars denote 95% CI. (**E**) Unfolding of a Ub^L^ substrate was measured in the presence of p97, UFD1-NPL4 variants and individual binders, with FAF2 as a positive reference. The rate was calculated by single exponential fitting of the trace. Data represent mean ± SD from *n* = 3 technical replicates. (**F**) Unfolding of K48-linked I3C di-ubiquitin measured by labelling of the cysteine residue of the unfolded ubiquitin with DyLight800 maleimide. Top: Fluorescence scan of the samples showing labelling efficiency in the presence of the indicated proteins and de novo binders. Bottom: Coomassie Blue staining of the corresponding samples showing the proteins included in each reaction mixture. (**G**) Sequence alignment of the functional FAF2 motif and selected scaffolded binders, highlighting conserved residues that were kept fixed during RFdiffusion-based binder design. FAF2 numbering and secondary structure elements are shown. Black arrows indicate FAF2 residues required for UFD1 binding and enhanced unfolding activity, as suggested by the structural model and validated by mutational analysis. (**H**) AF2 models of FAF2 and engineered binders bound to the UFD1 UT3 and diUb complex. FAF2 is shown in dark pink, UT3 in teal, proximal Ub in orange and distal Ub in yellow. Binder 4 and Binder 6 engage the complex in a similar overall orientation as FAF2 and contact largely overlapping surfaces on UT3 and ubiquitin. Source data are available online for this figure.

To this end, we used RFdiffusion (Watson et al, 2023) to scaffold potential p97-activating proteins around the FAF2 activation motif (286–312). The motif scaffolding was performed in the presence of the UT3 domain of UFD1 (17–196) and two ubiquitin molecules positioned in K48-linked orientation obtained from the AF3 model of the complex as described by the AF3 model (Fig. 6A). This structural model served as the input for binder generation using RFdiffusion (Watson et al, 2023), integrated with ProteinMPNN for amino acid sequence design (Dauparas et al, 2022) and AlphaFold2 (AF2)-based structure prediction and validation (Appendix Fig. S4). Residues in the FAF2 AM predicted to be involved in UFD1 and ubiquitin interactions (T286, L289, R290, Q292, Q293, D294, A296, Y297, S300, L301, D304, Q305, K307, E308 and K311) were held fixed during design. Various N- and C-terminal extensions were then sampled, while RFdiffusion redesigned the intervening residues within the FAF2 AM helix, optimising the sequence to pack efficiently against the fixed interaction surfaces (Wang et al, 2022). This approach maintained the FAF2 AM in the three-dimensional conformation it adopts upon UFD1 binding, thereby preserving the interaction surfaces with UFD1 and ubiquitin, while allowing the surrounding scaffold to reorganise and stabilise around it. The design process generated a diverse set of candidate scaffolds, representing multiple distinct backbone trajectories. Candidates were filtered for structural confidence, resulting in a pool of binder designs that preserve the geometry required for UFD1 and ubiquitin engagement (Appendix Table S2). In total, 15 binder designs were selected for experimental validation.

**Figure EV5:**
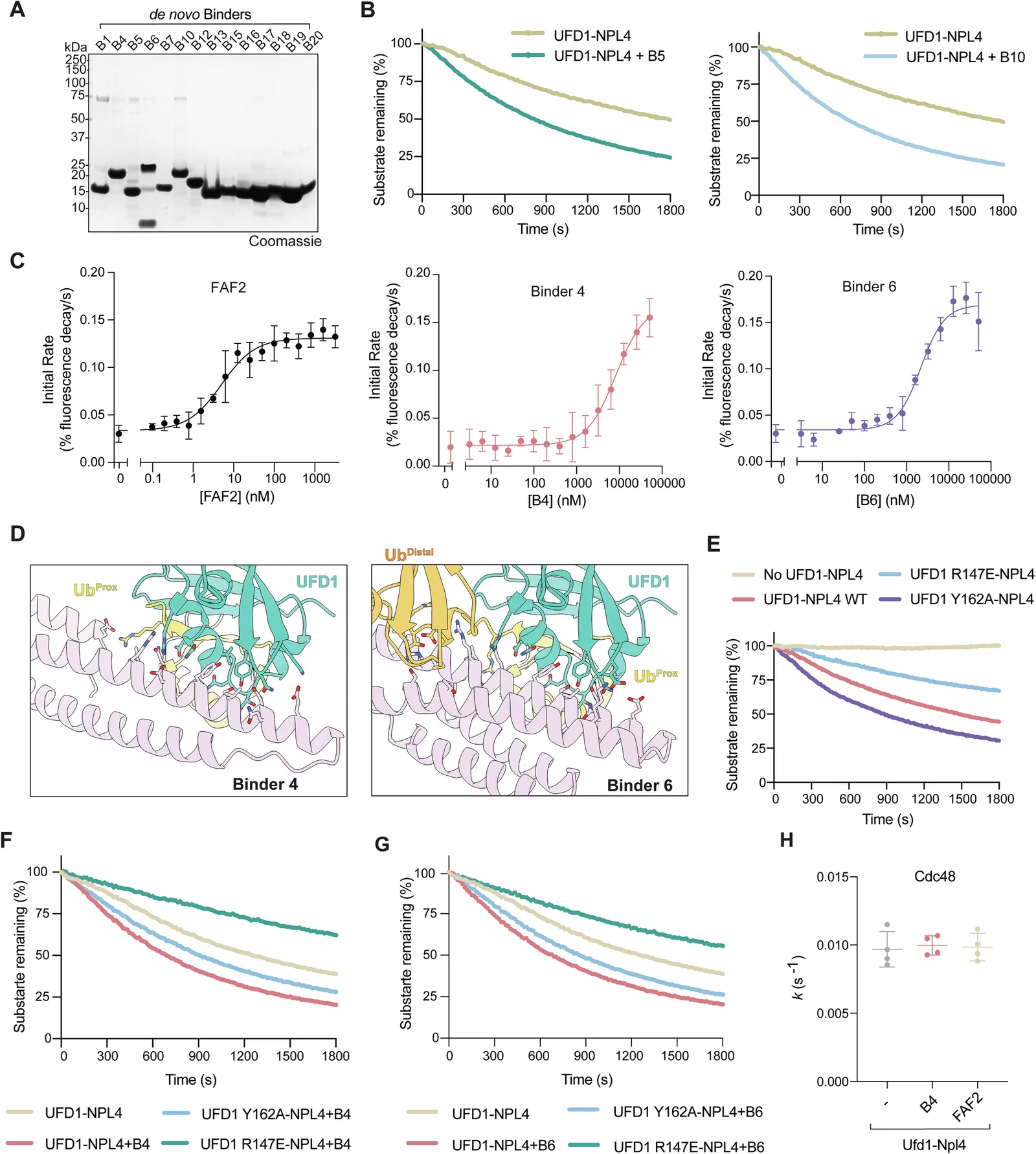
Characterisation of de novo binders. (**A**) Coomassie-stained SDS–PAGE shows 14 purified de novo binders used for experimental validation. (**B**) Unfolding activity of p97-UFD1-NPL4 complex in the presence of Binder 5 (left) and Binder 10 (right) shows modest activation of Ub^L^ substrate unfolding. Traces were normalised to fluorescence intensity at time 0 and substrate-only controls. (**C**) Initial rate of unfolding Ub^L^-Eos as a function of FAF2, Binder 4 and Binder 6 concentration. Initial rate was calculated from linear regression fitting of the first ∼2 min of the unfolding trace. Data shown as mean ± SD for *n* = 3 technical replicates for FAF2, *n* = 4 technical replicates for binders. (**D**) Structural models of UFD1 (teal) and two ubiquitin molecules bound to Binder 4 and Binder 6. Ubiquitin molecules are shown in yellow (proximal) and orange (distal), with interface residues highlighted as sticks. (**E**) Unfolding activity of WT UFD1-NPL4, UFD1·Y162A-NPL4 and UFD1·R147E-NPL4 complexes for Ub^L^ substrate. (**F**) Unfolding activity of WT and mutant UFD1-NPL4-p97 complexes in the presence of Binder 4, measured by fluorescence decay of Ub^L^-Eos substrate over time. (**G**) As in (**F**), showing the effect of UFD1 mutations on Binder 6-dependent activation of substrate unfolding. (**H**) Comparison of the unfolding rate constants of yeast Cdc48-Ufd1-Npl4 for Ub^L^ substrate in the presence of Binder 4 and FAF2. For (**E**–**H**), Traces were normalised to fluorescence intensity at time 0 and substrate-only controls. Unfolding rate *k* (s^−1^) was determined by fitting the unfolding trace to a single exponential decay. Data shown as mean ± SD for *n* = 4 technical replicates for (**E**, **H**) and mean ± SD for *n* = 3 technical replicates for (**F**, **G**). Source data are available online for this figure.

Of the 15 binders tested, 14 expressed well as soluble proteins (Fig. EV5A). We next assessed their ability to stimulate p97 activity by monitoring the unfolding of Ub^L^ substrate. Remarkably, among the 14 binders tested, 4 binders (Binder 5, Binder 10, Binder 4 and Binder 6) enhanced substrate unfolding in the presence of UFD1-NPL4 under the same experimental conditions as FAF2 (Figs. 6B and EV5B). The extent of stimulation varied across designs, with Binder 4 and Binder 6 showing the strongest effect with similar unfolding kinetics as observed with FAF2 (Fig. 6B,C). However, both binders exhibited ~500–1000-fold higher EC₅₀ values (Binder 4 EC_50_ = 8.2 µM, Binder 6 EC_50_ = 2.2 µM) compared with FAF2 (EC_50_ = 6 nM), indicating reduced potency despite achieving a similar maximal unfolding activity (Figs. 6D and EV5C). To test whether the engineered de novo activators use the same UFD1 interface as FAF2, we performed unfolding assays combining de novo activators with UFD1 mutants that were previously shown to disrupt interaction with FAF2 CR and impair unfolding. Both UFD1 R147E and Y162A impaired unfolding by Binder 4 and Binder 6 (Fig. EV5F,G). Notably, in the absence of FAF2, R147E partially impaired unfolding of the Ub^L^ substrate compared to WT UFD1, whereas Y162A showed higher activity than WT (Fig. EV5G). Nevertheless, when combined with Binder 4 and Binder 6, both R147E and Y162A showed a comparable reduction in unfolding to that observed with FAF2 CR (Figs. 6E and EV5F,G). Remarkably, both Binder 4 and Binder 6 stimulated the ATP-independent unfolding of K48-Ub_2_ by human UFD1 to a high degree, whereas B12, serving as a negative control, showed no enhanced unfolding under this condition, consistent with the substrate unfolding assay (Fig. 6F,B). However, this stimulatory activity was not restricted to only human UFD1, as Binder 4 and Binder 6 also significantly enhanced unfolding by yeast Ufd1 (Fig. 6F). Nevertheless, neither the de novo binder nor FAF2 altered the substrate unfolding kinetics of the yeast Cdc48-UN complex, highlighting that the unfolding of initiator ubiquitin is not the rate-limiting step for the yeast complex (Fig. EV5H). Sequence alignment of the de novo activators (Binder 4 and Binder 6) with FAF2 revealed strong conservation of residues within the FAF2 helical activation motif, whereas intervening residues showed great variability (Fig. 6G), further confirming that activity is primarily driven by this key interaction surface. Structural analysis of Binder 4 and Binder 6 in complex with UT3-diUb, and comparison with the corresponding FAF2-bound model, revealed that these de novo binders retain interactions with the UT3 domain as observed for FAF2. While Binder 4 shows no additional contacts with ubiquitin, Binder 6 engages both ubiquitin moieties as well as UFD1 (Figs. 6H and EV5D), explaining the lower EC_50_ for Binder 6. Together, these results demonstrate that the key UFD1-UT3 and ubiquitin-contacting residues within the FAF2 AM are both necessary and sufficient to stimulate initiator unfolding and confer activation of the p97-UN complex.

## Discussion

The core function of p97, the ATP-driven mechanical unfolding of ubiquitylated proteins, is highly conserved from yeast to human. However, human p97 has evolved into a more tightly regulated ATPase relying on cofactors that tailor p97 activity in a substrate- and context -dependent manner, which is necessary given that p97 must unfold diverse and challenging substrates across multiple subcellular compartments. This correlates with an expansion in the number of cofactors, wherein individual cofactors functionalise p97 to perform specialised functions in distinct cellular pathways (Koyano et al, 2024; Zheng et al, 2022; Ganji et al, 2023; Papadopoulos et al, 2017; Körner et al, 2025; Gwon et al, 2021; Turakhiya et al, 2018). Yet for many of these cofactors, the mechanism by which it modulates p97 activity remains poorly understood.

Despite a high degree of conservation, prior work revealed that the human p97-UN disassembles poorly ubiquitylated replisome complex differently (Fujisawa et al, 2022). We identify that this inefficiency in substrate unfolding capacity of the p97-UN complex is present even with substrates carrying long ubiquitin chains. We further show that this deficit can be rescued by a subset of UBA-UBX cofactors, which function beyond simple substrate recruitment and exhibit a marked selectivity for chain length. FAF1 and FAF2 accelerate unfolding regardless of chain length, whereas p47 and UBXN7 potentiate unfolding of long polyUb chains. This is consistent with previous reports implicating UBXN7 and FAF1 in mammalian replisome complex disassembly (Fujisawa et al, 2022). p47 is unique amongst these cofactors, having an additional SEP domain, also present in UBXN2B (p37) and UBXN2A (p47L). While UBXN2A and UBXN2B unfold substrates in a ubiquitin-independent manner (Kracht et al, 2020; Weith et al, 2018), p47 lacks this activity (Kracht et al, 2020). Intriguingly, we found that p47 partners with UN to stimulate ubiquitin-dependent substrate unfolding, challenging the prevailing model that these adaptors bind p97 in a mutually exclusive manner (Meyer et al, 2002). However, the underlying mechanism for this cooperativity and its potential cellular role requires further investigation. Notably, the membrane-anchored adaptor FAF2 supported the highest unfolding activity in our reconstituted system, possibly reflecting an adaptation to the mechanical demands of extracting substrates from the lipid bilayer, consistent with findings that FAF2 is required for membrane protein extraction from the ER and mitochondria (Huo et al, 2025). Alternatively, the reduced activity of FAF1 compared to FAF2 may be explained by differences in substrate preference (Lee et al, 2023).

Biochemical analysis of the FAF2-p97 complex further revealed that FAF2 binding to p97 is highly dependent on the presence of UN, contrasting with studies on the homologue FAF1 that reported direct FAF1-p97 interaction (Ewens et al, 2014). This suggests that FAF2 functions exclusively within the p97-UN complex to process ubiquitylated substrates. The simplest explanation would be that FAF2 works as an Ub binding adaptor, recruiting substrate via the UBA domain. Instead, we identify that the UBX domain and a small stretch of ~25 residues within FAF2 CR, which we term the activation motif, act as the critical region for FAF2-mediated enhancement of p97-UN activity. Intriguingly, we also discover that the region in and around the activation motif is unstructured in its apo state and adopts a secondary structure when bound to UFD1 and p97, likely driven by multivalent interactions with both partners. As the membrane-targeting HP motif of FAF2 lies N-terminal to the CR-UBX region, we expect the FAF2 ΔHP construct used here to behave like the full-length membrane-anchored FAF2. However, membrane tethering may provide additional spatial regulation of the complex and its substrate selection in vivo. In line with our observation, deletion of the UBA domain does not impact FAF2’s role in pexophagy (Koyano et al, 2024), lipid droplet biogenesis (Kim et al) and stress granule disassembly (Gwon et al, 2021), whereas the UBX domain and the activation motif identified in our study are critical for these processes.

It is unclear how long ubiquitin chains need to be in cells to elicit p97-dependent signalling response. K48-linked chains at steady state average only 2–7 ubiquitin units (Tsuchiya et al, 2018), raising the question of how human p97 would engage and process clients bearing such short chains. Using K48-linked ubiquitin chains of defined length, we quantitatively demonstrate that FAF2 alone is sufficient to restore the yeast-like efficiency to the human p97-UN complex, requiring a minimum of five K48-linked ubiquitin for optimal unfolding of a general reporter substrate (Twomey et al, 2019), consistent with the observations reported earlier for the mammalian replisome complex (Fujisawa et al, 2022). This chain length threshold can be partly rationalised at the molecular level. Structural analysis of the substrate-engaged human p97 complex suggests that the NPL4 tower can accommodate up to three ubiquitin moieties on the distal side of the initiator (Pan et al, 2021b, 2021a; Liao et al, 2026), while UFD1 engages the proximal and the initiator ubiquitin to confer K48 linkage selectivity (Williams et al, 2023). Together, this accounts for five ubiquitins in total, matching the threshold we observe experimentally. However, this structural and biochemical evidence does not explain why the human p97-UN complex is unable to unfold substrates even when this five-ubiquitin threshold is met.

We resolve this paradox by showing that human UFD1, unlike its yeast counterpart, is catalytically inefficient in ATP-independent initiator ubiquitin unfolding, a key step that licenses subsequent ATP-driven threading (Williams et al, 2023; preprint: Wang et al, 2026). We show that FAF2, through its activation motif, stimulates the initiator unfolding activity of human UFD1 engaged with two K48-linked ubiquitin. We believe this interaction is critical for disrupting the β-grasp fold of initiator ubiquitin, as suggested for yeast Ufd1. Importantly, the UT3 ridge and cleft sites that mediate diUb binding and unfolding are highly conserved between human and yeast (preprint: Wang et al, 2026), yet the molecular basis for this difference in catalytic activity remains unclear. It is also worth noting that FAF2 CR binds to polyUb chains containing 3 or more ubiquitin with low micromolar affinity (Fig. EV4D,E). PolyUb binding was also observed in a recent study (Huo et al, 2025). This suggests that, in addition to the interactions with the initiator and proximal Ub, FAF2 CR binds at least one other Ub. Such polyUb binding may further stabilise the ubiquitylated substrate for unfolding. However, it is unclear how the additional Ub moieties would be positioned on FAF2 CR and whether this may have any effect on substrate unfolding. Importantly, the FAF2-Ub_3_ binding model presented by Huo et al is sterically incompatible with the substrate-engaged p97-UN-FAF2 complex described here and in two independent complementary studies (Liao et al, 2026; Kracht et al, 2026), as the UFD1 UT3 domain occupies the same site on FAF2 as the predicted ubiquitin-binding site. Of note, our initiator unfolding experiments suggest that during unfolding initiation, the key Ub interactions of FAF2 are with UFD1, the initiator and its proximal Ub moiety. Future studies will have to determine the precise additional Ub binding sites on FAF2 CR and test their contribution to FAF2-potentiated substrate processing.

Additionally, we show that both FAF2 and the engineered activators use a common mechanism stabilising interactions between UFD1 and the proximal and the unfolded initiator ubiquitin to overcome the rate-limiting step of initiator unfolding. Another notable finding with the de novo design was that UBX-mediated p97 association is not strictly required for the enhanced substrate unfolding. This is partly because a helical confirmation was enforced around the AM region of engineered binders during design. It is also likely that our proof-of-principle de novo activators support additional interactions with the UFD1-Ub_2_ complex that substitute the need for p97 anchoring. This likely explains why the engineered binders show more efficient initiator unfolding than FAF2 even in the absence of p97. It also suggests that, in FAF2, the UBX domain enables localisation and increases avidity for the p97-UN complex that contributes to the transition of AM into the active helical state rather than having a direct role in initiator unfolding.

Interestingly, FAF1/FAF2 are not strictly required for unfolding of long chains, raising the question of how long chains are processed. It is possible that other adaptors, such as p47 or UBXN7, operate through a distinct mechanism to overcome the rate-limiting initiator unfolding step. Alternatively, the gradual decrease in thermodynamic stability of polyUb chains with increasing chain length accounts for more efficient initiator unfolding by UT3 for longer chains (Morimoto et al, 2015). p97 has also been implicated in processing substrates modified with branched ubiquitin linkages (Meyer and Rape, 2014; Lange et al, 2024; Suryo Rahmanto et al, 2023), and it will be important to determine whether di-Ub binding and initiator unfolding by UT3 are enhanced in the context of such alternative ubiquitin architectures.

Activation of p97 would be a desirable strategy to disassemble misfolded protein aggregates that are cytotoxic and a common feature of several proteinopathies. Indeed, p97 can disassemble protein aggregates both in vitro and in cells, and loss of p97 activity is associated with vacuolar tauopathy and ALS (Darwich et al, 2020; Meyer and Weihl, 2014). The p97 activators reported to date have emerged from screens and increase ATPase activity allosterically by targeting regulatory sites outside the D2 domain or through unknown mechanisms (Jones et al, 2024; Phan et al; Figuerola-Conchas et al, 2020; Wrobel et al, 2022). Nevertheless, these activators enhance the clearance of disease-causing aggregates from cell models. Paradoxically, several proteinopathy causing p97 mutations are activating and affect adaptor exchange (Blythe et al, 2019; Zhang et al, 2015; Bulfer et al, 2016). Our rational design of p97 activators that only work in a UFD1-dependent manner to enhance initiation of unfolding could provide advantages over non-specific activators of ATPase activity, as they only enhance unfolding of p97-UN complexes. While being a proof-of-principle demonstration, further optimisation of these de novo activators and development of peptides and small-molecule activators mimicking FAF2-mediated activation represent an attractive opportunity to modulate cellular proteostasis.

## Methods

**Table:** Reagents and tools table

Reagent/resource	Reference or source	Identifier or catalogue number
**Recombinant DNA**		
pFBHTB-6His-UBE1	MRC-PPU Reagents	DU3026
pET28a(+)-6His-UBE2R1	MRC-PPU Reagents	DU4317
pET24-Ub	MRC-PPU Reagents	DU20027
pET24-Ub ^K48R K63R^	MRC-PPU Reagents	DU23835
pET24-Ub—^1^Ub^1-72, K48R K63R^	MRC-PPU Reagents	DU67512
pET28a-gp78-UBE2G2	(Blythe et al, 2017)	N/A
pGEX6P1-GST-3C-UCHL5	MRC-PPU Reagents	DU12810
pET156P-6His-UBCH5c	MRC-PPU Reagents	DU15703
pET28a-6-His-Ub-mEOS3.2	This study	DU83546
pFL-6His-p97	This study	DU76862
pK27SUMO-14His-Smt3-UFD1	MRC-PPU Reagents	DU73315
pET15d-NPL4	MRC-PPU Reagents	DU73317
pK27SUMO-14His-Smt3-Cdc48	This study	DU76754
pK27SUMO-14His-Smt3-Ufd1	This study	DU76752
pK27SUMO-14His-Smt3-Npl4	This study	DU76771
pGEX 3 C Halo TEV NSFL1C	MRC-PPU Reagents	DU76358
pK27SUMO-14His-Smt3-FAF1	MRC-PPU Reagents	DU73318
pK27SUMO-14His-Smt3-FAF2 Δ90-118	MRC-PPU Reagents	DU73323
pK27SUMO-14His-Smt3-UBXN7	MRC-PPU Reagents	DU73329
pK27SUMO-14His-Smt3-UBXN1	MRC-PPU Reagents	DU73328
pK27SUMO-14His-Smt3-FAF2 Δ137	MRC-PPU Reagents	DU73324
pK27SUMO-14His-Smt3-FAF2 Δ90-118, Δ297-349	MRC-PPU Reagents	DU73326
pK27SUMO-14His-Smt3-FAF2 Δ90-118, Δ 358-441	MRC-PPU Reagents	DU73327
pK27SUMO-14His Smt3-FAF2 Δ90-118-3C-Twinstrep	MRC-PPU Reagents	DU76587
pK27SUMO-14His Smt3-FAF2 Δ137-3C-Twinstrep	MRC-PPU Reagents	DU76588
pK27SUMO-14His-Smt3-FAF2 Δ90-118 R290E	This Study	DU76864
pK27SUMO-14His-Smt3-FAF2 Δ90-118 R283E	This Study	DU83539
pK27SUMO-14His-Smt3-FAF2 Δ90-118 Q287A	This Study	DU76863
pK27SUMO-14His-Smt3-FAF2 Δ90-118 D304A	This Study	DU83544
pK27SUMO-14His-Smt3-FAF2 Δ90-118 D294E	This Study	DU83543
pK27SUMO-14His-Smt3-FAF2 Δ90-118 E307K	This Study	DU83541
pK27SUMO-14His-Smt3-FAF2 Δ90-118 K308E	This Study	DU83542
pK27SUMO-14His-Smt3-FAF2 Δ90-118 Y297A	This Study	DU83538
pK27SUMO-14His Smt3-FAF2 R290E Δ137-3C-Twinstrep	This Study	DU76865
pK27SUMO-14His Smt3-FAF2 D304A Δ137-3C-Twinstrep	This Study	DU83540
pK27SUMO-14His-Smt3-FLAG-UFD1	This Study	DU76867
pK27SUMO-14His-Smt3-FLAG-UFD1 Y162A	This Study	DU76870
pK27SUMO-14His-Smt3-FLAG-UFD1 R147E	This Study	DU76869
pK27SUMO-14His-Smt3-FLAG UFD1 L53A	This Study	DU76868
pET24-Ub^I3C^	This Study	DU83545
pET-28a-6His-T7-UFD1_Binder01	This study (synthesised by Twist Bioscience)	DU83270
pET-28a-6His-T7-UFD1_Binder04	This study (synthesised by Twist Bioscience)	DU83273
pET-28a-6His-T7-UFD1_Binder05	This study (synthesised by Twist Bioscience)	DU83274
pET-28a-6His-T7-UFD1_Binder06	This study (synthesised by Twist Bioscience)	DU83275
pET-28a-6His-T7-UFD1_Binder07	This study (synthesised by Twist Bioscience)	DU83276
pET-28a-6His-T7-UFD1_Binder10	This study (synthesised by Twist Bioscience)	DU83279
pET-28a-6His-T7-UFD1_Binder12	This study (synthesised by Twist Bioscience)	DU83281
pET-28a-6His-T7-UFD1_Binder13	This study (synthesised by Twist Bioscience)	DU83282
pET-28a-6His-T7-UFD1_Binder14	This study (synthesised by Twist Bioscience)	DU83283
pET-28a-6His-T7-UFD1_Binder15	This study (synthesised by Twist Bioscience)	DU83284
pET-28a-6His-T7-UFD1_Binder16	This study (synthesised by Twist Bioscience)	DU83285
pET-28a-6His-T7-UFD1_Binder17	This study (synthesised by Twist Bioscience)	DU83286
pET-28a-6His-T7-UFD1_Binder18	This study (synthesised by Twist Bioscience)	DU83287
pET-28a-6His-T7-UFD1_Binder19	This study (synthesised by Twist Bioscience)	DU83288
pET-28a-6His-T7-UFD1_Binder20	This study (synthesised by Twist Bioscience)	DU83289
**Chemicals, enzymes and other reagents**		
Q5® site-directed mutagenesis kit	New England Biolabs	E0554S
KOD Hot Start DNA Polymerase	Sigma-Aldrich	71086
In-Fusion Snap Assembly Master Mix	Takara Bio	638947
4-(2-aminoethyl) benzene sulphonyl fluoride hydrochloride (AEBSF)	Apollo Scientific	BIMB2003
Benzamidine	Apollo Scientific	1670-14-0
Tris (2-carboxyethyl) phosphine hydrochloride (TCEP)	Apollo Scientific	BIT0122
Isopropyl thio-β-D galactoside (IPTG)	Formedium	IPTG100
Roche cOmplete EDTA-free protease inhibitor cocktail	Roche	11873580001
Adenosine 5’-triphosphate (ATP)	Apollo Scientific	BIB3003
Ni2+NTA agarose	Amintra, Abcam	ab270549
Glutathione SH-4B sepharose	Amintra, Abcam	Ab270237
Anti-FLAG M2 affinity gel	Sigma-Aldrich	A2220
High-Capacity Streptavidin Agarose	Thermo Fisher	20359
DyLight800 Maleimide	Thermo Fisher	46621
Dithiothreitol (DTT)	Formedium	DTT100
BIOMOL Green	Enzo Life Sciences	BML-AK111
FLAG Peptide	Sigma-Aldrich	F3290
ATPγS tetralithium salt	Tocris	4080
DSSO	Thermo Fisher	A33545
BODIPY™ FL Maleimide	Thermo Fisher	B10250
**Software**		
Adobe Illustrator	https://www.adobe.com/products/illustrator.html	
ChimeraX	https://www.cgl.ucsf.edu/chimerax/	
GraphPad Prism	https://www.graphpad.com	
Image Studio	https://www.licorbio.com/image-studio	
MicroCal PEAQ-ITC	https://www.malvernpanalytical.com/en	

### Plasmids

A list of plasmids used in this study can be found in the Reagents and tools table. Unless otherwise stated, all plasmids used in this study were generated by PD, APR and the cloning team at the Medical Research Council Protein Phosphorylation and Ubiquitylation Unit (MRC-PPU) Reagents and Services. Plasmids are available upon request by contacting YK or at https://mrcppureagents.dundee.ac.uk/.

### Protein expression

For recombinant protein expression in *E. coli*, each plasmid was transformed into *E. coli* Rosetta (DE3) cells, which were grown in LB medium supplemented with 50 µg ml⁻¹ kanamycin or 100 µg ml⁻¹ ampicillin, as applicable. Protein expression was induced at an OD_600_ of ~0.8 with 0.5 mM IPTG, followed by overnight incubation at 18 °C. For expression of WT, mutant ubiquitin and mutant M1-Ub_2_ chains, an autoinduction medium was used instead of LB, and cultures were incubated at 25 °C for 24 h. Cells were harvested by centrifugation at 4000 rpm for 20 min at 4 °C.

Human p97 was expressed in insect (Sf9) cells using the baculovirus expression system. Sf9 cells were infected with baculovirus encoding 6xHis-p97, and protein expression was carried out at 27 °C for ~72 h, until the YFP signal indicated >60% infection. Cells were harvested by centrifugation at 2500 rpm for 30 min at 4 °C.

### Purification of human p97-UFD1-NPL4 unfoldase complex

For purification of 6×His-tagged human p97 from Sf9 cells, cell pellets were resuspended in ice-cold lysis buffer A (50 mM HEPES, pH 7.5, 200 mM NaCl, 10 mM MgCl₂, 5% glycerol, 1 mM ATP, 0.5 mM TCEP) supplemented with cOmplete EDTA-free protease inhibitor cocktail (Roche) and DNase (Sigma). Cells were lysed using a Dounce homogeniser with 20–30 strokes, followed by centrifugation at 20,000 rpm for 30 min. The clarified lysate was incubated with Ni-NTA agarose beads (Amintra) for 1 h at 4 °C, and bound protein was eluted with lysis buffer supplemented with 350 mM imidazole. The protein was further purified using a ResourceQ anion-exchange column (6 ml), eluting with a linear NaCl gradient from 150 to 750 mM. Elution fractions were concentrated, and the p97 hexamer was isolated by size-exclusion chromatography on a Superose 6 Increase 3.2/300 column in SEC buffer A (60 mM HEPES, pH 7.5, 200 mM NaCl, 0.5 mM TCEP, 10 mM MgCl₂, 5% glycerol).

To reconstitute the UFD1-NPL4 complex, cell pellets expressing each component were resuspended in an equal volume of lysis buffer B (50 mM Tris, pH 7.5, 300 mM NaCl, 0.5 mM TCEP, 20 mM imidazole) supplemented with protease inhibitor. Lysates containing His-Smt3-tagged UFD1 and untagged NPL4 were mixed at a 1:2 ratio. Cells were lysed by sonication, and the lysate was clarified by centrifugation at 20,000 rpm for 30 min. The supernatant was incubated with Ni-NTA agarose resin, after which the beads were washed, and the UFD1-NPL4 complex was eluted using lysis buffer B supplemented with 350 mM imidazole. The eluted protein was dialysed overnight in imidazole-free buffer in the presence of His-tagged Ulp1 protease to cleave the His-Smt3 tag from UFD1. The cleaved tag and Ulp1 protease were removed by incubation with Ni-NTA agarose, and the resulting flow-through was concentrated and applied to a Superdex 200 16/600 size-exclusion column equilibrated in SEC buffer A. Fractions containing both UFD1 and NPL4 were pooled, concentrated and flash frozen.

### Purification of additional p97 cofactors

All p97 cofactors were cloned with an N-terminal 14xHis or GST tag for affinity purification. Briefly, cell pellets were lysed with lysis buffer B supplemented with a protease inhibitor cocktail, followed by sonication and centrifugation at 20,000 rpm. The clarified lysate was incubated with either Ni-NTA agarose resin for His-tagged proteins or Glutathione Sepharose 4B resin (Amintra) for GST-tagged cofactors for 1 h, and bound proteins were eluted with lysis buffer containing 350 mM imidazole. The His-Smt3 tag was removed by incubation with Ulp1 protease. The GST tag was cleaved by incubation with GST-tagged TEV protease. Following tag cleavage, the cofactors were further purified on a ResourceQ anion-exchange column (6 mL) and eluted using a linear NaCl gradient from 100 to 750 mM. Finally, the pooled fractions were subjected to size-exclusion chromatography on a Superdex 75 16/600 column equilibrated in SEC buffer B (60 mM HEPES, pH 7.5, 200 mM NaCl and 0.5 mM TCEP) to isolate the purified protein.

### Purification of ubiquitin

For purification of wild-type and mutant ubiquitin, as well as mutant M1-Ub₂ chains, cell pellets were resuspended in 20 ml of Ub lysis buffer (1 mM EDTA, 1 mM AEBSF, and 1 mM benzamidine). Cells were lysed by sonication, and the lysate was clarified by centrifugation at 20,000 rpm for 30 min. The pH of the supernatant was adjusted to 4.5 by adding 7% perchloric acid, followed by overnight incubation. The mixture was clarified again by centrifugation at 20,000 rpm for 30 min at 4 °C. Ubiquitin was purified using cation-exchange chromatography on a Resource S column (6 ml) in 50 mM sodium acetate, pH 4.5, eluting with a NaCl gradient. The pH of the elution fractions was adjusted to pH 7.5 by adding 100 mM Tris-HCl pH 8.5, after which the protein was concentrated and finally buffer-exchanged into 50 mM HEPES, pH 7.5, 150 mM NaCl.

### Preparation of ubiquitylated Eos

To prepare the ubiquitylated mEOS3.2, His-tagged Ub-mEOS3.2 and His-tagged gp78-UBE2G2 fusion were purified from *E.coli* and His-tagged UBE1 was purified from insect cells. For bacterial expression, briefly, cell pellets were lysed in lysis buffer B supplemented with protease inhibitors, followed by sonication and centrifugation to obtain the clarified lysate. The lysate was incubated with Ni-NTA agarose beads for 1 h, and the beads were washed with lysis buffer. The bound protein was eluted with 350 mM imidazole. For gp78-UBE2G2, the His-tag was cleaved by incubating the protein with His-TEV protease overnight. The cleaved protein and cleaved tag were removed by reverse IMAC (Immobilised Metal Affinity Chromatography). For Ub-Eos, the His-tag was retained. The proteins were further purified using size-exclusion chromatography on a Superdex 75 16/600 column equilibrated with SEC buffer C (50 mM HEPES, pH 7.5, 150 mM NaCl and 0.5 mM TCEP).

UBE1 was expressed in Sf9 cells using the baculovirus expression system at 27 °C for ~72 h. Cells were harvested by centrifuging at 2500 rpm for 30 min. and re-suspend in lysis buffer (50 mM HEPES pH 7.5, 150 mM NaCl, 0.5 mM TCEP, 1 mM AEBSF, 1 mM Benzamidine). The lysate was passed through a Dounce homogeniser 12 times to thoroughly lyse the cells. After clarification at 20,000 rpm for 30 min 20 mM imidazole was added, and the lysate was incubated for 1 h with Ni-NTA resin. The resin was washed extensively, and bound protein was eluted from the beads using lysis buffer supplemented with 350 mM imidazole. The purified UBE1 was dialysed overnight in the presence of His-TEV to cleave the tag and further purified using a Superdex 200 16/600 size-exclusion column equilibrated with SEC buffer C.

Ubiquitylation of the Ub-Eos was carried out as described before (Blythe et al, 2017). 10 μM His-tagged Ub-Eos, 1 μM UBE1, 30 μM UBE2G2-gp78 and 400 μM ubiquitin were incubated in reaction buffer (20 mM Hepes, 150 mM NaCl and 0.1 mM TCEP) at 37 °C. Ubiquitin was added slowly during the first 6 h of the reaction, and the reaction was continued overnight. The ubiquitylated Eos were purified by incubation with Ni-NTA agarose resin equilibrated with reaction buffer supplemented with 10 mM imidazole. The beads were washed, and the bead-bound substrate was eluted with 300mM imidazole. The purified substrate was loaded onto a Superose 6 Increase 3.2/300 column in SEC buffer (20 mM Hepes, 150 mM NaCl and 0.1 mM TCEP) to separate the substrate based on Ub chain length. Substrates were pooled according to the average chain length and segregated into Ub^BT^, Ub^S^ and Ub^L^ substrates. The substrates were photoconverted using a 405 nM laser until 50% or more photoconversion was achieved.

### Preparation of Ub_6_-Eos substrate

K48-linked tetra-Ub chains were synthesised by 1.5 mM Ub, 1 μM UBE1 and 25 μM UBE2R1 in ligation buffer (40 mM Tris-HCl pH 7.5), 10 mM MgCl₂ and 10 mM ATP) at 37 °C for 4–6 h. The chains were purified using a Resource S column (6 ml) equilibrated in 50 mM sodium acetate (pH 4.5) and eluted with a linear NaCl gradient. For C-terminal capping, tetraubiquitin was incubated with 2 μM UBE1, 10 μM UBE2D3 and 100 μM L-lysine in ligation buffer at 37 °C for 16 h and purified by cation-exchange chromatography under identical conditions. N-ter capped di-Ub was prepared by using Ub ^K48R, K63R^ mutant and M1-Ub_2_ (Ub ^K48R, K63R^, Ub^1–72^. Ligation and purification were carried out as described previously (Lange et al, 2024). The capped di-Ub was conjugated to the protected tetra-Ub using 2 μM UBE1 and 20 μM UBE2R1 in ligation buffer. After 24 h, 1 μM GST-UCHL5 was added to deprotect the C-terminus, and the reaction was incubated at 30 °C for an additional 2 h. The resulting K48-linked hexamer with capped distal Ub (Ub ^K48R, K63R^) was purified using a Resource S column equilibrated in 50 mM sodium acetate (pH 4.5). The capped hexamer was subsequently loaded onto Ub-Eos using 2 μM UBE1 and 20 μM UBE2R1, and the Ub_6_-Eos substrate was purified by cation-exchange chromatography using conditions as described before.

### Synthesis of K48-linked polyUb chains

K48-linked Ub chains were assembled from 1.5 mM Ub in 40 mM Tris-HCl (pH 7.5), 10 mM MgCl₂, and 10 mM ATP at 37 °C for 4–6 h. Formation of K48 linkage was catalysed by 1 μM UBE1 and 25 μM UBE2R1. For assembly of Ub₆ or Ub₈ chains, diUb was used as the starting material. Ub chains were separated by length using cation-exchange chromatography on a Resource S column (6 ml) equilibrated in 50 mM sodium acetate (pH 4.5) and eluted with a linear NaCl gradient. Fractions were pooled according to chain length, concentrated using 3-kDa or 10-kDa MWCO centrifugal filter units (Amicon), and buffer-exchanged into 50 mM HEPES (pH 7.5), 150 mM NaCl.

### Single-turnover unfolding assay

Single-turnover unfolding assays were carried out by incubating 20 nM photoconverted red Eos substrate modified with Ub chains of various lengths, 400 nM p97, 400 nM UFD1-NPL4 and 1.6 μM of additional cofactors (p47, UBXN7, FAF1, FAF2 and UBXN1) or 1.6 μM of de novo binders in assay buffer (20 mM HEPES, pH 7.5, 150 mM KCl, 10 mM MgCl_2_ and 1 mM TCEP,1 mg/ml BSA) at 37 °C for 10 min. The unfolding reaction was initiated by the addition of 5 mM ATP. Fluorescence was monitored using Clariostar Plus plate reader (BMG Labtech) at 37 °C. Unfolding was measured by monitoring loss of fluorescence at 590 nM upon excitation at 540 nM. The percentage of folded substrate remaining at each time point was calculated by normalising to the fluorescence at time 0 and to changes in fluorescence observed for the substrate-only control. The unfolding rate (*k*, s^−1^) was calculated by fitting the unfolding traces to a single exponential decay. The initial unfolding rate was determined by linear regression of the fluorescence decay data from the first 2 min of the reaction. Rates are reported as the slope of the linear fit in units of % fluorescence decay per second.

EC_50_ values for cofactor-mediated activation were determined by performing single-turnover unfolding assays with varying concentrations of FAF1, FAF2 or de novo binders while maintaining constant concentrations of p97, UFD1-NPL4 and ubiquitylated fluorescence substrate as described above. Initial (approximately first 2 min) rates of unfolding were plotted as a function of log_10_ or log_2_ cofactor concentration and fit to a four-parameter logistic curve in GraphPad Prism to extract EC_50_ values.

### ATPase assay

ATPase assay was carried out using 10 nM p97, 100 nM UFD1-NPL4, 100 nM FAF2 and 100 nM Ub^L^ substrate or 500 nM free K48-linked Ub chains of specific length in assay buffer (20 mM HEPES, pH 7.5, 150 mM KCl, 10 mM MgCl_2_, 1 mM TCEP and 0.1 mg/ml BSA). Proteins were mixed and incubated in a 384-well microplate (Grenier 781101) at 37 °C for 10 min followed by the addition of 200 μM ATP to initiate the reaction. Reactions were incubated at 37 °C for 30 min to allow ATP hydrolysis. The reactions were stopped by the addition of 50 µl of BIOMOL Green (Enzo Life Sciences) and further developed at room temperature for 1 h. Absorbance was recorded at 620 nM using Clariostar Plus plate reader (BMG Labtech). Inorganic phosphate released from each reaction was quantified based on a phosphate standard curve. Relative ATPase activity was determined by normalising the measured activity of each sample to that of the control.

### Dye accessibility assay

To generate K48-linked I3C di-ubiquitin, 1.5 mM Ub^I3C^ was incubated with 1 µM UBE1, 25 µM UBE2R1 and 10 mM ATP at 37 °C in 40 mM Tris-HCl (pH 7.5), 10 mM MgCl₂, for 3 h. The di-ubiquitin was further purified using a Resource S column (6 ml) as described before. The dye accessibility assay was performed following the protocol as mentioned in (preprint: Wang et al, 2026) with a few modifications. 10 µM of the Ub^I3C^ di-ubiquitin was incubated with 20 µM yeast Ufd1/human UFD1, 10 µM FAF2/de novo binders in 20 mM Hepes, pH 7.5 and 150 mM NaCl at 37 °C for 10 min to allow binding. Then DyLight800 maleimide (Thermo Fisher) dye was added to the reaction mixture and incubated for 5 min to allow labelling. The labelling reactions were quenched by 20 mM dithiothreitol (DTT). The samples were mixed with 4X LDS sample buffer and analysed by SDS-PAGE, followed by fluorescence scanning on an Odyssey imager (LI-COR) and Coomassie blue staining.

### In vitro pulldown of p97-UN-FAF2 complex

Recombinant FAF2-Twin Strep-tagged, UFD1-NPL4 and p97 (hexamer) proteins were preincubated on ice for 30 min in binding buffer (20 mM HEPES, pH 7.5, 150 mM NaCl, 10 mM MgCl_2_, 1 mM ATP and 0.5 mM TCEP) at a final concentration of 5 µM in a 1:1 molar ratio. The resulting complexes were incubated with 10 µL of pre-equilibrated Pierce™ High-Capacity Streptavidin Agarose (Thermo Fisher) in pulldown buffer (20 mM HEPES, pH 7.5, 150 mM NaCl, 10 mM MgCl_2_, 0.1 mM ATP, 0.5 mM TCEP and 0.5% NP-40) on a roller at 4 °C for 1 h. Beads were washed six times with ice-cold pulldown buffer. Bead-bound protein was eluted with 2× LDS sample buffer and analysed by SDS-PAGE followed by oriole stain.

For FLAG pulldown, recombinant FLAG-tagged UFD1-NPL4 complex, FAF2 and p97 were incubated under the same conditions as described above. The resulting complexes were incubated with 20 µL of pre-equilibrated anti-FLAG M2 Affinity Gel (Sigma) in pulldown buffer for 1 h. Beads were washed six times, and the bound complex was eluted with 3x Flag peptide prepared in pulldown buffer. The input and pulldown samples were analysed by SDS-PAGE followed by Oriole stain.

### In vitro pulldown of ubiquitin

Twin strep-tagged FAF2 (WT and ΔUBA), K48-linked Ub trimer (Ub_3_), and hexamer (Ub_6_) chains were mixed in binding buffer (20 mM HEPES, pH 7.5, 150 mM NaCl and 0.5 mM TCEP) at a final concentration of 2.5 µM in a 1:1 molar ratio and were incubated with 10 µL of pre-equilibrated Pierce™ High-Capacity Streptavidin Agarose (Thermo Fisher) in pulldown buffer (20 mM HEPES, pH 7.5, 150 mM NaCl, 0.5 mM TCEP and 0.3% DDM) on a roller at 4 °C for 1 h. Beads were washed three times with ice-cold pulldown buffer. Bead-bound protein was eluted with 2× LDS sample buffer and analysed by SDS–PAGE followed by oriole stain.

### HDX-MS sample preparation

About 5 µM p9-UFD1-NPL4 with 1 mM ATPγS was incubated with or without 5 µM FAF2 or for FAF2 Apo Conditions 5 µM FAF2 alone, for 30 min in Protein Dilution Buffer (20 mM Tris, pH 7.5, 150 mM NaCl, 10 mM MgCl_2_ and 0.5 mM TCEP) on ice. 5 µl of this sample was diluted with 45 µL of Deuteration Buffer (20 mM Tris pH 7.5, 150 mM NaCl, 10 mM MgCl_2_, 0.5 mM TCEP and 94.6% D_2_O) (final D_2_O concentration = 85.1%) for time points 3, 30, 300 and 3000 s, before being quenched with 20 µl of ice-cold Quench Solution (6 M Urea, 2% Formic Acid), and being snap frozen in liquid nitrogen and stored at –70 °C. A further time point, 0.3 s, was achieved by incubating both the sample and deuteration buffer on ice and then conducting a 3 s exchange reaction. Each exchange reaction was conducted independently three times.

### HDX-MS data acquisition

HDX-MS data acquisition and analysis were carried out as described previously (Kanta et al, 2025). Briefly, samples were rapidly thawed at room temperature and then injected into automated HDX-MS fluidics and UPLC manager system (Waters). Samples were loaded into a 50 µl loop and then subsequently digested using a Waters Enzymate BEH Pepsin Column (Part No. 186007233) in a 0.1% Formic Acid solution with a flow rate of 200 µl/min at 20 °C. Peptic peptides then flowed onto a Waters ACQUITY UPLC BEH C18 VanGuard Pre-Column at 1 °C (Part No. 186003975). After digestion, the flow path was changed to elute the peptides via a Waters ACQUITY UPLC BEH C18 1.7 µm 1.0 × 100 mm reverse phase column (Part No. 186002346). A gradient from 0–85% 0.1% formic acid/acetonitrile, conducted at 1 °C with a 40 µl/min flow rate, was used to elute deuterated peptides, which were then ionised using an ESI source. Mass spectrometry data were collected using a Waters Select Series cIMS instrument, from a 50–2,000 m/z range with the instrument in HDMSe mode. A single pass of the cyclic ion mobility separator (with a cycle time of 47 ms) was conducted. A blank sample of protein dilution buffer with quench was run between samples, and carryover was routinely checked to be <1% intensity of the prior sample.

### HDX-MS data analysis

Peptide sequence identification was conducted using Protein Lynx Global Server (Waters), searching against a database of the protein complex, common contaminants, porcine pepsin, and the previous project’s sequences. Search criteria include S/T/Y phosphorylation. Minimum inclusion criteria were a minimum intensity of 5000 counts, minimum sequence length 5, maximum sequence length 35, a minimum of three fragment ions, a minimum of 0.1 products per amino acid, a minimum score of 5.0, a maximum MH + Error of 10 ppm. Subsequent analysis and determination of deuteration values were conducted using HDExaminer (Sierra Analytics/Trajan). Experimental design, data acquisition, analysis and reporting are in line with the community-agreed recommendations (Masson et al, 2019).

### Cross-linking mass spectrometry (XL-MS) sample preparation

Cross-linking reactions were optimised by varying disuccinimidyl sulfoxide (DSSO, Thermo Fisher Scientific) (Kao et al, 2011) concentration, protein concentration, and cross-linking time, and analysed by SDS-PAGE and mass photometry. 5 μM p97 hexamer was incubated for 30 min at 25 °C in 50 mM HEPES pH 7.5, 5 mM MgCl_2_, 1 mM ATPγS containing (as appropriate) 10 μM UFD1-NPL4, 10 μM FAF2 and 10 μM K48-linked Ub_6–8_ ubiquitin chain. Freshly prepared DSSO was added to a final concentration of 600 μM and the cross-linking reaction allowed to proceed for a further 15 min at 25 °C before quenching with 80 mM Tris-HCl, pH 8.8. Four independent replicates were performed for each condition.

Cross-linked samples were denatured and reduced in 1% (w/v) SDS, 10 mM DTT for 5 min at 95 °C. Ammonium bicarbonate buffer (pH 8) was added to a final concentration of 10 mM and proteins were alkylated with 50 mM iodoacetamide at room temperature for 30 min in the dark. Trichloroacetic acid (TCA) was added to a final concentration of 20% (v/v) to precipitate protein, and samples were incubated on ice for 15 min before centrifugation at 17,000 × *g* for 10 min at 4 °C. Proteins were resuspended in 10% (v/v) TCA and pelleted once more by centrifugation at 17,000 × *g* for 5 min. Pellets were washed 3 times with MS-grade water (17,000 × *g* for 5 min at 4 °C) and resuspended in 30 μL of a denaturing solution containing 8 M urea and 20 mM DTT. Following incubation for 10 min at 30 °C, each replicate was further split into 3 × 10 μL aliquots and 90 μL of 25 mM ammonium bicarbonate buffer was added to adjust the urea concentration to 0.8 M. All samples were digested for 20 h with 2 ng/μL MS-grade trypsin (Thermo Fisher Scientific) at 30 °C. Additionally, 4 h after the beginning of this incubation, double digests were created by adding to the auxiliary aliquots created for each initial replicate either Asp‑N endoprotease (Promega) to a final concentration of 0.2 ng/μL or Glu‑C endoprotease (Promega) to a final concentration of 1 ng/μL (thereby creating 3 digestion conditions in order to maximise sequence coverage).

### LC-MS/MS analysis of cross-linked peptides

Liquid chromatography tandem mass spectrometry (LC-MS/MS) experiments were performed on an UltiMate 3000 RSLC nano-HPLC system coupled to an Orbitrap Lumos mass spectrometer. About 5 µl solution from each digested cross-linked sample was loaded onto the nano-HPLC system individually. Peptides were trapped by a pre-column (Acclaim PepMapTM 100, C18, 100 µm × 2 cm, 5 µm, 100 A) using an aqueous solution containing 0.1% (v/v) TFA. Peptides were then separated by an analytical column (PepMapTM RSLC C18, 75 µm × 50 cm, 2 µm, 100 A) at 45 °C using a linear gradient of 3 to 35% solvent B (solution containing 80% ACN and 0.1% FA) over 90 min, 35 to 85% solvent B over 5 min, 85% solvent B for 10 min, 85 to 3% solvent B over 1 min and 3% solvent B for a father 8 min. The flow rate was set at 300 nl/min for all experiments. Data were acquired in data-dependent MS/MS mode. For each MS scan, the scan range was set between 375 and 1,500 m/z with the resolution at 120,000 and 300% automatic gain control (AGC) was used. The maximum injection time for each MS scan was 100 ms. Peptides with charge state between 2 and 8, as well as intensity threshold higher than 1.0e + 4 were then isolated with a 1.2 Da isolation window sequentially. Stepped HCD with normalised collision energy of 27, 30 and 33% was applied to fragment the isolated peptides. For each MS/MS scan, the resolution was set at 15,000 with a normalised AGC at 200%. The maximum injection time was set at 250 ms. Dynamic exclusion with a 60-s duration and 10 ppm window was enabled for the experiment.

### XL-MS data analysis

The .RAW files obtained from LC-MS/MS were converted into .mgf files using the MSConvert tool within the ProteoWizard software suite (Holman et al, 2014). The resulting .mgf files were analysed using MeroX to assign and discriminate the cross-linked products (Götze et al, 2015). Settings corresponding to peptide lengths ranging from 3 to 50 amino acids containing a maximum of three missed cleavages were applied, with the appropriate digestive enzyme(s) considered. Carbamidomethylation at cysteine residues was set as a static modification, and oxidation at methionine and deamidation at asparagine were included as variable modifications. DSSO was selected as the crosslinker, with its principle specificity set for lysine residues, but also considering reactivity for serine, threonine, tyrosine, and the N-terminus of target proteins (Iacobucci et al, 2019; Smith et al, 2018). 10 ppm and 20 ppm were used to filter the mass error in precursor ion (MS1) and fragment ion (MS2) scans. Only ions with a signal-to-noise ratio higher than 2 went forward for database searching. RISEUP searching mode was applied, with a minimum of two fragments per peptide and a false discovery rate (FDR) cut‑off of 5% set for cross-linked peptide identification. The common repository of adventitious proteins (cRAP) was included to increase the size of the decoy database and improve FDR estimation. Data from the three different protease digestion conditions (trypsin alone, trypsin + Asp-N, trypsin + Glu-C) were recombined into a single dataset and potential cross-linked peptides with a MeroX score higher than 50 were manually inspected to ensure unambiguous cross-link identification (Iacobucci et al, 2018). Filtered cross-linked data was exported from MeroX and uploaded to xiVIEW for visualisation and figure preparation (Combe et al, 2024).

### Mass photometry

Proteins were cross-linked with DSSO as described for cross-linking mass spectrometry. Samples were then diluted 50-fold in phosphate-buffered saline (PBS) to a final concentration of ~100 nM p97 hexamer. Mass photometry was performed using a Refeyn One^MP^ mass photometer (Refeyn Ltd, Oxford, UK) combined with AcquireMP and DiscoverMP software for data acquisition and analysis, respectively (Wu and Piszczek, 2021; Young et al, 2018). Microscope coverslips (VWR) and Grace Bio-Labs reusable Culturewell gaskets (Sigma) were cleaned by washing sequentially three times with isopropanol and deionised water and then dried with a stream of compressed air. A calibration curve was established consisting of sweet potato β-amylase monomer (56 kDa) and dimer (112 kDa), bovine thyroglobulin dimer (670 kDa) and the lactate dehydrogenase (146 kDa) and apoferritin band 2 (480 kDa) components of Thermo’s NativeMark unstained protein standard. To find focus, 5 µL of PBS was pipetted into a fresh well of the gasket/coverslip assembly and the focal position was identified. About 5 µL of cross-linked protein sample was added to the same well to achieve a final working concentration of ~50 nM p97 hexamer complex. A 60 s movie was recorded, and the distribution peaks were fitted with Gaussian functions to obtain the average molecular mass of each distribution component.

### Design of UFD1-binding FAF2 helix scaffolds with RFdiffusion

A motif scaffolding approach was taken to design potential p97-activating protein scaffolds upon the predicted UFD1-binding helix of FAF2. AF3 (Abramson et al, 2024) was used to design a model of the complex between p97 (N-terminal and D1 ATPase domains), NPL4, UFD1, FAF2 and 10 ubiquitin. This model was used as the input for binder generation using RFdiffusion (Watson et al, 2023), integrated with ProteinMPNN amino acid prediction (Dauparas et al, 2022) and AF2-based folding and validation (Jumper et al, 2021), and run online as a single pipeline via a Google Colab notebook. Residues T286, L289, R290, Q292, Q293, D294, A296, Y297, S300, L301, D304, Q305, K307, E308 and K311 were fixed and scaffolded and various N- and C-terminal extensions of between 5 and 160 amino acids were sampled, with RFdiffusion also inpainting the intervening residues within the FAF2 helical scaffold to implicitly reason over their sequence and better pack against them (Wang et al, 2022). This motif scaffolding of the FAF2 helix was performed in the presence of the UT3 domain of UFD1 (amino acids 17–196) and two ubiquitin molecules. While we did experiment with the selection of hotspot residues, none of the high-ranking nor successful designs were derived from design trajectories involving hotspots. RFdiffusion settings were left largely as default or Baker Lab recommended settings (50 iterations, 8 ProteinMPNN sequences per design, MPNN sampling temp of 0.1, cysteines excluded from consideration during MPNN step, AF2 + initial guess used to validate protein structures with three recycle steps).

In total, 2248 UFD1 binders, representing 281 distinct backbone trajectories, were generated over 16 Colab runtimes. Candidates were filtered based on pAE (<4) and plDDT (>0.85), with pTM scores above 0.75 and RMSD scores less than 2 Å also considered desirable. To promote diversity amongst the binder designs, only the top-ranked ProteinMPNN-generated sequence belonging to each design trajectory was considered. One exception to the filtered designs outlined above was Binder 4 (*Run12_Design8_mpnn5*), which failed on all metrics but stood out as the only design out of 256 generated during a rather unsuccessful runtime that was predicted by AF2 to bind to UFD1 in the same location as FAF2.

### Expression and purification of de novo binders

Codon-optimised genes encoding His-tagged versions of the designed binder sequences were synthesised by Twist Bioscience (see Reagents and tools table). Plasmids were transformed into chemically competent *E. coli* Rosetta (DE3) cells and grown in Luria-Bertani broth supplemented with 50 µg/mL kanamycin. Proteins were expressed in 250–1000 mL autoinduction LB medium (containing kanamycin) for 24 h at 28 °C. Cells were harvested by centrifugation at 4000 × *g* for 20 min at 4 °C, washed once with ice-cold PBS, and resuspended in 50 mM Tris-HCl, pH 7.5, 300 mM NaCl, 20 mM imidazole supplemented with 1 mg/mL lysozyme, 25 U/mL Pierce Universal Nuclease and “cOmplete” Protease Inhibitor cocktail (EDTA-free). Lysis was achieved by sonication, and the lysates were clarified by centrifugation at 40,000 × *g* for 40 min at 4 °C. The supernatants were incubated with Ni-NTA agarose and loaded onto gravity columns. The resin was washed with 3–5 column volumes of buffer (50 mM Tris-HCl, pH 7.5, 300 mM NaCl, 20 mM imidazole) and bound proteins eluted with 50 mM Tris-HCl, pH 7.5, 300 mM NaCl, supplemented with 300 mM imidazole. The eluted proteins were then dialysed overnight against 20 mM HEPES, pH 7.5, 150 mM NaCl, 10 mM MgCl_2_, 0.1 mM TCEP, or further purified by gel filtration chromatography using a Superdex 75 16/600 column equilibrated with 20 mM HEPES, pH 7.5, 150 mM NaCl, 10 mM MgCl_2_, 0.1 mM TCEP, as appropriate. Purity was assessed by SDS–PAGE, and protein solutions were aliquoted and stored at −80 °C for further use.

### AlphaFold prediction of p97-UN-FAF2 and polyUb complex

AlphaFold3 was used to model p97 (UniProt: P55072) -UFD1 (UniProt: Q92890)-NPL4 (UniProt: Q8TAT6)-FAF2 (UniProt: Q96CS3), p97-UN-FAF1 (UniProt: Q9UNN5), p97-UN-FAF2-10x Ub (UniProt: P0CG48) complex (Abramson et al, 2024) and UFD1 (UT3, 1-196)-2xUb-FAF2 (CR, 275–320) complex. From the five predictions generated for each complex, the model with the highest ipTM (interface predicted Template Modelling) score was selected for analysis.

### Bioinformatics analysis

Conservation analysis of UFD1 and FAF2 was performed by ConSurf (Ashkenazy et al, 2016). Multiple sequence alignments were performed using ClustalW (Sievers et al, 2011) and visualised with ESPript3 (Gouet et al, 1999). Structural homology search for FAF2 CR was performed using the DALI web server (Holm et al, 2023).

### Isothermal titration calorimetry (ITC)

ITC titration experiments were performed on a MicroCal PEAQ-ITC instrument (Malvern, version 1.29.32) operated with PEAQ-ITC software (Malvern) at 25 °C. Before measurement, all proteins were dialysed in ITC buffer (50 mM Tris (pH 7.5), 150 mM NaCl and 0.25 mM TCEP). UFD1 was added to the syringe and titrated into the cell which contained K48-Ub_2_. For titration of UFD1 and K48-Ub_2_, concentrations of 199 and 30.2 µM were used, respectively. Similarly, FAF2 ΔUBA was added to the syringe at a concentration of 141 uM and titrated into the cell, which contained K48-Ub_2_ at a concentration of 20.1 µM. Data were analysed, and titration curves were fitted using MicroCal PEAQ-ITC (Malvern) analysis software and GraphPad Prism 9 was used for figure generation.

### Microscale thermophoresis

FAF2 ΔUBA-Twin strep was diluted to 10 μM in 20 mM Hepes, pH 7.5, 150 mM NaCl, 0.5 mm TCEP and incubated with 3x molar concentration of second generation NHS-647 dye (Nanotemper) for 30 min before purifying unbound dye using a PD10 gravity desalting column equilibrated with the same buffer having 0.05% Tween20. Labelled FAF2 ΔUBA was diluted to 10 nM in the same buffer and mixed 1:1 with 16 serial dilutions of K48-Ub_3_, with the highest concentration of 262 µM, giving a final concentration of 5 nM FAF2 in each sample. Samples were loaded into premium capillaries and analysed using a Monolith Pico instrument. The instrument was set to 25 °C with the MST power set to medium and the laser power set to 20%. A total of 65 independent runs were averaged to produce the binding isotherm.

### Fluorescence polarisation binding assay

K48-Ub_5_ (Ub_4_-Ub^G76C^) chain was site-specifically labelled at the cysteine residue on proximal ubiquitin using BODIPY Maleimide Dye (Thermo Fisher), and free dye was removed by desalting and dialysis. Fluorescence polarisation assays were performed in black small-volume 384-well plates (Greiner). BODIPY-labelled Ub_5_ was diluted to 5x of the final concentration of 100 nM into assay buffer (20 mM Hepes, pH 7.5, 150 mM NaCl, 0.5 mM TCEP). The fluorophore was mixed with serial dilutions of FAF2 ΔUBA-Twin Strep at 1.25x concentration, with the highest at 100 µM. The plate was incubated at room temperature for 15 min. Fluorescence polarisation measurements were recorded with a 482 nM excitation filter and a 530 nM emission filter at a target gain of 100 using a Clariostar Plus plate reader (BMG Labtech). Measurements were performed in triplicate, and data were analysed using GraphPad Prism.

### Data analysis and figure preparation

Data were analysed and plotted using Prism v.10.6.1 (GraphPad). Figures were prepared using Adobe Illustrator and UCSF ChimeraX.

## Supplementary information

Appendix

Peer Review File

Source data Fig. 1

Source data Fig. 2

Source data Fig. 3

Source data Fig. 4

Source data Fig. 5

Source data Fig. 6

Figure EV1 Source Data

Figure EV2 Source Data

Figure EV3 Source Data

Figure EV4 Source Data

Figure EV5 Source Data

Expanded View Figures

## Data Availability

HDX-MS and cross-linking-MS data have been deposited to the ProteomeXchange Consortium via the PRIDE partner repository (https://www.ebi.ac.uk/pride/) with the dataset identifiers PXD073479 and PXD078865, respectively. The source data of this paper are collected in the following database record: biostudies:S-SCDT-10_1038-S44318-026-00894-x.

## References

[CR1] Abramson J, Adler J, Dunger J, Evans R, Green T, Pritzel A, Ronneberger O, Willmore L, Ballard AJ, Bambrick J et al (2024) Accurate structure prediction of biomolecular interactions with AlphaFold 3. Nature 630:493–500 38718835 10.1038/s41586-024-07487-w PMC11168924

[CR2] Ahlstedt BA, Ganji R, Raman M (2022) The functional importance of VCP to maintaining cellular protein homeostasis. Biochem Soc Trans 50:1457–1469 36196920 10.1042/BST20220648 PMC9704522

[CR3] Ashkenazy H, Abadi S, Martz E, Chay O, Mayrose I, Pupko T, Ben-Tal N (2016) ConSurf 2016: an improved methodology to estimate and visualize evolutionary conservation in macromolecules. Nucleic Acids Res 44:W344–W350 27166375 10.1093/nar/gkw408 PMC4987940

[CR4] Bard JAM, Bashore C, Dong KC, Martin A (2019) The 26S proteasome utilizes a kinetic gateway to prioritize substrate degradation. Cell 177:286–298.e15 30929903 10.1016/j.cell.2019.02.031 PMC6519451

[CR5] Beskow A, Grimberg KB, Bott LC, Salomons FA, Dantuma NP, Young P (2009) A conserved unfoldase activity for the p97 AAA-ATPase in proteasomal degradation. J Mol Biol 394:732–746 19782090 10.1016/j.jmb.2009.09.050

[CR6] Blythe EE, Gates SN, Deshaies RJ, Martin A (2019) Multisystem proteinopathy mutations in VCP/p97 increase NPLOC4·UFD1L binding and substrate processing. Structure 27:1820–1829.e4 31623962 10.1016/j.str.2019.09.011 PMC6929323

[CR7] Blythe EE, Olson KC, Chau V, Deshaies RJ (2017) Ubiquitin- and ATP-dependent unfoldase activity of P97/VCP•NPLOC4•UFD1L is enhanced by a mutation that causes multisystem proteinopathy. Proc Natl Acad Sci USA 114:E4380–E4388 28512218 10.1073/pnas.1706205114 PMC5465906

[CR8] Bodnar NO, Rapoport TA (2017) Molecular mechanism of substrate processing by the Cdc48 ATPase complex. Cell 169:722–735.e9 28475898 10.1016/j.cell.2017.04.020 PMC5751438

[CR9] Brandman O, Stewart-Ornstein J, Wong D, Larson A, Williams CC, Li G-W, Zhou S, King D, Shen PS, Weibezahn J et al (2012) A ribosome-bound quality control complex triggers degradation of nascent peptides and signals translation stress. Cell 151:1042–1054 23178123 10.1016/j.cell.2012.10.044 PMC3534965

[CR10] Buchberger A, Schindelin H, Hänzelmann P (2015) Control of p97 function by cofactor binding. FEBS Lett 589:2578–2589 26320413 10.1016/j.febslet.2015.08.028

[CR11] Bulfer SL, Chou T-F, Arkin MR (2016) p97 disease mutations modulate nucleotide-induced conformation to alter protein–protein interactions. ACS Chem Biol 11:2112–2116 27267671 10.1021/acschembio.6b00350 PMC5224236

[CR12] Castañeda CA, Dixon EK, Walker O, Chaturvedi A, Nakasone MA, Curtis JE, Reed MR, Krueger S, Cropp TA, Fushman D (2016) Linkage via K27 bestows ubiquitin chains with unique properties among polyubiquitins. Structure 24:423–436 26876099 10.1016/j.str.2016.01.007 PMC4787624

[CR13] Chou T-F, Brown SJ, Minond D, Nordin BE, Li K, Jones AC, Chase P, Porubsky PR, Stoltz BM, Schoenen FJ et al (2011) Reversible inhibitor of p97, DBeQ, impairs both ubiquitin-dependent and autophagic protein clearance pathways. Proc Natl Acad Sci USA 108:4834–4839 21383145 10.1073/pnas.1015312108 PMC3064330

[CR14] Combe CW, Graham M, Kolbowski L, Fischer L, Rappsilber J (2024) xiVIEW: visualisation of crosslinking mass spectrometry data. J Mol Biol 436:168656 39237202 10.1016/j.jmb.2024.168656

[CR15] Cooney I, Schubert HL, Cedeno K, Fisher ON, Carson R, Price JC, Hill CP, Shen PS (2024) Visualization of the Cdc48 AAA+ ATPase protein unfolding pathway. Nat Commun 15:7505 39209885 10.1038/s41467-024-51835-3 PMC11362554

[CR16] Dantuma NP, Lindsten K, Glas R, Jellne M, Masucci MG (2000) Short-lived green fluorescent proteins for quantifying ubiquitin/proteasome-dependent proteolysis in living cells. Nat Biotechnol 18:538–543 10802622 10.1038/75406

[CR17] Darwich NF, Phan JM, Kim B, Suh E, Papatriantafyllou JD, Changolkar L, Nguyen AT, O’Rourke CM, He Z, Porta S et al (2020) Autosomal dominant VCP hypomorph mutation impairs disaggregation of PHF-tau. Science 370:eaay8826 33004675 10.1126/science.aay8826 PMC7818661

[CR18] Dauparas J, Anishchenko I, Bennett N, Bai H, Ragotte RJ, Milles LF, Wicky BIM, Courbet A, de Haas RJ, Bethel N et al (2022) Robust deep learning–based protein sequence design using ProteinMPNN. Science 378:49–56 36108050 10.1126/science.add2187 PMC9997061

[CR19] Ewens CA, Panico S, Kloppsteck P, McKeown C, Ebong I-O, Robinson C, Zhang X, Freemont PS (2014) The p97-FAF1 protein complex reveals a common mode of p97 adaptor binding. J Biol Chem 289:12077–12084 24619421 10.1074/jbc.M114.559591 PMC4002113

[CR20] Figuerola-Conchas A, Saarbach J, Daguer J-P, Cieren A, Barluenga S, Winssinger N, Gotta M (2020) Small-molecule modulators of the ATPase VCP/p97 affect specific p97 cellular functions. ACS Chem Biol 15:243–253 31790201 10.1021/acschembio.9b00832

[CR21] Fujisawa R, Polo Rivera C, Labib KP (2022) Multiple UBX proteins reduce the ubiquitin threshold of the mammalian p97-UFD1-NPL4 unfoldase. eLife 11:e76763 35920641 10.7554/eLife.76763 PMC9377798

[CR22] Ganji R, Paulo JA, Xi Y, Kline I, Zhu J, Clemen CS, Weihl CC, Purdy JG, Gygi SP, Raman M (2023) The p97-UBXD8 complex regulates ER-Mitochondria contact sites by altering membrane lipid saturation and composition. Nat Commun 14:638 36746962 10.1038/s41467-023-36298-2 PMC9902492

[CR23] Götze M, Pettelkau J, Fritzsche R, Ihling CH, Schäfer M, Sinz A (2015) Automated assignment of MS/MS cleavable cross-links in protein 3D-structure analysis. J Am Soc Mass Spectrom 26:83–97 25261217 10.1007/s13361-014-1001-1

[CR24] Gouet P, Courcelle E, Stuart DI, Métoz F (1999) ESPript: analysis of multiple sequence alignments in PostScript. Bioinformatics 15:305–308 10320398 10.1093/bioinformatics/15.4.305

[CR25] Gwon Y, Maxwell BA, Kolaitis R-M, Zhang P, Kim HJ, Taylor JP (2021) Ubiquitination of G3BP1 mediates stress granule disassembly in a context-specific manner. Science 372:eabf6548 34739333 10.1126/science.abf6548 PMC8574224

[CR26] Hänzelmann P, Buchberger A, Schindelin H (2011) Hierarchical binding of cofactors to the AAA ATPase p97. Structure 19:833–843 21645854 10.1016/j.str.2011.03.018

[CR27] Hänzelmann P, Schindelin H (2017) The interplay of cofactor interactions and post-translational modifications in the regulation of the AAA+ ATPase p97. Front Mol Biosci 4:21 10.3389/fmolb.2017.00021 PMC5389986 28451587

[CR28] Heidelberger JB, Voigt A, Borisova ME, Petrosino G, Ruf S, Wagner SA, Beli P (2018) Proteomic profiling of VCP substrates links VCP to K6-linked ubiquitylation and c-Myc function. EMBO Rep 19:e44754 29467282 10.15252/embr.201744754 PMC5891417

[CR29] Holm L, Laiho A, Törönen P, Salgado M (2023) DALI shines a light on remote homologs: One hundred discoveries. Protein Sci 32:e4519 36419248 10.1002/pro.4519 PMC9793968

[CR30] Holman JD, Tabb DL, Mallick P (2014) Employing ProteoWizard to convert raw mass spectrometry data. Curr Protoc Bioinform 46:13.24.1–13.24.9 10.1002/0471250953.bi1324s46 PMC4113728 24939128

[CR31] Huo X-Y, Liu D, Zou R, Li Z-P, Li Y, Pan L, Zhang Y, Zhang Z-R (2025) A UBH-UBX module amplifies p97/VCP’s unfolding power to facilitate protein extraction and degradation. Nat Commun 16:10162 41258083 10.1038/s41467-025-65166-4 PMC12630741

[CR32] Iacobucci C, Götze M, Ihling CH, Piotrowski C, Arlt C, Schäfer M, Hage C, Schmidt R, Sinz A (2018) A cross-linking/mass spectrometry workflow based on MS-cleavable cross-linkers and the MeroX software for studying protein structures and protein–protein interactions. Nat Protoc 13:2864–2889 30382245 10.1038/s41596-018-0068-8

[CR33] Iacobucci C, Piotrowski C, Aebersold R, Amaral BC, Andrews P, Bernfur K, Borchers C, Brodie NI, Bruce JE, Cao Y et al (2019) First community-wide, comparative cross-linking mass spectrometry study. Anal Chem 91:6953–6961 31045356 10.1021/acs.analchem.9b00658 PMC6625963

[CR34] Ji Z, Li H, Peterle D, Paulo JA, Ficarro SB, Wales TE, Marto JA, Gygi SP, Engen JR, Rapoport TA (2022) Translocation of polyubiquitinated protein substrates by the hexameric Cdc48 ATPase. Mol Cell 82:570–584.e8 34951965 10.1016/j.molcel.2021.11.033 PMC8818041

[CR35] Johnson ES, Ma PCM, Ota IM, Varshavsky A (1995) A proteolytic pathway that recognizes ubiquitin as a degradation signal. J Biol Chem 270:17442–17456 7615550 10.1074/jbc.270.29.17442

[CR36] Johnson JO, Mandrioli J, Benatar M, Abramzon Y, Deerlin VMV, Trojanowski JQ, Gibbs JR, Brunetti M, Gronka S, Wuu J et al (2010) Exome sequencing reveals VCP mutations as a cause of familial ALS. Neuron 68:857–864 21145000 10.1016/j.neuron.2010.11.036 PMC3032425

[CR37] Jones NH, Liu Q, Urnavicius L, Dahan NE, Vostal LE, Kapoor TM (2024) Allosteric activation of VCP, an AAA unfoldase, by small molecule mimicry. Proc Natl Acad Sci USA 121:e2316892121 38833472 10.1073/pnas.2316892121 PMC11181084

[CR38] Ju J-S, Miller SE, Hanson PI, Weihl CC (2008) Impaired protein aggregate handling and clearance underlie the pathogenesis of p97/VCP-associated disease. J Biol Chem 283:30289–30299 18715868 10.1074/jbc.M805517200 PMC2573070

[CR39] Jumper J, Evans R, Pritzel A, Green T, Figurnov M, Ronneberger O, Tunyasuvunakool K, Bates R, Žídek A, Potapenko A et al (2021) Highly accurate protein structure prediction with AlphaFold. Nature 596:583–589 34265844 10.1038/s41586-021-03819-2 PMC8371605

[CR40] Kanta S, Vinciauskaite V, Neill G, Muqit MMK, Masson GR (2025) Structural insights into allosteric inhibition of HRI kinase by heme binding via HDX-MS. Biochem J 482:859–875 40471279 10.1042/BCJ20253072 PMC12235045

[CR41] Kao A, Chiu C, Vellucci D, Yang Y, Patel VR, Guan S, Randall A, Baldi P, Rychnovsky SD, Huang L (2011) Development of a Novel Cross-linking Strategy for Fast and Accurate Identification of Cross-linked Peptides of Protein Complexes. Mol Cell Proteom 10:M110.002170 10.1074/mcp.M110.002212 PMC3013449 20736410

[CR42] Kim C, Gabriel KR, Boone D, Brown MR, Oppenheimer K, Kost-Alimova M, Pablo JLB, Greka A (2025) FAF2 is a bifunctional regulator of peroxisomal homeostasis and saturated lipid responses. Sci Adv 11:eadu9104 10.1126/sciadv.adu9104 PMC12219479 40601736

[CR43] Kochenova OV, Mukkavalli S, Raman M, Walter JC (2022) Cooperative assembly of p97 complexes involved in replication termination. Nat Commun 13:6591 36329031 10.1038/s41467-022-34210-y PMC9633789

[CR44] Körner M, Müller P, Das H, Kraus F, Pfeuffer T, Spielhaupter S, Oeljeklaus S, Schülein-Völk C, Harper JW, Warscheid B et al (2025) p97/VCP is required for piecemeal autophagy of aggresomes. Nat Commun 16:4243 40335532 10.1038/s41467-025-59556-x PMC12059050

[CR45] Koyano F, Yamano K, Hoshina T, Kosako H, Fujiki Y, Tanaka K, Matsuda N (2024) AAA+ ATPase chaperone p97/VCPFAF2 governs basal pexophagy. Nat Commun 15:9347 39472561 10.1038/s41467-024-53558-x PMC11522385

[CR46] Kracht M, Kröning A, van den Boom J, Gahlot P, Koska S, Kiss L, Meyer H (2026) The accessory adapters FAF1, FAF2, and UBXN7 accelerate proteasomal degradation by increasing prior p97-mediated substrate unfolding. Sci Adv 12:eaea7381 41790892 10.1126/sciadv.aea7381 PMC12965308

[CR47] Kracht M, van den Boom J, Seiler J, Kröning A, Kaschani F, Kaiser M, Meyer H (2020) Protein phosphatase-1 complex disassembly by p97 is initiated through multivalent recognition of catalytic and regulatory subunits by the p97 SEP-domain adapters. J Mol Biol 432:6061–6074 33058883 10.1016/j.jmb.2020.10.001

[CR48] Lange SM, McFarland MR, Lamoliatte F, Carroll T, Krshnan L, Pérez-Ràfols A, Kwasna D, Shen L, Wallace I, Cole I et al (2024) VCP/p97-associated proteins are binders and debranching enzymes of K48–K63-branched ubiquitin chains. Nat Struct Mol Biol 31:1872–1887 10.1038/s41594-024-01354-y PMC11638074 38977901

[CR49] Lee HG, Lemmon AA, Lima CD (2023) SUMO enhances unfolding of SUMO–polyubiquitin-modified substrates by the Ufd1/Npl4/Cdc48 complex. Proc Natl Acad Sci USA 120:e2213703120 36574706 10.1073/pnas.2213703120 PMC9910466

[CR50] Liao Z, Arkinson C, Martin A (2026) Faf1 accelerates p97-mediated protein unfolding by promoting ubiquitin engagement. Cell Rep 45:117393 42228561 10.1016/j.celrep.2026.117393 PMC13356908

[CR51] Maric M, Maculins T, De Piccoli G, Labib K (2014) Cdc48 and a ubiquitin ligase drive disassembly of the CMG helicase at the end of DNA replication. Science 346:1253596 25342810 10.1126/science.1253596 PMC4300516

[CR52] Masson GR, Burke JE, Ahn NG, Anand GS, Borchers C, Brier S, Bou-Assaf GM, Engen JR, Englander SW, Faber J et al (2019) Recommendations for performing, interpreting and reporting hydrogen deuterium exchange mass spectrometry (HDX-MS) experiments. Nat Methods 16:595–602 31249422 10.1038/s41592-019-0459-y PMC6614034

[CR53] Meyer H, Weihl CC (2014) The VCP/p97 system at a glance: connecting cellular function to disease pathogenesis. J Cell Sci 127:3877–3883 25146396 10.1242/jcs.093831 PMC4163641

[CR54] Meyer HH, Shorter JG, Seemann J, Pappin D, Warren G (2000) A complex of mammalian Ufd1 and Npl4 links the AAA-ATPase, p97, to ubiquitin and nuclear transport pathways. EMBO J 19:2181–2192 10811609 10.1093/emboj/19.10.2181 PMC384367

[CR55] Meyer HH, Wang Y, Warren G (2002) Direct binding of ubiquitin conjugates by the mammalian p97 adaptor complexes, p47 and Ufd1–Npl4. EMBO J 21:5645–5652 12411482 10.1093/emboj/cdf579 PMC131076

[CR56] Meyer H-J, Rape M (2014) Enhanced protein degradation by branched ubiquitin chains. Cell 157:910–921 24813613 10.1016/j.cell.2014.03.037 PMC4028144

[CR57] Morimoto D, Walinda E, Fukada H, Sou Y-S, Kageyama S, Hoshino M, Fujii T, Tsuchiya H, Saeki Y, Arita K et al (2015) The unexpected role of polyubiquitin chains in the formation of fibrillar aggregates. Nat Commun 6:6116 25600778 10.1038/ncomms7116 PMC4309437

[CR58] Olszewski MM, Williams C, Dong KC, Martin A (2019) The Cdc48 unfoldase prepares well-folded protein substrates for degradation by the 26S proteasome. Commun Biol 2:29 30675527 10.1038/s42003-019-0283-z PMC6340886

[CR59] Olzmann JA, Richter CM, Kopito RR (2013) Spatial regulation of UBXD8 and p97/VCP controls ATGL-mediated lipid droplet turnover. Proc Natl Acad Sci USA 110:1345–1350 23297223 10.1073/pnas.1213738110 PMC3557085

[CR60] Pan M, Yu Y, Ai H, Zheng Q, Xie Y, Liu L, Zhao M (2021a) Mechanistic insight into substrate processing and allosteric inhibition of human p97. Nat Struct Mol Biol 28:614–625 34262183 10.1038/s41594-021-00617-2

[CR61] Pan M, Zheng Q, Yu Y, Ai H, Xie Y, Zeng X, Wang C, Liu L, Zhao M (2021b) Seesaw conformations of Npl4 in the human p97 complex and the inhibitory mechanism of a disulfiram derivative. Nat Commun 12:121 33402676 10.1038/s41467-020-20359-x PMC7785736

[CR62] Papadopoulos C, Kirchner P, Bug M, Grum D, Koerver L, Schulze N, Poehler R, Dressler A, Fengler S, Arhzaouy K et al (2017) VCP/p97 cooperates with YOD1, UBXD1 and PLAA to drive clearance of ruptured lysosomes by autophagy. EMBO J 36:135–150 27753622 10.15252/embj.201695148 PMC5242375

[CR63] Park S, Isaacson R, Kim HT, Silver PA, Wagner G (2005) Ufd1 Exhibits the AAA-ATPase fold with two distinct ubiquitin interaction sites. Structure 13:995–1005 16004872 10.1016/j.str.2005.04.013

[CR64] Phan JM, Creekmore BC, Nguyen AT, Bershadskaya DD, Darwich NF, Mann CN, Lee EB (2024) VCP activator reverses nuclear proteostasis defects and enhances TDP-43 aggregate clearance in multisystem proteinopathy models. J Clin Invest 134:e169039 10.1172/JCI169039 PMC11257039 38787785

[CR65] Raman M, Sergeev M, Garnaas M, Lydeard JR, Huttlin EL, Goessling W, Shah JV, Harper JW (2015) Systematic proteomics of the VCP–UBXD adaptor network identifies a role for UBXN10 in regulating ciliogenesis. Nat Cell Biol 17:1356–1369 26389662 10.1038/ncb3238 PMC4610257

[CR66] Richly H, Rape M, Braun S, Rumpf S, Hoege C, Jentsch S (2005) A series of ubiquitin binding factors connects CDC48/p97 to substrate multiubiquitylation and proteasomal targeting. Cell 120:73–84 15652483 10.1016/j.cell.2004.11.013

[CR67] Saracino D, Clot F, Camuzat A, Anquetil V, Hannequin D, Guyant-Maréchal L, Didic M, Guillot-Noël L, Rinaldi D, Latouche M et al (2018) Novel *VCP* mutations expand the mutational spectrum of frontotemporal dementia. Neurobiol Aging 72:187.e11–187.e14 30005904 10.1016/j.neurobiolaging.2018.06.037

[CR68] Sato Y, Tsuchiya H, Yamagata A, Okatsu K, Tanaka K, Saeki Y, Fukai S (2019) Structural insights into ubiquitin recognition and Ufd1 interaction of Npl4. Nat Commun 10:5708 31836717 10.1038/s41467-019-13697-y PMC6910952

[CR69] Sievers F, Wilm A, Dineen D, Gibson TJ, Karplus K, Li W, Lopez R, McWilliam H, Remmert M, Söding J et al (2011) Fast, scalable generation of high-quality protein multiple sequence alignments using Clustal Omega. Mol Syst Biol 7:539 21988835 10.1038/msb.2011.75 PMC3261699

[CR70] Smith D-L, Götze M, Bartolec TK, Hart-Smith G, Wilkins MR (2018) Characterization of the interaction between arginine methyltransferase hmt1 and its substrate Npl3: use of multiple cross-linkers, mass spectrometric approaches, and software platforms. Anal Chem 90:9101–9108 30004689 10.1021/acs.analchem.8b01525

[CR71] Stein A, Ruggiano A, Carvalho P, Rapoport TA (2014) Key steps in ERAD of luminal ER proteins reconstituted with purified components. Cell 158:1375–1388 25215493 10.1016/j.cell.2014.07.050 PMC4163015

[CR72] Suryo Rahmanto A, Blum CJ, Scalera C, Heidelberger JB, Mesitov M, Horn-Ghetko D, Gräf JF, Mikicic I, Hobrecht R, Orekhova A et al (2023) K6-linked ubiquitylation marks formaldehyde-induced RNA-protein crosslinks for resolution. Mol Cell 83:4272–4289.e10 37951215 10.1016/j.molcel.2023.10.011

[CR73] Tsuchiya H, Burana D, Ohtake F, Arai N, Kaiho A, Komada M, Tanaka K, Saeki Y (2018) Ub-ProT reveals global length and composition of protein ubiquitylation in cells. Nat Commun 9:524 29410401 10.1038/s41467-018-02869-x PMC5802829

[CR74] Turakhiya A, Meyer SR, Marincola G, Böhm S, Vanselow JT, Schlosser A, Hofmann K, Buchberger A (2018) ZFAND1 recruits p97 and the 26S proteasome to promote the clearance of arsenite-induced stress granules. Mol Cell 70:906–919.e7 29804830 10.1016/j.molcel.2018.04.021

[CR75] Twomey EC, Ji Z, Wales TE, Bodnar NO, Ficarro SB, Marto JA, Engen JR, Rapoport TA (2019) Substrate processing by the Cdc48 ATPase complex is initiated by ubiquitin unfolding. Science 365:eaax1033 31249135 10.1126/science.aax1033 PMC6980381

[CR76] van den Boom J, Meyer H (2018) VCP/p97-mediated unfolding as a principle in protein homeostasis and signaling. Mol Cell 69:182–194 29153394 10.1016/j.molcel.2017.10.028

[CR77] Wang J, Lisanza S, Juergens D, Tischer D, Watson JL, Castro KM, Ragotte R, Saragovi A, Milles LF, Baek M et al (2022) Scaffolding protein functional sites using deep learning. Science 377:387–394 35862514 10.1126/science.abn2100 PMC9621694

[CR78] Wang Y, Zhang Z, He W, Wang P, Ji Z (2026) ATP-independent unfolding of ubiquitin by Ufd1 initiates Cdc48/p97-mediated substrate processing. Preprint at bioRxiv 10.64898/2026.03.07.710261 42722750

[CR79] Watson JL, Juergens D, Bennett NR, Trippe BL, Yim J, Eisenach HE, Ahern W, Borst AJ, Ragotte RJ, Milles LF et al (2023) De novo design of protein structure and function with RFdiffusion. Nature 620:1089–1100 37433327 10.1038/s41586-023-06415-8 PMC10468394

[CR80] Watts GDJ, Wymer J, Kovach MJ, Mehta SG, Mumm S, Darvish D, Pestronk A, Whyte MP, Kimonis VE (2004) Inclusion body myopathy associated with Paget disease of bone and frontotemporal dementia is caused by mutant valosin-containing protein. Nat Genet 36:377–381 15034582 10.1038/ng1332

[CR81] Weihl CC, Dalal S, Pestronk A, Hanson PI (2006) Inclusion body myopathy-associated mutations in p97/VCP impair endoplasmic reticulum-associated degradation. Hum Mol Genet 15:189–199 16321991 10.1093/hmg/ddi426

[CR82] Weith M, Seiler J, van den Boom J, Kracht M, Hülsmann J, Primorac I, del Pino Garcia J, Kaschani F, Kaiser M, Musacchio A et al (2018) Ubiquitin-independent disassembly by a p97 AAA-ATPase complex drives PP1 holoenzyme formation. Mol Cell 72:766–777.e6 30344098 10.1016/j.molcel.2018.09.020

[CR83] Williams C, Dong KC, Arkinson C, Martin A (2023) The Ufd1 cofactor determines the linkage specificity of polyubiquitin chain engagement by the AAA+ ATPase Cdc48. Mol Cell 83:759–769.e7 36736315 10.1016/j.molcel.2023.01.016 PMC9992269

[CR84] Wójcik C, Rowicka M, Kudlicki A, Nowis D, McConnell E, Kujawa M, DeMartino GN (2006) Valosin-containing protein (p97) is a regulator of endoplasmic reticulum stress and of the degradation of N-end rule and ubiquitin-fusion degradation pathway substrates in mammalian cells. Mol Biol Cell 17:4606–4618 16914519 10.1091/mbc.E06-05-0432 PMC1635394

[CR85] Wrobel L, Hill SM, Djajadikerta A, Fernandez-Estevez M, Karabiyik C, Ashkenazi A, Barratt VJ, Stamatakou E, Gunnarsson A, Rasmusson T et al (2022) Compounds activating VCP D1 ATPase enhance both autophagic and proteasomal neurotoxic protein clearance. Nat Commun 13:4146 35842429 10.1038/s41467-022-31905-0 PMC9288506

[CR86] Wu D, Piszczek G (2021) Standard protocol for mass photometry experiments. Eur Biophys J 50:403–409 33651123 10.1007/s00249-021-01513-9 PMC8344692

[CR87] Ye Y, Tang WK, Zhang T, Xia D (2017) A mighty “protein extractor” of the cell: structure and function of the p97/CDC48 ATPase. Front Mol Biosci 4:39 10.3389/fmolb.2017.00039 PMC5468458 28660197

[CR88] Young G, Hundt N, Cole D, Fineberg A, Andrecka J, Tyler A, Olerinyova A, Ansari A, Marklund EG, Collier MP et al (2018) Quantitative mass imaging of single molecules. Science 360:423–427 29700264 10.1126/science.aar5839 PMC6103225

[CR89] Zhang X, Gui L, Zhang X, Bulfer SL, Sanghez V, Wong DE, Lee Y, Lehmann L, Lee JS, Shih P-Y et al (2015) Altered cofactor regulation with disease-associated p97/VCP mutations. Proc Natl Acad Sci USA 112:E1705–E1714 25775548 10.1073/pnas.1418820112 PMC4394316

[CR90] Zhao G, Zhou X, Wang L, Li G, Schindelin H, Lennarz WJ (2007) Studies on peptide:N-glycanase–p97 interaction suggest that p97 phosphorylation modulates endoplasmic reticulum-associated degradation. Proc Natl Acad Sci USA 104:8785–8790 17496150 10.1073/pnas.0702966104 PMC1885580

[CR91] Zheng J, Cao Y, Yang J, Jiang H (2022) UBXD8 mediates mitochondria-associated degradation to restrain apoptosis and mitophagy. EMBO Rep 23:e54859 35979733 10.15252/embr.202254859 PMC9535754

