# Supplementary material for: FAF1 and FAF2 enhance unfolding by p97-UFD1-NPL4 complex enabling rational design of p97 activators
Source: EMBO J. 2026 Aug 15;45(18):6595–629. doi: 10.1038/s44318-026-00894-x (PMC13578712; doi:10.1038/s44318-026-00894-x)
Supplement: Supplementary file 1 [file 44318_2026_894_MOESM14_ESM.pdf]

# Expanded View Figures

**Figure EV1. Differential modulation of human p97 activity by UBA-UBX cofactors requires UFD1-NPL4.**

(**A**) Coomassie-stained SDS–PAGE showing purified human p97/yeast Cdc48, yeast Ufd1-Npl4 complex, human UFD1-NPL4 complex and UBA-UBX cofactor proteins used in this study. (**B**) Schematic representing the overview of the method employed to prepare ubiquitylated reporter Eos substrates. (**C**) Coomassie-stained SDS–PAGE of K48-linked ubiquitylated Eos substrates of various lengths used in the unfolding assays. (**D**) Unfolding of $Ub^{VS}$, $Ub^{S}$, and $Ub^{L}$ substrates by yeast Cdc48-Ufd1-Npl4 (left) and human p97-UFD1-NPL4 (right). (**E**) Comparison of unfolding of $Ub_6$-Eos by human p97-UFD1-NPL4 and yeast Cdc48-Ufd1-Npl4 unfoldase complex. (**F**) $Ub^{L}$ substrate unfolding by human p97 with UBA-UBX cofactors (p47, UBXN7, FAF1, FAF2 or UBXN1), alone or combined with UFD1-NPL4, showing cofactor-specific modulation of activity. (**G**) Substrate unfolding kinetics of $Ub_6$-Eos substrate by human p97 in the presence of UBA-UBX cofactors, either alone or in combination with UFD1-NPL4, demonstrating cofactor-specific effects on unfolding depend on ubiquitin chain length on the client. For panels (**D–G**), fluorescence intensity values were normalised to values at time 0 and substrate-only controls. Data shown as mean ± SD ($n \geq 3$ technical replicates for (**D**, **E**), $n = 3$ independent experiments, each with three technical replicates for (**F**) and $n = 2$ independent experiments, each with three technical replicates for (**G**). Source data are available online for this figure.


© The Author(s)

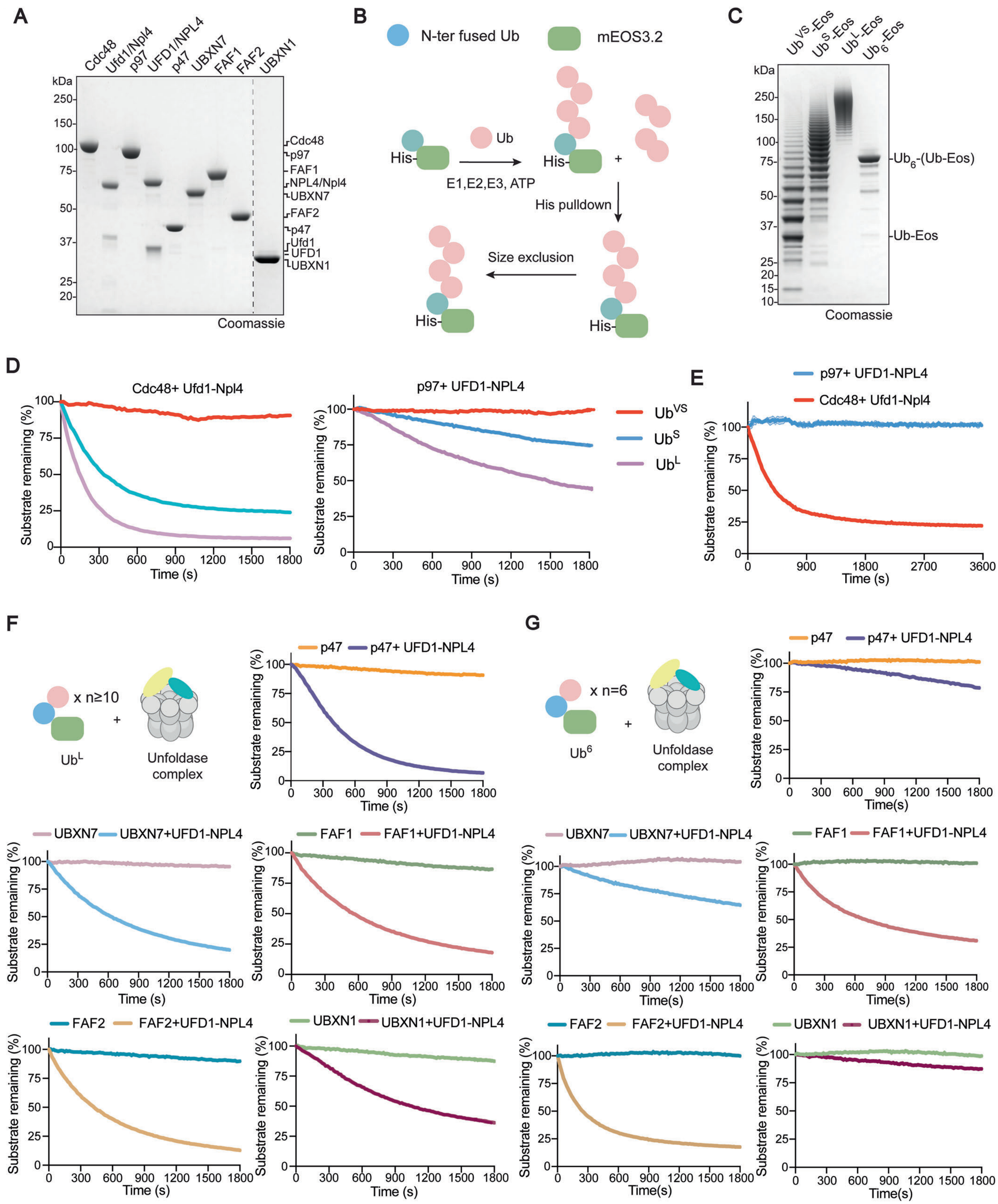
A
Cdc48
Ufd1/Npl4
p97
UFD1/NPL4
p47
UBXN7
FAF1
FAF2
UBXN1
kDa
250
150
100
75
50
37
25
20
Cdc48
p97
FAF1
NPL4/Npl4
UBXN7
FAF2
p47
Ufd1
UFD1
UBXN1
Coomassie
B
N-ter fused Ub
mEOS3.2
Ub
His-
E1,E2,E3, ATP
His pulldown
Size exclusion
C
Ub^VS-Eos
Ub^S-Eos
Ub^L-Eos
Ub6-Eos
kDa
Ub6-(Ub-Eos)
Ub-Eos
Coomassie
D
Cdc48+ Ufd1-Npl4
p97+ UFD1-NPL4
Substrate remaining (%)
Time (s)
Ub^VS
Ub^S
Ub^L
E
p97+ UFD1-NPL4
Cdc48+ Ufd1-Npl4
Substrate remaining (%)
Time (s)
F
x n≥10
Ub^L
Unfoldase complex
p47
p47+ UFD1-NPL4
UBXN7
UBXN7+UFD1-NPL4
FAF1
FAF1+UFD1-NPL4
FAF2
FAF2+UFD1-NPL4
UBXN1
UBXN1+UFD1-NPL4
G
x n=6
Ub^6
Unfoldase complex
p47
p47+ UFD1-NPL4
UBXN7
UBXN7+UFD1-NPL4
FAF1
FAF1+UFD1-NPL4
FAF2
FAF2+UFD1-NPL4
UBXN1
UBXN1+UFD1-NPL4
Time(s)

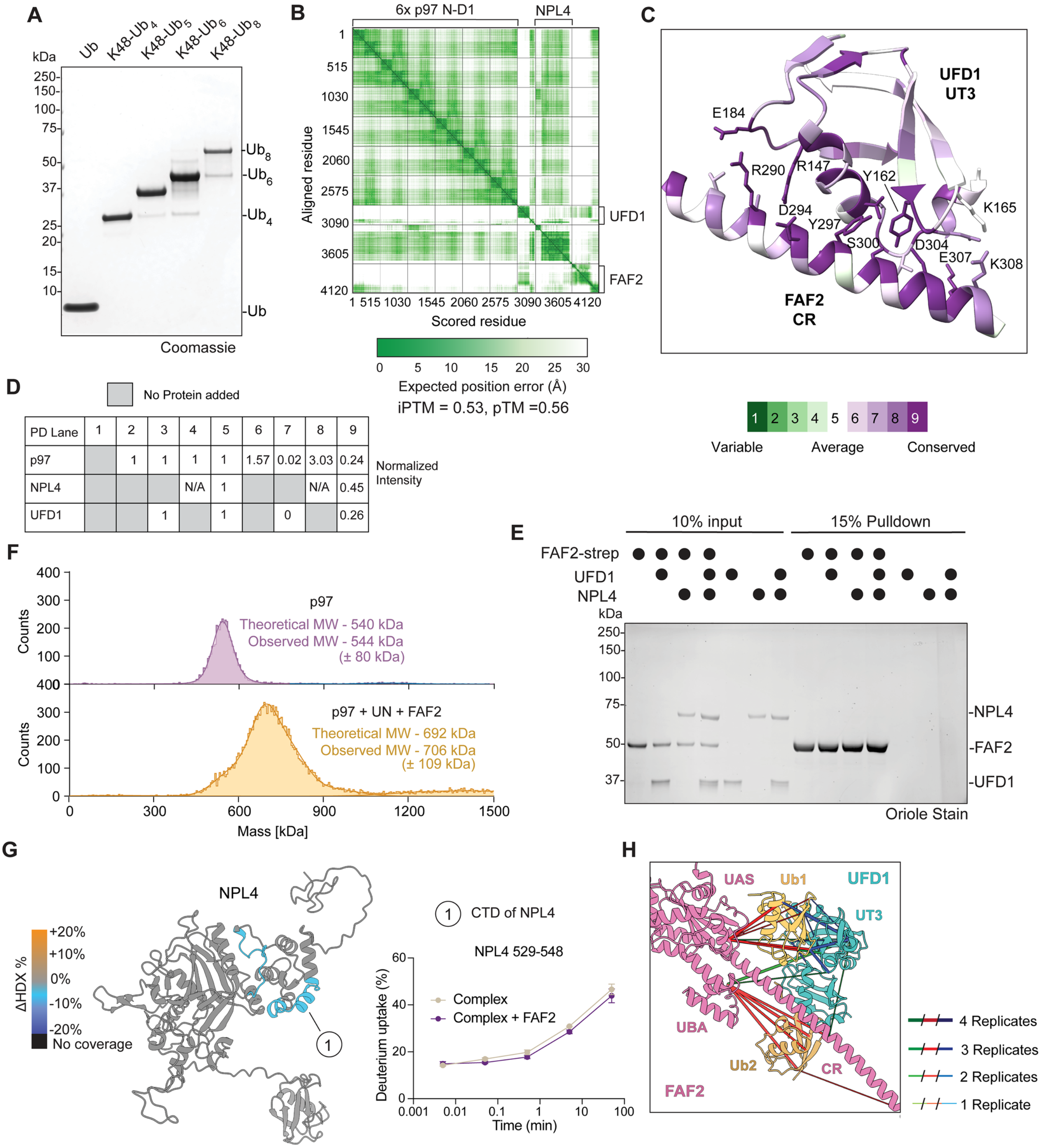
A
Ub
K48-Ub4
K48-Ub5
K48-Ub6
K48-Ub8
kDa
250
150
100
75
50
37
25
20
15
10
Ub8
Ub6
Ub4
Ub
Coomassie
B
6x p97 N-D1
NPL4
UFD1
FAF2
Aligned residue
Scored residue
1 515 1030 1545 2060 2575 3090 3605 4120
Expected position error (Å)
0 5 10 15 20 25 30
iPTM = 0.53, pTM =0.56
C
UFD1
UT3
FAF2
CR
E184
R290
R147
Y162
D294
Y297
S300
D304
E307
K308
K165
1 2 3 4 5 6 7 8 9
Variable
Average
Conserved
D
No Protein added
PD Lane | 1 | 2 | 3 | 4 | 5 | 6 | 7 | 8 | 9
p97 | | 1 | 1 | 1 | 1 | 1.57 | 0.02 | 3.03 | 0.24
NPL4 | | | | N/A | 1 | | | N/A | 0.45
UFD1 | | | 1 | | 1 | | 0 | | 0.26
Normalized Intensity
E
10% input
15% Pulldown
FAF2-strep
UFD1
NPL4
kDa
250
150
100
75
50
37
NPL4
FAF2
UFD1
Oriole Stain
F
p97
Theoretical MW - 540 kDa
Observed MW - 544 kDa
(± 80 kDa)
p97 + UN + FAF2
Theoretical MW - 692 kDa
Observed MW - 706 kDa
(± 109 kDa)
Counts
Mass [kDa]
0 300 600 900 1200 1500
G
NPL4
ΔHDX %
+20%
+10%
0%
-10%
-20%
No coverage
1
1 CTD of NPL4
NPL4 529-548
Complex
Complex + FAF2
Deuterium uptake (%)
Time (min)
0.001 0.01 0.1 1 10 100
H
UAS
Ub1
UFD1
UT3
UBA
Ub2
CR
FAF2
4 Replicates
3 Replicates
2 Replicates
1 Replicate



© The Author(s)

**Figure EV2. Analysis of p97: UN: FAF2 complex formation.**

(**A**) Coomassie-stained SDS–PAGE of K48-linked ubiquitin chains used for substrate-stimulated ATPase activity assay. (**B**) PAE plot of the represented AF3 model of p97(ND1)-NPL4-UFD1 complex. (**C**) Conservation analysis of FAF2 and UFD1 generated by ConSurf from the sequences of 150 eukaryotic species. Conservation of each residue is represented by a score between 1 and 9, with 1 being the least conserved and 9 being the most conserved. ConSurf analysis is then mapped onto the AF3 prediction of the p97-UFD1-FAF2 complex, and the UFD1-FAF2 interface is highlighted. (**D**) Quantification of pulldown samples in Fig. 3C. Values are represented by normalising the intensity of each protein under a specific condition to that in the condition where FAF2 was present. (**E**) Strep pull-down of Twin Strep-tagged FAF2 was performed with UFD1, NPL4, or the UFD1-NPL4 complex, followed by Oriole staining. (**F**) Mass photometry analysis of cross-linked p97-UFD1-NPL4-FAF2 complex. Conditions include 5 µM p97 hexamer alone (purple), 5 µM p97 hexamer + 10 µM UFD1-NPL4 + 10 µM FAF2 (orange), in the presence of 600 µM DSSO crosslinker. Histograms are representative of $n = 3$ independent experiments. MW, molecular weight. (**G**) ΔHDX mapping of NPL4 bound to p97 and UFD1 upon FAF2 binding (left). Regions with reduced solvent exchange are shown in blue, increased exchange in orange, no coverage in black, and no significant change (−5% to +5%) in grey. Deuterium uptake kinetics of the peptide in the NPL4 CTD domain showing FAF2-dependent protection (right). Data represent mean ± SD ($n = 3$ independent replicates). (**H**) Experimental crosslinks mapped onto the AlphaFold3 model of the p97-UFD1-NPL4-FAF2-polyUb complex. Cross-linked residue pairs are shown as connecting lines (colouring is the same as Fig. 3F), supporting the predicted complex architecture. Source data are available online for this figure.

© The Author(s)

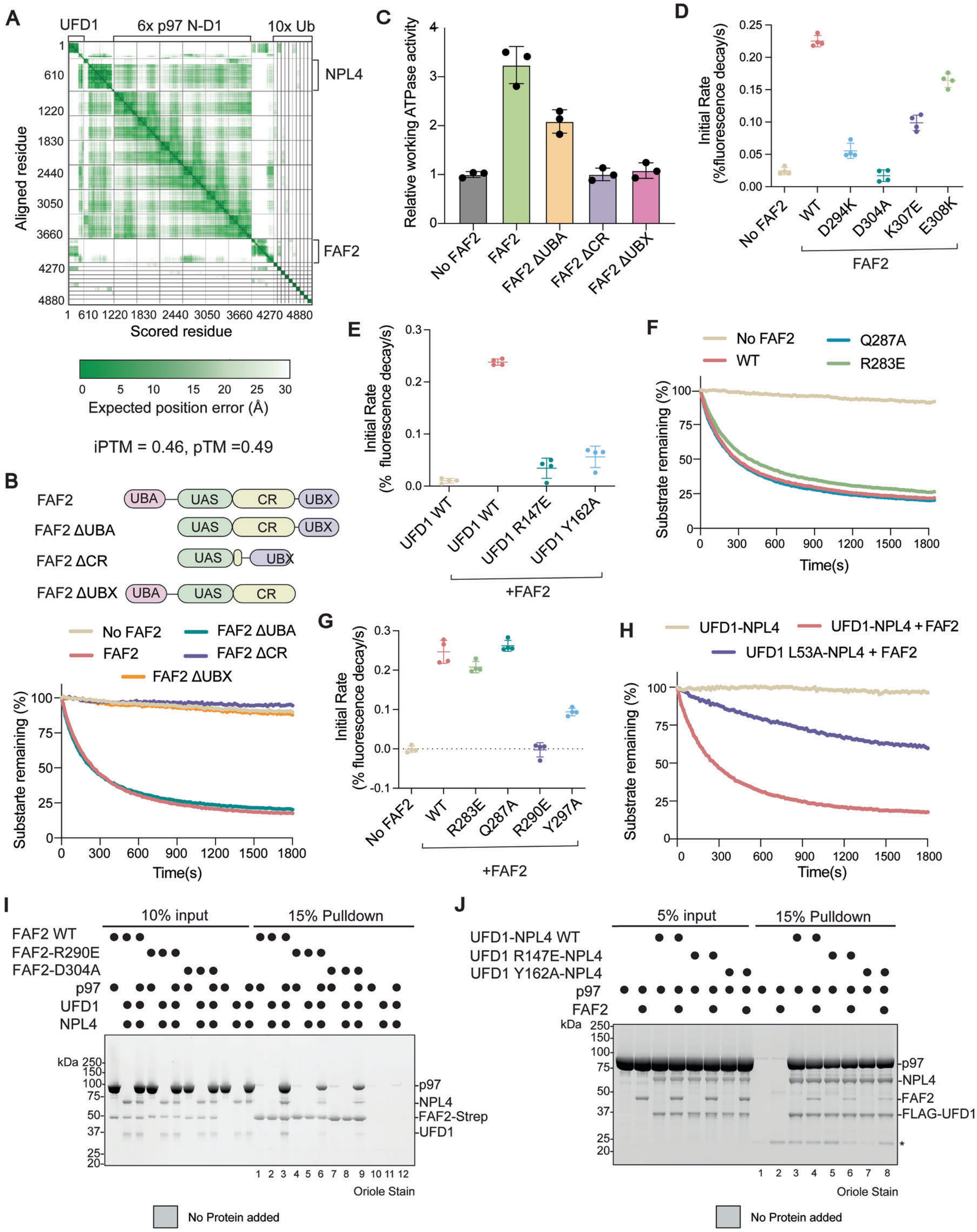


| PD Lane | 1 | 2 | 3 | 4 | 5 | 6 | 7 | 8 | 9 | 10 | 11 | 12 |
|---|---|---|---|---|---|---|---|---|---|---|---|---|
| p97 | 0.02 | | 1 | 0.01 | | 0.54 | 0.01 | | 0.3 | N/A | | N/A |
| NPL4 | | N/A | 1 | | N/A | 0.53 | | N/A | 0.36 | | N/A | N/A |
| UFD1 | | N/A | 1 | | N/A | 0.28 | | N/A | 0.14 | | N/A | N/A |

Normalized Intensity

| PD Lane | 1 | 2 | 3 | 4 | 5 | 6 | 7 | 8 |
|---|---|---|---|---|---|---|---|---|
| FAF2 | | N/A | | 1 | | 0.47 | | 0.33 |

Normalized Intensity

 © The Author(s)

**Figure EV3. Analysis of FAF2, UFD1 and ubiquitin interactions.**

(**A**) PAE plot for the AF3 model of p97(ND1)-UFD1-NPL4-FAF2 with ten ubiquitin molecules. (**B**) Top: Schematic representation of FAF2 truncation constructs used. Bottom: Unfolding of $Ub_6$-Eos by p97-UFD1-NPL4 in the presence of WT FAF2 or FAF2 truncation variants. (**C**) ATPase activity of p97 in the presence of the indicated FAF2 truncations and $Ub^L$ substrate, normalised to ATPase activity of p97 in the presence of the UFD1–NPL4 complex and substrate. Data represent the mean ± SD ($n = 3$ technical replicates) and are extended from the data shown in Fig. 2F. (**D**) Comparison of substrate unfolding kinetics for FAF2 mutants predicted to impair UFD1 interactions. (**E**) Comparison of substrate unfolding kinetics for UFD1 mutants predicted to impair FAF2 interactions. (**F**, **G**) Comparison of substrate unfolding kinetics for FAF2 mutants predicted to impair UFD1 and ubiquitin interactions. (**H**) Unfolding activity of FAF2 mutant predicted to abrogate interaction with proximal Ub, measured in the presence of UFD1-NPL4. (**I**) Streptavidin pulldown assays assessing interactions between FAF2 (WT and indicated mutants) and the p97-UFD1-NPL4 complex. Intensities of each protein were normalised to the amount of bait (FAF2) recovered in each pulldown and to the amount pulled down with WT FAF2. (**J**) FLAG pulldown assays assessing binding of wild-type or mutant Flag-tagged UFD1-NPL4 complexes to p97 and FAF2. The asterisk (*) indicates non-specific bands, likely resulting from the light chain of the antibody. Intensities of FAF2 were normalised to the amount pulled down with WT UFD1-NPL4. In (**B**, **F**, **H**) traces were normalised to fluorescence intensity at time 0 and substrate-only controls. For (**B**, **D**–**H**), data were shown as mean ± SD for $n = 4$ technical replicates. Source data are available online for this figure.

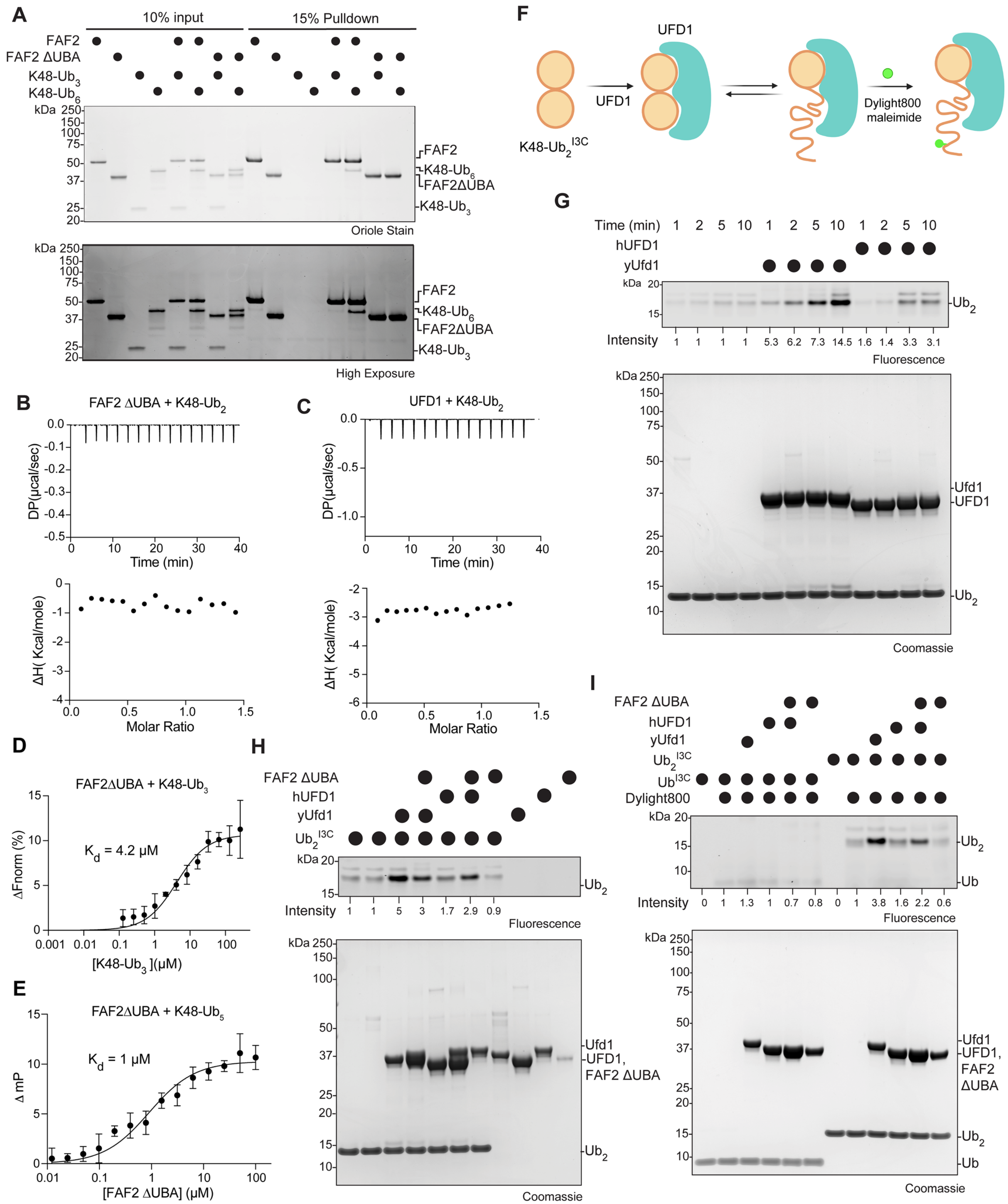
A
10% input
15% Pulldown
FAF2
FAF2 ΔUBA
K48-Ub3
K48-Ub6
kDa 250
150
100
75
50
37
25
20
FAF2
K48-Ub6
FAF2ΔUBA
K48-Ub3
Oriole Stain
High Exposure
B
FAF2 ΔUBA + K48-Ub2
DP(μcal/sec)
Time (min)
ΔH( Kcal/mole)
Molar Ratio
C
UFD1 + K48-Ub2
D
FAF2ΔUBA + K48-Ub3
Kd = 4.2 μM
ΔFnorm (%)
[K48-Ub3](μM)
E
FAF2ΔUBA + K48-Ub5
Kd = 1 μM
Δ mP
[FAF2 ΔUBA] (μM)
F
UFD1
K48-Ub2I3C
Dylight800 maleimide
G
Time (min)
hUFD1
yUfd1
Ub2
Intensity
Fluorescence
Ufd1
UFD1
Coomassie
H
FAF2 ΔUBA
Ub2I3C
UFD1, FAF2 ΔUBA
I
UbI3C
Dylight800
Ub

 © The Author(s)

**Figure EV4. Analysis of ubiquitin binding and unfolding by UFD1 and FAF2.**

(**A**) Pulldown assays testing interactions between strep-tagged FAF2 and K48-linked ubiquitin chains ($Ub_3$ and $Ub_6$) in the absence of the UBA domain. Full-length FAF2 was included as a control. (**B**) Isothermal titration calorimetry (ITC) analysis of FAF2 ΔUBA binding to K48-linked diUb. Representative thermograms (top) and binding isotherms (bottom) are shown. (**C**) Isothermal titration calorimetry (ITC) analysis of UFD1 binding to K48-linked di-Ub. Representative thermograms (top) and binding isotherms (bottom) are shown. (**D**) Analysis of binding between FAF2 ΔUBA-Twin strep and K48-linked tri-Ub using microscale thermophoresis. FAF2 ΔUBA-Twin strep was labelled with NHS-647 dye and mixed with different concentrations of K48-$Ub_3$. Data from 65 independent runs were averaged and fitted into a one-site specific binding function in Prism to calculate the dissociation constant ($K_d$). (**E**) Analysis of binding between FAF2 ΔUBA-Twin strep and K48-linked penta-Ub using fluorescence polarisation. K48-$Ub_5$ was labelled with BODIPY-maleimide dye and mixed with varying concentrations of FAF2 ΔUBA. Data were shown for $n = 3$ technical replicates. Differences in polarisation (ΔmP) was calculated by subtracting the mP value of the only tracer (K48-$Ub_5$) control from that of each condition. Data were fitted into a one-site specific binding function in Prism to calculate the dissociation constant ($K_d$). (**F**) Schematic of the dye accessibility assay. UFD1-bound K48-linked I3C di-ubiquitin undergoes unfolding and exposure of the C3 residue, which is covalently labelled with DyLight800 maleimide, providing a cumulative readout of ubiquitin unfolding over time. (**G**) Time-course of K48-linked I3C di-ubiquitin unfolding measured by DyLight800 maleimide labelling of the exposed C3 residue in the presence of yeast Ufd1 or human UFD1. Fluorescence scan (top) and Coomassie staining (bottom) of the corresponding samples are shown, with relative band intensities indicated. (**H**) As in (**G**), testing the ability of FAF2ΔUBA to enhance unfolding of K48-linked I3C di-ubiquitin by yeast and human UFD1. Fluorescence scan (top) and Coomassie staining (bottom) of the corresponding samples are shown. (**I**) Comparing unfolding of K48-linked I3C di-ubiquitin versus I3C monoubiquitin in the presence of FAF2ΔUBA and UFD1 homologues. Fluorescence scan (top) and Coomassie staining (bottom) of the corresponding samples are shown. Source data are available online for this figure.

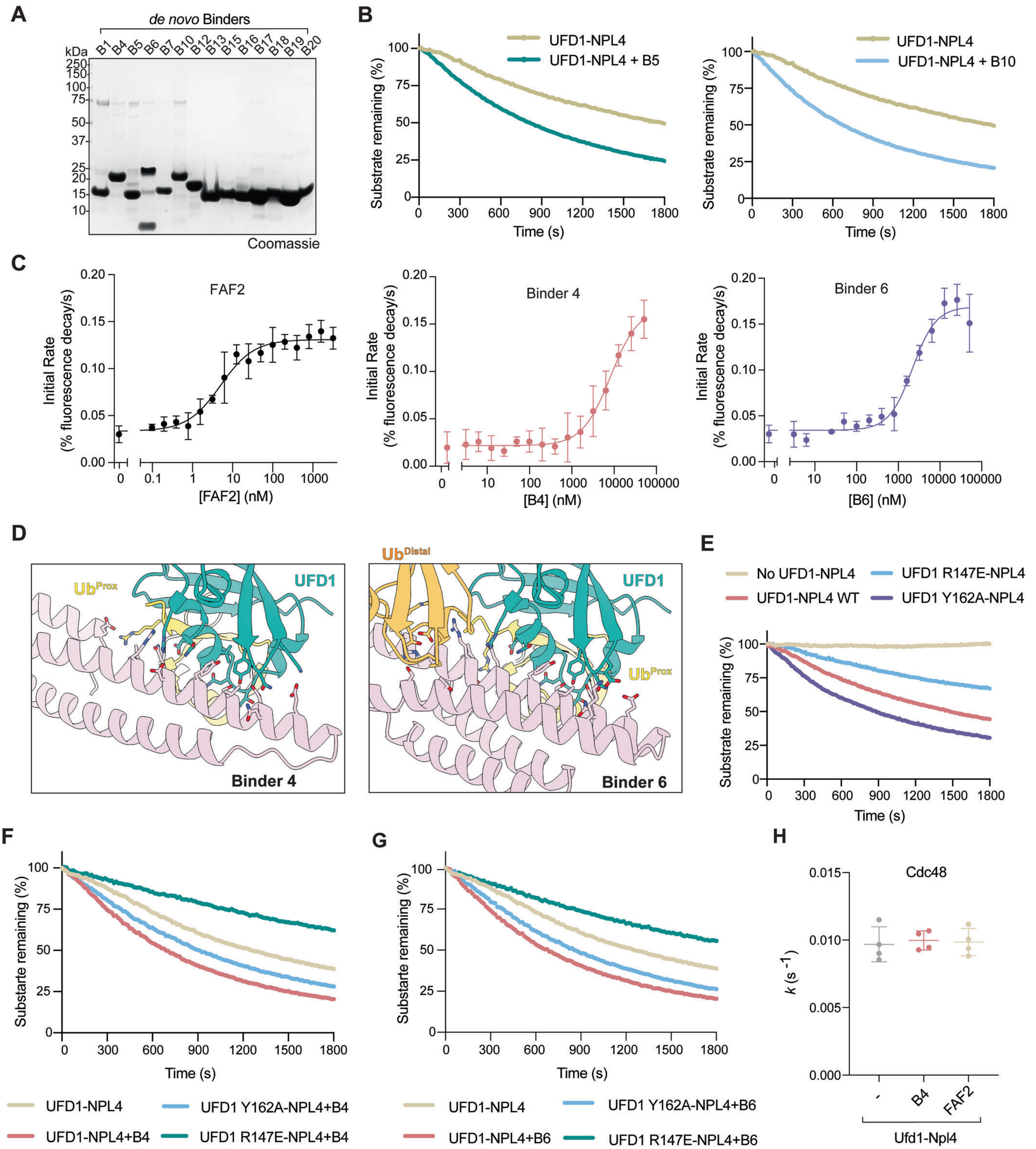
A
de novo Binders
kDa
B1 B4 B5 B6 B7 B10 B12 B13 B15 B16 B17 B18 B19 B20
250 150 100 75 50 37 25 20 15 10
Coomassie
B
UFD1-NPL4
UFD1-NPL4 + B5
UFD1-NPL4 + B10
Substrate remaining (%)
Time (s)
C
FAF2
Binder 4
Binder 6
Initial Rate (% fluorescence decay/s)
[FAF2] (nM)
[B4] (nM)
[B6] (nM)
D
Ub^Prox
UFD1
Ub^Distal
Binder 4
Binder 6
E
No UFD1-NPL4
UFD1-NPL4 WT
UFD1 R147E-NPL4
UFD1 Y162A-NPL4
Substrate remaining (%)
Time (s)
F
Substarte remaining (%)
Time (s)
UFD1-NPL4
UFD1-NPL4+B4
UFD1 Y162A-NPL4+B4
UFD1 R147E-NPL4+B4
G
Substarte remaining (%)
Time (s)
UFD1-NPL4
UFD1-NPL4+B6
UFD1 Y162A-NPL4+B6
UFD1 R147E-NPL4+B6
H
Cdc48
k (s⁻¹)
-
B4
FAF2
Ufd1-Npl4

 © The Author(s)

**Figure EV5. Characterisation of de novo binders.**

(**A**) Coomassie-stained SDS–PAGE shows 14 purified de novo binders used for experimental validation. (**B**) Unfolding activity of p97-UFD1-NPL4 complex in the presence of Binder 5 (left) and Binder 10 (right) shows modest activation of $Ub^L$ substrate unfolding. Traces were normalised to fluorescence intensity at time 0 and substrate-only controls. (**C**) Initial rate of unfolding $Ub^L$-Eos as a function of FAF2, Binder 4 and Binder 6 concentration. Initial rate was calculated from linear regression fitting of the first ~2 min of the unfolding trace. Data shown as mean ± SD for $n = 3$ technical replicates for FAF2, $n = 4$ technical replicates for binders. (**D**) Structural models of UFD1 (teal) and two ubiquitin molecules bound to Binder 4 and Binder 6. Ubiquitin molecules are shown in yellow (proximal) and orange (distal), with interface residues highlighted as sticks. (**E**) Unfolding activity of WT UFD1-NPL4, UFD1·Y162A-NPL4 and UFD1·R147E-NPL4 complexes for $Ub^L$ substrate. (**F**) Unfolding activity of WT and mutant UFD1-NPL4-p97 complexes in the presence of Binder 4, measured by fluorescence decay of $Ub^L$-Eos substrate over time. (**G**) As in (**F**), showing the effect of UFD1 mutations on Binder 6-dependent activation of substrate unfolding. (**H**) Comparison of the unfolding rate constants of yeast Cdc48-Ufd1-Npl4 for $Ub^L$ substrate in the presence of Binder 4 and FAF2. For (**E**–**H**), Traces were normalised to fluorescence intensity at time 0 and substrate-only controls. Unfolding rate $k$ ($s^{-1}$) was determined by fitting the unfolding trace to a single exponential decay. Data shown as mean ± SD for $n = 4$ technical replicates for (**E**, **H**) and mean ± SD for $n = 3$ technical replicates for (**F**, **G**). Source data are available online for this figure.
