# Supplementary material for: FAF1 and FAF2 enhance unfolding by p97-UFD1-NPL4 complex enabling rational design of p97 activators
Source: EMBO J. 2026 Aug 15;45(18):6595–629. doi: 10.1038/s44318-026-00894-x (PMC13578712; doi:10.1038/s44318-026-00894-x)
Supplement: Supplementary file 2 [file 44318_2026_894_MOESM1_ESM.pdf]

Appendix to “FAF1 and FAF2 enhance unfolding by p97-UFD1-NPL4 complex enabling rational design of p97 activators”

Table of Contents

Appendix Figure S1: DSSO-mediated cross-linking of p97:UFD1-NPL4:FAF2 complex - / + ubiquitin 3

Appendix Figure S2: FAF1 engages p97-UFD1-NPL4 via a binding mode similar to FAF2 4

Appendix Figure S3: Predicted aligned error (PAE) plots of UT3-2xUb and UT3-2xUb-FAF2 CR complexes generated by AF3. 6

Appendix Figure S4: An overview of UFD1 binder design pipelines. 7

Appendix Table S1: HDX-MS statistics 9

AppendixTable S2: RFdiffusion scoring metrics for protein binder designs 10

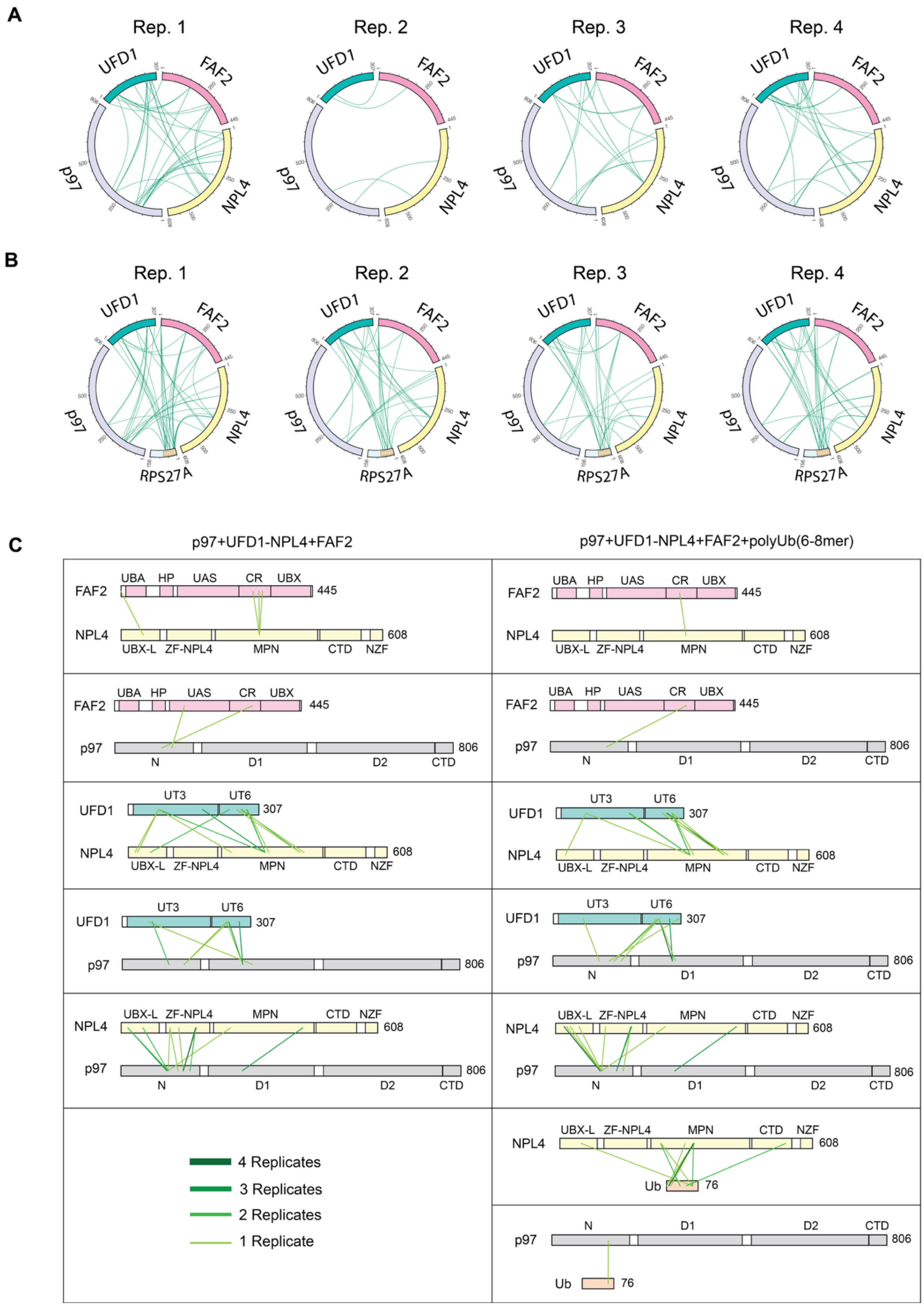
A
Rep. 1
Rep. 2
Rep. 3
Rep. 4
UFD1
FAF2
NPL4
p97
B
RPS27A
C
p97+UFD1-NPL4+FAF2
p97+UFD1-NPL4+FAF2+polyUb(6-8mer)
UBA HP UAS CR UBX
445
UBX-L ZF-NPL4 MPN CTD NZF
608
N D1 D2 CTD
806
UT3 UT6
307
Ub
76
4 Replicates
3 Replicates
2 Replicates
1 Replicate

Appendix Figure S1: DSSO-mediated cross-linking of p97:UFD1-NPL4:FAF2 complex - / + ubiquitin.

(A) Circular diagrams of DSSO-mediated intermolecular cross-links observed between components of the p97:UFD1-NPL4:FAF2 ternary complex are shown for 4 independent replicates.

(B) Circular diagrams of interprotein cross-links observed between components of the quaternary complex formed between p97:UFD1-NPL4:FAF2 and a chain of K48-linked polyubiquitin (denoted as the first 76 amino acids of Ribosomal Protein S27a).

(C) The domain architectures of the indicated complex components are shown, highlighting the sites of chemical cross-linking in n = 4 independent replicates. Cross-links are represented by straight lines, the colour and width of which indicates reproducibility across different replicates. Panels on the left indicate the cross-links observed in the absence of ubiquitin, those on the right highlight those cross-links recorded in the presence of poly-Ub.

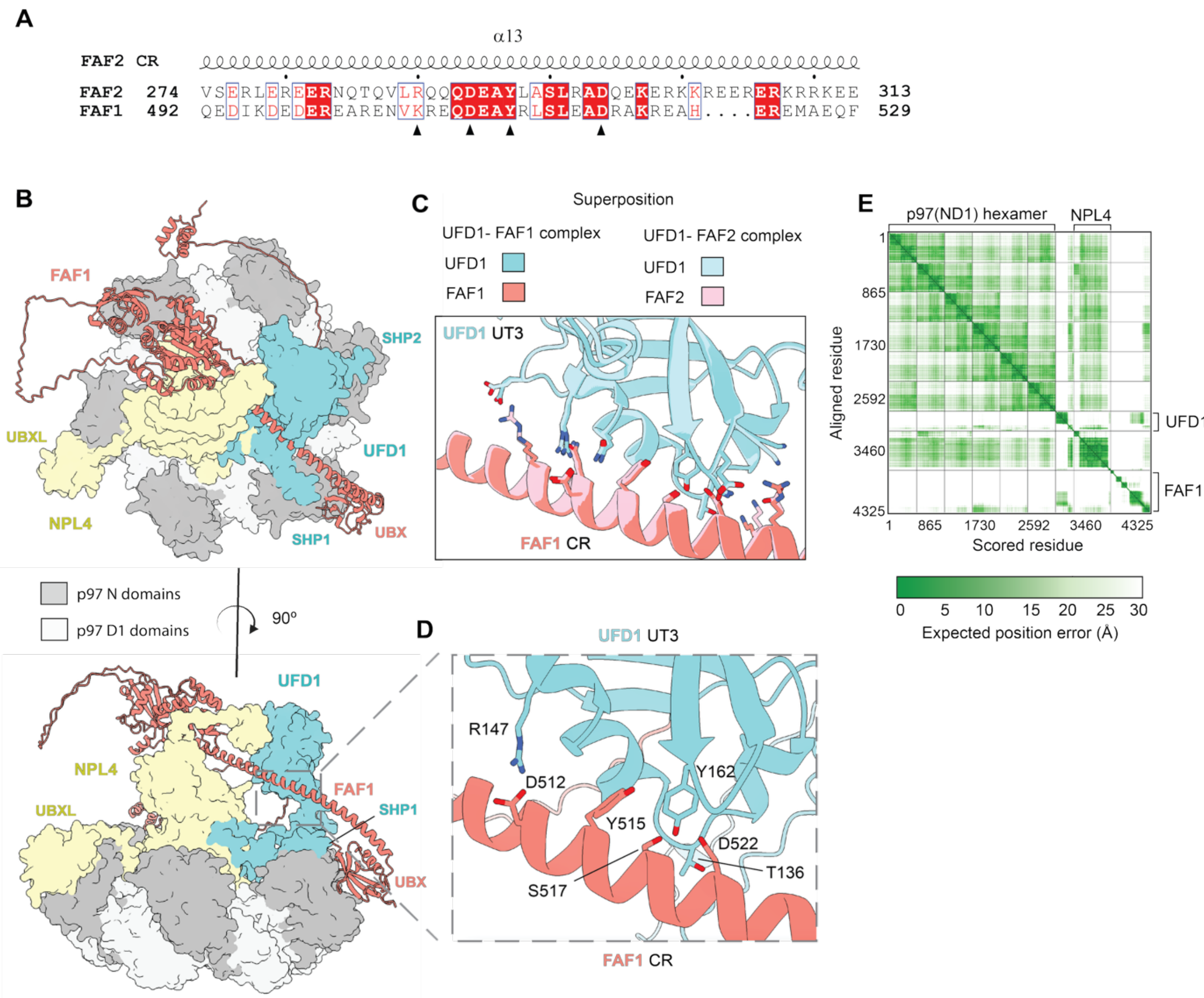

Appendix Figure S2: FAF1 engages p97-UFD1-NPL4 via a binding mode similar to FAF2.

(A) Sequence alignment of the CR of human FAF1 and FAF2. Black triangles denote residues previously shown to contribute to UFD1 binding and enhanced unfolding activity. (B) AF3-based structural model of the p97(ND1) hexamer bound to the UFD1-NPL4 complex and FAF1. p97 N domains are shown in dark grey and D1 domains in light grey, NPL4 in yellow, UFD1 in teal, and FAF1 in salmon. The top panel shows a top view of the hexamer, highlighting the position of FAF1 relative to the NPL4 UBX domain, UFD1 SHP domains, and p97 N domains. The bottom panel shows a side view of the hexamer, illustrating how FAF1 binds the p97 N domain while bridging the UFD1-NPL4 complex.

(C) Structural superposition of FAF1-UFD1-NPL4-p97 and FAF2-UFD1-NPL4-p97 AF models, revealing a conserved binding mode. Key interface residues conserved between FAF1 and FAF2 are shown as sticks.

(D) Enlarged view of the FAF1-UFD1 interface showing specific residue-residue contacts. Key interacting residues are labelled.

(E) PAE plot for the AlphaFold3 model of p97(ND1)-UFD1-NPL4 with FAF1.

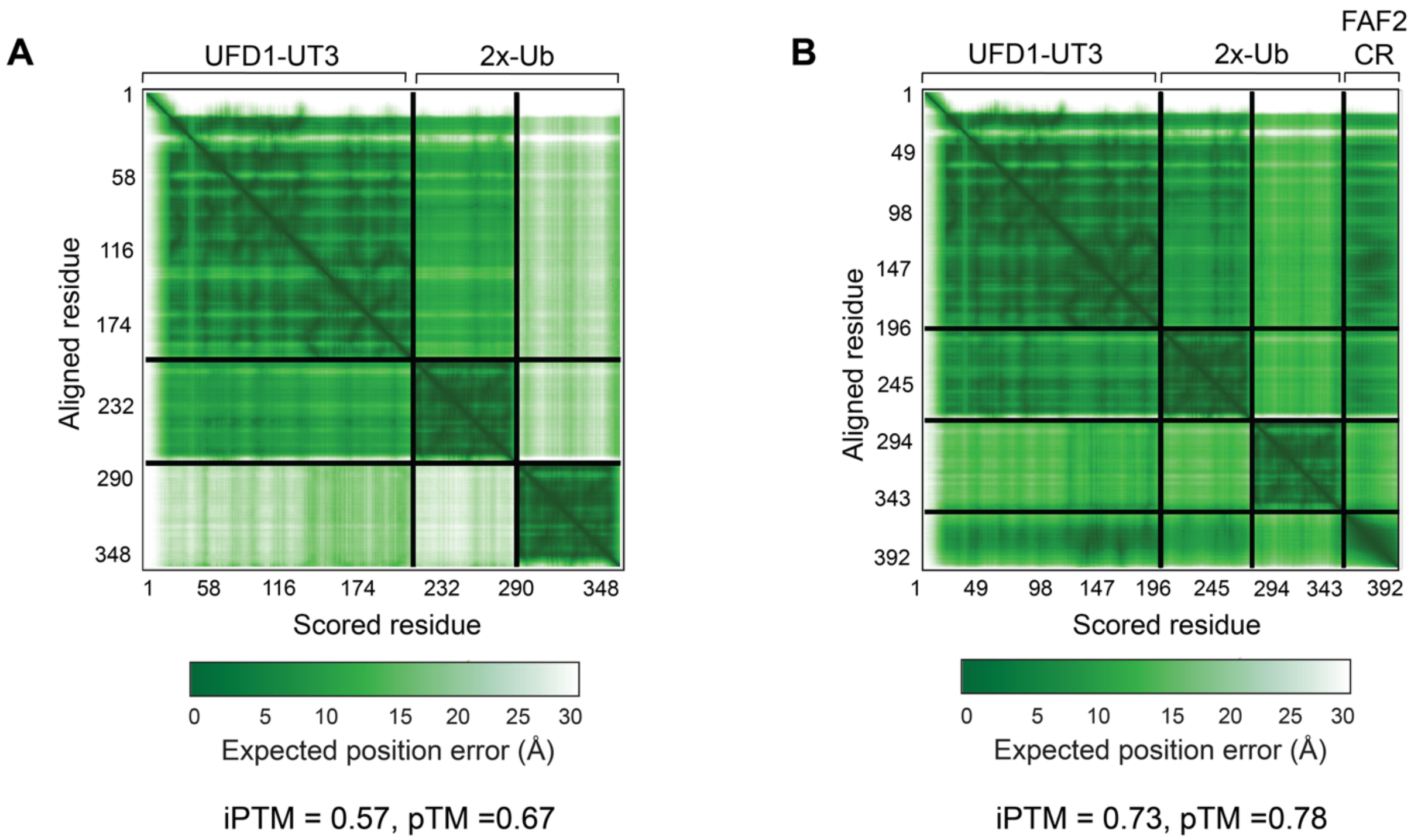

Appendix Figure S3: Predicted aligned error (PAE) plots of UT3-2xUb and UT3-2xUb-FAF2 CR complexes generated by AF3.

(A) PAE plot for the complex containing the UFD1-UT3 construct bound to di-Ub (2x Ub). The color indicates the expected positional error (Å), with darker green representing higher confidence and lighter colors indicating greater uncertainty in relative positioning of the residues. The model yielded an interface predicted TM-score (ipTM) of 0.57 and a predicted TM-score (pTM) of 0.67.

(B) PAE plot for the complex containing UFD1-UT3, 2x Ub, and the FAF2-CR. Compared to the model in panel A, the inclusion of FAF2-CR resulted in improved confidence in the relative placement of the components, reflected by lower inter-domain PAE values and higher model confidence scores (ipTM = 0.73, pTM = 0.78).

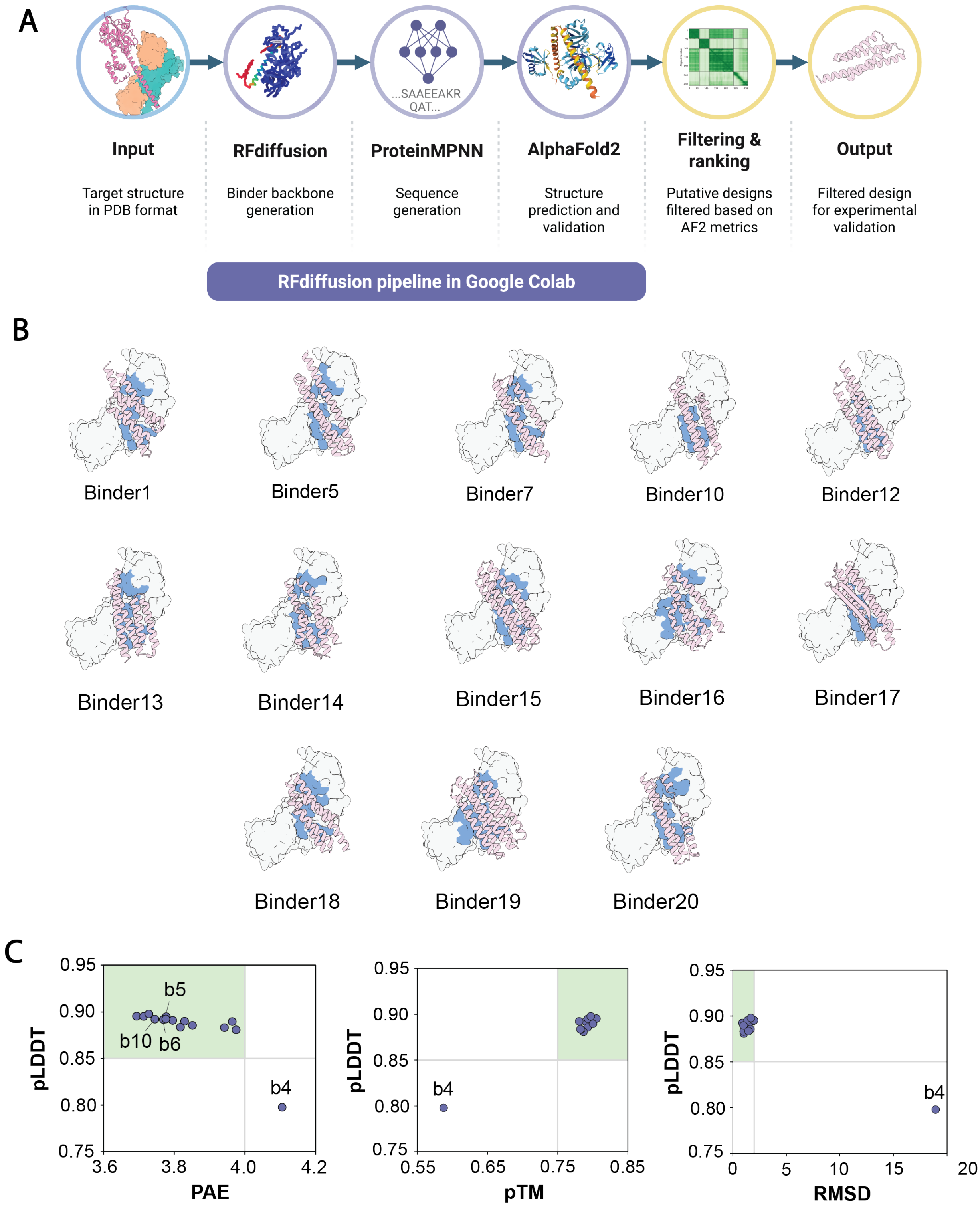

Appendix Figure S4: An overview of UFD1 binder design pipelines.

(A) Schematic representation of the RFdiffusion pipeline used to design p97 activators. Given an AlphaFold prediction of the p97-UFD1-NPL4-FAF2-diUb complex as a target structure, RFdiffusion generates binder backbones conditioned upon the FAF2 helix. These backbone structures are then run through Protein MPNN to design amino acid sequences encoding the predicted structures, followed by model validation with initial guess AlphaFold2. The resulting designs are then filtered based upon AF2 output metrics.
(B) Designs of potential p97 activators (pink) binding to UFD1 and a K48-linked ubiquitin dimer (shown in grey surface model). The residues on UFD1 and ubiquitin predicted to make the strongest and most stable contacts with the binder are shown in blue.
(C) AF2 metrics of potential activators chosen by experimental validation are shown here. Regions corresponding to accepted designs are shown in green (PAE <4, pLDDT >0.85, pTM >0.75, RMSD <2). Binder4 initially failed on all metrics (as shown), but subsequent remodelling of the sequence in ColabFold (Mirdita et al. 2022) using the RFdiffusion input model as a custom template to guide the prediction improved the metrics considerably (pLDDT = 0.86, pTM = 0.76).

Appendix Table S1: HDX-MS statistics.

DataSet 1:

	p97	UFD1	NPL4	Overall
# of Peptides	323	36	86	445
Coverage (%)	96.4%	92.5%	91.1	93.9%
Gaps in Coverage	(-6-4),408-453	86-97, 225-232,	336-339,481-494,604-609	
Av. Length	15.3	17.9	17.1	15.9
Redundancy	6.1	2.1	2.4	4.1
Time Points	0.3s[1] / 3s/ 30s/ 300s/ 3000s			
Replicates	3 ND, 3D			
Replicability[2]	0.09 Da			
Significance Threshold[3]	>5%/ >0.5 Da/ passes T-test			

DataSet 2:

	FAF2 Δ90-118
# of Peptides	122
Coverage (%)	87.7%
Gaps in Coverage	81-90, (90-118), 119-140, 300-324
Av. Length	21.0
Redundancy	5.8
Time Points	0.3s[1] / 3s/ 30s/ 300s/ 3000s
Replicates	3 ND, 3D
Replicability[2]	0.2 Da
Significance Threshold[3]	>5%/ >1.0 Da/ passes T-test

1 0.3 s timepoint created by pipetting 3 s by hand with ice-cold solutions.

2 Mean standard deviation between replicates

3 A peptide must meet all these criteria to have a significant change in solvent exchange. The threshold is set as 5σ.

AppendixTable S2: RFdiffusion scoring metrics for protein binder designs.

Binder #	pLDDT	pTM	PAE	RMSD	Amino acid sequence
1	0.89082187	0.79225338	3.79605606	1.31694317	SLEELLKKLALIETTRLRYQQDLAYAQSLVKDQIKENKKKGIENETLENLLKKLDEIEKVL KTEDLETALERSSKLLTEMIDEINKSKDESLKEILEALQKAKTTVESLKKLLEELE
4*	0.7977826	0.58776951	4.10570423	18.9439144	RELSEAELRALLEAAKRAGAPAELIALIQEALRLRTQLLRKQQDAAYTESLIADQKKEAA KTGTEADTTELEKQLAKLKAEAAELQARLAAVLAEIAAA
5	0.89479476	0.79489315	3.77789436	1.95688951	DLEKELEKAYEALQKKIKEAQAEARALFEAARPQLTPEQLALAETALLRFQQDLAYLESL AADQKKEVEKTKKDETIKKALKKLEEMQKQLDELQAIFRAQLEQLLAKAAAARAA
6	0.89188683	0.79795468	3.76990956	1.54283166	AAAEAARRRALIRTRLLRAQQDMAYLLSLLKDQEKEAKKTGDKEKLEKAKENAIKILEKI ATILRSIASEGVAEAKELLKEVEELIKKITEENLPIEEIIKQSEELLAKLQALLAAE
7	0.89003599	0.79382074	3.82986008	1.47030568	DEEALKLFETSLLRLQQDSAYLISLIKDQIKEIEKGKEVNYEKIEKEREELKKLMESQLKL WAERFSPELVAKAAALVEELLKKLDEEMKKLKEKIEKKKKE
10	0.89214611	0.78997982	3.74557893	1.48420465	AEEAEKRKEALAKVEALMKELFAKLEEKLDAKLKAEGVPEELRETRLLRLQQDYAYLMS LLKDQIKELEKKGAPFEEFIKVLKETLEQFLELVKTYLPELEPEAQAAFAKLLASL
12	0.89230782	0.78077936	3.77683283	0.9135927	VLEEFKKAKAAFDAAMAAATATVTALLDALPPTEETQLLRTRFLRFQQDAAYQLSLATD QVKEAEKKGDEEKIKEQVEKVKALVKDVAAAEAAFRAELAALRARLEA
13	0.88542902	0.79289949	3.85172697	1.64689159	ETEKKFQELKKELDKLTKETNEKIAKELEEEAKKDKSVTQEQLETRKLRYQQDLAYVKS LFLDQTKEYIKNKEKKEEILEKTKKEAEELIKKFNSLSPIMAETIKEMLELFEKFV
14	0.88057679	0.78707427	3.97520547	1.04548109	PLEKGKEALEHILKTLKERQKENAAAYEKELASVTPEVRETRKLRAQQDRAYLLSLSMD QFKELAKKGWKLPKSFSEIRKEIEAALGPVPAKGAAPKILAFIKKLEA
15	0.89530259	0.8054353	3.69355593	1.99423647	AVEELLAAIKEAFALLIKKLEENKDNKEKVEAIIKKALELLDLLSELVAGLAALSRRLPADT RAALDTILLRAQQDIAYLKSLLEDQLKEMKKTGDYKTNLKKAIEYIKTEGEKIITLLRIALTR LEAALRAEGREALLAELEALVRAATERVKALIEEVKK
16	0.88298279	0.78349519	3.94219783	1.03257906	KSEEKIKKIEQQLKDFQERVATLKKALERAKKEGTPEEAETIELRLQQDIAYQMSLYVDQ LKEEIKKGGELEVLQAILDALETISKYPEAAKYKPVVEKYKKEIKEL
17	0.89512384	0.79334664	3.71347461	1.39852273	MEEVKELIKAVKEMVDFVLEVVRLILENAPERTLTKTIEVNGETIVSLTLTVKPVTFETEEL RRRFETRALRFQQDVAYHLSLLADQMKELEKAKTEEEKKKIMKKIVETIEKLSEDVSTHL SSMATLLADDKEAVAVLDAMRDAAVKAFTKAAEKLKKLIE
18	0.89775556	0.79732138	3.72848545	1.71081901	MEEKKIEEYKKKSIEENLEALNKVLDSLKNLKQYLERGDEELAKTTRLRLQQDLAYLKSL LQDQIKEAEKLGLKELAEIYKELLKLVEEAEKALETDLDKSEEILEKAKELLEKK
19	0.88339168	0.78265071	3.81778946	1.47169948	AAAAMLAEMKAATEKVLKRIEEAKKSANPVMKKLLDTISDAVRNTTADIENLINTLLNPS ASRETILTNVLRFQQDIAYNISLLLDQAKEYEKETGDEELVKLVKEEADKILKAAQKLADL VFTEPLTPEALAKILAALRELGKLVIEAAEKILEKIEKILK
20	0.88949502	0.80048823	3.96454537	1.01554108	MEEEKEIEKLNKEIVEKVKKEKQPLDTDELLAKMDPSYMPTRMLRIQQDAAYRASLLKD QTKEVLKTKNLSEESKKKLEELTQKEIDALNKETEAQIARMQAAVAAS

* Initial Binder 4 metrics are shown. These are poor, likely due to a failure to utilise initial guess in AlphaFold during the design process. Subsequent remodelling of the binder in ColabFold (Mirdita *et al*, 2022) suggests a pLDDT value of 0.86 and a pTM of 0.76.
