# Supplementary material for: FAF1 and FAF2 enhance unfolding by p97-UFD1-NPL4 complex enabling rational design of p97 activators
Source: EMBO J. 2026 Aug 15;45(18):6595–629. doi: 10.1038/s44318-026-00894-x (PMC13578712; doi:10.1038/s44318-026-00894-x)
Supplement: Supplementary file 3 [file 44318_2026_894_MOESM2_ESM.pdf]

THE EMBO JOURNAL

FAF1 and FAF2 enhance unfolding by p97-UFD1-NPL4 complex enabling rational design of p97 activators

Pritha Dasgupta, Ian Kelsall, Gaurav Anand, Anna Pérez-Ràfols, Thomas Jowitt, Axel Knebel, Robert Gourlay, Glenn Masson, and Yogesh Kulathu

Corresponding author(s): Yogesh Kulathu (y.kulathu@dundee.ac.uk)

Review Timeline:

Submission Date:	3rd Feb 26
Editorial Decision:	19th Mar 26
Revision Received:	22nd Jun 26
Editorial Decision:	15th Jul 26
Revision Received:	29th Jul 26
Accepted:	31st Jul 26

Editor: Hartmut Vodermaier

Transaction Report:

(Note: With the exception of the correction of typographical or spelling errors that could be a source of ambiguity, letters and reports are not edited. Depending on transfer agreements, referee reports obtained elsewhere may or may not be included in this compilation. Referee reports are anonymous unless the Referee chooses to sign their reports.)

Prof. Yogesh Kulathu
University of Dundee
MRC Protein Phosphorylation and Ubiquitylation Unit
Dow Street
Dundee DD1 5EH
United Kingdom

19th Mar 2026

Re: EMBOJ-2026-123779
FAF1 and FAF2 enhance human p97 unfoldase activity enabling rational design of p97 activators

Dear Yogesh,

Thank you again for submitting your manuscript on FAF2-p97 interplay to The EMBO Journal. We have received the reports by three expert referees, which are copied below. The referees overall appreciate the importance of the subject, the experimental quality of the study, as well as the potential interest of your de novo design of p97-activating peptides. At the same time, they also note varying degrees of conceptual precedent for some of the findings of the study, and criticize that description of reported background vs. novel findings is not sufficiently clear throughout the presentation.

Given the apparent elevated interest in the questions addressed in this study (and the fact that we do not consider preprints or studies appearing after your EMBO Press submission as compromising novelty), we decided to offer publication of this work in The EMBO Journal, pending adequate responses to/revisions of the specific points raised in the reports, as well as rewriting to more clearly differentiate truly novel results and conclusions from confirmatory ones. Please keep in mind that it is our policy to consider only a single round of major revision, making it important to adequately answer all comments at the time of resubmission; please do not hesitate to get back to me in case you would like to clarify/discuss any of the referees' points or plans for addressing them ahead of time.

Detailed information on preparing, formatting and uploading a revised manuscript can be found below and in our Guide to Authors, and adhering to them as closely as possible shall greatly facilitate editorial processing upon resubmission. Thank you again for the opportunity to consider this work for The EMBO Journal, and I look forward to your revision in due time.

With kind regards,

Hartmut

Hartmut Vodermaier, PhD
Senior Editor, The EMBO Journal
h.vodermaier@embojournal.org

*** PLEASE NOTE: All revised manuscript are subject to initial checks for completeness and adherence to our formatting guidelines. Revisions may be returned to the authors and delayed in their editorial re-evaluation if they fail to comply to the following requirements. As a first step please read our guidelines for revised submissions:
https://link.springer.com/journal/44318/submission-guidelines#cms-Revised-submissions

1) Every manuscript requires a Data Availability section (even if only stating that no deposited datasets are included). Primary datasets or computer code produced in the current study have to be deposited in appropriate public repositories prior to resubmission, and reviewer access details provided in case that public access is not yet allowed.

2) Each figure legend must specify
- size of the scale bars that are mandatory for all micrograph panels
- the statistical test used to generate error bars and P-values
- the type error bars (e.g., S.E.M., S.D.)
- the number (n) and nature (biological or technical replicate) of independent experiments underlying each data point
- Figures may not include error bars for experiments with n<3; scatter plots showing individual data points should be used instead.

3) Revised manuscript text (including main tables, and figure legends for main and EV figures) has to be submitted as editable text file (e.g., .docx format). We encourage highlighting of changes (e.g., via text color) for the referees' reference.

4) Each main and each Expanded View (EV) figure should be uploaded as individual production-quality files (preferably in .eps, .tif, .jpg formats). For suggestions on figure preparation/layout, please refer to our Figure Preparation Guidelines: https://media.springernature.com/original/springer-cms/rest/v1/content/27825798/data/v1

5) Point-by-point response letters should include the original referee comments in full together with your detailed responses to them (and to specific editor requests if applicable), and also be uploaded as editable (e.g., .docx) text files.

6) Please complete our Author Checklist, and make sure that information entered into the checklist is also reflected in the manuscript; the checklist will be available to readers as part of the Review Process File.

7) All authors listed as (co-)corresponding need to deposit, in their respective author profiles in our submission system, a unique ORCiD identifier linked to their name. Please see our Guide to Authors for detailed instructions.

8) Please note that supplementary information at EMBO Press has been superseded by the 'Expanded View' for inclusion of additional figures, tables, movies or datasets; with up to five EV Figures being typeset and directly accessible in the HTML version of the article.

9) To facilitate reproducibilty and cross-laboratory adoption of methodologies, please structure the Materials & Methods section as outlined in our guide to authors, including a completed Reagents and Tools Table.

10) Digital image enhancement is acceptable practice, as long as it accurately represents the original data and conforms to community standards. If a figure has been subjected to significant electronic manipulation, this must be clearly noted in the figure legend and/or the 'Materials and Methods' section. The editors reserve the right to request original versions of figures and the original images that were used to assemble the figure. Finally, we generally encourage uploading of numerical as well as gel/blot image source data.

At EMBO Press we ask authors to provide source data for the main manuscript figures. You will receive a separate email with instructions for providing source data with your revised manuscript, including how to upload and organize the files.

In the interest of ensuring the conceptual advance provided by the work, we recommend submitting a revision within 3 months (17th Jun 2026). Please discuss the revision progress ahead of this time with the editor if you require more time to complete the revisions. Use the link below to submit your revision:

Link Not Available

Referee #1:

The manuscript by Dasgupta et al. reports the discovery that substrate unfolding by the p97-Ufd1-Npl4 (p97-UN) complex is activated by adaptors containing UBX domains. The study focuses on two related adaptors, FAF1 and FAF2. Through a combination of biochemical reconstitution, AlphaFold3 structural modeling, crosslinking mass spectrometry, HDX-MS, mutational analysis, and computational protein design, the authors propose a compelling mechanism by which FAF1 and FAF2 potentiate substrate unfolding by p97-UN. The identification of an "activation motif" within FAF2, together with the derivation of de novo binders based on this region that recapitulate stimulation of p97-UN, is particularly elegant and represents a conceptual advance. Overall, the data are compelling, the experiments appear to be carefully executed, and the conclusions are largely supported by the data.
Below are suggestions aimed at clarifying mechanistic interpretation and improving presentation.

Major comments
1. Most experiments with FAF2 are performed with a construct lacking its membrane-targeting region. Given that endogenous FAF2 is ER-anchored, it would be appropriate to more explicitly discuss how removal of the membrane-binding region could influence effective concentration, spatial organization, and/or stoichiometry relative to p97-UN. While the truncation is entirely reasonable for in vitro reconstitution, the manuscript would benefit from acknowledging how membrane tethering might alter the complex in vivo, and to what extent the mechanistic conclusions are expected to extrapolate to the membrane-bound state.
2. The crosslinking MS and HDX-MS data appear broadly consistent with the AlphaFold3 model. However, it would strengthen the manuscript to more clearly articulate what unique mechanistic insights these approaches provide beyond structural validation. For example, do specific crosslinks discriminate between alternative structural arrangements? Do the data validate interfaces that are not confidently predicted by AF3? Do ubiquitin-dependent changes in crosslinking or HDX patterns reveal conformational rearrangements that extend beyond the static model? A more explicit integration of these datasets with the proposed activation mechanism would clarify their added value.

3. The FAF2 D304A mutant shows reduced binding to p97-UN, yet a detectable amount of p97 and Npl4 is still recovered in IPs (Fig. EV4G; Fig. 5H). Despite this residual association, the mutant behaves similarly to the "no FAF2" condition in the unfolding assay. This suggests that reduced binding alone may not fully explain the strong functional defect. The authors should clarify whether the degree of binding reduction is quantitatively sufficient to account for the loss of activity, or consider the possibility that D304A selectively perturbs a conformational state required for activation rather than simply weakening bulk association.
4. In Fig. EV4H, substantial recovery of FAF2 is observed in Ufd1 IPs for the R147E and Y162A mutants. These data do not appear to demonstrate strong disruption of the FAF2-Ufd1 interaction as stated (lines 372-373). If the interaction is only partially reduced, this should be quantified and described more cautiously. As currently presented, the data seem more consistent with partial impairment rather than a strong disruption.
5. The binder activation experiments are elegant and conceptually powerful. As a potentially high-impact extension, it would be informative to test whether the most potent binders can rescue unfolding defects in p97 loss-of-function mutants (e.g., D395G). Similarly, testing whether binder-mediated activation is lost or reduced in the Ufd1 UT3 mutants (R147E and Y162A) would help determine whether the designed binders operate through the same interface as endogenous FAF2.
6. To fully establish necessity and sufficiency of the activating motif, it would strengthen the claim to test the isolated 286-312 region in a minimal format. Such an experiment would further define the minimal requirements for activation.
7. Please describe expression, purification, and quality control of the designed binders in the Methods.

Minor Comments
1. Fig. 2C: Consider reporting the x-axis in terms of molar ratio relative to p97-UN to simplify interpretation.
2. Fig. 2E: Please indicate in the figure or legend that WT and ΔUBX refer to FAF2.
3. Fig. 3C: There appears to be non-negligible recovery of p97-UN in control Strep pulldowns. It would be helpful to comment on background levels and provide quantification relative to controls.
4. Fig. EV4H: The asterisk should be defined in the legend.
5. Fig. 6B: It is unclear why binders B1-B20 are presented in two separate plots. Combining them or clarifying the rationale for separation would improve readability.
6. AlphaFold Methods: Please include UniProt accession numbers for all proteins used in structure prediction. Provide details on modeling parameters and confidence metrics, including pLDDT scores.
7. Please ensure that references to AlphaFold2 versus AlphaFold3 are consistent with the described modeling pipeline.

Referee #2:

In this study, Dasgupta and co-workers studied the role of FAF1 and FAF2 in the handling of ubiquitylated proteins by p97. They present some important conceptual findings on the difference between mammalian p97 and the yeast orthologue Cdc48 as well as the mechanism by which the FAF1 and FAF2 aid the UFD1/NPL4 adaptor in initiating the processing of ubiquitylated substrates by p97. This study builds further on the recent model that p97, unlike the proteasome, can unfold proteins starting from the proximal ubiquitin explaining why p97, unlike the proteasome, does not require an unstructured initiation site and why folded substrates require processing by p97 prior to degradation by the proteasome. They elegantly use the acquired knowledge in the design of small peptides that can stimulate protein unfolding by p97/UFD1/NPL4. The study is detailed and well performed and provides a compelling case for the proposed model. While this study, like any conceptual study, raises new questions that could be experimentally addressed, I feel that the data presented provides ample support for the proposed model and that further experimentation is not required.

Comments
FAF2 promotes faster unfolding than FAF1. FAF2 is normally tethered to membranes and involved in ERAD whereas FAF1 is soluble. Is it expected that the membrane-associated FAF2 is also more active than FAF1 and, if so, can the authors speculate if there could be a functional significance for this difference in activity? Could it be that extraction of ERAD substrates requires higher activity? Is it known if membrane tethered FAF1 can functionally substitute for FAF2 in ERAD?

The study is based on reporter substrates modified with K48-linked substrates. There are no data on endogenous substrates (which will be technically more challenging) but I find the data with the reporter substrates sufficiently convincing. Targeting with only K48-linked ubiquitin chains may, however, be less physiological or may at least not give the full picture as it has been shown in earlier studies that branched K48/K63-linked (by the same lab) and K27-linked ubiquitin chains (Mailand lab) target substrates to p97. The conclusions regarding the minimal length may only apply to K48-linked ubiquitin chains. Could the preference of human p97 for branched and atypical K27-linked chains (which are likely also to be branched) relate to higher demand by human p97 for ubiquitin interactions? It would be helpful to discuss these studies. Note also that the publication by the Mailand lab is not cited even though it is relevant for this study. The K48/K63 publication by the same group is only cited in the Methods section.

"Activation of p97 would be a desirable strategy to disassemble misfolded protein aggregates that are cytotoxic and a common feature of several proteinopathies." It should be noted though that mutant p97 that is linked to these proteinopathies has been shown to be hyperactive, not less active, which raises the question if one really would like to stimulate p97 in these diseases. I realize that the type of stimulation that the authors are referring to, namely enhance the initiation of unfolding, is different from

the increase in ATPase activity in mutant p97 observed in these diseases but I think that it would be helpful if the authors would discuss this in more detail.

Referee #3:

p97 and its cofactors are essential and evolutionarily conserved ATPases that regulate the disassembly of various cellular complexes from different cellular locations, thereby ensuring protein homeostasis and preventing proteinopathies. In recent years, many laboratories have shown increasing interest in studying the structure and cofactor organization of multiple p97 cofactors within the complex. The work described in this manuscript is carried out elegantly with well-controlled experiments. However, the manuscript lacks conceptual novelty, as similar models and activation mechanisms have unfortunately been recently described. For instance:

1. Fujisawa, R., Polo Rivera, C., & Labib, K. P. (2022). Multiple UBX proteins reduce the ubiquitin threshold of the mammalian p97-UFD1-NPL4 unfoldase. Elife, 11, e76763.

2. Huo, X. Y., Liu, D., Zou, R., Li, Z. P., Li, Y., Pan, L., ... & Zhang, Z. R. (2025). A UBH-UBX module amplifies p97/VCP's unfolding power to facilitate protein extraction and degradation. Nature Communications, 16(1), 10162.

3. Lee, H. G., Lemmon, A. A., & Lima, C. D. (2023). SUMO enhances unfolding of SUMO-polyubiquitin-modified substrates by the Ufd1/Npl4/Cdc48 complex. Proceedings of the National Academy of Sciences, 120(1), e2213703120.

4. Liao, Z., Arkinson, C., & Martin, A. (2025). Faf1 accelerates p97-mediated protein unfolding by promoting ubiquitin engagement. bioRxiv, 2025-10.

The two main strengths of this manuscript are: (1) identification of the AM region involved in the FAF2-UFD1 interaction; and (2) design of de novo p97 activators.

Point-by-point manuscript analysis:

Dasgupta et al. used Ub-Eos substrates bearing various levels of K48-linked ubiquitination to monitor p97-UN unfoldase activity and to screen a panel of UBA-UBX cofactors for their ability to further accelerate p97 activity. FAF2 was selected as the main focus of the study. The authors then identified the key interaction region (AM) within FAF2-CR that binds UFD1, using AF prediction, XL-MS, and HDX-MS, and validated this interaction through a series of point mutations. Finally, based on the AM, they designed several de novo proteins to demonstrate their potential utility in p97 activation.

Most findings in Figures 1 and 2, as well as about half of the data in Figure 5 (including the unfolding and ATPase assays), have already been reported (Fujisawa et al., 2022; Huo et al., 2025).

The authors invested considerable effort, using both XL-MS and HDX-MS, to characterise the FAF2-p97-UN complex, particularly the FAF2-UFD1 interaction, but they do not convincingly explain how "multivalent interactions" coordinate FAF2-dependent activation of p97 unfolding. This is especially true for the Ub-binding aspects, where some of the claims appear insufficiently supported and somewhat speculative.

For Figures 1 and 2, most of the conclusions overlap with what has already been reported in Fujisawa et al., 2022.

First, the authors used the Ub-Eos system to monitor p97 unfolding, which differs from Fujisawa et al., where disassembly of the CMG helicase was used as a readout for p97 unfolding. However, the resulting conclusions are largely the same:

• Line 157-159 (Figure 1D):
"we observed a significant enhancement of unfolding activity in the presence of p47, UBXN7, FAF1 and FAF2..."
• Line 161 (Figure EV1F, EV1G): "importantly, UN was indispensable..."
• Line 483-486 (Discussion): "Our finding that FAF1 and FAF2 act in concert with UFD1-NPL4 and accelerate the activity of human p97 supports this idea. Biochemical analysis of the FAF2-p97 complex revealed that FAF2 binding to p97 is highly dependent on the presence of UN..."
The similar results for UBXN7, FAF1, FAF2, and UN, which are indispensable for activation, have already been well documented in Fujisawa et al., 2022 (Figure 4D). It appears that the authors simply repeated similar experiments and reached the same conclusion. Although p47 emerged as a strong candidate for activating p97-UN in the current manuscript, the authors did not further investigate this finding.

The conclusions are similar or identical to those reported in Fujisawa et al.
• Line 231-233 (Figure 2H): "Collectively, FAF2 enhances p97-UN-mediated substrate unfolding and ATP hydrolysis once a threshold ubiquitin chain length of at least 5 K48-linked ubiquitins is reached."
• Line 505-507 (Discussion): "Hence, another important finding of our work is that FAF2 restores yeast-like efficiency to the human p97-UN complex and confers the same requirement of minimum five K48-linked ubiquitin..."
Fujisawa et al. have already specified a ubiquitin threshold of at least five K48-linked ubiquitin chains (Fujisawa et al., 2022; see their abstract/introduction and Figure 4E). I am concerned that this "important" finding did not originate from the current manuscript, especially given the claim that an "important finding of our work is that FAF2 restores yeast-like efficiency to the human p97-UN complex," whereas Fujisawa et al. already state that "association of p97-UFD1-NPL4 with the UBX proteins UBXN7, FAF1, or FAF2 reduces the ubiquitin threshold to a comparable level to yeast Cdc48-Ufd1-Npl4" (from the last paragraph of the introduction in Fujisawa et al., 2022).

The authors attempt to conclude the same ubiquitin threshold for p97 unfolding; however, Figure 2H includes only UbL- and Ub6-Eos as substrates, which is not sufficient to establish a precise cutoff of five K48-linked ubiquitin chains. The authors should additionally include UbVS- and Ub5-Eos for a proper comparison.

• Figure 5F:
The Ub-Eos system has been widely used in several publications (Lee et al., 2023; Huo et al., 2025). In particular, results for FAF2 (including some truncations), obtained using both the CMG disassembly assay (Fujisawa et al., 2022; see Figure 5G-I) and a similar Ub-Eos system (Huo et al., 2025; see Figure 5G and Supplementary Figure 6C-E), have already been reported. This manuscript uses Ub-Eos with various levels of K48 ubiquitination, which can be a distinguishing feature, but it does not alter the main conclusion that FAF2 lowers the Ub threshold, as previously shown by Fujisawa et al. (2022).

The main original contribution of this manuscript is that the authors further separate the CR and UBX domains from FAF2 for the p97 unfolding assay. This is a potentially valuable addition; however, a recent preprint reports similar findings (Liao et al., 2025; see Figure 4C-E). The authors should not omit this work from their discussion (Liao et al., 2025, doi: https://doi.org/10.1101/2025.10.27.684972), including a comparison with similar AF prediction models.
• Line 213-214 (Figure 2E): "the addition of FAF2 to p97 by itself or to p97-UN complex did not further increase ATP hydrolysis..." and

• Figure 5G:
Most of the results are similar to those reported in Huo et al., 2025 (see Figure 5A-F), where detailed ATP hydrolysis kinetics have already been characterized. Although the authors present new data for the ΔUBX and ΔCR constructs, they still need to properly discuss the findings from Huo et al., particularly because the data in the current manuscript lack kinetic analysis.

• Discrepancies to Huo et al., 2025:
The data regarding Ub binding in this manuscript are confusing and controversial in light of Huo et al., 2025, although the authors do address this issue. The characterization of FAF2's helical domain (HD, residues 275-349, also referred to as UBH) and its interaction with K48-linked Ub chains was thoroughly established in Huo et al., 2025. In that study, a series of detailed in vitro pull-down assays not only demonstrated direct binding between the HD and K48-linked Ub chains (with a minimal chain length of diUb), but also identified the L8-I44-V70 patch of Ub as the major binding surface. Huo et al. clearly showed that Ub binding by FAF2 is critical for enhancing p97-UN unfolding activity, a point that is not extensively discussed in the current manuscript. In addition, the "cooperative binding model" proposed here aims to emphasize that the FAF2 AM domain bridges the UFD1-Ub interaction, but the presented data are not sufficient to support this conclusion.
• Line 374-382:
The authors attempted to validate that R290 is essential for mediating Ub binding, as it contacts the K48 linkage in the AF model. However, mutation of R290 also abolishes the interaction of FAF2 with p97-UN in the absence of Ub. Therefore, the effect of R290 observed in the unfolding assay is not a direct readout of its specific role in Ub binding. This is also in contrast to the authors' later conclusion that FAF2-CR does not bind Ub. The authors need to properly dissect these two events: binding to Ub and binding to the p97-UN complex.
• Line 396-400:
The authors later conclude that FAF2 CR is not a UBD because FAF2-ΔUBA does not bind to Ub chains in the pull-down assay. This is in stark contrast to Huo et al., where the isolated CR was experimentally demonstrated to interact with Ub. The authors' claim that "These results support the structural model wherein the FAF2-CR by itself does not have high enough affinity for ubiquitin binding..." is therefore unsubstantiated and unconvincing. The authors specifically need to use the isolated CR for Ub-binding characterization before drawing such conclusions.
• Line 406-408: "Together, these results support a cooperative binding model in which the FAF2 activation motif enhances UFD1's effective affinity for K48-linked ubiquitin chains through multivalent interactions."
This conclusion again appears quite arbitrary. Although the authors used ITC to test that FAF2/UFD1 cannot bind K48-diUb, this is not sufficient to prove that "the FAF2 activation motif enhances UFD1's effective affinity for K48-linked ubiquitin chains." First, the ubiquitin chain length used in the ITC experiments (Figure EV4J and EV4K) differs from that in the pull-down assay in Figure EV4I, where FAF2 was shown to bind K48-Ub6. This discrepancy is confusing. Second, the authors do not provide any direct evidence that the FAF2 activation motif enhances UFD1's affinity for ubiquitin chains. The unfolding assay data so far only show that the ubiquitin threshold is reduced in the presence of FAF2, but they do not directly demonstrate a change in affinity for

ubiquitin. It is essential that the authors test whether FAF2 and UFD1 together display increased affinity for ubiquitin chains.
• Figure 2F:
The legend states, "in the presence or absence of UbL substrate." However, the data presented in the figure appear to be incomplete.
Minor comments:
• Abstract:
AM is shown for the first time, but without a full explanation of what is "AM"
• Line 248-250 (Figure 3B):
The view in Figure 3B is not sufficient to demonstrate that UBA and UAS do not engage with p97-UN; a more informative view is required. It would also be helpful to compare views in the presence and absence of ubiquitin.
• Figure 3F:
The chain type of the free Ub6-Ub8 chains in the legend is not clearly indicated.
• Figure 4:
The asterisks in some of the figures are shifted, especially 4G.
• Figure 5D:
• The orientation in the enlarged view appears to differ from that in Figure 5A.
Figure 5J:
• It's not clear if NPL4 is present in the reaction.

We thank the reviewers for carefully reading and critically reviewing our manuscript. We provide a point-by-point response to the reviewer's comments below.

Referee #1 (Report for Author)

The manuscript by Dasgupta et al. investigates how substrate unfolding by the p97-Ufd1-Npl4 (p97-UN) complex is activated by adaptors containing UBX domains. The study focuses on two related adaptors, FAF1 and FAF2. Through a combination of biochemical reconstitution, AlphaFold3 structural modeling, crosslinking mass spectrometry, HDX-MS, mutational analysis, and computational protein design, the authors propose a mechanism by which FAF1 and FAF2 potentiate substrate unfolding by p97-UN. The identification of an "activation motif" within FAF2, together with the derivation of de novo binders based on this region that recapitulate stimulation of p97-UN, is particularly elegant. Overall, the data are compelling, the experiments appear to be carefully executed, and the conclusions are largely supported by the data.
However, several aspects of the current study overlap with previously published work. Fujisawa et al. (eLife, 2022) showed that UBX adaptors including FAF1 and FAF2 reduce the ubiquitin threshold for mammalian p97-UFD1-NPL4 activity, while Huo et al. (Nat. Commun., 2025) described a UBH-UBX module in FAF family proteins that enhances p97-mediated unfolding. In addition, a recent study (Kracht et al., Sci. Adv., 2026) proposes a closely related mechanism in which a helix adjacent to the UBX domain engages the UT3 region of Ufd1 to promote substrate engagement and p97-mediated unfolding. In this context, the manuscript would benefit from more clearly positioning its findings relative to these studies and articulating which aspects of the mechanism represent new insight.
Below are suggestions aimed at clarifying mechanistic interpretation and improving presentation.

Major comments

1. Most experiments with FAF2 are performed with a construct lacking its membrane-targeting region. Given that endogenous FAF2 is ER-anchored, it would be appropriate to more explicitly discuss how removal of the membrane-binding region could influence effective concentration, spatial organization, and/or stoichiometry relative to p97-UN. While the truncation is entirely reasonable for in vitro reconstitution, the manuscript would benefit from acknowledging how membrane tethering might alter the complex in vivo, and to what extent the mechanistic conclusions are expected to extrapolate to the membrane-bound state.

We thank the reviewer for this comment. We agree with the reviewer that it is important to state that the membrane tethering can alter the complex in vivo and influence substrate selection, and we incorporated this point in the discussion of the revised manuscript.

2. The crosslinking MS and HDX-MS data appear broadly consistent with the AlphaFold3 model. However, it would strengthen the manuscript to more clearly articulate what unique mechanistic insights these approaches provide beyond structural validation. For example, do specific crosslinks discriminate between alternative structural arrangements? Do the data validate interfaces that are not confidently predicted by

AF3? Do ubiquitin-dependent changes in crosslinking or HDX patterns reveal conformational rearrangements that extend beyond the static model? A more explicit integration of these datasets with the proposed activation mechanism would clarify their added value.

In this manuscript we present the first AlphaFold predicted structure of the substrate engaged complex. We have primarily used the crosslinking MS and HDX-MS to validate the predicted structural model. The long length of some of the cross-links when mapped onto the AlphaFold model does suggest a certain amount of flexibility within the model and the existence of alternative structural arrangements. However, we have been cautious in our interpretation of the XL-MS data as we are not confident of making inferences or predictions based solely on this method that may later prove to be incorrect.

3. The FAF2 D304A mutant shows reduced binding to p97-UN, yet a detectable amount of p97 and Npl4 is still recovered in IPs (Fig. EV4G; Fig. 5H). Despite this residual association, the mutant behaves similarly to the "no FAF2" condition in the unfolding assay. This suggests that reduced binding alone may not fully explain the strong functional defect. The authors should clarify whether the degree of binding reduction is quantitatively sufficient to account for the loss of activity or consider the possibility that D304A selectively perturbs a conformational state required for activation rather than simply weakening bulk association.

The pulldown and unfolding assays operate under different experimental conditions, and direct quantitative comparison between the two readouts is therefore not straightforward. We have quantified the binding data for FAF2 mutants and reported in the revised manuscript. Upon quantification we observe recovery of only 15% UFD1 with FAF2 which we believe is the primary interaction driving initiator ubiquitin unfolding.

4. In Fig. EV4H, substantial recovery of FAF2 is observed in Ufd1 IPs for the R147E and Y162A mutants. These data do not appear to demonstrate strong disruption of the FAF2-Ufd1 interaction as stated (lines 372-373). If the interaction is only partially reduced, this should be quantified and described more cautiously. As currently presented, the data seem more consistent with partial impairment rather than a strong disruption.

We agree with this comment, and we modified (lines 370-372) accordingly.

5. The binder activation experiments are elegant and conceptually powerful. As a potentially high-impact extension, it would be informative to test whether the most potent binders can rescue unfolding defects in p97 loss-of-function mutants (e.g., D395G). Similarly, testing whether binder-mediated activation is lost or reduced in the Ufd1 UT3 mutants (R147E and Y162A) would help determine whether the designed binders operate through the same interface as endogenous FAF2.

We appreciate this elegant suggestion from the reviewer. D395G has been reported as a loss-of-function p97 mutation with defects in ATPase and unfolding activity. However, all unfolding assays in our study use p97 purified from insect cells due to catalytic inefficiency of bacterially expressed protein. Generating the D395G mutant in this system

would require cloning and expression in the baculoviral expression system, which is not feasible within the timeframe of this revision especially given the competing publications in the field. We consider this an important and exciting direction for future investigation. Regarding the suggestion to test binder-mediated activation in the UFD1 UT3 mutants R147E and Y162A, we thank the reviewer for this idea. We have performed this experiment and included the data. During revision we noticed that the UFD1 R147E mutant is less efficient in unfolding compared to the WT most likely due to the proximity or involvement of this residue in initiator ubiquitin binding. Conversely, UFD1 Y162A displayed enhanced basal unfolding activity in the absence of FAF2 (Figure EV5E). However, the Y162A mutation significantly impaired FAF2-mediated enhancement of p97 unfoldase activity, implicating Y162 as a critical residue at the FAF2–UFD1 interface. We have changed the interpretation of the results for these mutants accordingly in the revised manuscript. Importantly, Y162A equally impaired enhanced unfolding by the de novo binders B4 and B6, providing evidence that the designed activators operate through the same UFD1 interface as FAF2 (Figure 6E).

6. To fully establish necessity and sufficiency of the activating motif, it would strengthen the claim to test the isolated 286-312 region in a minimal format. Such an experiment would further define the minimal requirements for activation.

We thank the reviewer for this suggestion. However, the isolated 286–312 region of FAF2 alone would be insufficient to activate the p97-UN complex, as FAF2-mediated stimulation of p97 is lost when the UBX domain is deleted. Hence, FAF2 requires concurrent engagement with p97 via the UBX domain. We believe this is in large part due to the intrinsically disordered nature of this region, as evidenced by our HDX-MS data, which shows that it undergoes structural rearrangement upon binding to the p97-UFD1-NPL4 complex through the concerted action of both the UBX and activation motif regions. Notably, we enforced a helical structure for the 286–312 region in our activator design. We believe that this was important as it now bypasses the requirement for the UBX domain and the associated structural rearrangement through multivalent interactions with the p97-UFD1-NPL4 complex.

7. Please describe expression, purification, and quality control of the designed binders in the Methods.

Done

Minor Comments

1. Fig. 2C: Consider reporting the x-axis in terms of molar ratio relative to p97-UN to simplify interpretation.

Changed as suggested.

2. Fig. 2E: Please indicate in the figure or legend that WT and ΔUBX refer to FAF2.

Done

3. Fig. 3C: There appears to be non-negligible recovery of p97-UN in control Strep

pulldowns. It would be helpful to comment on background levels and provide quantification relative to controls.

Quantification is added.

4. Fig. EV4H: The asterisk should be defined in the legend.

Done

5. Fig. 6B: It is unclear why binders B1-B20 are presented in two separate plots. Combining them or clarifying the rationale for separation would improve readability.

The assays with 20 binders were performed across two separate experiments to accommodate the 15-second data collection interval, as the instrument could not support all samples simultaneously at this temporal resolution. Thus, we have represented the results in two separate plots with negative control (only UFD1-NPL4) and positive control (UFD1-NPL4 and FAF2) in both the assays for comparison.

6. AlphaFold Methods: Please include UniProt accession numbers for all proteins used in structure prediction. Provide details on modeling parameters and confidence metrics, including pLDDT scores.

Uniprot accession numbers of each protein are now provided in the Methods section. iPTM and PTM scores were added with each prediction alongside the PAE plots as a measure of the confidence of predicted interface and overall structural accuracy of the model.

7. Please ensure that references to AlphaFold2 versus AlphaFold3 are consistent with the described modelling pipeline.

Done

Referee #2 (Report for Author)

In this study, Dasgupta and co-workers studied the role of FAF1 and FAF2 in the handling of ubiquitylated proteins by p97. They present some important conceptual findings on the difference between mammalian p97 and the yeast orthologue Cdc48 as well as the mechanism by which the FAF1 and FAF2 aid the UFD1/NPL4 adaptor in initiating the processing of ubiquitylated substrates by p97. This study builds further on the recent model that p97, unlike the proteasome, can unfold proteins starting from the proximal ubiquitin explaining why p97, unlike the proteasome, does not require an unstructured initiation site and why folded substrates require processing by p97 prior to degradation by the proteasome. They elegantly use the acquired knowledge in the design of small peptides that can stimulate protein unfolding by p97/UFD1/NPL4. The study is detailed and well performed and provides a compelling case for the proposed model. While this study, like any conceptual study, raises new questions that could be experimentally addressed, I feel that the data presented provides ample support for the

proposed model and that further experimentation is not required.

We thank the reviewer for these suggestions to improve the manuscript. We included the suggested points in discussion of the revised manuscript.

Comments
FAF2 promotes faster unfolding than FAF1. FAF2 is normally tethered to membranes and involved in ERAD whereas FAF1 is soluble. Is it expected that the membrane-associated FAF2 is also more active than FAF1 and, if so, can the authors speculate if there could be a functional significance for this difference in activity? Could it be that extraction of ERAD substrates requires higher activity? Is it known if membrane tethered FAF1 can functionally substitute for FAF2 in ERAD?

We have included our view on the potential physiological relevance of differences in activity of FAF1 and FAF2 in our assay. While we do not have any evidence on whether membrane tethered FAF1 can functionally replace FAF2 for the task of membrane protein extraction, we think the conserved nature of the AM motif between FAF1 and FAF2 suggests that membrane tethered FAF1 would likely retain this activity and may therefore be capable of supporting membrane protein extraction in principle, though this remains to be tested experimentally.

The study is based on reporter substrates modified with K48-linked substrates. There are no data on endogenous substrates (which will be technically more challenging) but I find the data with the reporter substrates sufficiently convincing. Targeting with only K48-linked ubiquitin chains may, however, be less physiological or may at least not give the full picture as it has been shown in earlier studies that branched K48/K63-linked (by the same lab) and K27-linked ubiquitin chains (Mailand lab) target substrates to p97. The conclusions regarding the minimal length may only apply to K48-linked ubiquitin chains. Could the preference of human p97 for branched and atypical K27-linked chains (which are likely also to be branched) relate to higher demand by human p97 for ubiquitin interactions? It would be helpful to discuss these studies. Note also that the publication by the Mailand lab is not cited even though it is relevant for this study. The K48/K63 publication by the same group is only cited in the Methods section.

These are great suggestions to analyse the preference for branched chains. However, making these substrates is not trivial and will be the focus of future work. For the other points raised, we have now included these citations in the revised manuscript and stated that it will be important to understand the role of branched chain architectures in initiator unfolding process.

"Activation of p97 would be a desirable strategy to disassemble misfolded protein aggregates that are cytotoxic and a common feature of several proteinopathies." It should be noted though that mutant p97 that is linked to these proteinopathies has been shown to be hyperactive, not less active, which raises the question if one really would like to stimulate p97 in these diseases. I realize that the type of stimulation that the authors are referring to, namely enhance the initiation of unfolding, is different from the increase in ATPase activity in mutant p97 observed in these diseases but I think that it would be helpful if the authors would discuss this in more detail.

This is a very valid point, and we believe that the de novo activators designed here provide an advantage as they only work in a UFD1-dependent manner and would not indiscriminately activate p97 at all times. We have now added this to the Discussion section.

Referee #3 (Report for Author)

p97 and its cofactors are essential and evolutionarily conserved ATPases that regulate the disassembly of various cellular complexes from different cellular locations, thereby ensuring protein homeostasis and preventing proteinopathies. In recent years, many laboratories have shown increasing interest in studying the structure and cofactor organization of multiple p97 cofactors within the complex. The work described in this manuscript is carried out elegantly with well-controlled experiments. However, the manuscript lacks conceptual novelty, as similar models and activation mechanisms have unfortunately been recently described. For instance:

1. Fujisawa, R., Polo Rivera, C., & Labib, K. P. (2022). Multiple UBX proteins reduce the ubiquitin threshold of the mammalian p97-UFD1-NPL4 unfoldase. Elife, 11, e76763.

2. Huo, X. Y., Liu, D., Zou, R., Li, Z. P., Li, Y., Pan, L., ... & Zhang, Z. R. (2025). A UBH-UBX module amplifies p97/VCP's unfolding power to facilitate protein extraction and degradation. Nature Communications, 16(1), 10162.

3. Lee, H. G., Lemmon, A. A., & Lima, C. D. (2023). SUMO enhances unfolding of SUMO-polyubiquitin-modified substrates by the Ufd1/Npl4/Cdc48 complex. Proceedings of the National Academy of Sciences, 120(1), e2213703120.

4. Liao, Z., Arkinson, C., & Martin, A. (2025). Faf1 accelerates p97-mediated protein unfolding by promoting ubiquitin engagement. bioRxiv, 2025-10.

The two main strengths of this manuscript are: (1) identification of the AM region involved in the FAF2-UFD1 interaction; and (2) design of de novo p97 activators.

We agree with reviewer that Fujisawa et al., 2022 were the first to establish that UBX proteins (UBXN7, FAF1 and FAF2) reduce the ubiquitin threshold of mammalian p97-UFD1-NPL4, and we have cited and acknowledged this foundational contribution in our manuscript. However, their study was limited to a specialised chromatin-associated substrate, used an endpoint disassembly assay and did not resolve the molecular mechanism underlying cofactor-mediated stimulation. The importance of this mechanistic gap is underscored by the fact that multiple independent laboratories have recently sought to address it, including Huo et al. (Nat. Commun., 2025), Kracht et al. (Sci. Adv., 2026), Liao et al. (Cell Reports, 2026). Despite this substantial collective effort, the rules governing how the human p97-adaptor complex engages and processes ubiquitylated substrates still remain poorly understood at the mechanistic level. Our study addressed this gap by screening a panel of UBA-UBX cofactors across a range of defined ubiquitin chain lengths on a general reporter substrate, demonstrating that cofactor-dependent enhancement of p97-UN activity is not restricted to poorly

ubiquitylated substrates but represents a more fundamental mode of p97 regulation across diverse ubiquitination levels. Our quantitative kinetic assay allowed us to evaluate the most potent stimulator among the cofactors and led to identification of FAF2. Using AlphaFold prediction, XL-MS, HDX-MS, and mutational validation of both FAF2 and UFD1, we identify a conserved activation motif in FAF2 that directly contacts the UT3 domain of UFD1, providing the first structurally supported mechanistic explanation for FAF2 - mediated stimulation.

Leveraging this mechanistic framework, we further developed de novo p97 activators, a conceptual advance that none of the above studies describe. We acknowledge that despite our work using different techniques, and the competing manuscripts listed above, several questions remain to be fully resolved regarding FAF2 and other cofactor-driven stimulation of p97 activity. We consider these as important directions for future investigations. Therefore, we believe that our study represents a significant and distinct mechanistic advance that we hope will help guide and inform these future investigations.

Point-by-point manuscript analysis:

Dasgupta et al. used Ub-Eos substrates bearing various levels of K48-linked ubiquitination to monitor p97-UN unfoldase activity and to screen a panel of UBA-UBX cofactors for their ability to further accelerate p97 activity. FAF2 was selected as the main focus of the study. The authors then identified the key interaction region (AM) within FAF2-CR that binds UFD1, using AF prediction, XL-MS, and HDX-MS, and validated this interaction through a series of point mutations. Finally, based on the AM, they designed several de novo proteins to demonstrate their potential utility in p97 activation.

Most findings in Figures 1 and 2, as well as about half of the data in Figure 5 (including the unfolding and ATPase assays), have already been reported (Fujisawa et al., 2022; Huo et al., 2025).

We respectfully disagree with the reviewer and clarify how our work relates to these cited publications.

Fig 1 A and B: Schematic

Fig 1C: This panel shows that even at long chain lengths, well above the threshold for both human and yeast systems, human p97-UN still unfolds substrates markedly more slowly than its yeast counterpart. This kinetic gap has not been demonstrated in Fujisawa et al., 2022 or Huo et al., 2025.

Fig 1D and E: We screened the same cofactors as Fujisawa et al. and p47 additionally but in a different context. The results demonstrate that cofactor-dependent stimulation of p97-UN activity persists even on long ubiquitin chain-bearing substrates, a finding not reported by Fujisawa et al. We acknowledge that Huo et al. have shown it, but only for FAF1 and FAF2. Intriguingly, we find that p47 activates unfolding of long ubiquitin chain containing substrates in a UFD1-NPL4 dependent manner.

Fig 1F and G: While consistent with Fujisawa et al., these results provide a quantitative ranking of cofactor activity, revealing FAF1 and FAF2 as the strongest stimulators and

providing the rationale for selecting FAF2 as the primary focus of subsequent mechanistic investigation.

Figure 2A and 2B: AlphaFold model of FAF1 and FAF2

Figure 2C and 2D: Provides careful analysis of maximal initial rate and EC50 of FAF1 and FAF2 to identify FAF2 as the most potent stimulator that was not shown in any of the listed studies.

Figure 2E and 2F: We acknowledge that these results were also reported by Huo et al. 2025 as the results are similar in our end point assay and the kinetic assay performed by Huo et al. 2025.

Figure 2G and 2H: In 2G, we perform the ATPase assay in presence of Ub chain of defined length to provide a quantitative readout of chain length threshold of enhanced unfolding and consequent increase in substrate stimulated ATPase activity. In 2H, we compare the potency of FAF2 in unfolding long vs short (Ub_6) chain. These are unique results and we don't find the same or similar data elsewhere.

about half of the data in Figure 5 (including the unfolding and ATPase assays)**:** In Figure 5F and 5G we provide kinetics of unfolding and ATPase activity for ΔUBA, ΔUBX and ΔCR constructs of FAF2. We acknowledge that Huo et al., 2025 show data for ΔUBA. Figures 5H, 5I, and 5J present unfolding activity data for FAF2 activation motif point mutants and reciprocal UFD1 UT3 mutations. Except for one overlapping residue (Y297), none of the point mutations in our study correspond to those reported by Huo et al. We wish to highlight the mechanistic difference in the rationale for the mutational studies performed in our manuscript and Huo et al. Huo et al. used AlphaFold to predict two Ub binding sites on CR – UBS1 (L289, R290) and UBS2 (Y297, L301) and concluded that it interacts with L8-I44-V70 patch of ubiquitin which were performed in presence of CR and three ubiquitins. Notably, however, Huo et al. do not address whether this three-ubiquitin binding mode is compatible with a K48-linked conformation. Huo et al. subsequently mutated L289, Y297, and L301 to disrupt ubiquitin binding and assessed the functional consequences in unfolding and ATPase assays.
In contrast, the mutational analysis in our manuscript was performed to disrupt the FAF2-CR/UFD1 UT3 contact, an interface identified through several orthogonal approaches such as AlphaFold prediction of the unfoldase complex, HDX-MS and crosslinking-MS. Importantly, our AlphaFold model of the complex identifies D294, Y297, S300, D304, K307, and E308 of FAF2-CR as contacting the UT3 domain of UFD1, not ubiquitin. Furthermore, our model identifies R290 as contacting the Asp58 patch of the proximal Ub and K48 linkage between proximal and initiator ubiquitin exclusively in the context of the substrate-engaged complex. Hence, we provide a fundamentally distinct mechanistic rationale than that proposed by Huo et al. Importantly, we provide data on reciprocal mutations on UFD1 to support our model. The two mutational studies, therefore, address entirely different mechanistic hypotheses, and our data provide a compelling interpretation of a surface in the context of substrate engagement and initiator unfolding by p97-UN complex, which Huo et al. characterise solely as a ubiquitin-binding interface.

The authors invested considerable effort, using both XL-MS and HDX-MS, to characterise the FAF2-p97-UN complex, particularly the FAF2-UFD1 interaction, but they do not convincingly explain how "multivalent interactions" coordinate FAF2-dependent activation of p97 unfolding. This is especially true for the Ub-binding aspects, where some of the claims appear insufficiently supported and somewhat speculative.
See below

For Figures 1 and 2, most of the conclusions overlap with what has already been reported in Fujisawa et al., 2022.

This is a repetition of the previous comment which we have addressed in detail above.

First, the authors used the Ub-Eos system to monitor p97 unfolding, which differs from Fujisawa et al., where disassembly of the CMG helicase was used as a readout for p97 unfolding. However, the resulting conclusions are largely the same:

• Line 157-159 (Figure 1D):
"we observed a significant enhancement of unfolding activity in the presence of p47, UBXN7, FAF1 and FAF2..."

We wish to clarify that the enhancement of p97-UN unfolding activity by p47, UBXN7, FAF1, and FAF2 reported in Figure 1D is observed using long ubiquitin chain-modified Ub-Eos substrate, an observation that was not reported by Fujisawa et al.

• Line 161 (Figure EV1F, EV1G): "importantly, UN was indispensable..."

The indispensability of UN shown in Figures EV1F and EV1G represents an important experimental control rather than a principal conclusion.

• Line 483-486 (Discussion): "Our finding that FAF1 and FAF2 act in concert with UFD1-NPL4 and accelerate the activity of human p97 supports this idea. Biochemical analysis of the FAF2-p97 complex revealed that FAF2 binding to p97 is highly dependent on the presence of UN..."

We revised the lines 524–531 to clearly attribute our distinct finding that FAF1 and FAF2 stimulate p97-UN activity not only on short ubiquitin chains but across a broad range of chain lengths on a general substrate, extending their role beyond lowering threshold.

The similar results for UBXN7, FAF1, FAF2, and UN, which are indispensable for activation, have already been well documented in Fujisawa et al., 2022 (Figure 4D). It appears that the authors simply repeated similar experiments and reached the same conclusion. Although p47 emerged as a strong candidate for activating p97-UN in the current manuscript, the authors did not further investigate this finding.

We explicitly mentioned the differences between our study and Fujisawa et al. in our responses above, and we refer the reviewer to those points. We agree that p47 is an interesting and unexpected finding that warrants further investigation. However, a

thorough mechanistic characterisation of p47-mediated stimulation falls outside the scope of the current manuscript.

The conclusions are similar or identical to those reported in Fujisawa et al.
• Line 231-233 (Figure 2H): "Collectively, FAF2 enhances p97-UN-mediated substrate unfolding and ATP hydrolysis once a threshold ubiquitin chain length of at least 5 K48-linked ubiquitins is reached."

• Line 505-507 (Discussion): "Hence, another important finding of our work is that FAF2 restores yeast-like efficiency to the human p97-UN complex and confers the same requirement of minimum five K48-linked ubiquitin..."

Fujisawa et al. have already specified a ubiquitin threshold of at least five K48-linked ubiquitin chains (Fujisawa et al., 2022; see their abstract/introduction and Figure 4E). I am concerned that this "important" finding did not originate from the current manuscript, especially given the claim that an "important finding of our work is that FAF2 restores yeast-like efficiency to the human p97-UN complex," whereas Fujisawa et al. already state that "association of p97-UFD1-NPL4 with the UBX proteins UBXN7, FAF1, or FAF2 reduces the ubiquitin threshold to a comparable level to yeast Cdc48-Ufd1-Npl4" (from the last paragraph of the introduction in Fujisawa et al., 2022).

The authors attempt to conclude the same ubiquitin threshold for p97 unfolding; however, Figure 2H includes only Ub_L- and Ub_6-Eos as substrates, which is not sufficient to establish a precise cutoff of five K48-linked ubiquitin chains. The authors should additionally include Ub_{VS}- and Ub_5-Eos for a proper comparison.

We acknowledge that the ubiquitin threshold of at least five K48-linked ubiquitin and the restoration of yeast-like efficiency to human p97-UN by UBX cofactors were first established by Fujisawa et al. We revised lines 232-235 and 569–574 to clearly attribute priority to Fujisawa et al. and apologise if the phrasing in the current manuscript created the impression that these observations originated from our work.
However, we draw the reviewer's attention to Figure 2G, which provides the quantitative basis for this conclusion in our system. While Fujisawa et al. establish the threshold using an endpoint disassembly assay with enzymatically generated heterogeneous ubiquitin chains and for a mixture of all three UBX proteins together (their Figure 4E), our conclusion is derived from a quantitative ATPase activity assay using defined discrete K48-linked chain lengths with FAF2 alone. We used the ATPase assay as a proxy for unfolding activity, justified by the strong correlation between ATPase and unfolding rates demonstrated in our system. We agree that a direct comparison of unfolding rates using Ub_4-, Ub_5- and Ub_6-Eos substrates would provide more convincing proof of the threshold. However, the synthesis of Ub-Eos substrates with precisely defined chain length is technically demanding and time-consuming, and generating additional chain length variants on Eos within the timeframe of this revision is not feasible.

• Figure 5F:
The Ub-Eos system has been widely used in several publications (Lee et al., 2023; Huo et al., 2025). In particular, results for FAF2 (including some truncations), obtained using both the CMG disassembly assay (Fujisawa et al., 2022; see Figure 5G-I) and a similar Ub-Eos system (Huo et al., 2025; see Figure 5G and Supplementary Figure 6C-E), have

already been reported. This manuscript uses Ub-Eos with various levels of K48 ubiquitination, which can be a distinguishing feature, but it does not alter the main conclusion that FAF2 lowers the Ub threshold, as previously shown by Fujisawa et al. (2022).

We have already addressed the comment regarding Figure 5 in detail above. We would like to emphasise that demonstrating FAF2 lowers the ubiquitin threshold is not the main conclusion of Figure 5F or our manuscript. We have revised the text to make this clear.

The main original contribution of this manuscript is that the authors further separate the CR and UBX domains from FAF2 for the p97 unfolding assay. This is a potentially valuable addition; however, a recent preprint reports similar findings (Liao et al., 2025; see Figure 4C-E). The authors should not omit this work from their discussion (Liao et al., 2025, doi: https://doi.org/10.1101/2025.10.27.684972), including a comparison with similar AF prediction models.

We thank the reviewer for drawing our attention to Liao et al. (bioRxiv, 2025). We agree that their study reports complementary finding, specifically that the CR-UBX of FAF2 is sufficient to stimulate p97-UN mediated substrate unfolding, consistent with our model. However, we note that Liao et al. focus primarily on FAF1, with FAF2 characterized only by truncation analysis in Figure 4C–E and do not provide analysis of an AlphaFold model including ubiquitin. Our study provides the mechanistic resolution specifically for FAF2 and validates the interface through reciprocal mutations on both FAF2 and UFD1, provides direct evidence for the interaction by HDX-MS. Additionally, our study extends beyond mechanistic characterization through the design of de novo p97 activators.

We have included this citation in the revised manuscript.

• Line 213-214 (Figure 2E): "the addition of FAF2 to p97 by itself or to p97-UN complex did not further increase ATP hydrolysis..." and

• Figure 5G:
Most of the results are similar to those reported in Huo et al., 2025 (see Figure 5A-F), where detailed ATP hydrolysis kinetics have already been characterized. Although the authors present new data for the ΔUBX and ΔCR constructs, they still need to properly discuss the findings from Huo et al., particularly because the data in the current manuscript lack kinetic analysis.

We note that Huo et al.'s ATPase assay data reveals changes in the kinetic parameters of ATP hydrolysis exclusively in the presence of ubiquitylated substrate and are absent under basal conditions, consistent with our endpoint ATPase activity measurements in Figure 2E and 2F. The absence of any CR-UBX -dependent change in basal ATPase activity indicates that FAF2 does not fundamentally alter p97's intrinsic motor capacity (as concluded in Line 215-216), and that the kinetic changes Huo et al. observe are strictly substrate-dependent, and therefore mechanistically consistent with our model, in which FAF2 stabilises the substrate-engaged p97-UFD1-NPL4 complex through the CR-UFD1 interface, leading to more efficient substrate processing as a downstream consequence. We have included this point now.

• Discrepancies to Huo et al., 2025: The data regarding Ub binding in this manuscript are confusing and controversial in light of Huo et al., 2025, although the authors do address this issue. The characterization of FAF2's helical domain (HD, residues 275-349, also referred to as UBH) and its interaction with K48-linked Ub chains was thoroughly established in Huo et al., 2025. In that study, a series of detailed in vitro pull-down assays not only demonstrated direct binding between the HD and K48-linked Ub chains (with a minimal chain length of diUb) but also identified the L8-I44-V70 patch of Ub as the major binding surface. Huo et al. clearly showed that Ub binding by FAF2 is critical for enhancing p97-UN unfolding activity, a point that is not extensively discussed in the current manuscript. In addition, the "cooperative binding model" proposed here aims to emphasize that the FAF2 AM domain bridges the UFD1-Ub interaction, but the presented data are not sufficient to support this conclusion.

We acknowledge that the relationship between our findings and those of Huo et al. regarding ubiquitin binding by the FAF2 helical domain requires careful clarification. We wish to emphasise that our data do not contradict Huo et al. but rather provide a mechanistically distinct perspective. Huo et al. identify a low-affinity ubiquitin-binding activity of the isolated CR and propose this as a substrate recruitment mechanism, while acknowledging that this weak interaction alone is insufficient to explain the full stimulatory effect provided by FAF2. Our manuscript addresses this gap by identifying a more stable CR-UT3 interaction. We address the specific points raised below.

• Line 374-382:
The authors attempted to validate that R290 is essential for mediating Ub binding, as it contacts the K48 linkage in the AF model. However, mutation of R290 also abolishes the interaction of FAF2 with p97-UN in the absence of Ub. Therefore, the effect of R290 observed in the unfolding assay is not a direct readout of its specific role in Ub binding. This is also in contrast to the authors' later conclusion that FAF2-CR does not bind Ub. The authors need to properly dissect these two events: binding to Ub and binding to the p97-UN complex.

We thank the reviewer for this careful observation and agree that the R290 mutation has a complex phenotype. We attempted to address this in Line 376-379. "This finding raises the possibility that R290 in FAF2 adopts a slightly different conformation upon ubiquitin binding, enabling simultaneous recognition of UFD1 and ubiquitin". The AlphaFold model suggests R290 may simultaneously contact both UFD1 and the K48 linkage, which would inherently preclude dissecting the two binding events through mutagenesis alone. As no experimental structure of this region in FAF2 is currently available, a precise dissection of these two events is not feasible currently, and we consider it as an important question for future investigation.

We also wish to clarify that we do not conclude that the CR has no contact with ubiquitin. We rather suggested that FAF2 CR contacts proximal Ub and distal ubiquitin exclusively in the context of initiator Ub engaged FAF2-p97-UFD1-NPL4 complex, when the UT3 domain simultaneously engage with the proximal and distal Ub and FAF2 AM.

• Line 396-400:
The authors later conclude that FAF2 CR is not a UBD because FAF2-ΔUBA does not

bind to Ub chains in the pull-down assay. This is in stark contrast to Huo et al., where the isolated CR was experimentally demonstrated to interact with Ub. The authors' claim that "These results support the structural model wherein the FAF2-CR by itself does not have high enough affinity for ubiquitin binding..." is therefore unsubstantiated and unconvincing. The authors specifically need to use the isolated CR for Ub-binding characterization before drawing such conclusions.

We would like to point out that Huo et al. themselves state that "UBH binds ubiquitin chains with surprisingly low affinity, as only a small fraction of chains were recovered even with excess UBH. This weak binding alone cannot explain the strong enhancement of ubiquitin targeting to p97-UN, suggesting additional mechanisms," and acknowledge that "how UBH engages tri-ubiquitin or longer chains within the p97-UNF complex remains unresolved." Our identification of the FAF2-CR/UFD1 UT3 interface provides precisely the kind of additional mechanism that Huo et al. mention, and we consider the two studies complementary in this regard.
Importantly, we identify the same surface of FAF2 proposed by Huo et al. suggest as ubiquitin interacting to instead interact primarily with the UFD1 UT3 domain. This important distinction of our study is supported by multiple orthogonal approaches including AlphaFold, XL-MS, HDX-MS, and mutational analysis. Therefore, we propose that the CR-UFD1 UT3 interaction, rather than direct CR-ubiquitin binding, is the primary mechanistic basis for FAF2-mediated stimulation of p97 activity, while acknowledging that the precise contribution of CR-ubiquitin engagement in the catalytic cycle remains an important question for future investigation.

• Line 406-408: "Together, these results support a cooperative binding model in which the FAF2 activation motif enhances UFD1's effective affinity for K48-linked ubiquitin chains through multivalent interactions." This conclusion again appears quite arbitrary. Although the authors used ITC to test that FAF2/UFD1 cannot bind K48-diUb, this is not sufficient to prove that "the FAF2 activation motif enhances UFD1's effective affinity for K48-linked ubiquitin chains." First, the ubiquitin chain length used in the ITC experiments (Figure EV4J and EV4K) differs from that in the pull-down assay in Figure EV4I, where FAF2 was shown to bind K48-Ub6. This discrepancy is confusing. Second, the authors do not provide any direct evidence that the FAF2 activation motif enhances UFD1's affinity for ubiquitin chains. The unfolding assay data so far only show that the ubiquitin threshold is reduced in the presence of FAF2, but they do not directly demonstrate a change in affinity for ubiquitin. It is essential that the authors test whether FAF2 and UFD1 together display increased affinity for ubiquitin chains.

We acknowledge the reviewer's criticism on this point. We recognise that the chain lengths used in the ITC experiments (Figures EV4J and EV4K using Ub_2) are inconsistent with those used in the pulldown assay (Figure EV4I using Ub_3 and Ub_6), and we apologise for this confusion. We have performed MST with K48-Ub_3 (that is proposed as a threshold for Ub binding by FAF2 CR in Huo et al. 2025) and FP based binding assay with K48-Ub_5. We detected binding with longer chains using both techniques. We have reported this in the revised manuscript.

We agree that our current experimental data do not provide direct evidence that the FAF2 activation motif enhances UFD1's affinity for ubiquitin chains, although our AlphaFold model of the complex engaged with ubiquitin chain suggests such cooperative mode of

binding. To prove this model, we tested whether FAF2 and UFD1 together display increased affinity of UFD1 for ubiquitin chains in the absence of p97. However, we were unable to detect any Ub binding by UFD1 and FAF2 in pulldown assays under these conditions, suggesting that the cooperative interaction may require the fully assembled substrate engaged p97-UFD1-NPL4-FAF2 complex.

We draw the reviewer's attention to a recent preprint dissecting the role of yeast Ufd1 in ATP independent initiator ubiquitin unfolding, which demonstrate yeast Ufd1 alone is capable of unfolding initiator ubiquitin upon binding to K48 linked di-ubiquitin (Wang *et al*, 2026). Strikingly, the proposed mode of binding of K48 linked di-ubiquitin to yeast Ufd1 UT3 domain based on the crystal structure is highly similar to our AlphaFold3 predicted model of cooperative di ubiquitin binding by human UFD1 UT3 and FAF2 CR. To test whether human UFD1 retains this unfolding activity, we employed a similar dye accessibility assay and find that human UFD1 alone is inefficient in this activity and presence of FAF2 stimulates initiator unfolding by UFD1. Furthermore, we show that the function is preserved in de novo binders incorporating critical residues and confirmation of FAF2 CR. We believe this constitute direct functional evidence for cooperative ubiquitin engagement by UFD1 and FAF2, strongly supporting our proposed model. These new data is included in the revised manuscript. We agree with the reviewer that a direct binding assay would have further strengthened this argument. However, we note that direct binding measurements are not feasible in this context, as the unfolded initiator ubiquitin rapidly refolds in the absence of NPL4, precluding stable complex formation for biochemical characterization. Thus, we were unable to reliably capture or measure such an interaction directly. We therefore consider the ubiquitin unfolding assay to be the most direct measure of this cooperative mechanism.

• Figure 2F:
The legend states, "in the presence or absence of UbL substrate." However, the data presented in the figure appear to be incomplete.

This was an error in the figure legend. The experiment was performed in the presence of the Ub^{L} substrate. We have corrected the legend accordingly in the revised manuscript.

Minor comments:
• Abstract:
AM is shown for the first time, but without a full explanation of what is "AM"

Fixed

• Line 248-250 (Figure 3B):
The view in Figure 3B is not sufficient to demonstrate that UBA and UAS do not engage with p97-UN; a more informative view is required. It would also be helpful to compare views in the presence and absence of ubiquitin.

The CR-UT3 interaction is largely unaltered in AlphaFold predicted model in presence of ubiquitin. This has now been added to the revised manuscript.

• Figure 3F:
The chain type of the free Ub6-Ub8 chains in the legend is not clearly indicated.

Done

• Figure 4:
The asterisks in some of the figures are shifted, especially 4G.
Corrected

• Figure 5D:
• The orientation in the enlarged view appears to differ from that in Figure 5A.
Fixed

Figure 5J:
• It's not clear if NPL4 is present in the reaction.
Included NPL4 in the legend.

Prof. Yogesh Kulathu
University of Dundee
MRC Protein Phosphorylation and Ubiquitylation Unit
Dow Street
Dundee DD1 5EH
United Kingdom

15th Jul 2026

Re: EMBOJ-2026-123779R
FAF1 and FAF2 enhance unfolding by p97-UFD1-NPL4 complex enabling rational design of p97 activators

Dear Yogesh,

Thank you for submitting your revised manuscript on FAF1/2 and p97 activators for our consideration. It has now been re-reviewed by two of the original referees (comments copied below), who both found their concerns well-addressed and the study now ready for publication. At this stage, there are only a few editorial issues that should be taken care of at this stage:

- Our pre-acceptance text similarity checks found that certain passages of Material & Methods section passages appear to be near-verbatim copies from a previous paper by Kanta, Masson et al, which is however not cited. While this is acceptable in principle in the interest of method reproducibility, please however make sure to cite the earlier work on those occasions ('...was done essentially as in ... Briefly, ...').

- Please adjust the order of the manuscript sections, and also make sure to use the correct section headers:
Single title page with complete (full-name!) author information, Abstract, Introduction, Results, Discussion, Methods, Data Availability, Acknowledgements, Disclosure and Competing Interests Statement, References, Main Figure Legends, Tables, Expanded Figure Legends.

- Please move all funding information into the Acknowledgements section, ande double-check to make sure to all relevant funding information in the manuscript is congruent with the info entered into our submission system. Currently missing in the submission system seems to be: Prize Studentship from the Tricia Cohen Memorial Trust

- Please adjust the format for citation of preprints as specified in our author guidelines:
The citation in the text should be: "(preprint: NAME1 et al, YEAR)"
The citation in the reference list: "Author NAME1, Author NAME2, ... (YEAR) article title. bioRxiv doi: XXX"

- Please remove the Reagents and Tools table from the manuscript text, and upload it separately (making sure to adhere to the table template downloadable from our Guide to Authors).

- As we are switching from a free-text author contribution statement towards a more formal statement based on Contributor Role Taxonomy (CRediT) terms, please remove the present Author Contribution section and instead specify each author's contribution(s) directly in the Author Information page of our submission system during upload of the final manuscript. See https://casrai.org/credit/ for more information.

- Please check that each data item is referenced at least once in the main text - we note that callouts to Appendix Table S1 are currently missing.

- Please make sure to preface the Appendix file with a title page stating 'Appendix for [paper title]' and a table of content listing individual items together with respective page numbers. Make sure to label the items 'Appendix Figure S1/2/...' and 'Appendix Table S1/2' rather than 'supplementary...', both within the Appendix and when referring to them in the text. Finally, I am not sure about the significance of the last four items ('coverage', 'plots'), which also do not appear to be referenced anywhere in the manuscript. I think they should either become additional Appendix Figures and referenced as such at least once each; or possibly constitute Source Data, and be uploaded as such?

- Please provide the exact p values in the legends of figures 1E, G; 2G, 4B, D, F, G.

- In the Data Availability section, please include URL(s) for the databases (PRIDE?) in which the listed datasets have been deposited. Reviewer access information should be removed, and public release initiated at this point.

- Finally, please provide suggestions for a short 'blurb' text prefacing and summing up the conceptual aspect of the study in two sentences (max. 250 characters), followed by 3-5 one-sentence 'bullet points' with brief factual statements of key results of the

paper; they will form the basis of an editor-written 'Synopsis' accompanying the online version of the article. Please also upload a synopsis image, which can be used as a "visual title" for the synopsis section of your paper. The image should be ideally in JPG format, and please make sure that it remains in the modest dimensions of (exactly) 550 pixels wide and 300 pixels high.

I am returning the manuscript to you for a final round of minor revision, solely to allow you to make these modifications and upload the revised files. Once we will have received them, we could swiftly proceed with formal acceptance and production of the manuscript.

With kind regards,

Hartmut

Hartmut Vodermaier, PhD
Senior Editor, The EMBO Journal
h.vodermaier@embojournal.org

*** PLEASE NOTE: All revised manuscript are subject to initial checks for completeness and adherence to our formatting guidelines. Revisions may be returned to the authors and delayed in their editorial re-evaluation if they fail to comply to the following requirements. As a first step please read our guidelines for revised submissions:
https://link.springer.com/journal/44318/submission-guidelines#cms-Revised-submissions

1) Every manuscript requires a Data Availability section (even if only stating that no deposited datasets are included). Primary datasets or computer code produced in the current study have to be deposited in appropriate public repositories prior to resubmission, and reviewer access details provided in case that public access is not yet allowed.

2) Each figure legend must specify
- size of the scale bars that are mandatory for all micrograph panels
- the statistical test used to generate error bars and P-values
- the type error bars (e.g., S.E.M., S.D.)
- the number (n) and nature (biological or technical replicate) of independent experiments underlying each data point
- Figures may not include error bars for experiments with n<3; scatter plots showing individual data points should be used instead.

3) Revised manuscript text (including main tables, and figure legends for main and EV figures) has to be submitted as editable text file (e.g., .docx format). We encourage highlighting of changes (e.g., via text color) for the referees' reference.

4) Each main and each Expanded View (EV) figure should be uploaded as individual production-quality files (preferably in .eps, .tif, .jpg formats). For suggestions on figure preparation/layout, please refer to our Figure Preparation Guidelines:
https://media.springernature.com/original/springer-cms/rest/v1/content/27825798/data/v1

5) Point-by-point response letters should include the original referee comments in full together with your detailed responses to them (and to specific editor requests if applicable), and also be uploaded as editable (e.g., .docx) text files.

6) Please complete our Author Checklist, and make sure that information entered into the checklist is also reflected in the manuscript; the checklist will be available to readers as part of the Review Process File.

7) All authors listed as (co-)corresponding need to deposit, in their respective author profiles in our submission system, a unique ORCiD identifier linked to their name. Please see our Guide to Authors for detailed instructions.

8) Please note that supplementary information at EMBO Press has been superseded by the 'Expanded View' for inclusion of additional figures, tables, movies or datasets; with up to five EV Figures being typeset and directly accessible in the HTML version of the article.

9) To facilitate reproducibilty and cross-laboratory adoption of methodologies, please structure the Materials & Methods section as outlined in our guide to authors, including a completed Reagents and Tools Table.

10) Digital image enhancement is acceptable practice, as long as it accurately represents the original data and conforms to community standards. If a figure has been subjected to significant electronic manipulation, this must be clearly noted in the figure legend and/or the 'Materials and Methods' section. The editors reserve the right to request original versions of figures and the original images that were used to assemble the figure. Finally, we generally encourage uploading of numerical as well as gel/blot image source data.

At EMBO Press we ask authors to provide source data for the main manuscript figures. You will receive a separate email with instructions for providing source data with your revised manuscript, including how to upload and organize the files.

In the interest of ensuring the conceptual advance provided by the work, we recommend submitting a revision within 3 months (13th Oct 2026). Please discuss the revision progress ahead of this time with the editor if you require more time to complete the revisions. Use the link below to submit your revision:

Link Not Available

Referee #1:

I appreciate the authors' thoughtful responses and substantial revisions. In particular, the new experiments examining the designed binders with UFD1 interface mutants strengthen the mechanistic conclusions and address one of my main concerns. The Discussion has also been appropriately expanded to acknowledge limitations and to better place the work in the context of recent studies. While I still think some mechanistic interpretations (particularly those based on predicted structural modeling and XL-MS/HDX-MS) could be phrased somewhat more cautiously, these are matters of emphasis rather than substantive flaws. Overall, I believe the authors have adequately addressed my concerns and I support publication.

Referee #2:

The authors have addressed my concerns.

All minor editorial requests have been addressed by the authors

EMBO Press Author Checklist

Corresponding Author Name: Yogesh Kulathu	
Journal Submitted to: The EMBO Journal	
Manuscript Number: EMBOJ-2026-123779R	

USEFUL LINKS FOR COMPLETING THIS FORM
[The EMBO Journal - Author Guidelines]
[EMBO Reports - Author Guidelines]
[Molecular Systems Biology - Author Guidelines]
[EMBO Molecular Medicine - Author Guidelines]

Reporting Checklist for Life Science Articles (updated January
This checklist is adapted from Materials Design Analysis Reporting (MDAR) Checklist for Authors. MDAR establishes a minimum set of requirements in transparent reporting in the life sciences (see Statement of Task: 10.31222/osf.io/9sm4x). Please follow the journal's guidelines in preparing your
Please note that a copy of this checklist will be published alongside your article.

Abridged guidelines for figures
1. Data
The data shown in figures should satisfy the following conditions:
- the data were obtained and processed according to the field's best practice and are presented to reflect the results of the experiments in an accurate and unbiased manner.
- ideally, figure panels should include only measurements that are directly comparable to each other and obtained with the same assay.
- plots include clearly labeled error bars for independent experiments and sample sizes. Unless justified, error bars should not be shown for technical
- if n<5, the individual data points from each experiment should be plotted. Any statistical test employed should be justified.
- Source Data should be included to report the data underlying figures according to the guidelines set out in the authorship guidelines on Data

2. Captions
Each figure caption should contain the following information, for each panel where they are relevant:
- a specification of the experimental system investigated (eg cell line, species name).
- the assay(s) and method(s) used to carry out the reported observations and measurements.
- an explicit mention of the biological and chemical entity(ies) that are being measured.
- an explicit mention of the biological and chemical entity(ies) that are altered/varied/perturbed in a controlled manner.
- the exact sample size (n) for each experimental group/condition, given as a number, not a range;
- a description of the sample collection allowing the reader to understand whether the samples represent technical or biological replicates (including how many animals, litters, cultures, etc.).
- a statement of how many times the experiment shown was independently replicated in the laboratory.
- definitions of statistical methods and measures:
  - common tests, such as t-test (please specify whether paired vs. unpaired), simple $\chi2$ tests, Wilcoxon and Mann-Whitney tests, can be unambiguously identified by name only, but more complex techniques should be described in the methods section;
  - are tests one-sided or two-sided?
  - are there adjustments for multiple comparisons?
  - exact statistical test results, e.g., P values = x but not P values < x;
  - definition of 'center values' as median or average;
  - definition of error bars as s.d. or s.e.m.

Please complete ALL of the questions below.
Select "Not Applicable" only when the requested information is not relevant for your study.

Materials

Newly Created Materials	Information included in the manuscript?	In which section is the information available? (Reagents and Tools Table, Materials and Methods, Figures, Data Availability Section)
New materials and reagents need to be available; do any restrictions apply?	Not Applicable	Reagents and Tools Table, Materials and Methods; No restrictions

Antibodies	Information included in the manuscript?	In which section is the information available? (Reagents and Tools Table, Materials and Methods, Figures, Data Availability Section)
For **antibodies** provide the following information:<br>- Commercial antibodies: RRID (if possible) or supplier name, catalogue number and or/clone number<br>- Non-commercial: RRID or citation	Not Applicable	

DNA and RNA sequences	Information included in the manuscript?	In which section is the information available? (Reagents and Tools Table, Materials and Methods, Figures, Data Availability Section)
**Short novel DNA or RNA including primers, probes**: provide the sequences.	Not Applicable	

Cell materials	Information included in the manuscript?	In which section is the information available? (Reagents and Tools Table, Materials and Methods, Figures, Data Availability Section)
**Cell lines:** Provide species information, strain. Provide accession number in repository **OR** supplier name, catalog number, clone number, and/**OR** RRID.	Not Applicable	
**Primary cultures:** Provide species, strain, sex of origin, genetic modification status.	Not Applicable	
Report if the cell lines were recently **authenticated** (e.g., by STR profiling) and tested for mycoplasma contamination.	Not Applicable	

Experimental animals	Information included in the manuscript?	In which section is the information available? (Reagents and Tools Table, Materials and Methods, Figures, Data Availability Section)
**Laboratory animals or Model organisms:** Provide species, strain, sex, age, genetic modification status. Provide accession number in repository **OR** supplier name, catalog number, clone number, **OR** RRID.	Not Applicable	
**Animal observed in or captured from the field:** Provide species, sex, and age where possible.	Not Applicable	
Please detail **housing and husbandry conditions**.	Not Applicable	

Plants and microbes	Information included in the manuscript?	In which section is the information available? (Reagents and Tools Table, Materials and Methods, Figures, Data Availability Section)
**Plants:** provide species and strain, ecotype and cultivar where relevant, unique accession number if available, and source (including location for collected wild specimens).	Not Applicable	
**Microbes:** provide species and strain, unique accession number if available, and source.	Not Applicable	

Human research participants	Information included in the manuscript?	In which section is the information available? (Reagents and Tools Table, Materials and Methods, Figures, Data Availability Section)
If collected and within the bounds of privacy constraints report on age, sex and gender or ethnicity for all study participants.	Not Applicable	

Core facilities	Information included in the manuscript?	In which section is the information available? (Reagents and Tools Table, Materials and Methods, Figures, Data Availability Section)
If your work benefited from core facilities, was their service mentioned in the acknowledgments section?	Yes	Acknowledgement

Design

Study protocol	Information included in the manuscript?	In which section is the information available? (Reagents and Tools Table, Materials and Methods, Figures, Data Availability Section)
If study protocol has been **pre-registered, provide DOI in the manuscript**. For clinical trials, provide the trial registration number **OR** cite DOI.	Not Applicable	
Report the **clinical trial registration number** (at ClinicalTrials.gov or equivalent), where applicable.	Not Applicable	

Laboratory protocol	Information included in the manuscript?	In which section is the information available? (Reagents and Tools Table, Materials and Methods, Figures, Data Availability Section)
Provide DOI OR other citation details if **external detailed step-by-step protocols** are available.	Not Applicable	

Experimental study design and statistics	Information included in the manuscript?	In which section is the information available? (Reagents and Tools Table, Materials and Methods, Figures, Data Availability Section)
Include a statement about **sample size** estimate even if no statistical methods were used.	Not Applicable	
Were any steps taken to minimize the effects of subjective bias when allocating animals/samples to treatment (e.g. **randomization procedure**)? If yes, have they been described?	Not Applicable	
Include a statement about **blinding** even if no blinding was done.	Not Applicable	
Describe **inclusion/exclusion criteria** if samples or animals were excluded from the analysis. Were the criteria pre-established?<br><br>If sample or data points were omitted from analysis, report if this was due to attrition or intentional exclusion and provide justification.	Not Applicable	
For every figure, are **statistical tests** justified as appropriate? Do the data meet the assumptions of the tests (e.g., normal distribution)? Describe any methods used to assess it. Is there an estimate of variation within each group of data? **I**s the variance similar between the groups that are being statistically compared?	Not Applicable	

Sample definition and in-laboratory replication	Information included in the manuscript?	In which section is the information available? (Reagents and Tools Table, Materials and Methods, Figures, Data Availability Section)
In the figure legends: state number of times the experiment was **replicated** in laboratory.	Yes	Figure legend
In the figure legends: define whether data describe **technical or biological replicates**.	Yes	Figure legend

Ethics

Ethics	Information included in the manuscript?	In which section is the information available? (Reagents and Tools Table, Materials and Methods, Figures, Data Availability Section)
Studies involving **human participants**: State details of **authority granting ethics approval** (IRB or equivalent committee(s), provide reference number for approval.	Not Applicable	
Studies involving **human participants**: Include a statement confirming that **informed consent** was obtained from all subjects and that the experiments conformed to the principles set out in the WMA Declaration of Helsinki and the Department of Health and Human Services Belmont Report.	Not Applicable	
Studies involving **human participants:** For publication of **patient photos**, include a statement confirming that consent to publish was obtained.	Not Applicable	
Studies involving experimental **animals**: State details of **authority granting ethics approval** (IRB or equivalent committee(s), provide reference number for approval. Include a statement of compliance with ethical regulations.	Not Applicable	
Studies involving **specimen and field samples:** State if relevant **permits** obtained, provide details of authority approving study; if none were required, explain why.	Not Applicable	

Dual Use Research of Concern (DURC)	Information included in the manuscript?	In which section is the information available? (Reagents and Tools Table, Materials and Methods, Figures, Data Availability Section)
Could your study fall under dual use research restrictions? Please check biosecurity documents and list of **select agents and toxins** (CDC): https://www.selectagents.gov/sat/list.htm	Not Applicable	
If you used a select agent, is the security level of the lab appropriate and reported in the manuscript?	Not Applicable	
If a study is subject to dual use research of concern regulations, is the name of the **authority granting approval and reference number** for the regulatory approval provided in the manuscript?	Not Applicable	

Reporting

The MDAR framework recommends adoption of discipline-specific guidelines, established and endorsed through community initiatives. Journals have their own policy about requiring specific guidelines and recommendations to complement MDAR.

Adherence to community standards	Information included in the manuscript?	In which section is the information available? (Reagents and Tools Table, Materials and Methods, Figures, Data Availability Section)
State if relevant guidelines or checklists (e.g., **ICMJE, MIBBI, ARRIVE, PRISMA**) have been followed or provided.	Not Applicable	
For **tumor marker prognostic studies,** we recommend that you follow the **REMARK** reporting guidelines (see link list at top right). See author guidelines, under 'Reporting Guidelines'. Please confirm you have followed these guidelines.	Not Applicable	
For **phase II and III randomized controlled trials**, please refer to the **CONSORT** flow diagram (see link list at top right) and submit the CONSORT checklist (see link list at top right) with your submission. See author guidelines, under 'Reporting Guidelines'. Please confirm you have submitted this list.	Not Applicable	

Data Availability

Data availability	Information included in the manuscript?	In which section is the information available? (Reagents and Tools Table, Materials and Methods, Figures, Data Availability Section)
Have **primary datasets** been deposited according to the journal's guidelines (see 'Data Deposition' section) and the respective accession numbers provided in the Data Availability Section?	Yes	Data availability
Were **human clinical and genomic datasets** deposited in a public access-controlled repository in accordance to ethical obligations to the patients and to the applicable consent agreement?	Not Applicable	
Are **computational models** that are central and integral to a study available without restrictions in a machine-readable form? Were the relevant accession numbers or links provided?	Not Applicable	
If publicly available data were reused, provide the respective **data citations in the reference list**.	Not Applicable	
