# Supplementary material for: FAF1 and FAF2 enhance unfolding by p97-UFD1-NPL4 complex enabling rational design of p97 activators
Source: EMBO J. 2026 Aug 15;45(18):6595–629. doi: 10.1038/s44318-026-00894-x (PMC13578712; doi:10.1038/s44318-026-00894-x)

UFD1 1-19: MFSFNMFDHPIPRVFQNRF (#1)

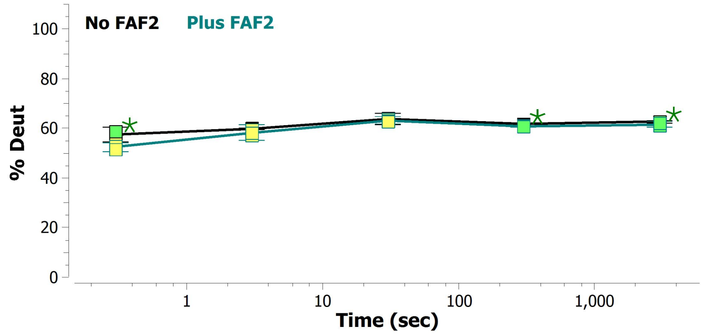

UFD1 23-30: YRCFSVSM (#2)

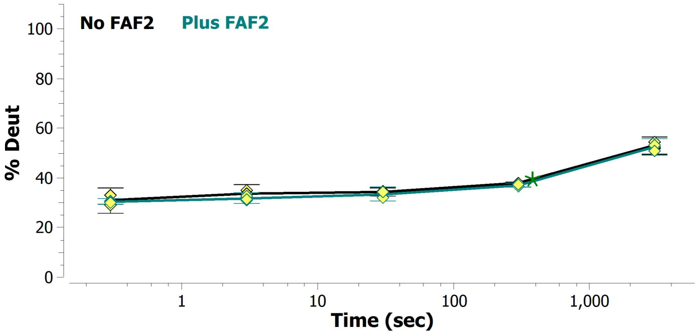

UFD1 23-57: YRCFSVSMLAGPNDRSDVEKGGKIIMPPSALDQLS (#3)

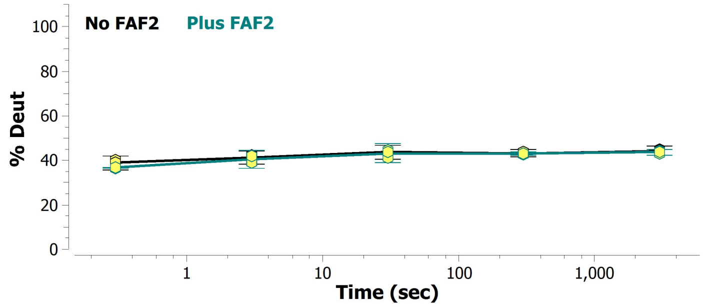

UFD1 26-35: FSVSMLAGPN (#4)

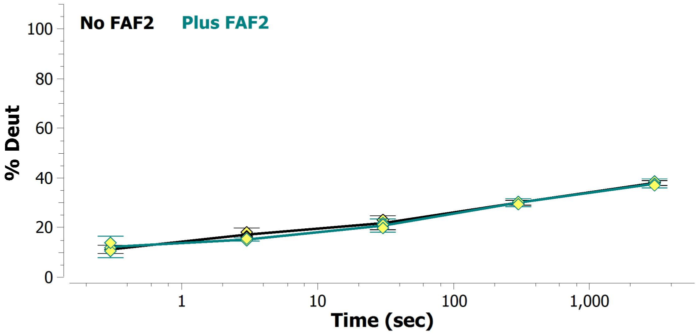

UFD1 31-53: LAGPNDRSDVEKGGKIIMPPSAL (#5)

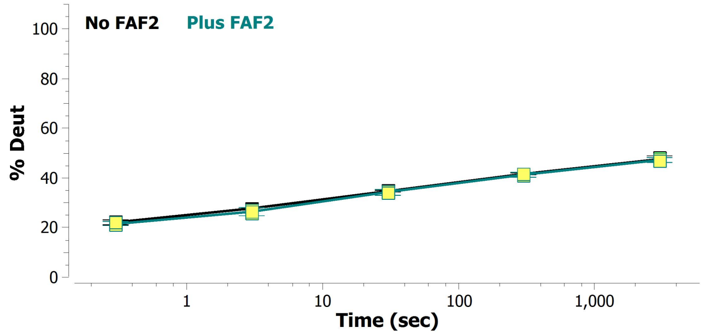

UFD1 31-56: LAGPNDRSDVEKGGKIIMPPSALDQL (#6)

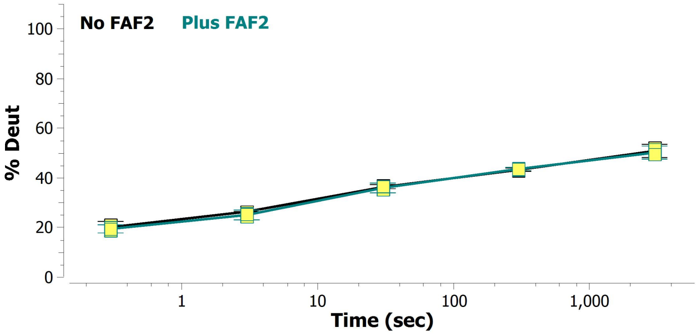

UFD1 37-67: RSDVEKGGKIIMPPSALDQLSRLNITYPMLF (#7)

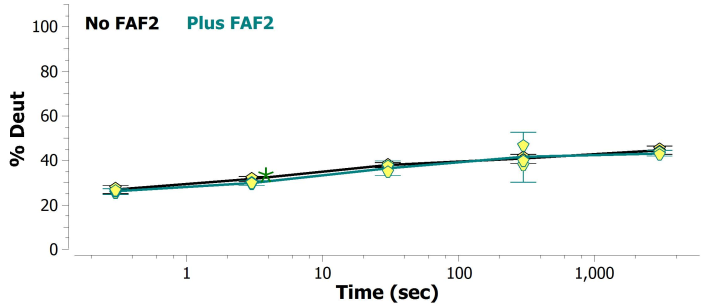

UFD1 45-58: KIIMPPSALDQLSR (#8)

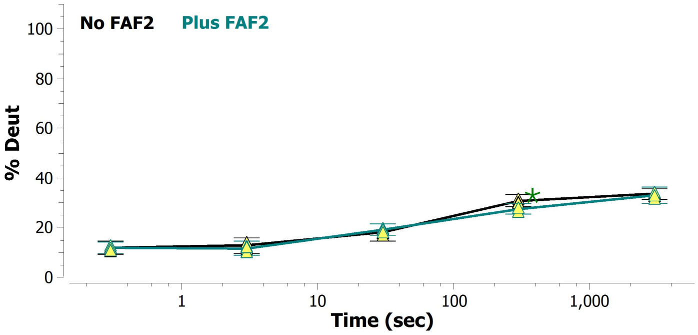

UFD1 47-70: IMPPSALDQLSRLNITYPMLFKLT (#9)

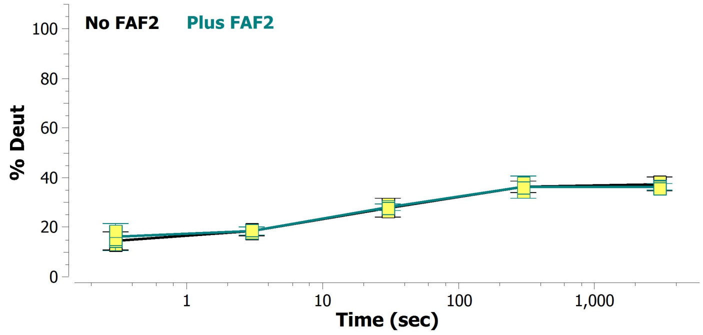

UFD1 67-84: FKLTNKNSDRMTHCGVLE (#10)

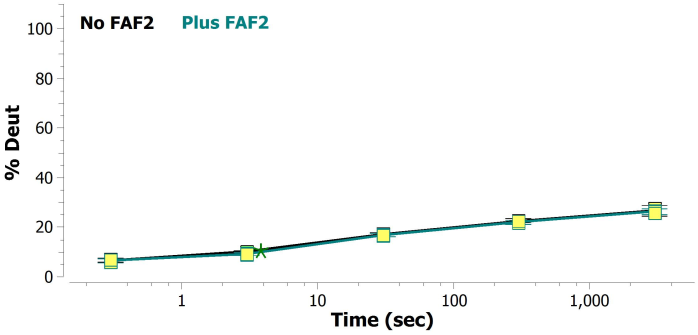

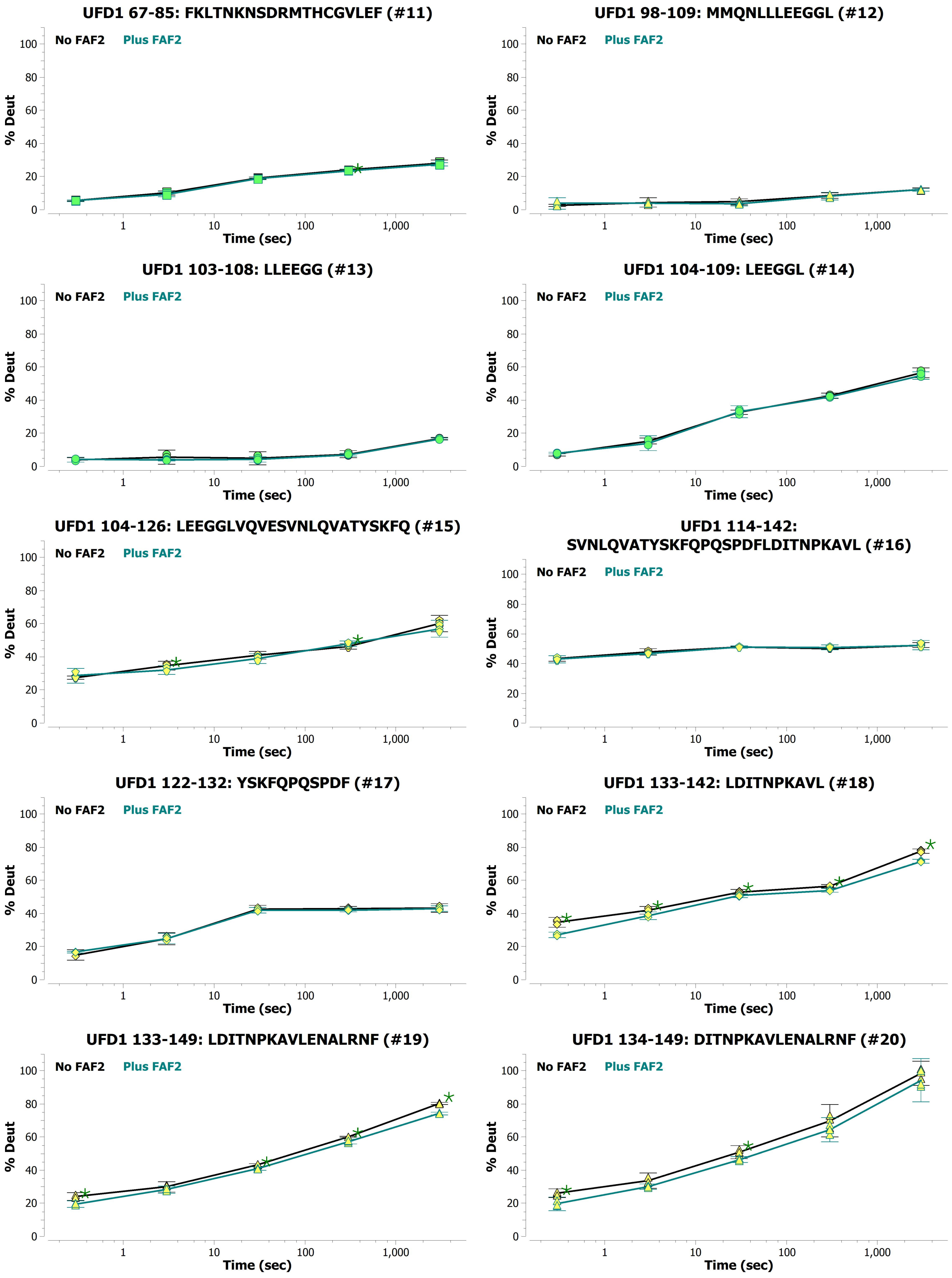
UFD1 67-85: FKLTNKNSDRMTHCGVLEF (#11)
UFD1 98-109: MMQNLLLEEGGL (#12)
UFD1 103-108: LLEEGG (#13)
UFD1 104-109: LEEGGL (#14)
UFD1 104-126: LEEGGLVQVESVNLQVATYSKFQ (#15)
UFD1 114-142: SVNLQVATYSKFQPQSPDFLDITNPKAVL (#16)
UFD1 122-132: YSKFQPQSPDF (#17)
UFD1 133-142: LDITNPKAVL (#18)
UFD1 133-149: LDITNPKAVLENALRNF (#19)
UFD1 134-149: DITNPKAVLENALRNF (#20)
No FAF2
Plus FAF2
% Deut
Time (sec)
0
20
40
60
80
100
1
10
100
1,000

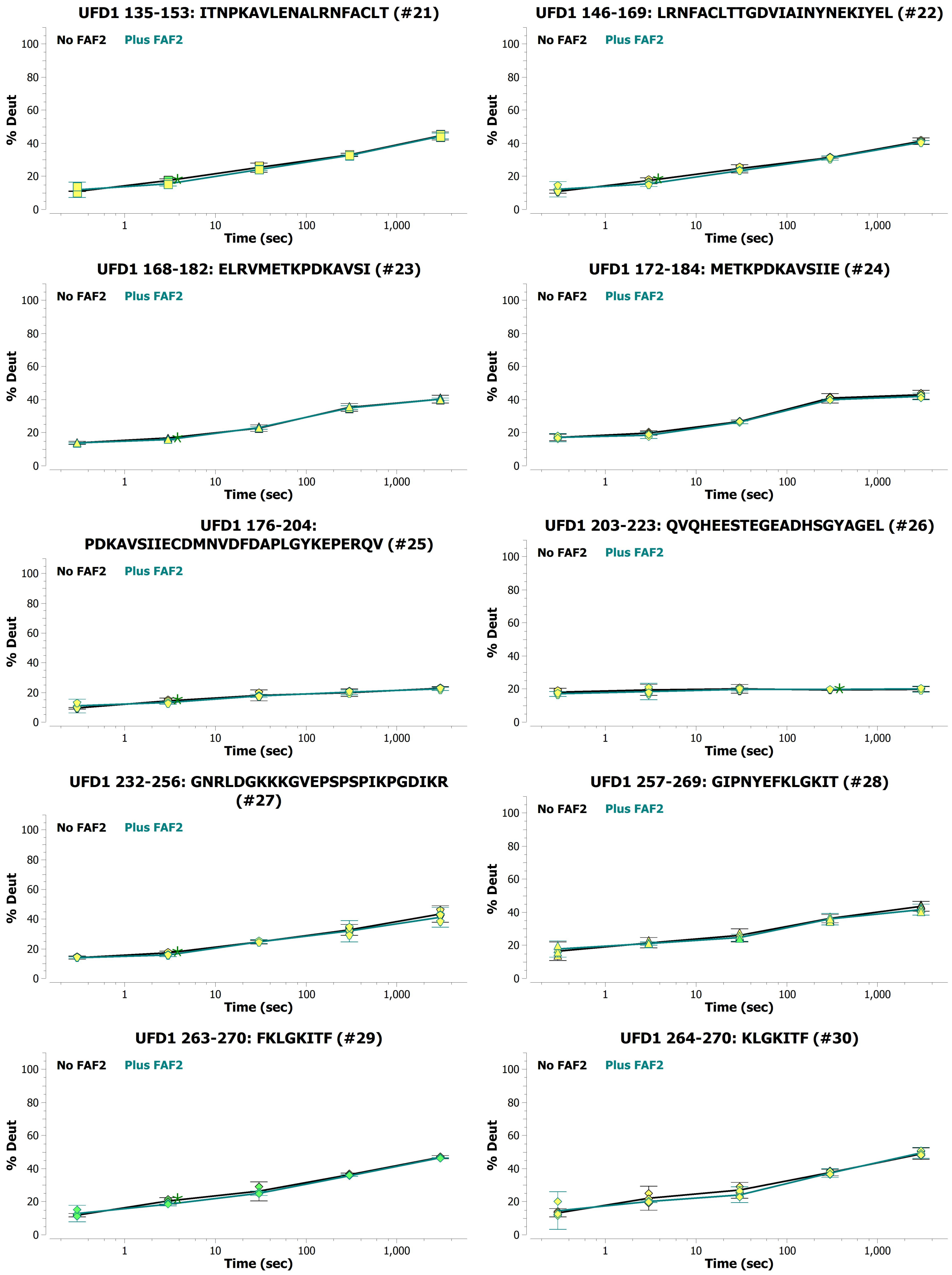

UFD1 135-153: ITNPKAVLENALRNFACLT (#21)
UFD1 146-169: LRNFACLTTGDVIAINYNEKIYEL (#22)
UFD1 168-182: ELRVMETKPDKAVSI (#23)
UFD1 172-184: METKPDKAVSIIE (#24)
UFD1 176-204: PDKAVSIIECDMNVDFDAPLGYKEPERQV (#25)
UFD1 203-223: QVQHEESTEGEADHSGYAGEL (#26)
UFD1 232-256: GNRLDGKKKGVEPSPSPIKPGDIKR (#27)
UFD1 257-269: GIPNYEFKLGKIT (#28)
UFD1 263-270: FKLGKITF (#29)
UFD1 264-270: KLGKITF (#30)
No FAF2
Plus FAF2
% Deut
Time (sec)
0
20
40
60
80
100
1
10
100
1,000

UFD1 264-291: KLGKITFIRNSRPLVKKVEEDEAGGRFV (#31)

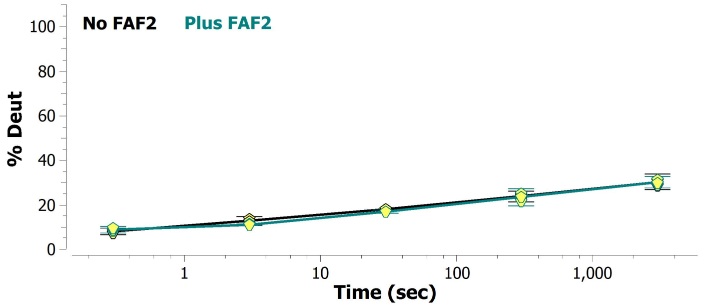

UFD1 270-289: FIRNSRPLVKKVEEDEAGGR (#32)

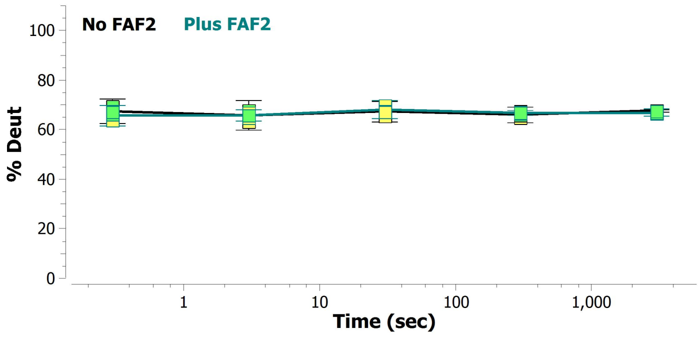

UFD1 271-290: IRNSRPLVKKVEEDEAGGRF (#33)

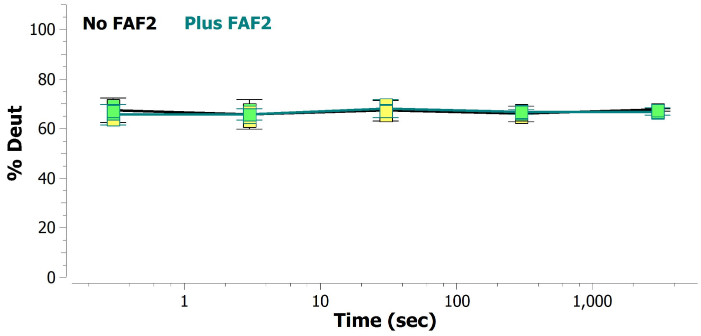

UFD1 289-301: RFVAFSGEGQSLR (#34)

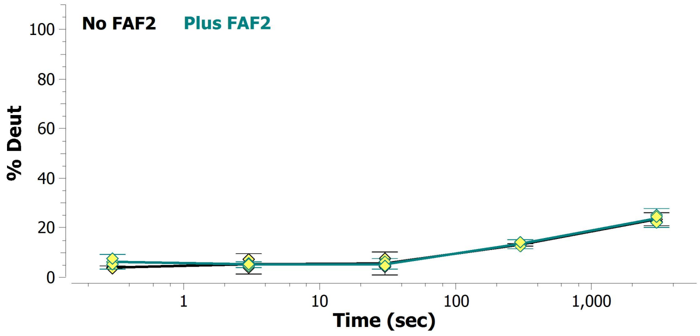

UFD1 291-307: VAFSGEGQSLRKKGRKP (#35)

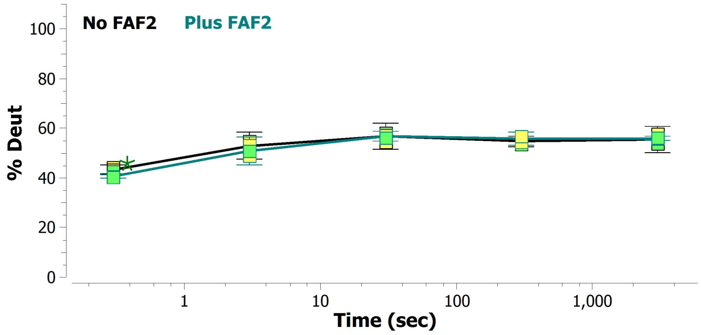

UFD1 293-307: FSGEGQSLRKKGRKP (#36)

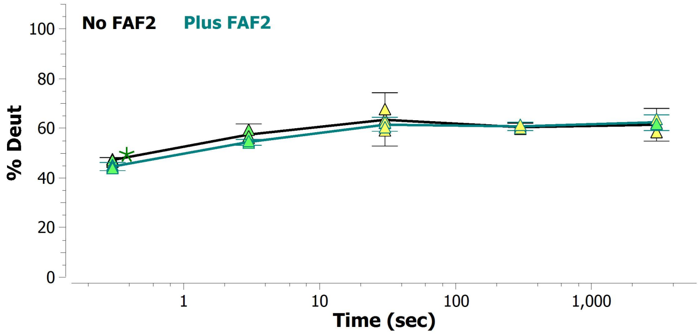

NPL4 2-28: AESIIIRVQSPDGVKRITATKRETAAT (#37)

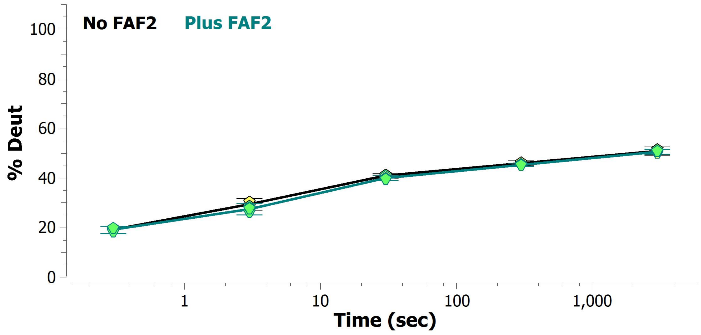

NPL4 2-29: AESIIIRVQSPDGVKRITATKRETAATF (#38)

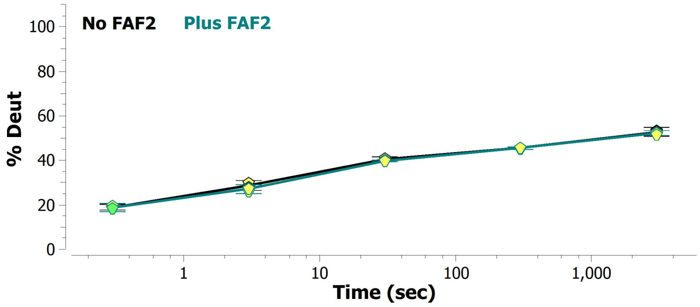

NPL4 4-28: SIIIRVQSPDGVKRITATKRETAAT (#39)

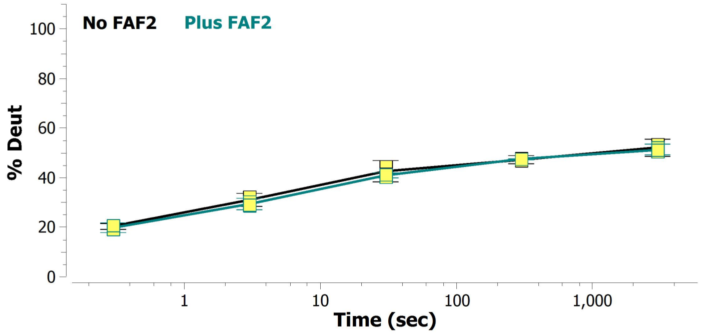

NPL4 5-20: IIIRVQSPDGVKRITA (#40)

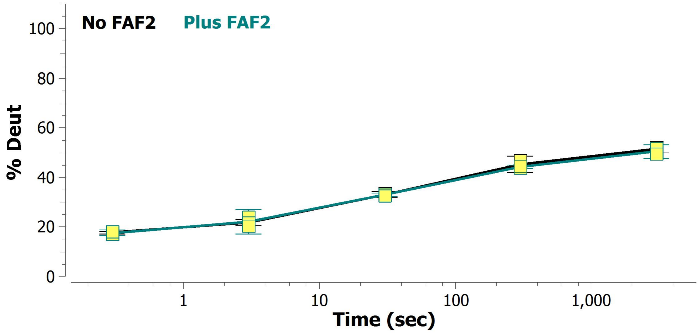

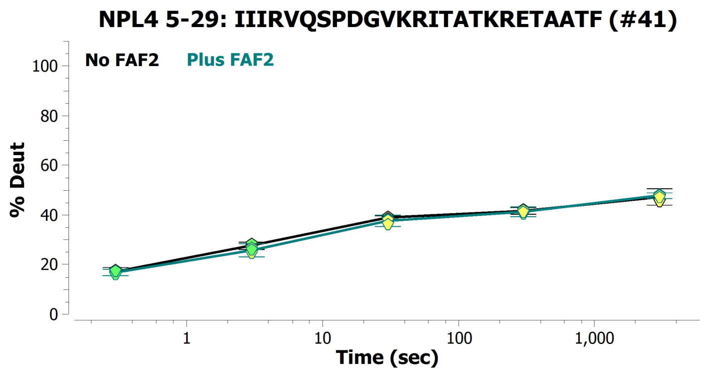
NPL4 5-29: IIIRVQSPDGVKRITATKRETAATF (#41)
No FAF2
Plus FAF2
% Deut
0
20
40
60
80
100
1
10
100
1,000
Time (sec)

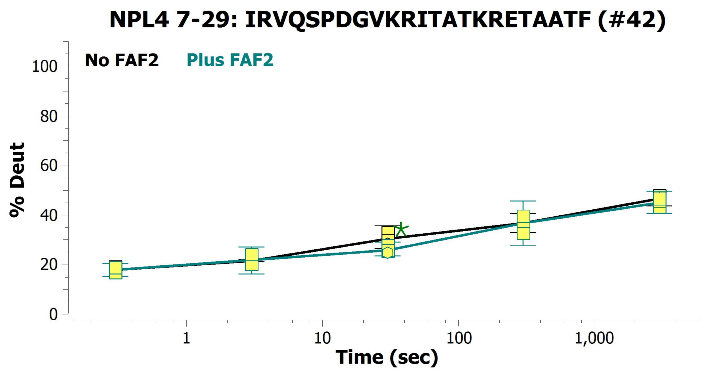
NPL4 7-29: IRVQSPDGVKRITATKRETAATF (#42)
No FAF2
Plus FAF2
% Deut
0
20
40
60
80
100
1
10
100
1,000
Time (sec)

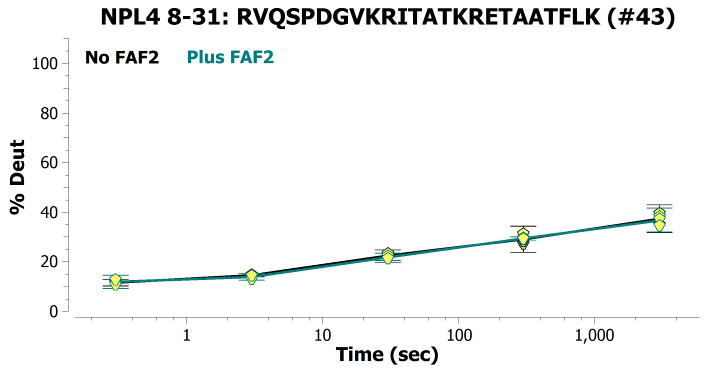
NPL4 8-31: RVQSPDGVKRITATKRETAATFLK (#43)
No FAF2
Plus FAF2
% Deut
0
20
40
60
80
100
1
10
100
1,000
Time (sec)

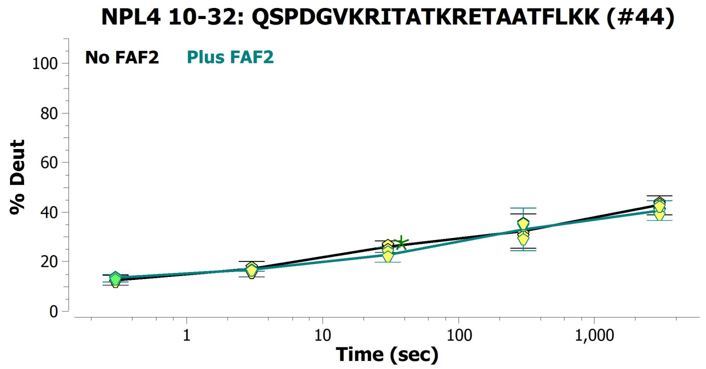
NPL4 10-32: QSPDGVKRITATKRETAATFLKK (#44)
No FAF2
Plus FAF2
% Deut
0
20
40
60
80
100
1
10
100
1,000
Time (sec)

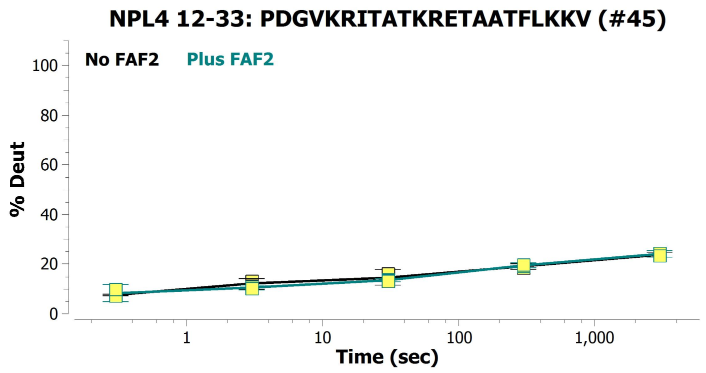
NPL4 12-33: PDGVKRITATKRETAATFLKKV (#45)
No FAF2
Plus FAF2
% Deut
0
20
40
60
80
100
1
10
100
1,000
Time (sec)

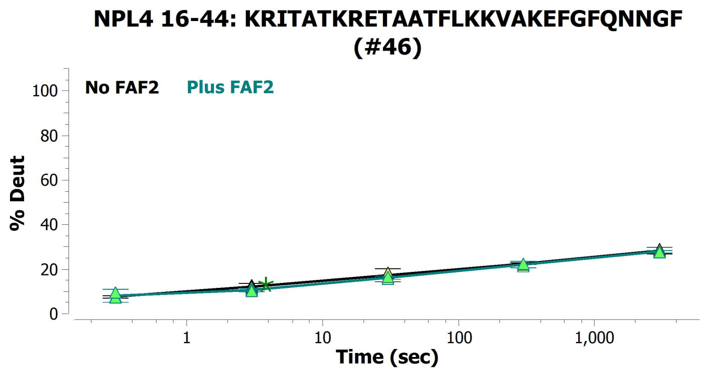
NPL4 16-44: KRITATKRETAATFLKKVAKEFGFQNNGF (#46)
No FAF2
Plus FAF2
% Deut
0
20
40
60
80
100
1
10
100
1,000
Time (sec)

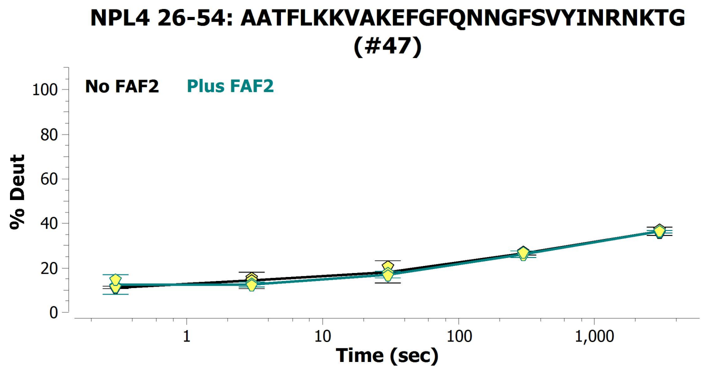
NPL4 26-54: AATFLKKVAKEFGFQNNGFSVYINRNKTG (#47)
No FAF2
Plus FAF2
% Deut
0
20
40
60
80
100
1
10
100
1,000
Time (sec)

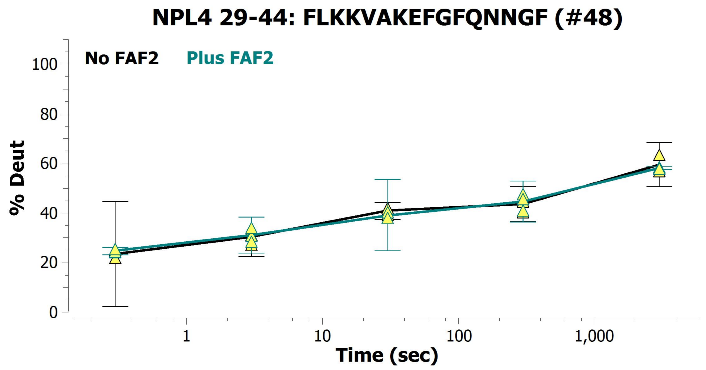
NPL4 29-44: FLKKVAKEFGFQNNGF (#48)
No FAF2
Plus FAF2
% Deut
0
20
40
60
80
100
1
10
100
1,000
Time (sec)

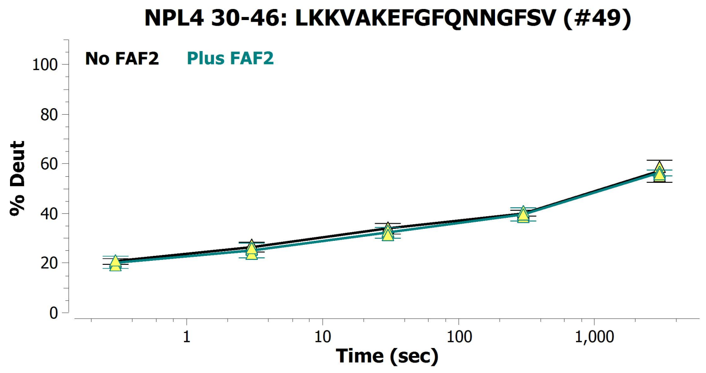
NPL4 30-46: LKKVAKEFGFQNNGFSV (#49)
No FAF2
Plus FAF2
% Deut
0
20
40
60
80
100
1
10
100
1,000
Time (sec)

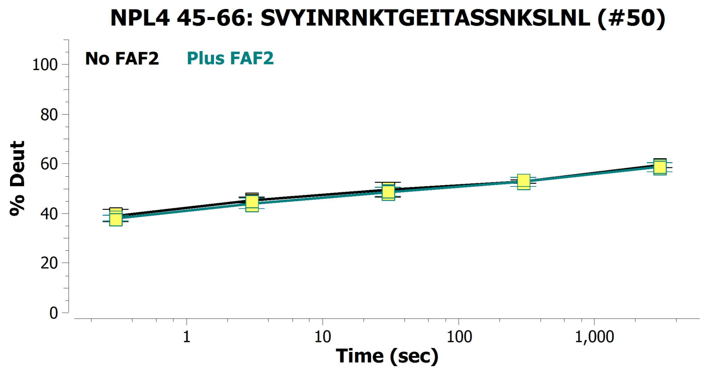
NPL4 45-66: SVYINRNKTGEITASSNKSLNL (#50)
No FAF2
Plus FAF2
% Deut
0
20
40
60
80
100
1
10
100
1,000
Time (sec)

NPL4 47-66: YINRNKTGEITASSNKSLNL (#51)

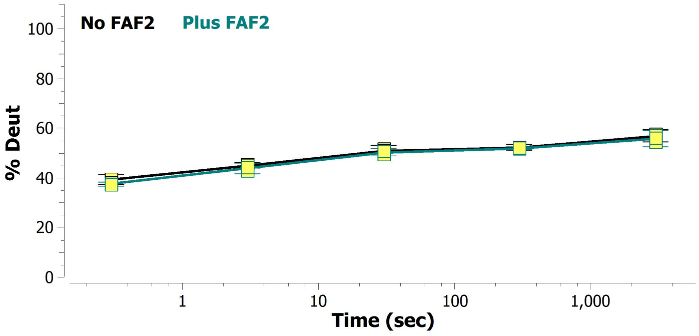

NPL4 48-66: INRNKTGEITASSNKSLNL (#52)

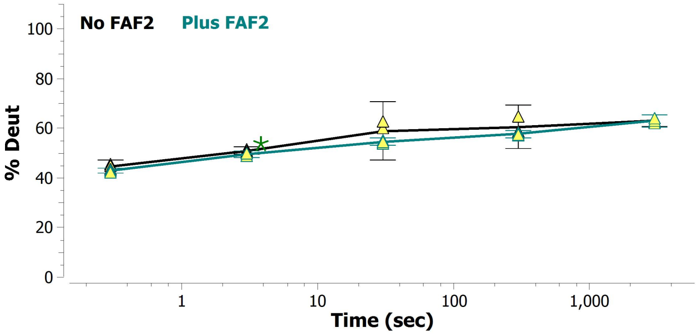

NPL4 57-71: TASSNKSLNLLKIKH (#53)

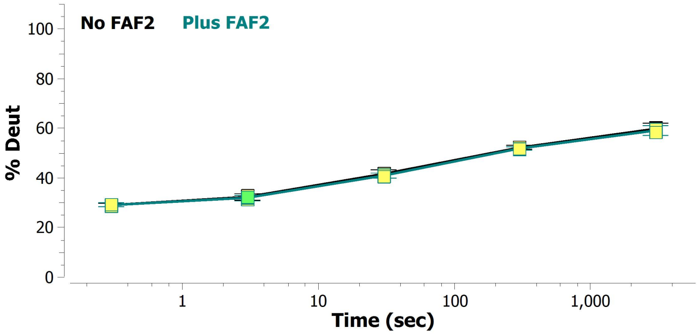

NPL4 67-75: LKIKHGDLL (#54)

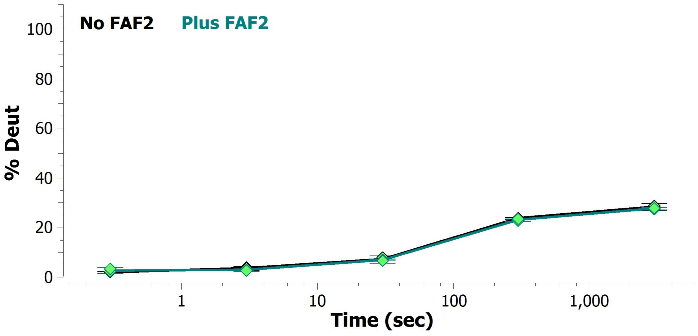

NPL4 76-88: FLFPSSLAGPSSE (#55)

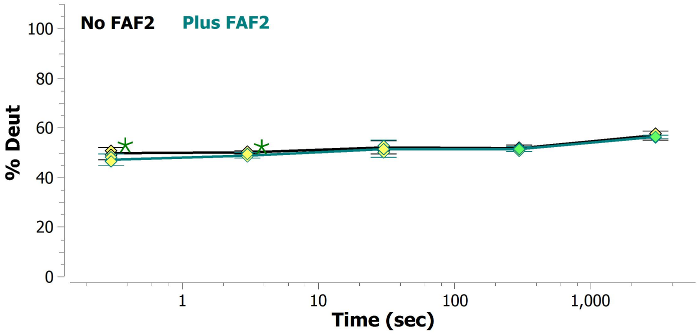

NPL4 76-89: FLFPSSLAGPSSEM (#56)

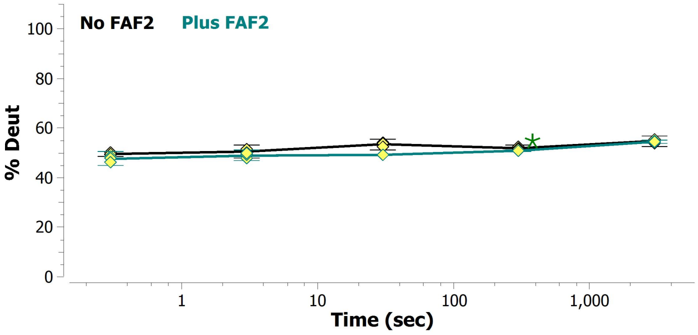

NPL4 89-100: METSVPPGFKVF (#57)

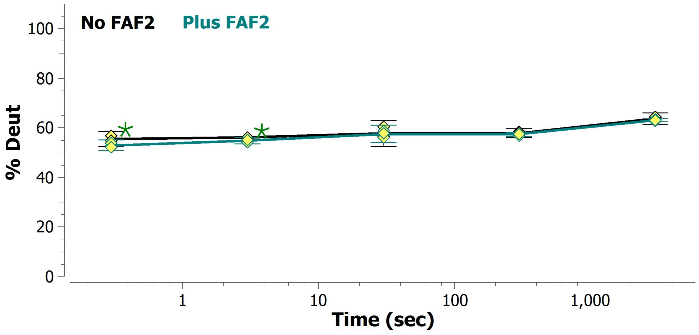

NPL4 89-107: METSVPPGFKVFGAPNVVE (#58)

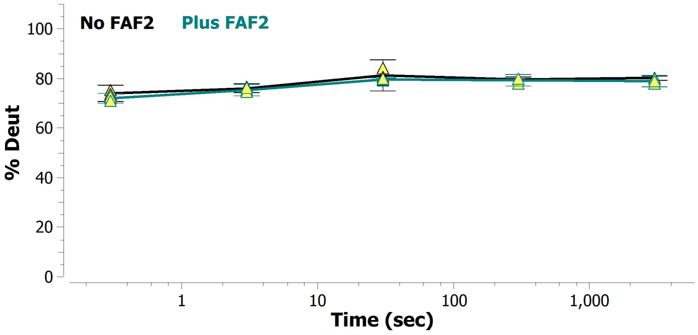

NPL4 89-109: METSVPPGFKVFGAPNVVEDE (#59)

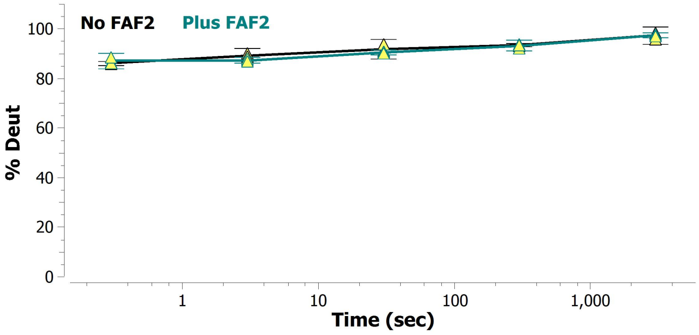

NPL4 108-129: DEIDQYLSKQDGKIYRSRDPQL (#60)

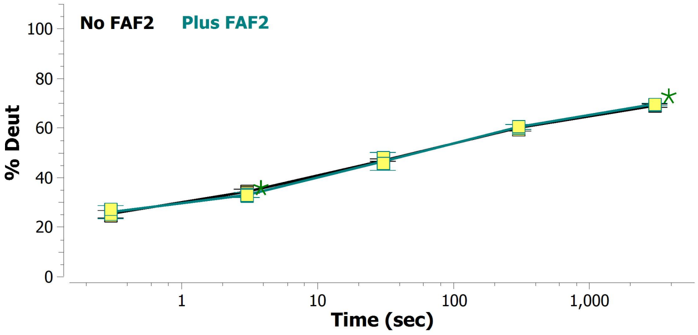

NPL4 112-124: QYLSKQDGKIYRS (#61)

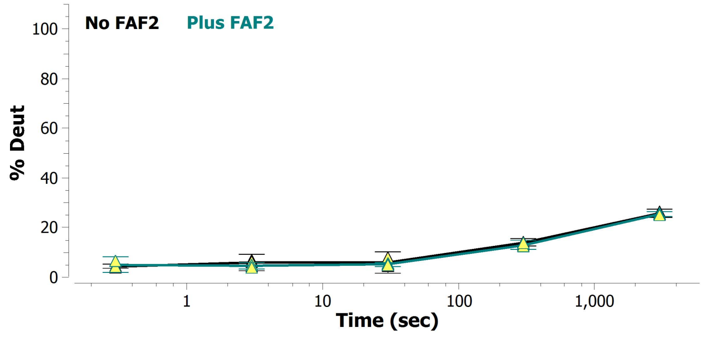

NPL4 118-134: DGKIYRSRDPQLCRHGP (#62)

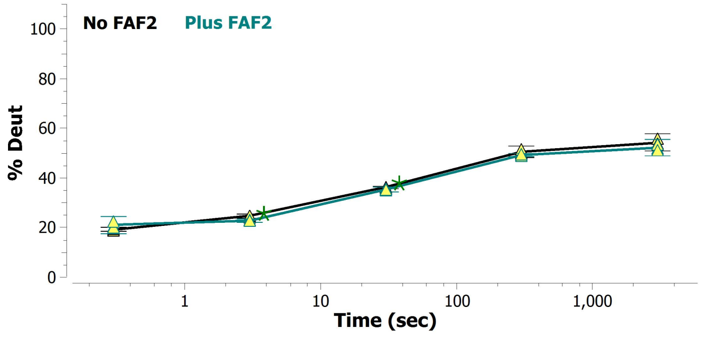

NPL4 130-164: CRHGPLGKCVHCVPLEPFDEDYLNHLEPPVKHMSF (#63)

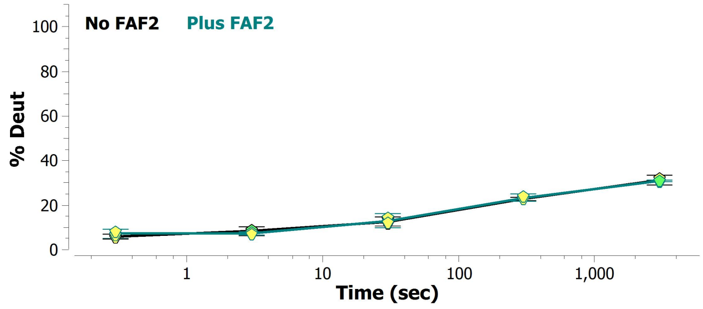

NPL4 148-167: DEDYLNHLEPPVKHMSFHAY (#64)

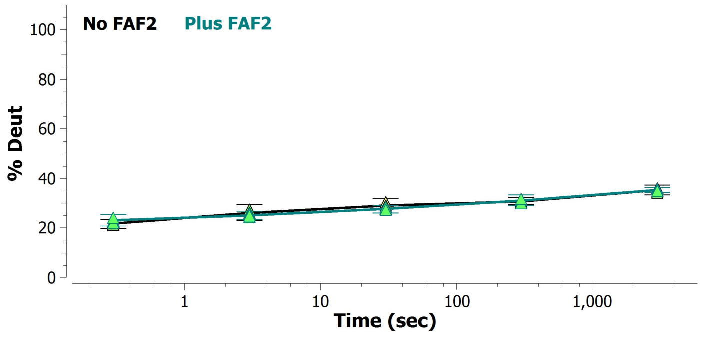

NPL4 152-164: LNHLEPPVKHMSF (#65)

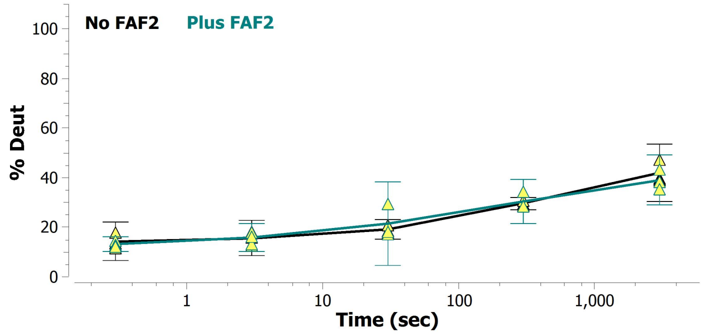

NPL4 164-179: FHAYIRKLTGGADKGK (#66)

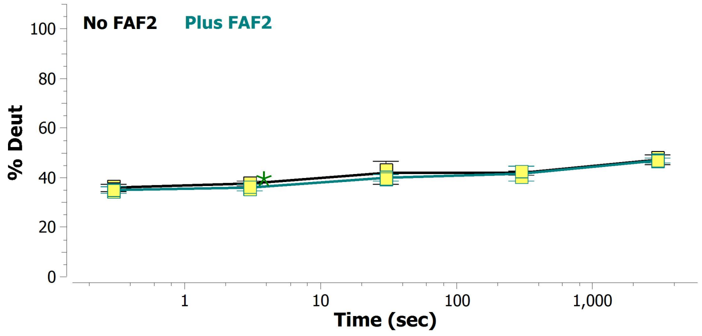

NPL4 165-180: HAYIRKLTGGADKGKF (#67)

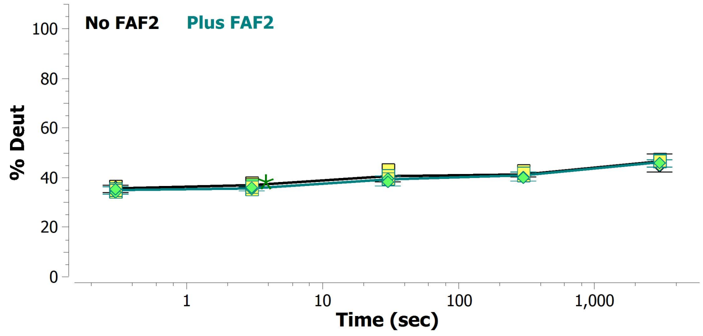

NPL4 168-202: IRKLTGGADKGKFVALENISCKIKSGCEGHLPWPN (#68)

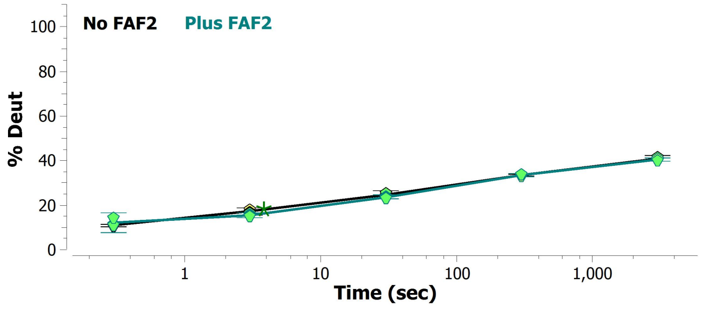

NPL4 173-190: GGADKGKFVALENISCKI (#69)

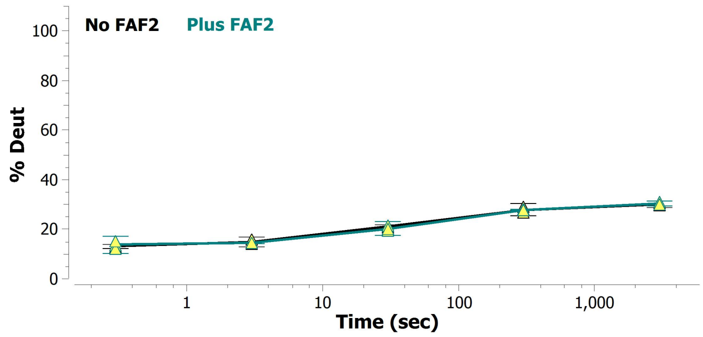

NPL4 185-215: NISCKIKSGCEGHLPWPNGICTKCQPSAITL (#70)

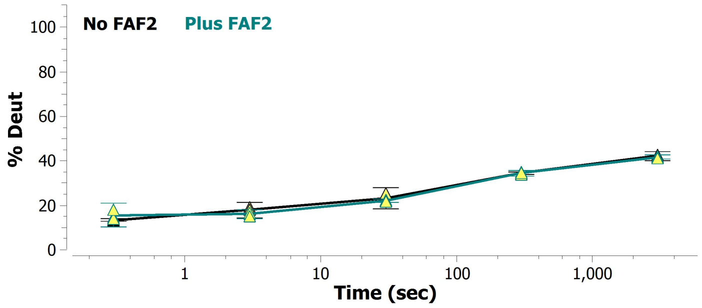

NPL4 189-203: KIKSGCEGHLPWPNG (#71)

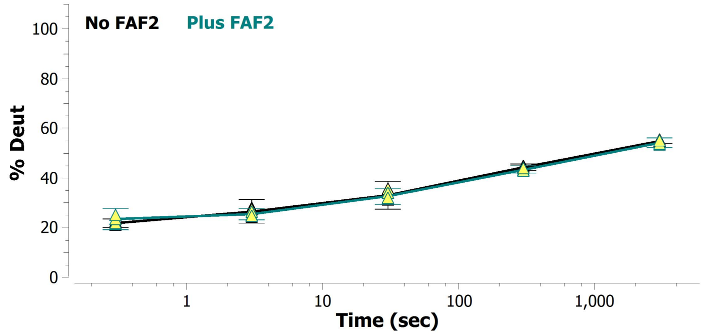

NPL4 189-213: KIKSGCEGHLPWPNGICTKCQPSAI (#72)

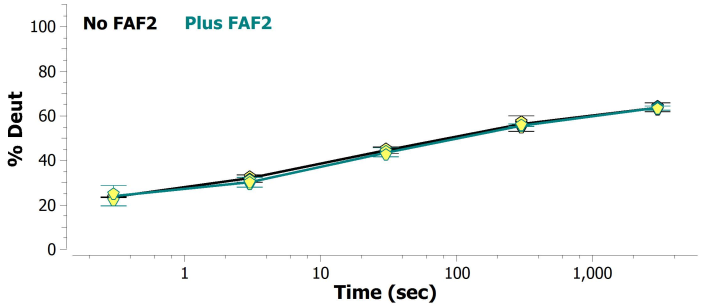

NPL4 207-227: KCQPSAITLNRQKYRHVDNIM (#73)

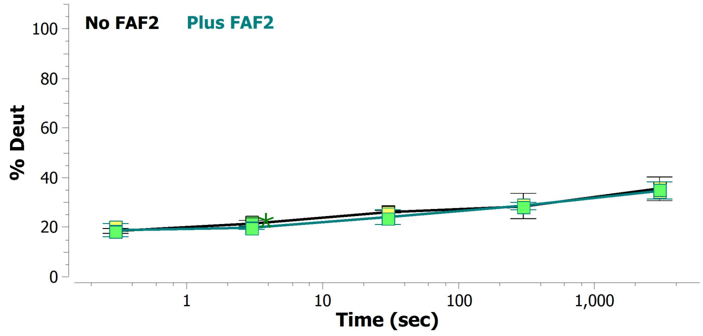

NPL4 209-222: QPSAITLNRQKYRH (#74)

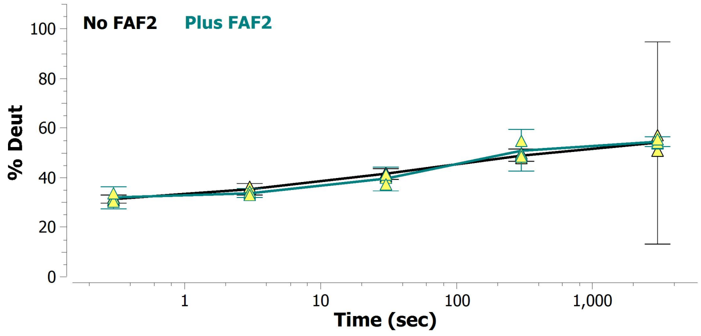

NPL4 228-240: FENHTVADRFLDF (#75)

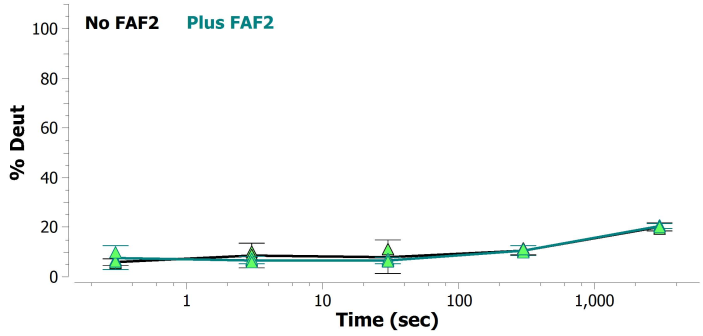

NPL4 229-240: ENHTVADRFLDF (#76)

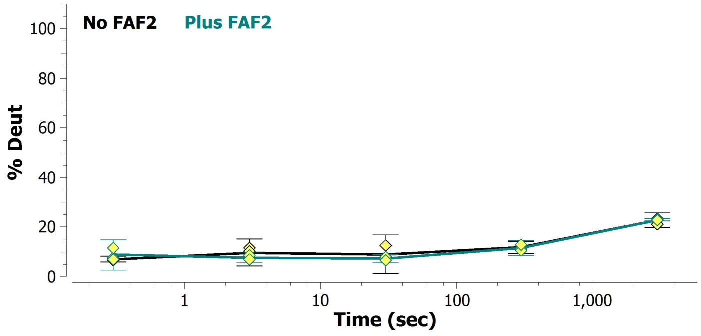

NPL4 241-252: WRKTGNQHFGYL (#77)

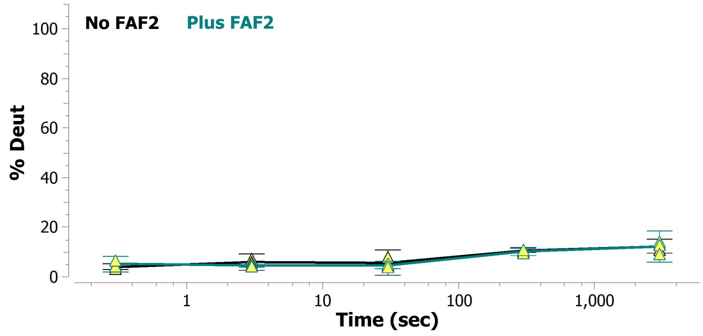

NPL4 253-268: YGRYTEHKDIPLGIRA (#78)

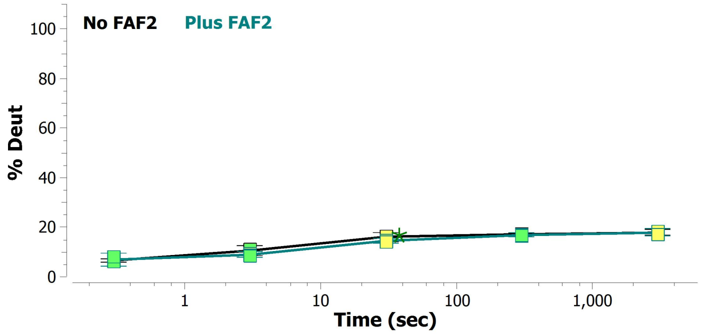

NPL4 253-271: YGRYTEHKDIPLGIRAEVA (#79)

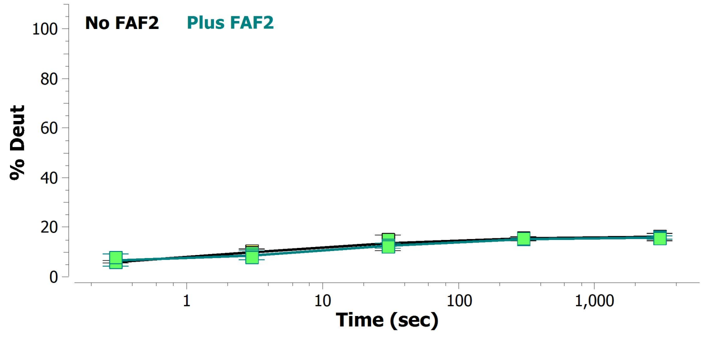

NPL4 253-272: YGRYTEHKDIPLGIRAEVAA (#80)

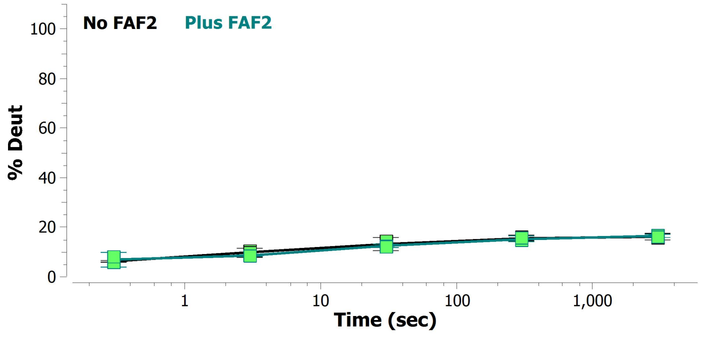

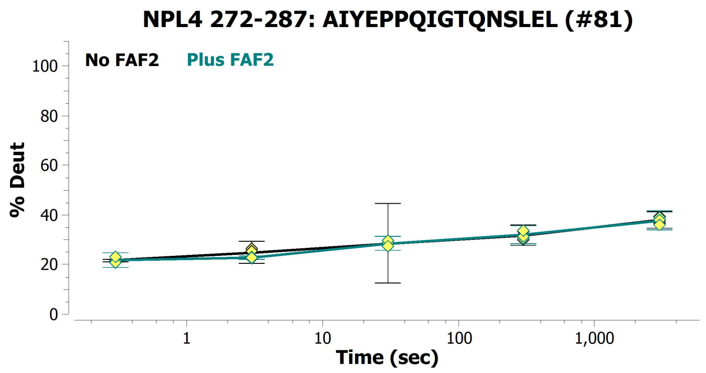
NPL4 272-287: AIYEPPQIGTQNSLEL (#81)
No FAF2
Plus FAF2
% Deut
Time (sec)

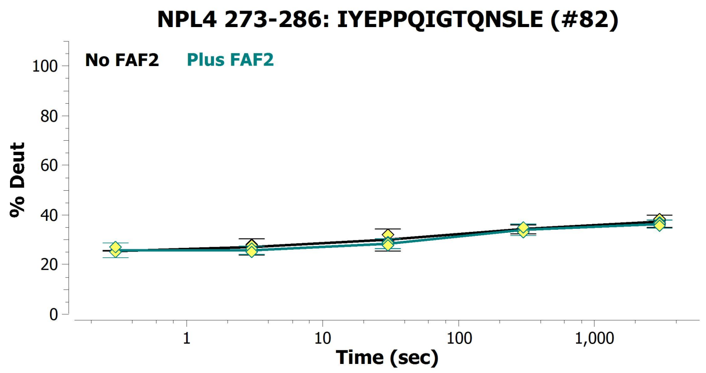
NPL4 273-286: IYEPPQIGTQNSLE (#82)
No FAF2
Plus FAF2
% Deut
Time (sec)

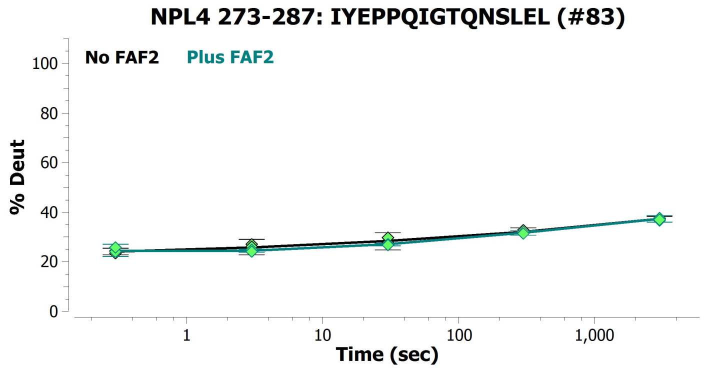
NPL4 273-287: IYEPPQIGTQNSLEL (#83)
No FAF2
Plus FAF2
% Deut
Time (sec)

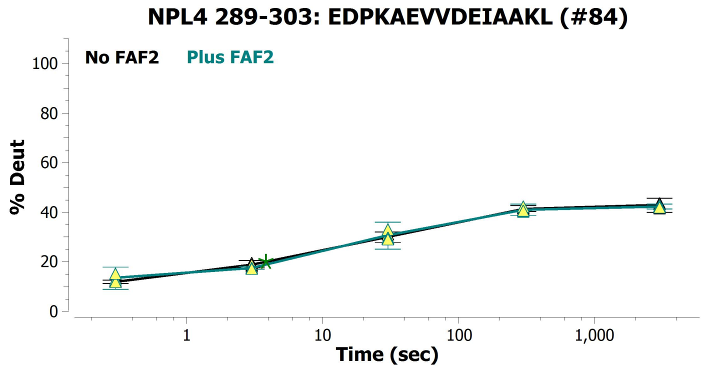
NPL4 289-303: EDPKAEVVDEIAAKL (#84)
No FAF2
Plus FAF2
% Deut
Time (sec)

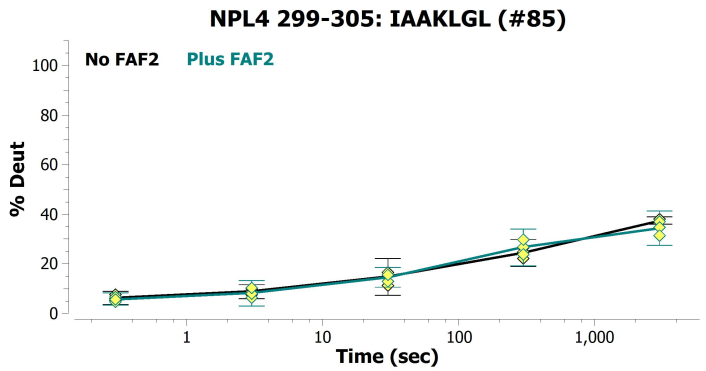
NPL4 299-305: IAAKLGL (#85)
No FAF2
Plus FAF2
% Deut
Time (sec)

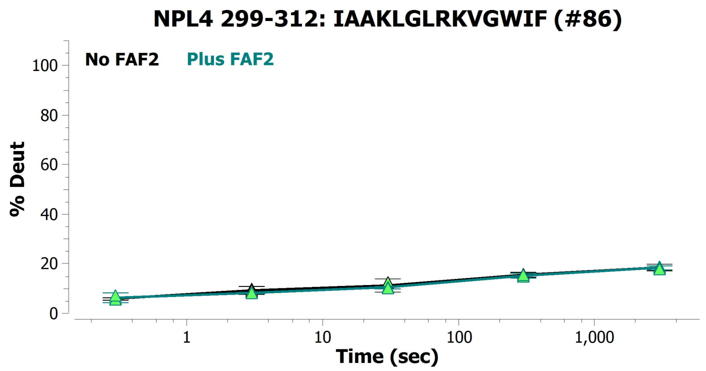
NPL4 299-312: IAAKLGLRKVGWIF (#86)
No FAF2
Plus FAF2
% Deut
Time (sec)

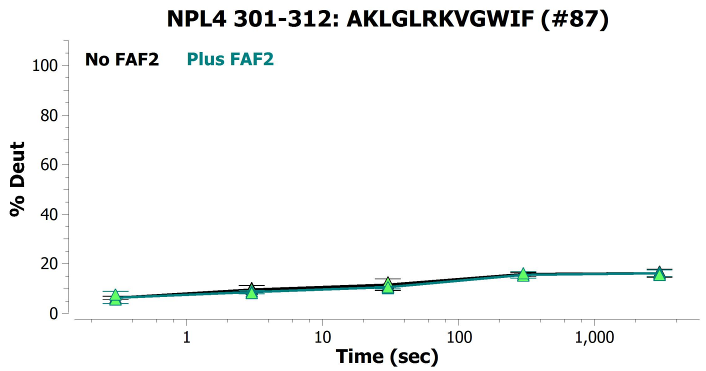
NPL4 301-312: AKLGLRKVGWIF (#87)
No FAF2
Plus FAF2
% Deut
Time (sec)

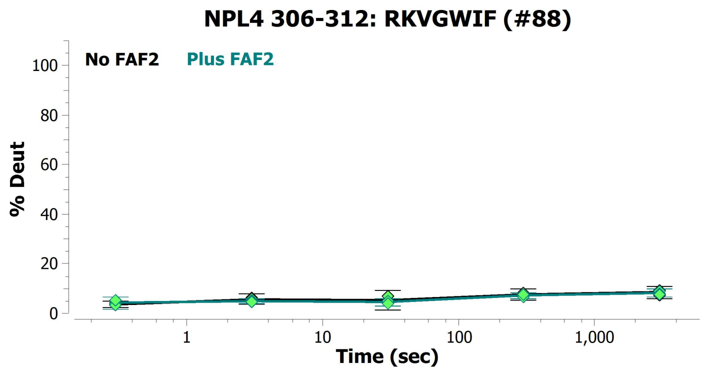
NPL4 306-312: RKVGWIF (#88)
No FAF2
Plus FAF2
% Deut
Time (sec)

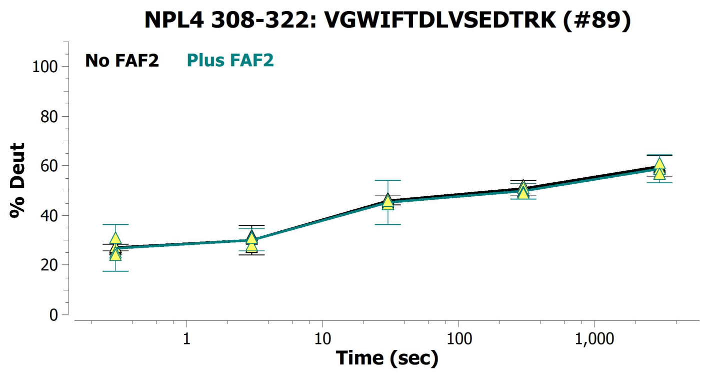
NPL4 308-322: VGWIFTDLVSEDTRK (#89)
No FAF2
Plus FAF2
% Deut
Time (sec)

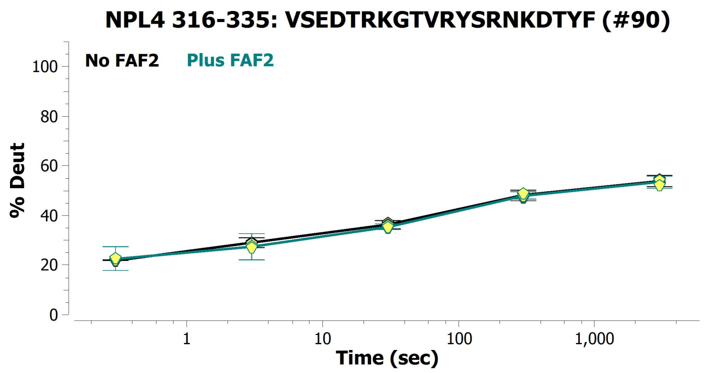
NPL4 316-335: VSEDTRKGTVRYSRNKDTYF (#90)
No FAF2
Plus FAF2
% Deut
Time (sec)

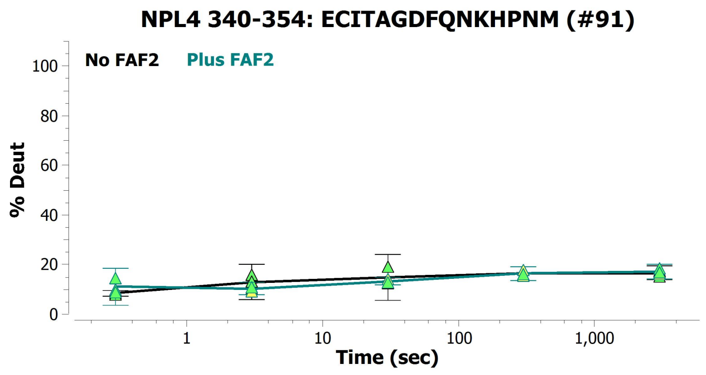
NPL4 340-354: ECITAGDFQNKHPNM (#91)
No FAF2
Plus FAF2
% Deut
Time (sec)
0
20
40
60
80
100
1
10
100
1,000

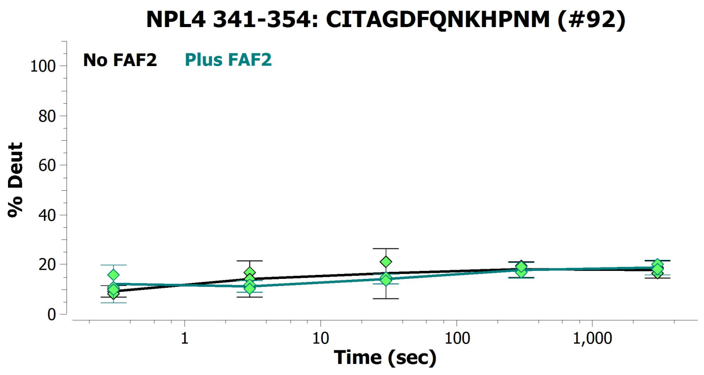
NPL4 341-354: CITAGDFQNKHPNM (#92)
No FAF2
Plus FAF2
% Deut
Time (sec)
0
20
40
60
80
100
1
10
100
1,000

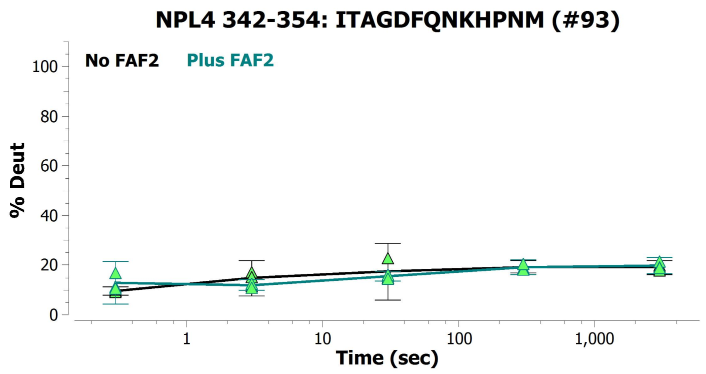
NPL4 342-354: ITAGDFQNKHPNM (#93)
No FAF2
Plus FAF2
% Deut
Time (sec)
0
20
40
60
80
100
1
10
100
1,000

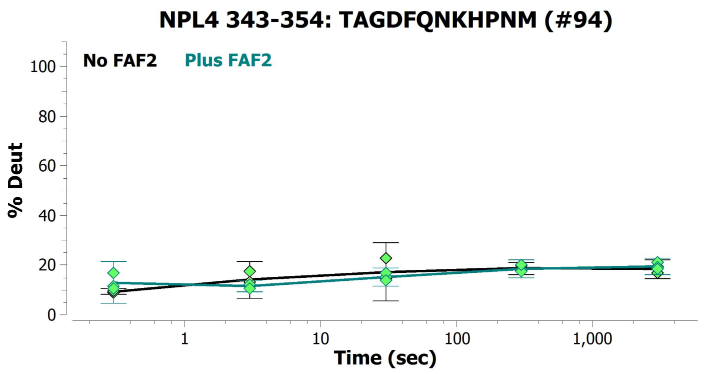
NPL4 343-354: TAGDFQNKHPNM (#94)
No FAF2
Plus FAF2
% Deut
Time (sec)
0
20
40
60
80
100
1
10
100
1,000

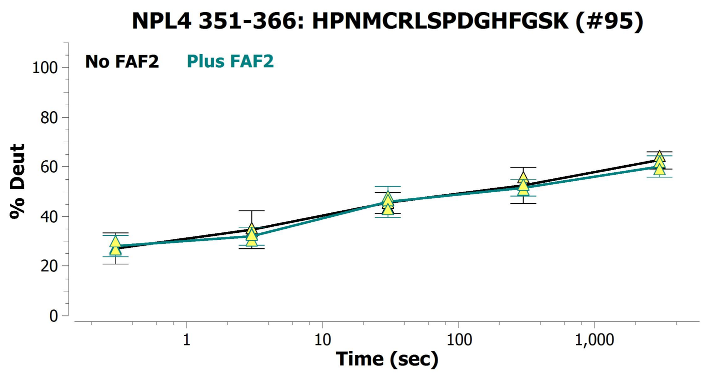
NPL4 351-366: HPNMCRLSPDGHFGSK (#95)
No FAF2
Plus FAF2
% Deut
Time (sec)
0
20
40
60
80
100
1
10
100
1,000

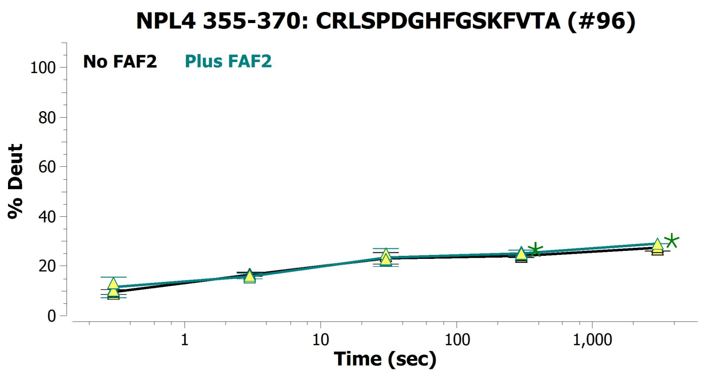
NPL4 355-370: CRLSPDGHFGSKFVTA (#96)
No FAF2
Plus FAF2
% Deut
Time (sec)
0
20
40
60
80
100
1
10
100
1,000

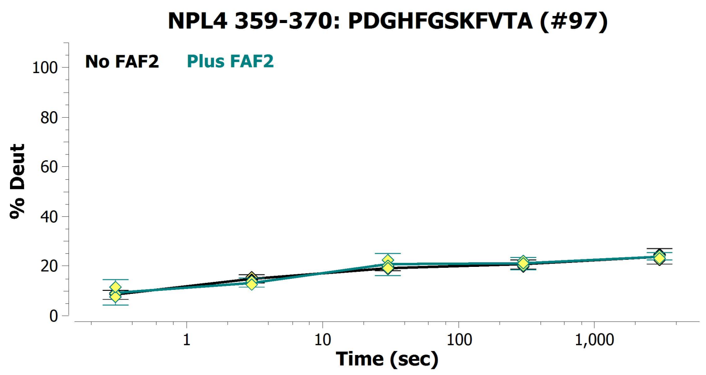
NPL4 359-370: PDGHFGSKFVTA (#97)
No FAF2
Plus FAF2
% Deut
Time (sec)
0
20
40
60
80
100
1
10
100
1,000

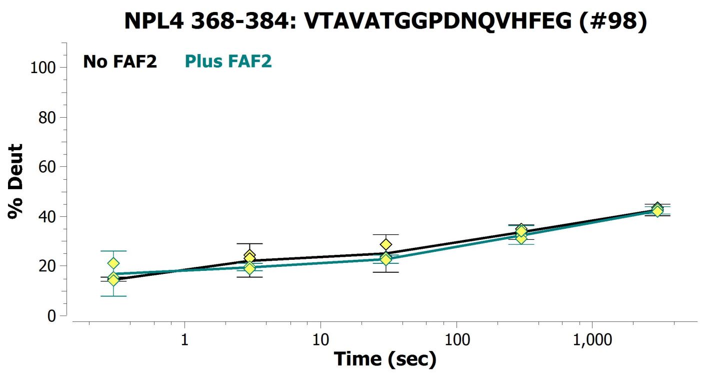
NPL4 368-384: VTAVATGGPDNQVHFEG (#98)
No FAF2
Plus FAF2
% Deut
Time (sec)
0
20
40
60
80
100
1
10
100
1,000

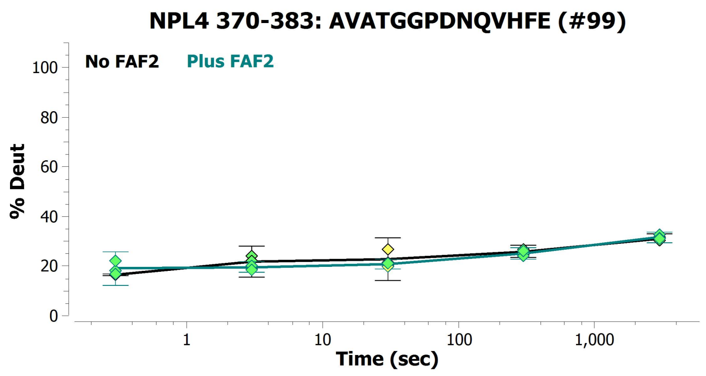
NPL4 370-383: AVATGGPDNQVHFE (#99)
No FAF2
Plus FAF2
% Deut
Time (sec)
0
20
40
60
80
100
1
10
100
1,000

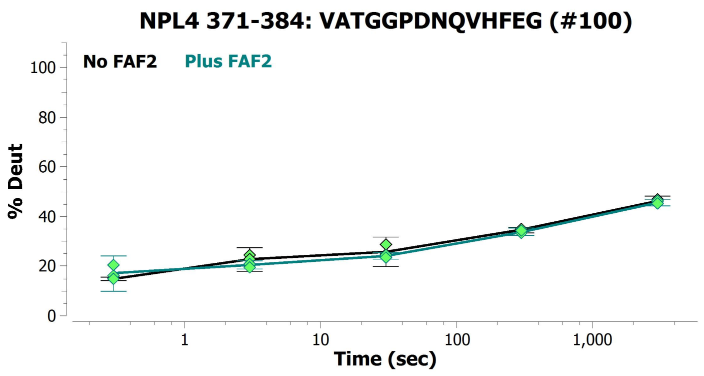
NPL4 371-384: VATGGPDNQVHFEG (#100)
No FAF2
Plus FAF2
% Deut
Time (sec)
0
20
40
60
80
100
1
10
100
1,000

NPL4 393-401: ALVRDECLL (#101)

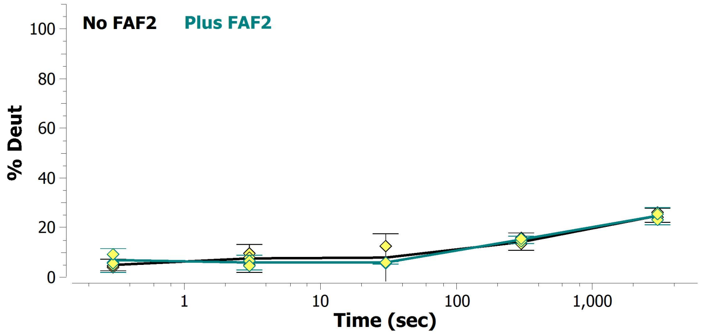

NPL4 402-411: PCKDAPELGY (#102)

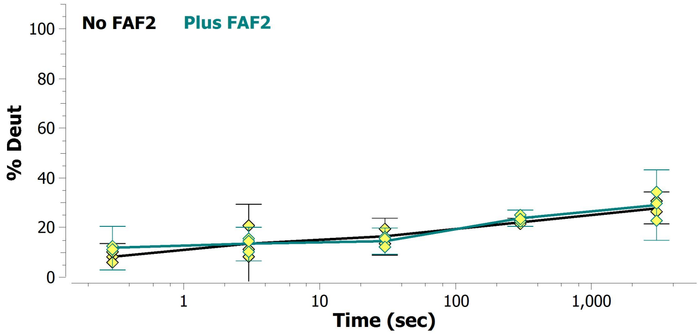

NPL4 411-425: YAKESSSEQYVPDVF (#103)

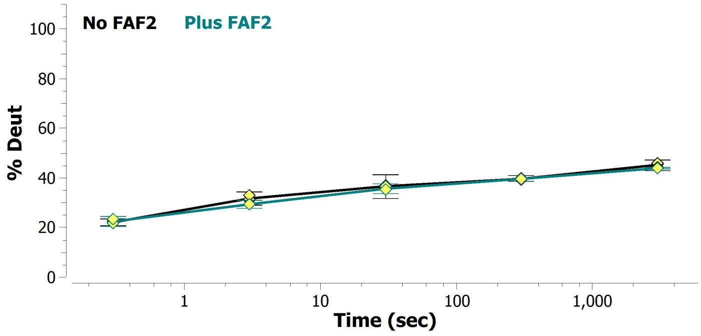

NPL4 427-436: KDVDKFGNEI (#104)

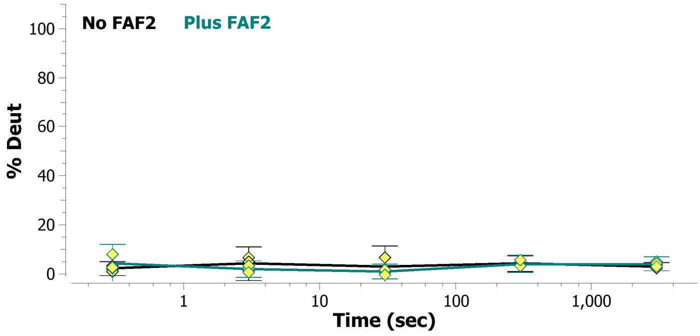

NPL4 430-445: DKFGNEITQLARPLPV (#105)

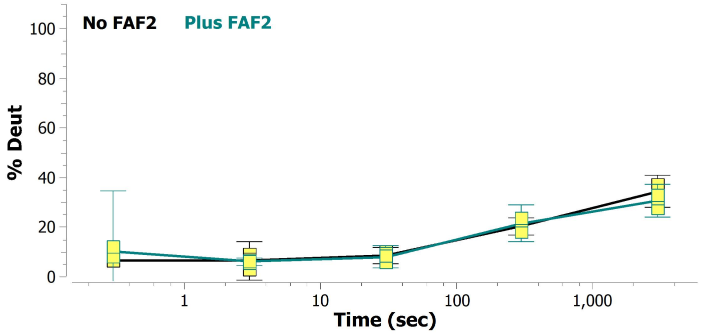

NPL4 436-448: ITQLARPLPVEYL (#106)

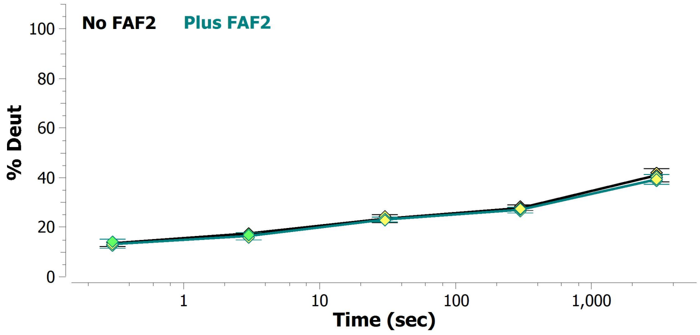

NPL4 439-448: LARPLPVEYL (#107)

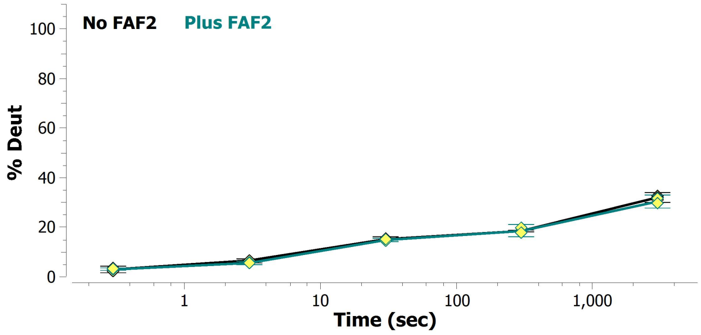

NPL4 446-468: EYLIIDITTTFPKDPVYTFSISQ (#108)

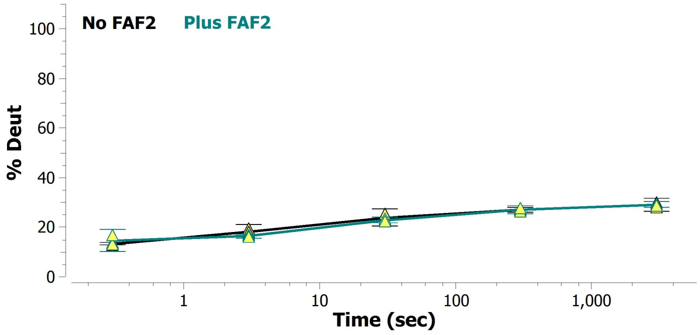

NPL4 449-463: IIDITTTFPKDPVYT (#109)

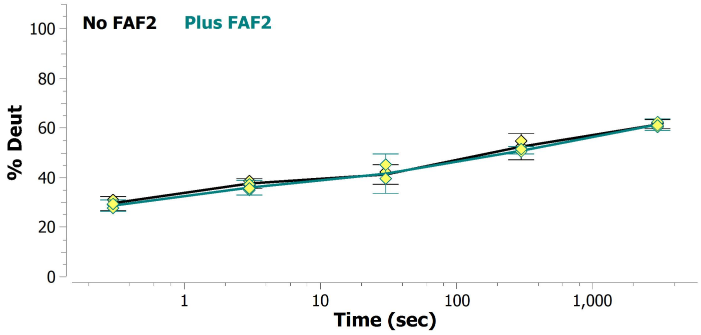

NPL4 452-463: ITTTFPKDPVYT (#110)

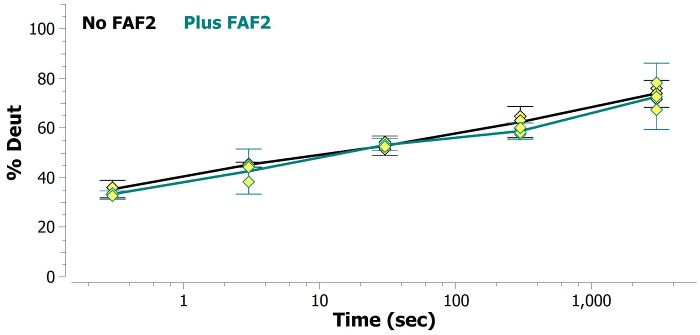

NPL4 458-466: KDPVYTFSI (#111)

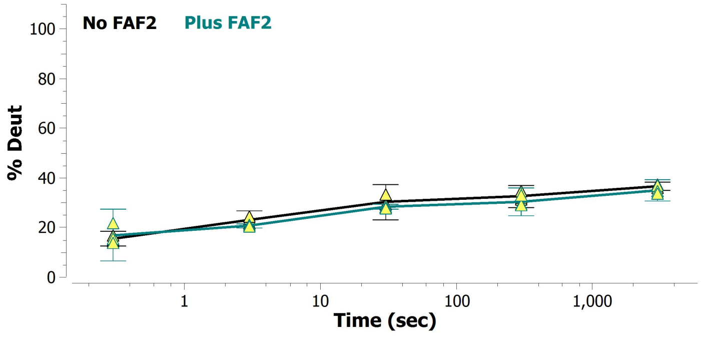

NPL4 464-479: FSISQNPFPIENRDVL (#112)

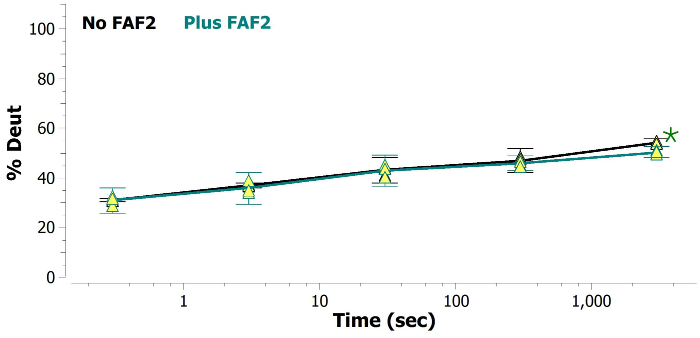

NPL4 513-523: LVTNEVMPLQD (#113)

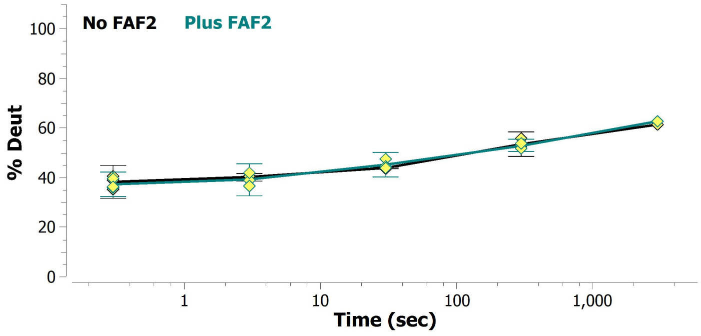

NPL4 523-543: DSISLLLEAVRTRNEELAQTW (#114)

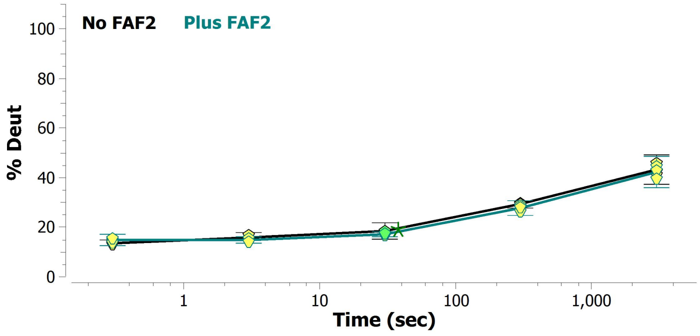

NPL4 529-548: LEAVRTRNEELAQTWKRSEQ (#115)

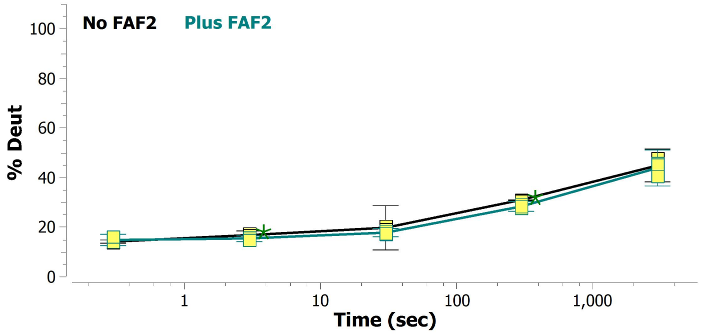

NPL4 549-555: WATIEQL (#116)

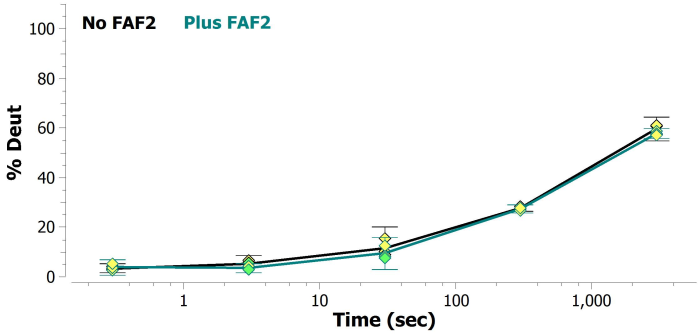

NPL4 549-566: WATIEQLCSTVGGQLPGL (#117)

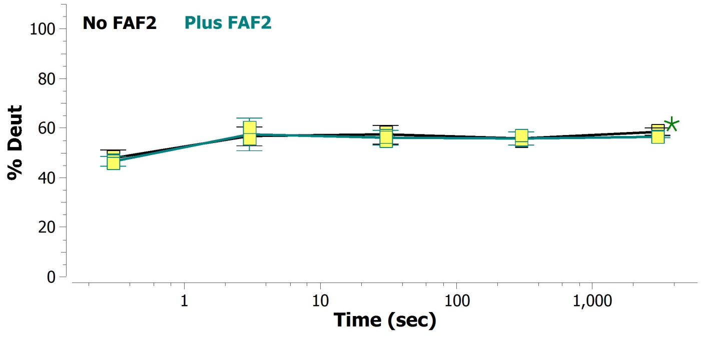

NPL4 556-580: CSTVGGQLPGLHEYGAVGGSTHTAT (#118)

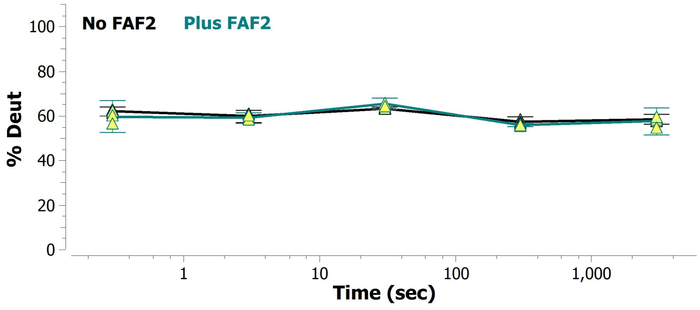

NPL4 556-583: CSTVGGQLPGLHEYGAVGGSTHTATAAM (#119)

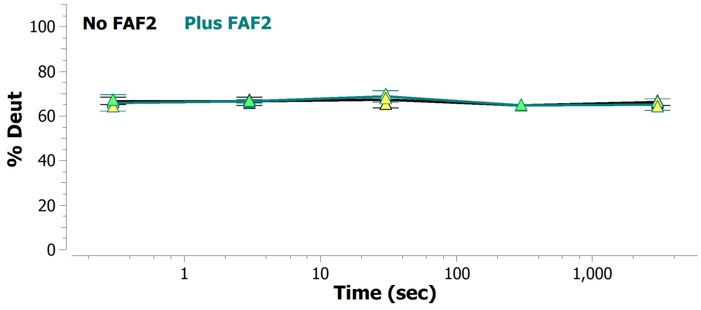

NPL4 564-581: PGLHEYGAVGGSTHTATA (#120)

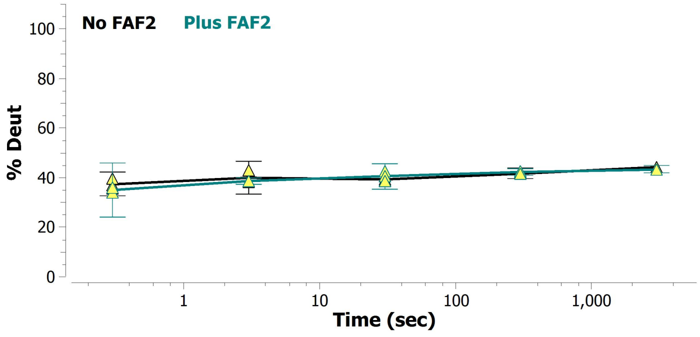

NPL4 584-591: WACQHCTF (#121)

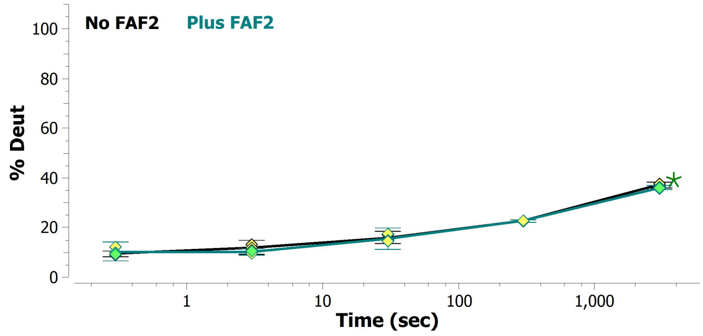

NPL4 592-602: MNQPGTGHCEM (#122)

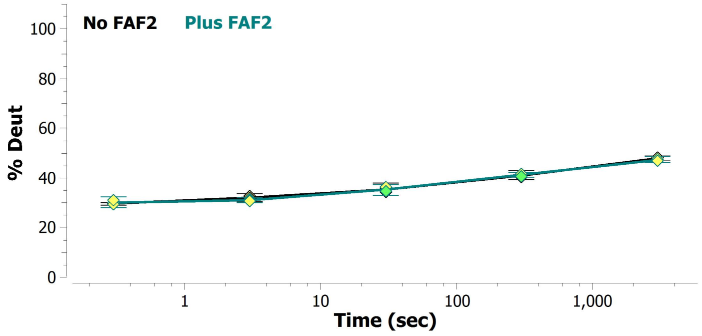

TERA 5-24: ADSKGDDLSTAILKQKNRPN (#123)

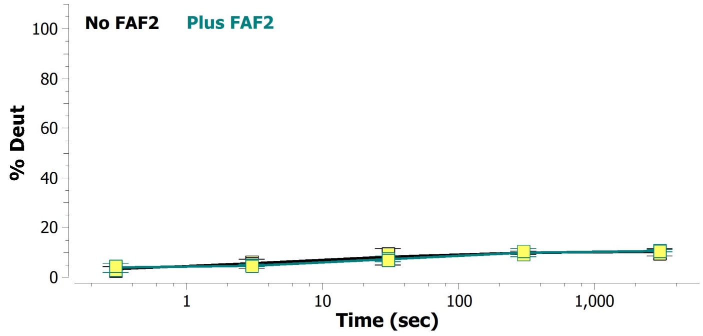

TERA 15-29: AILKQKNRPNRLIVD (#124)

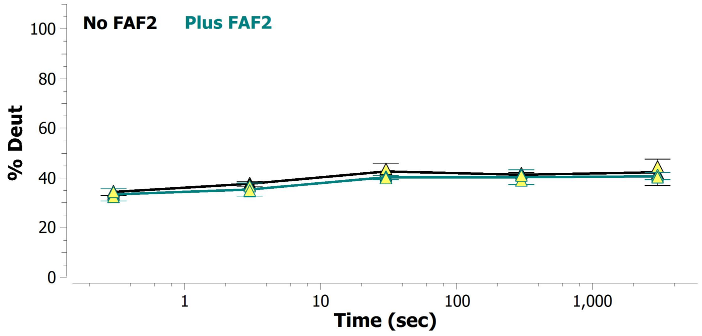

TERA 16-25: ILKQKNRPNR (#125)

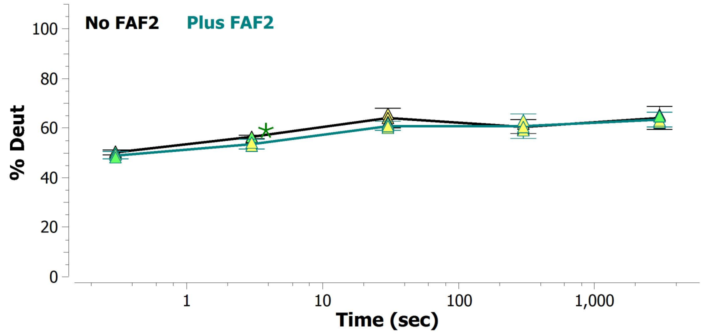

TERA 16-26: ILKQKNRPNRL (#126)

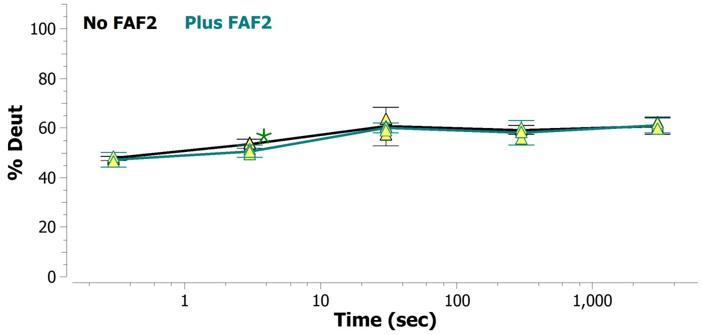

TERA 16-30: ILKQKNRPNRLIVDE (#127)

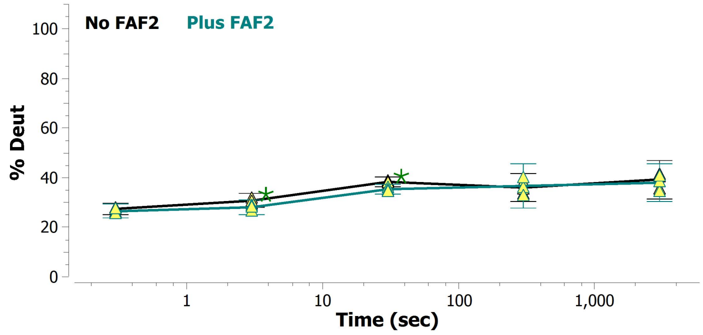

TERA 17-26: LKQKNRPNRL (#128)

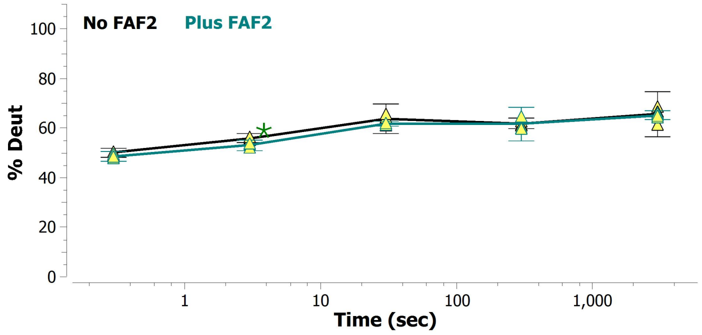

TERA 26-50: LIVDEAINEDNSVVSLSQPKMDELQ (#129)

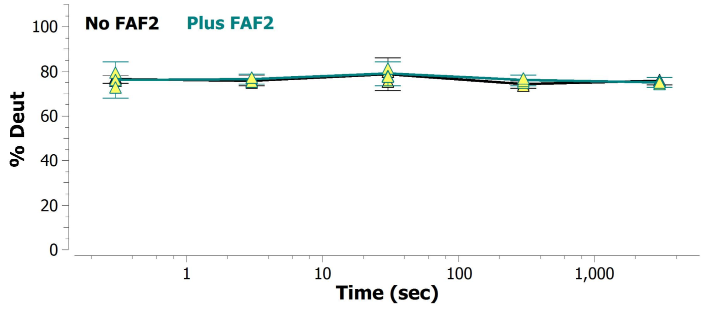

TERA 27-37: IVDEAINEDNS (#130)

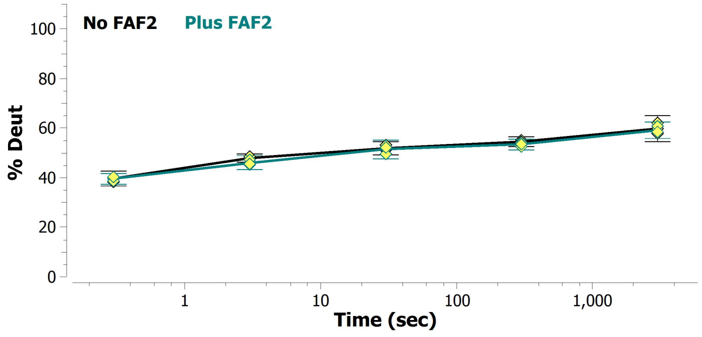

TERA 27-38: IVDEAINEDNSV (#131)

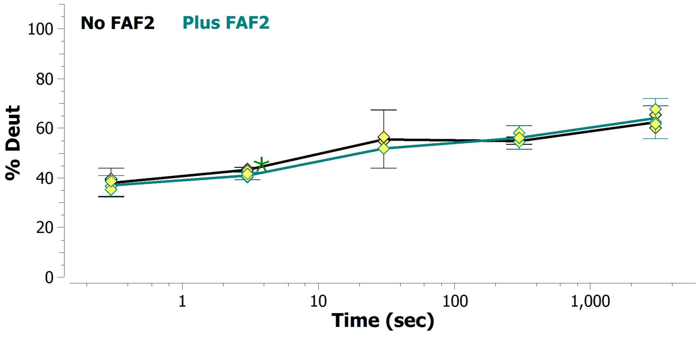

TERA 27-40: IVDEAINEDNSVVS (#132)

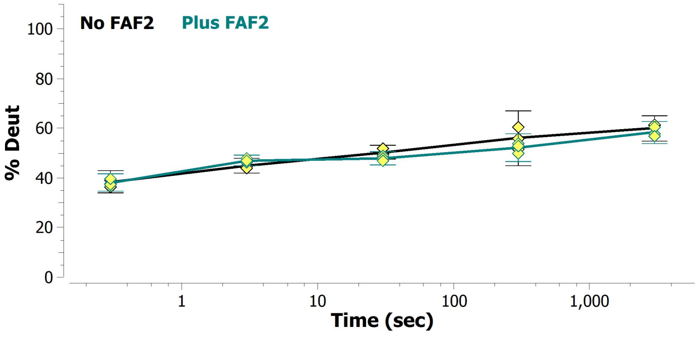

TERA 27-48: IVDEAINEDNSVVSLSQPKMDE (#133)

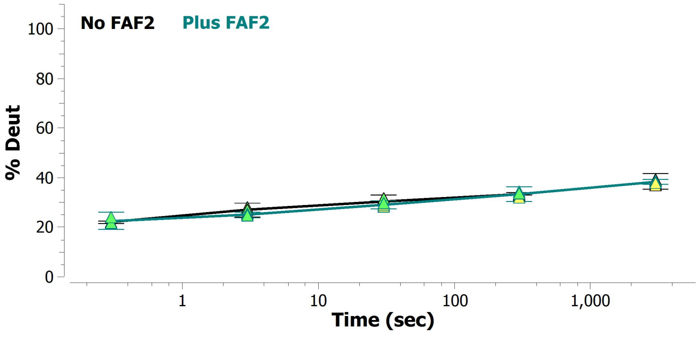

TERA 28-41: VDEAINEDNSVVSL (#134)

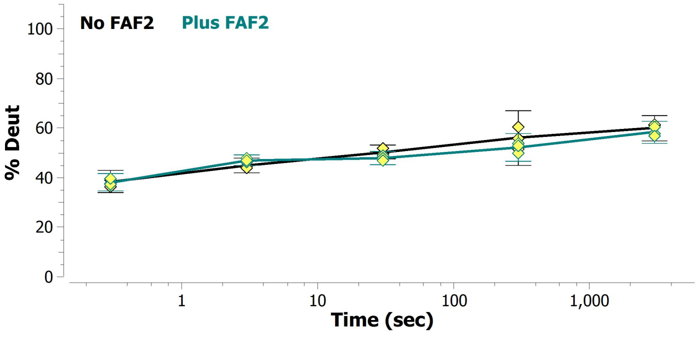

TERA 38-51: VVSLSQPKMDELQL (#135)

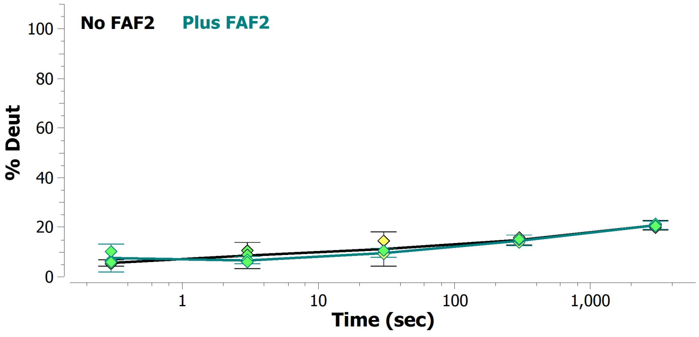

TERA 39-51: VSLSQPKMDELQL (#136)

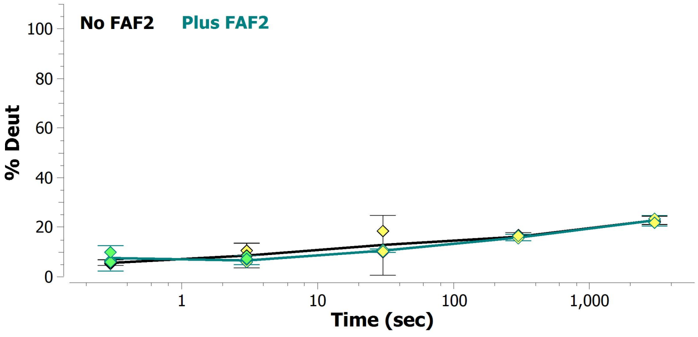

TERA 49-69: LQLFRGDTVLLKGKKRREAVC (#137)

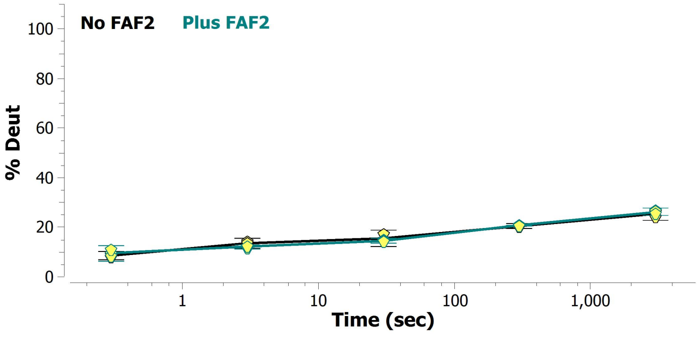

TERA 50-58: QLFRGDTVL (#138)

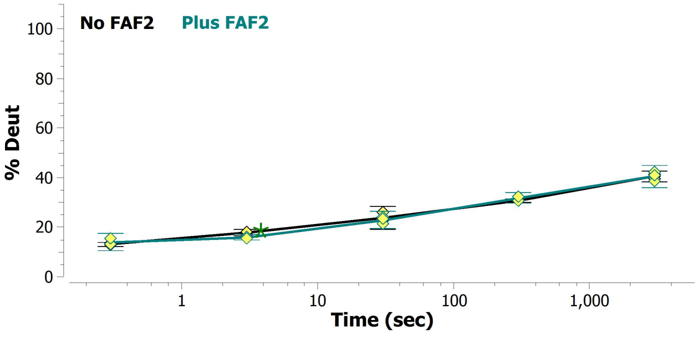

TERA 52-58: FRGDTVL (#139)

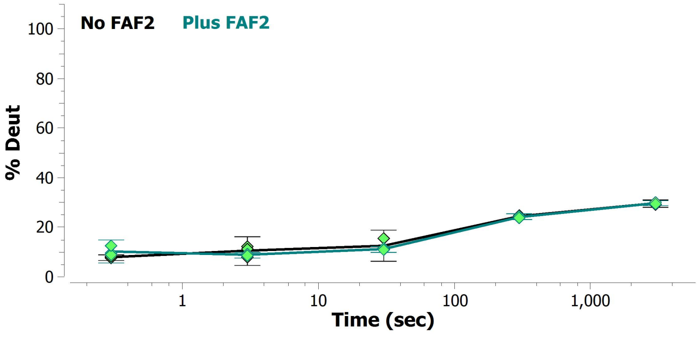

TERA 52-69: FRGDTVLLKGKKRREAVC (#140)

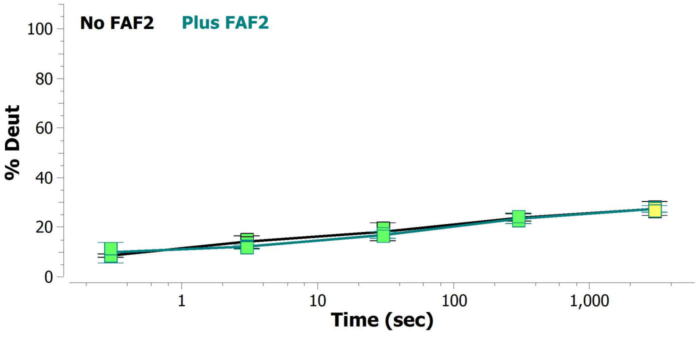

TERA 59-69: LKGKKRREAVC (#141)

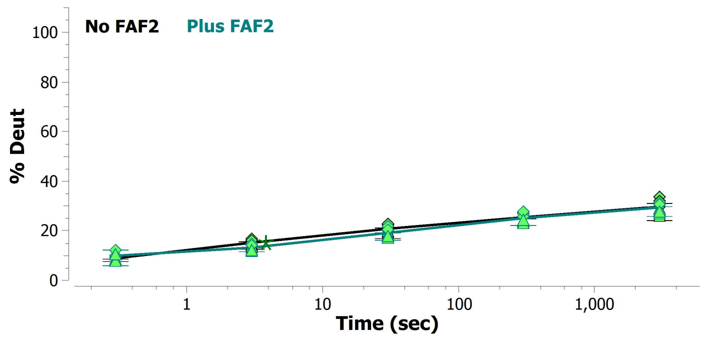

TERA 61-69: GKKRREAVC (#142)

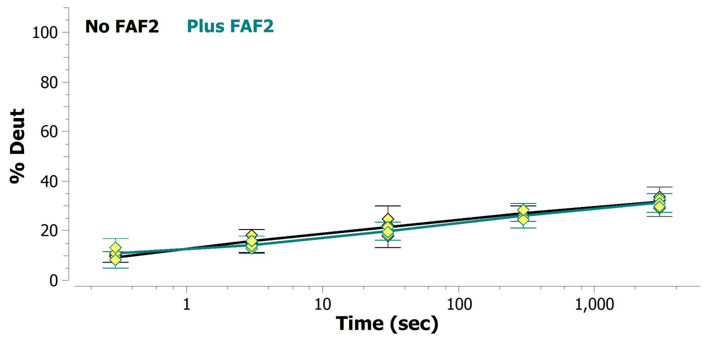

TERA 70-80: IVLSDDTCSDE (#143)

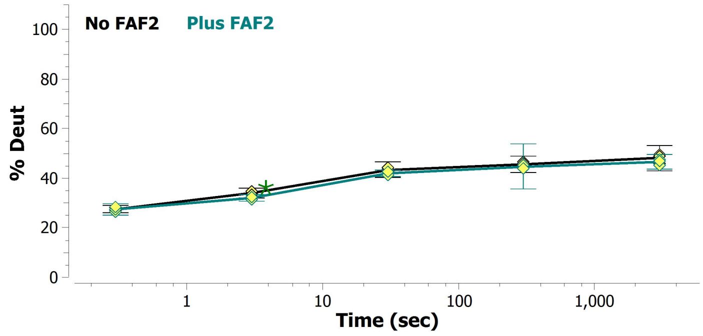

TERA 70-84: IVLSDDTCSDEKIRM (#144)

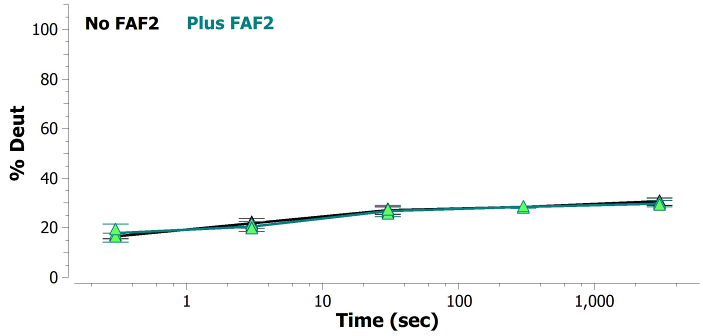

TERA 70-92: IVLSDDTCSDEKIRMNRVVRNNL (#145)

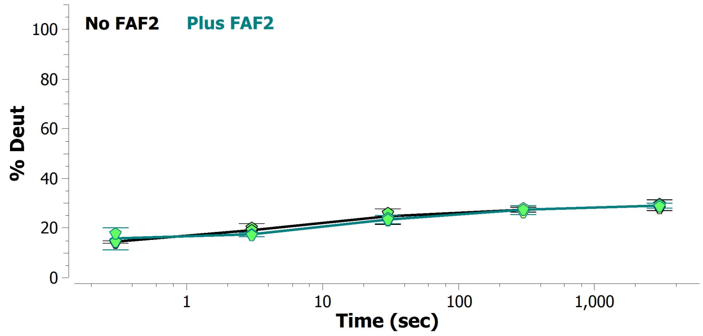

TERA 72-84: LSDDTCSDEKIRM (#146)

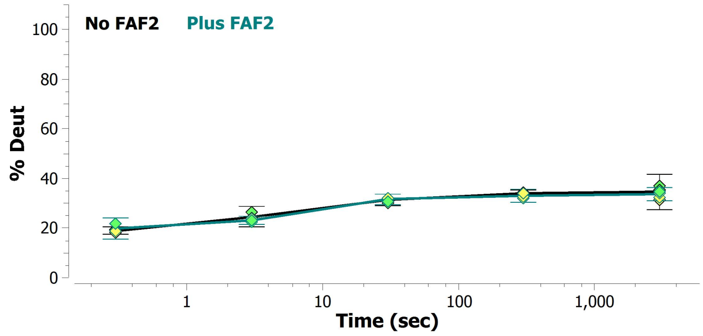

TERA 73-84: SDDTCSDEKIRM (#147)

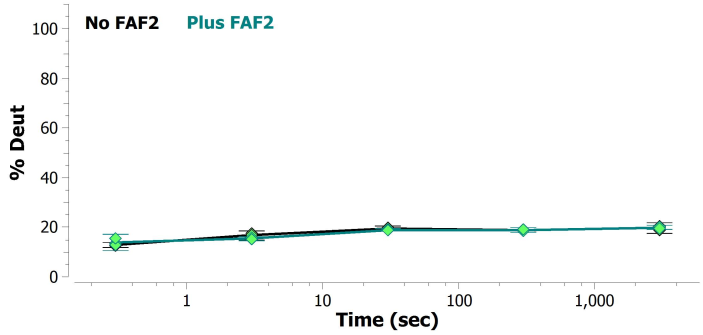

TERA 81-92: KIRMNRVVRNNL (#148)

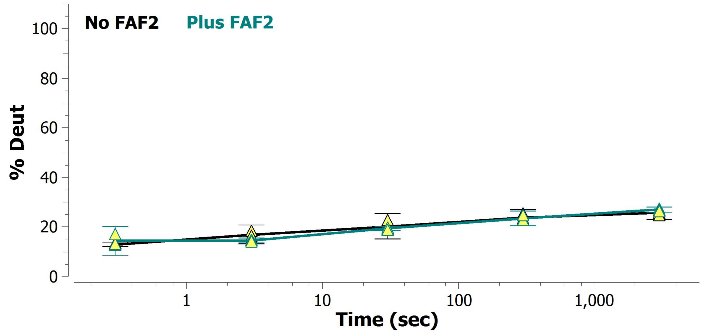

TERA 93-120: RVRLGDVISIQPCPDVKYGKRIHVLPID (#149)

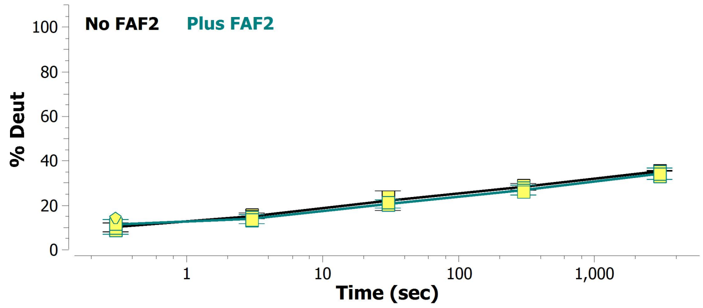

TERA 93-121: RVRLGDVISIQPCPDVKYGKRIHVLPIDD (#150)

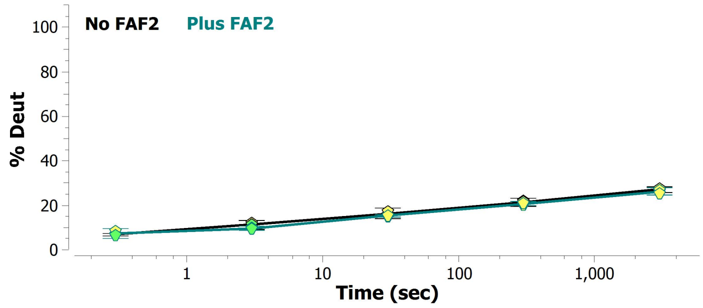

TERA 93-124:
RVRLGDVISIQPCPDVKYGKRIHVLPIDDTVE (#151)

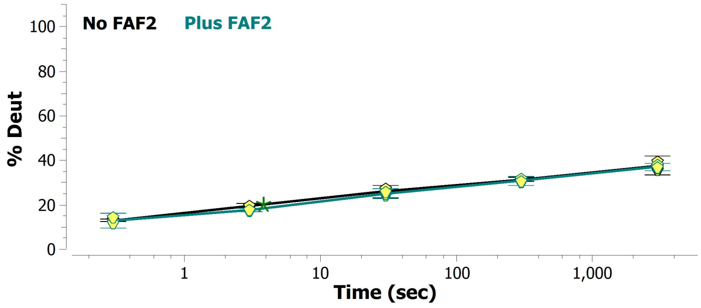

TERA 100-121: ISIQPCPDVKYGKRIHVLPIDD (#152)

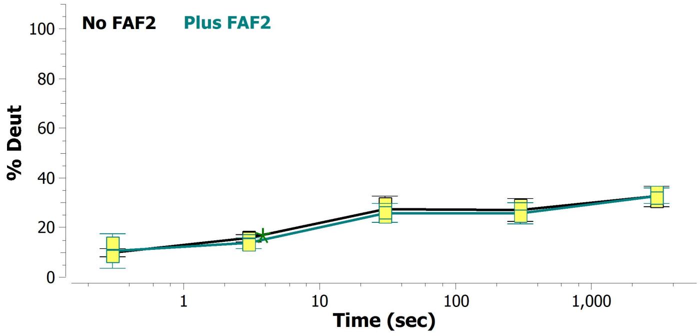

TERA 100-130:
ISIQPCPDVKYGKRIHVLPIDDTVEGITGNL (#153)

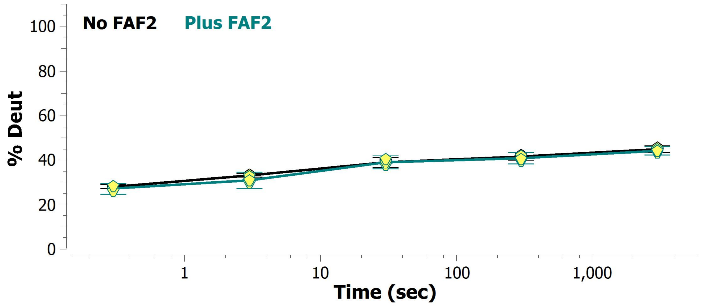

TERA 103-121: QPCPDVKYGKRIHVLPIDD (#154)

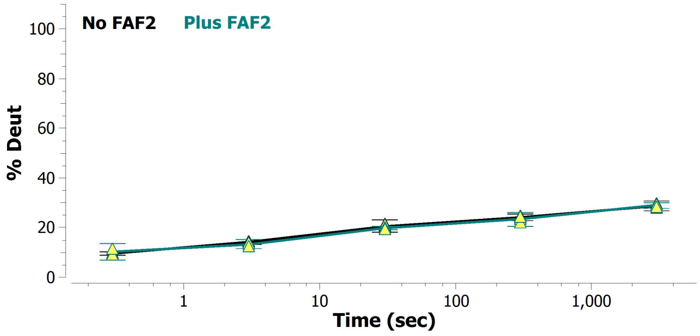

TERA 107-140:
DVKYGKRIHVLPIDDTVEGITGNLFEVYLKPYFL (#155)

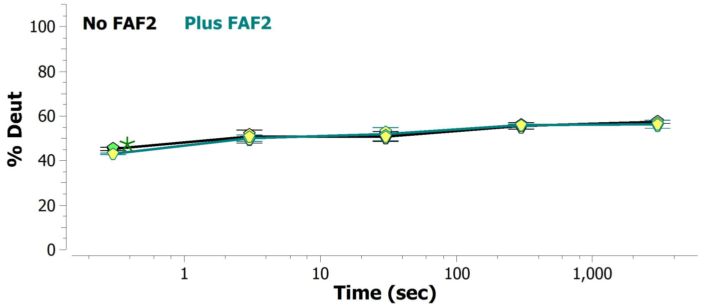

TERA 116-130: VLPIDDTVEGITGNL (#156)

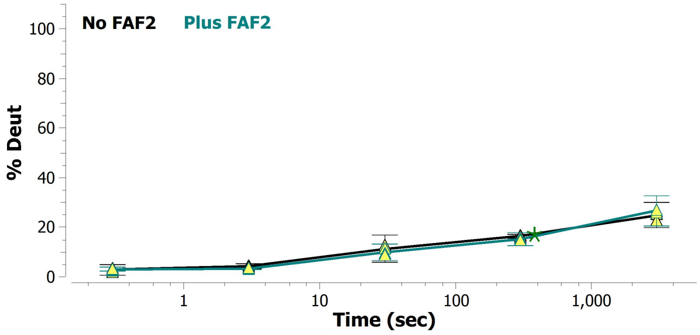

TERA 119-128: IDDTVEGITG (#157)

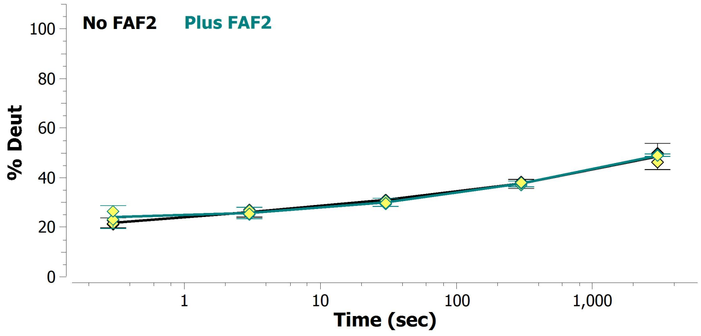

TERA 131-139: FEVYLKPYF (#158)

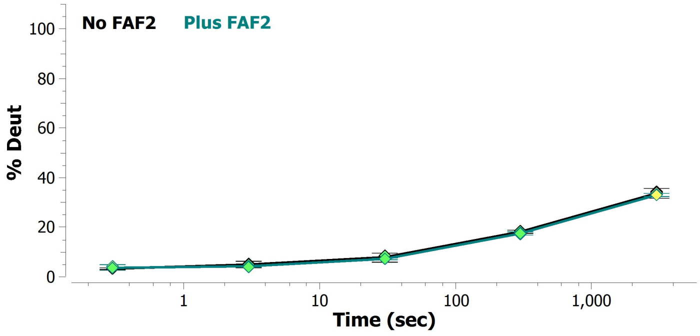

TERA 131-140: FEVYLKPYFL (#159)

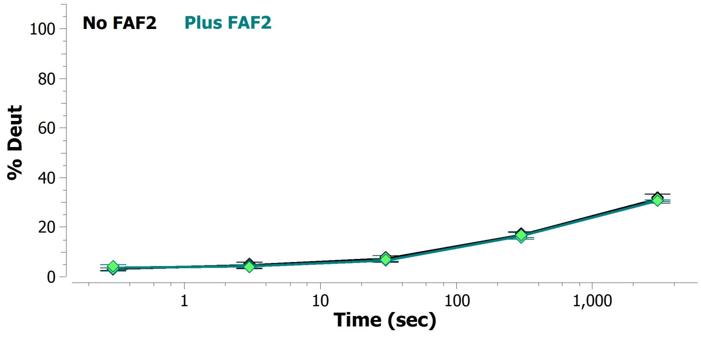

TERA 131-141: FEVYLKPYFLE (#160)

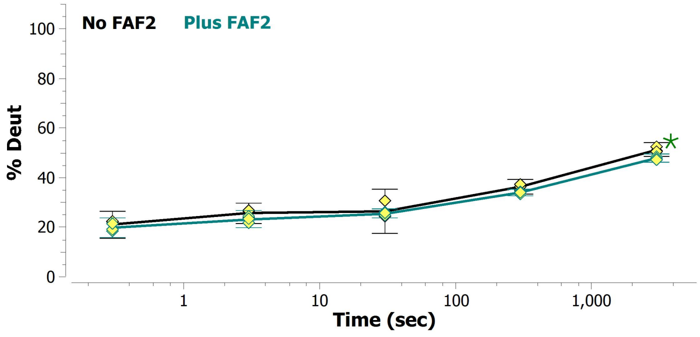

TERA 131-142: FEVYLKPYFLEA (#161)

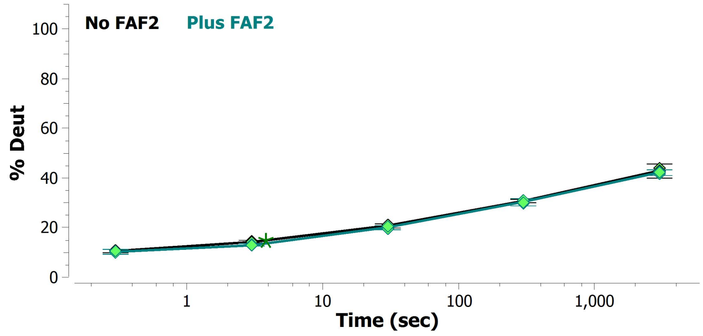

TERA 132-139: EVYLKPYF (#162)

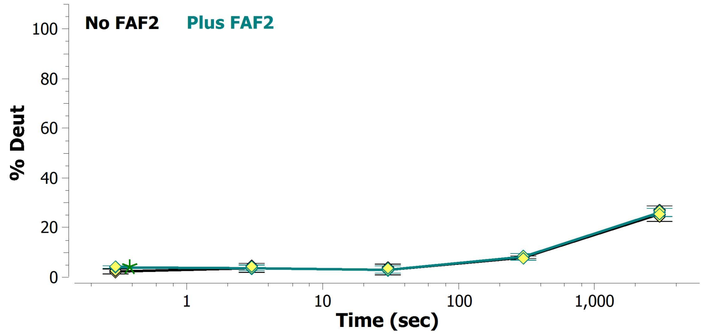

TERA 132-140: EVYLKPYFL (#163)

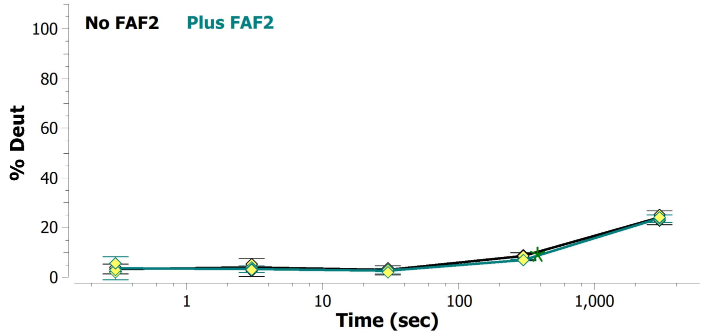

TERA 132-142: EVYLKPYFLEA (#164)

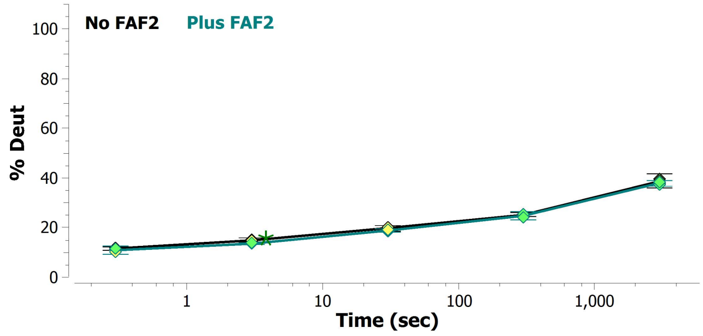

TERA 133-139: VYLKPYF (#165)

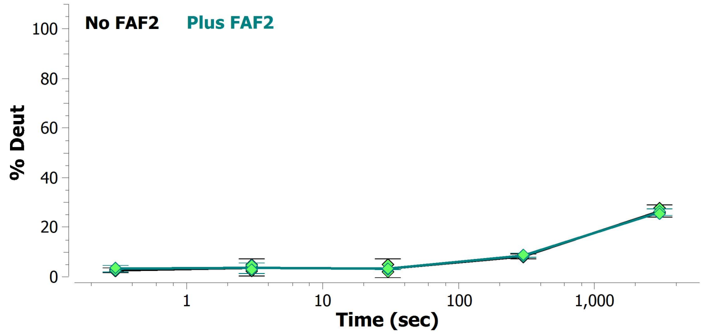

TERA 133-140: VYLKPYFL (#166)

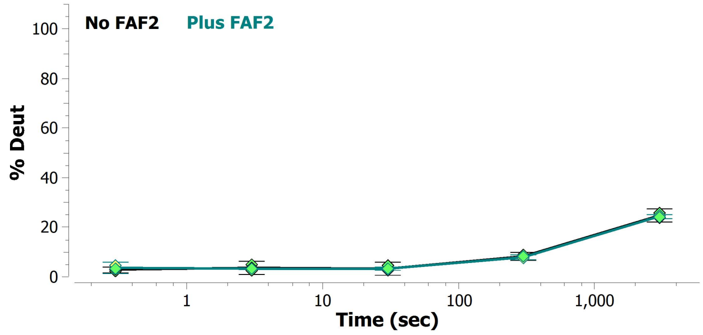

TERA 133-142: VYLKPYFLEA (#167)

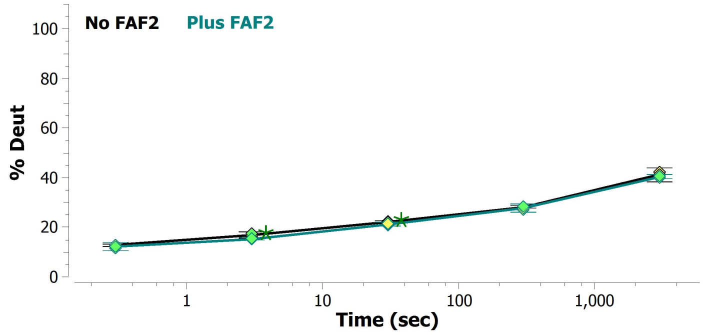

TERA 134-142: YLKPYFLEA (#168)

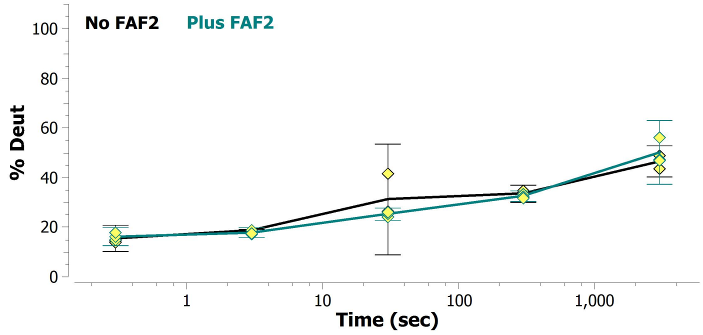

TERA 139-150: FLEAYRPIRKGD (#169)

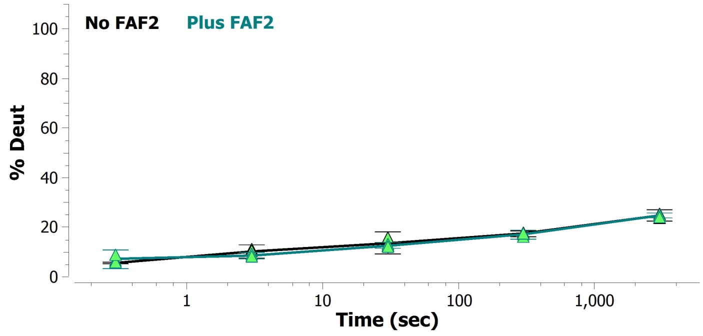

TERA 139-151: FLEAYRPIRKGDI (#170)

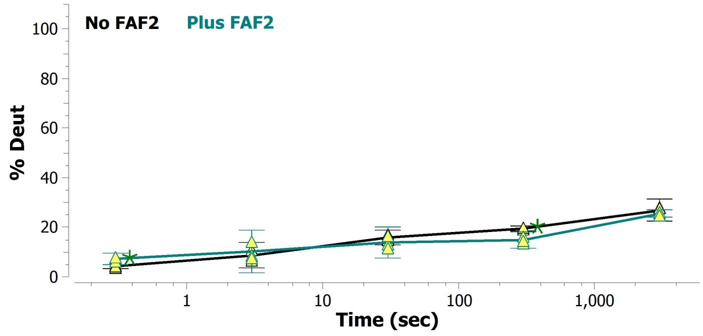

TERA 140-152: LEAYRPIRKGDIF (#171)

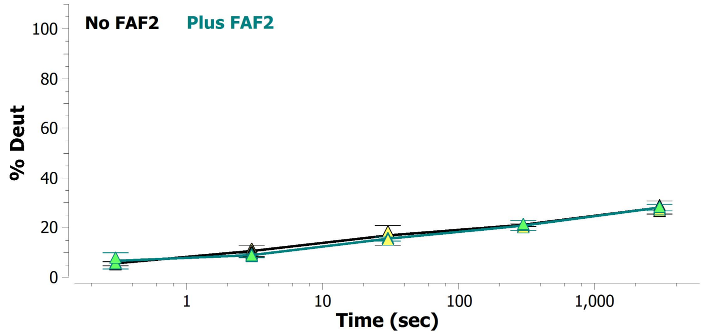

TERA 140-153: LEAYRPIRKGDIFL (#172)

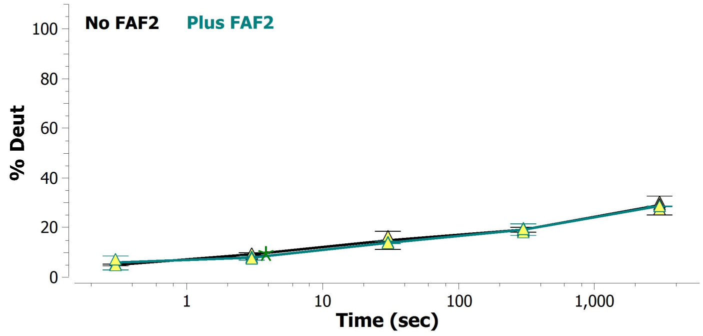

TERA 141-152: EAYRPIRKGDIF (#173)

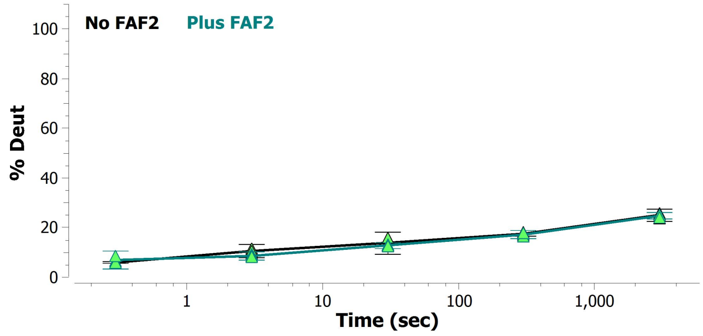

TERA 141-153: EAYRPIRKGDIFL (#174)

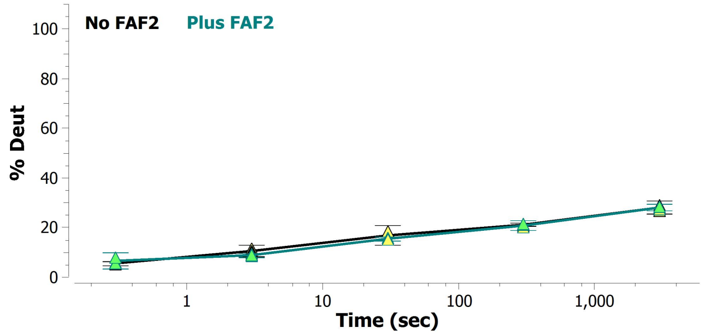

TERA 143-152: YRPIRKGDIF (#175)

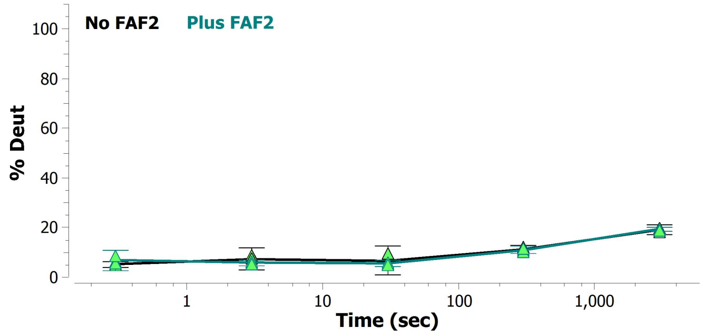

TERA 143-153: YRPIRKGDIFL (#176)

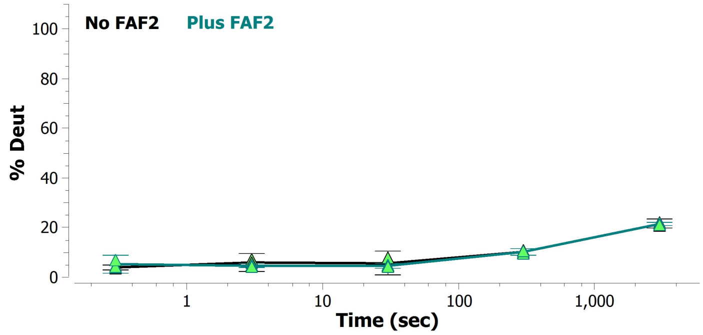

TERA 145-152: PIRKGDIF (#177)

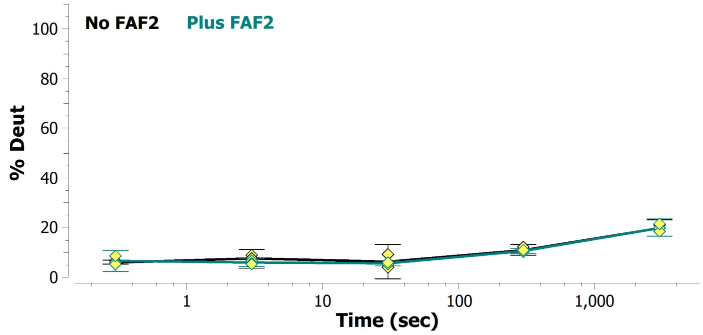

TERA 153-162: LVRGGMRAVE (#178)

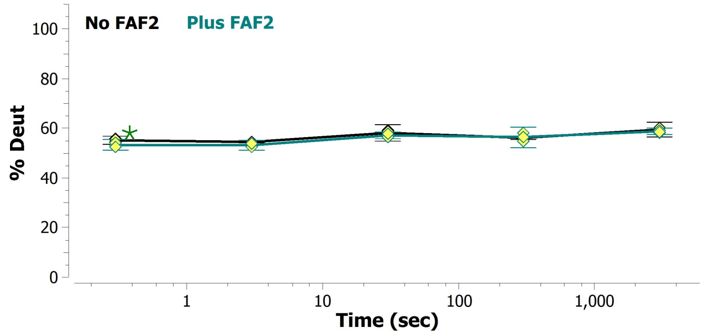

TERA 153-163: LVRGGMRAVEF (#179)

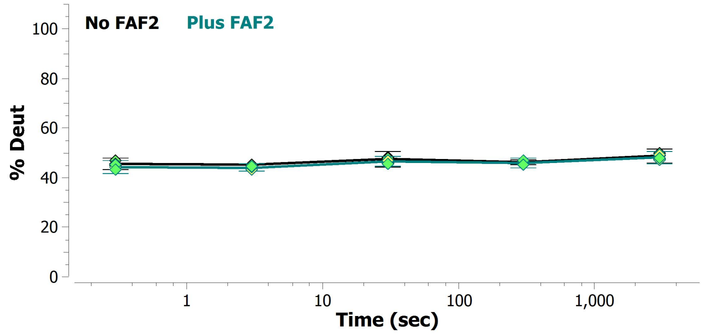

TERA 155-166: RGGMRAVEFKVV (#180)

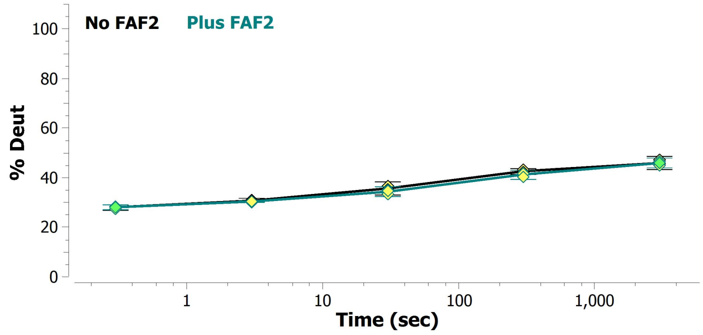

TERA 161-183: VEFKVVETDPSPYCIVAPDTVIH (#181)

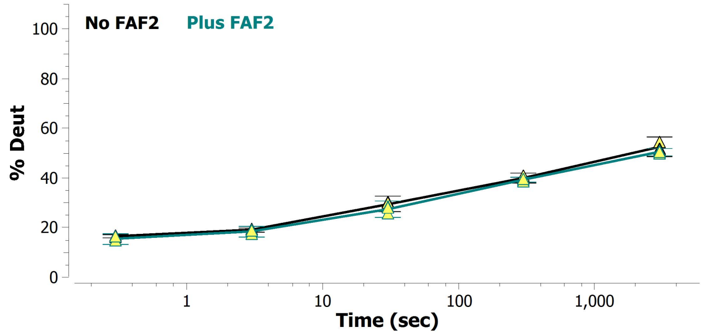

TERA 163-174: FKVVETDPSPYC (#182)

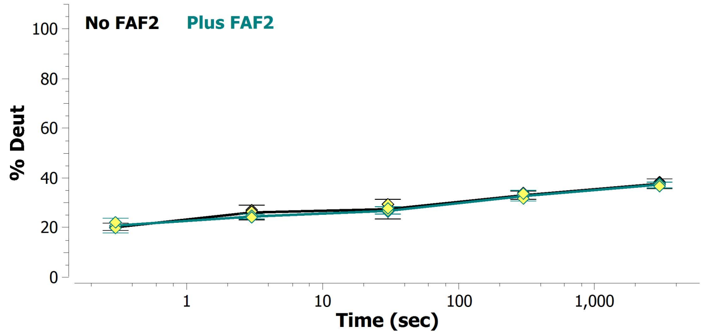

TERA 163-184: FKVVETDPSPYCIVAPDTVIHC (#183)

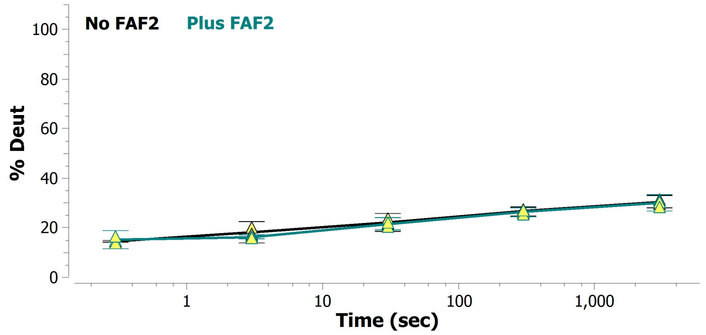

TERA 164-174: KVVETDPSPYC (#184)

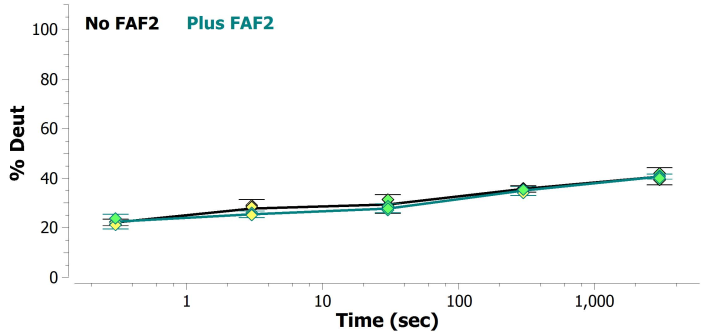

TERA 164-177: KVVETDPSPYCIVA (#185)

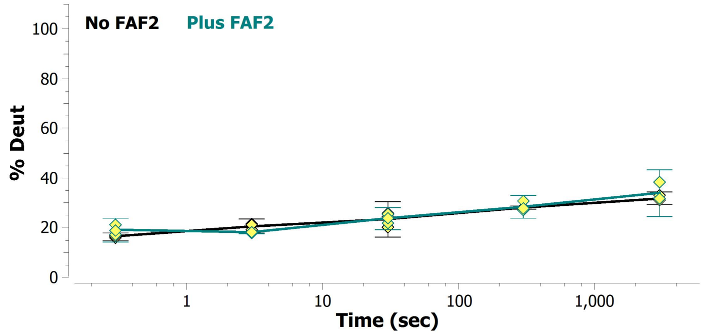

TERA 164-195: KVVETDPSPYCIVAPDTVIHCEGEPIKREDEE (#186)

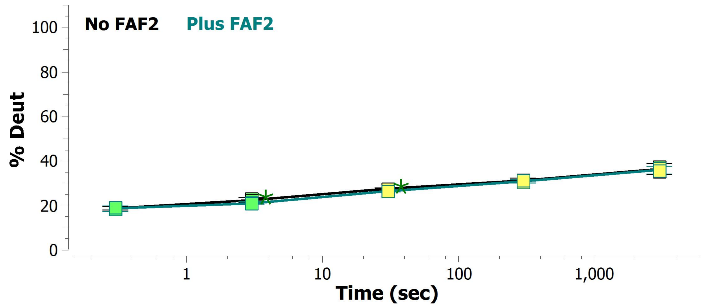

TERA 175-195: IVAPDTVIHCEGEPIKREDEE (#187)

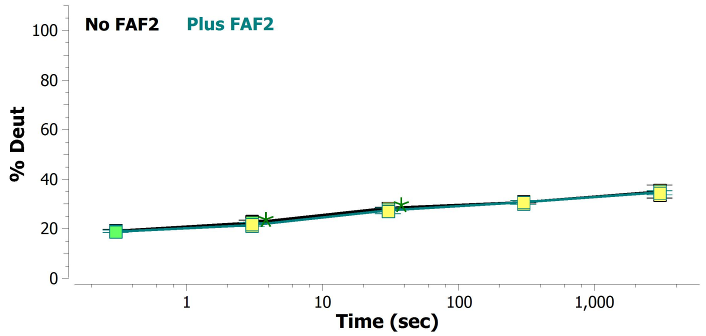

TERA 175-196: IVAPDTVIHCEGEPIKREDEEE (#188)

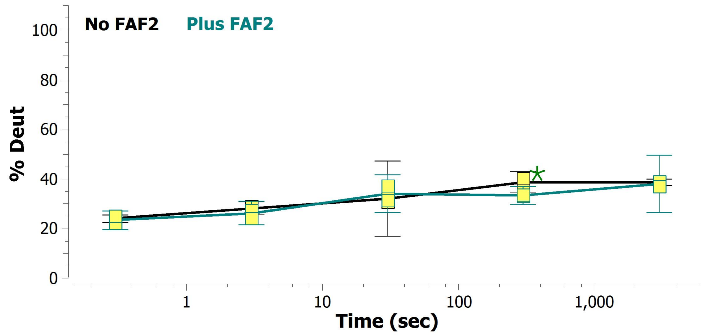

TERA 175-198: IVAPDTVIHCEGEPIKREDEEESL (#189)

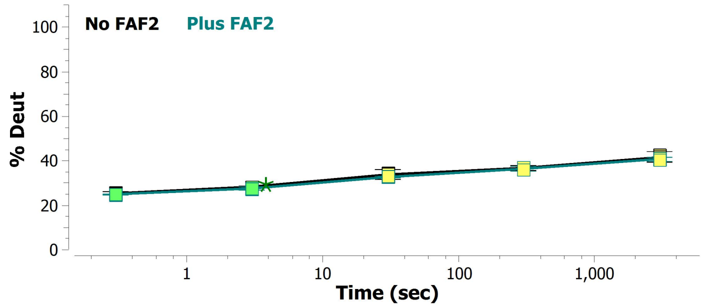

TERA 175-200: IVAPDTVIHCEGEPIKREDEEESLNE (#190)

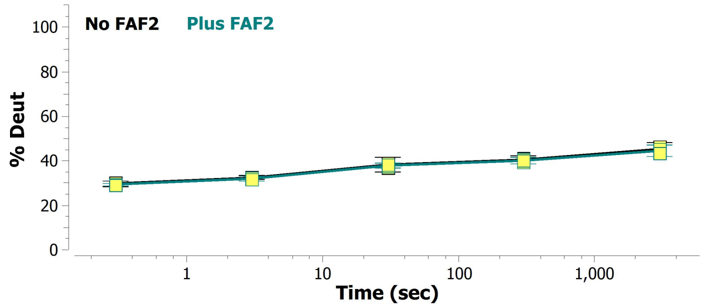

TERA 177-198: APDTVIHCEGEPIKREDEEESL (#191)

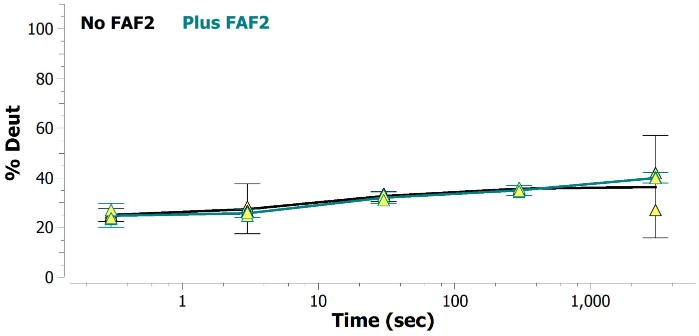

TERA 178-184: PDTVIHC (#192)

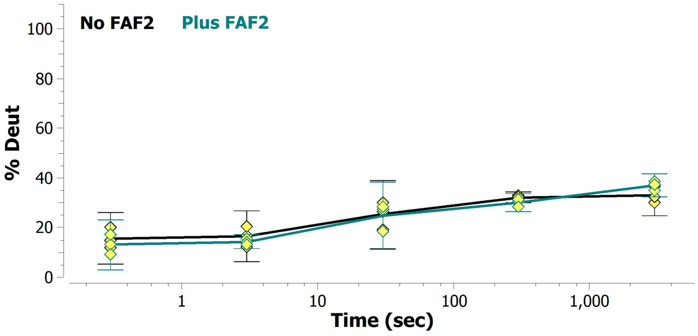

TERA 178-200: PDTVIHCEGEPIKREDEEESLNE (#193)

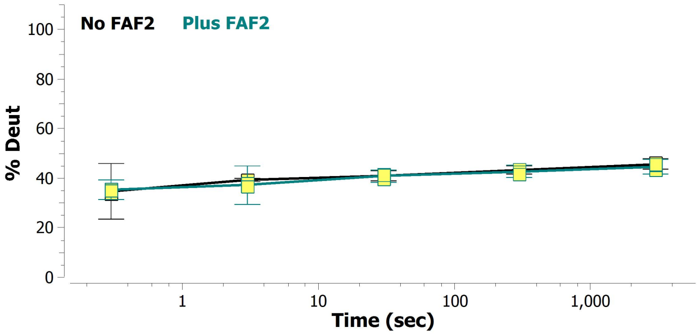

TERA 187-193: EPIKRED (#194)

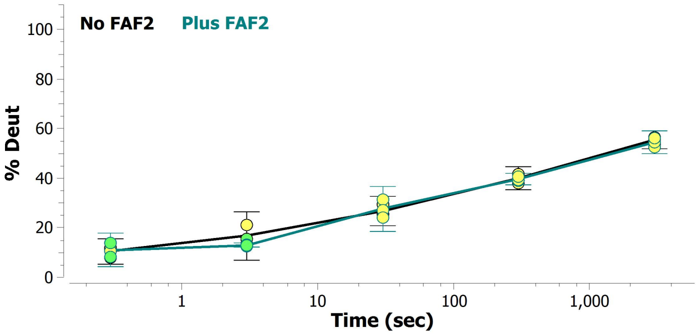

TERA 196-218: ESLNEVGYDDIGGCRKQLAQIKE (#195)

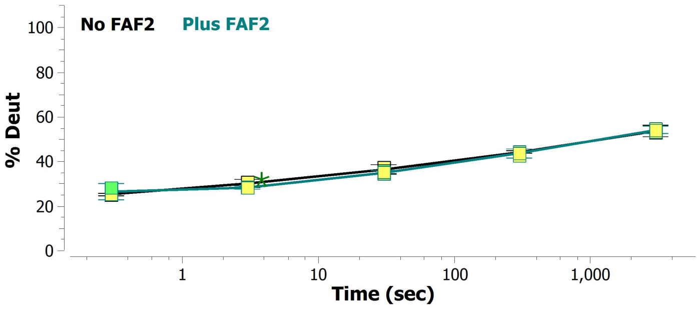

TERA 199-215: NEVGYDDIGGCRKQLAQ (#196)

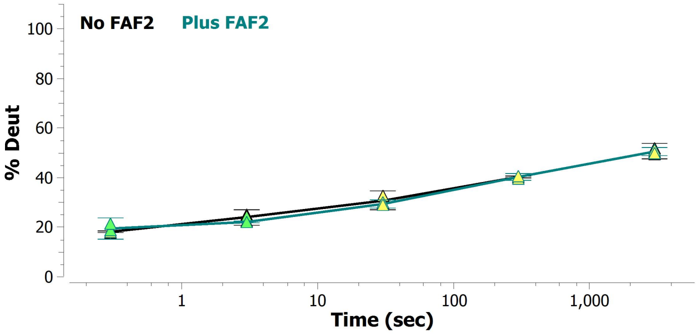

TERA 199-218: NEVGYDDIGGCRKQLAQIKE (#197)

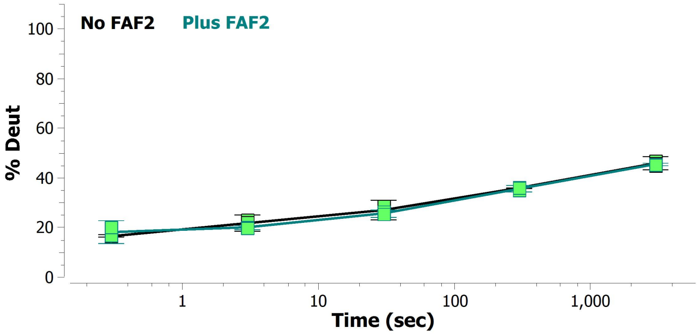

TERA 199-219: NEVGYDDIGGCRKQLAQIKEM (#198)

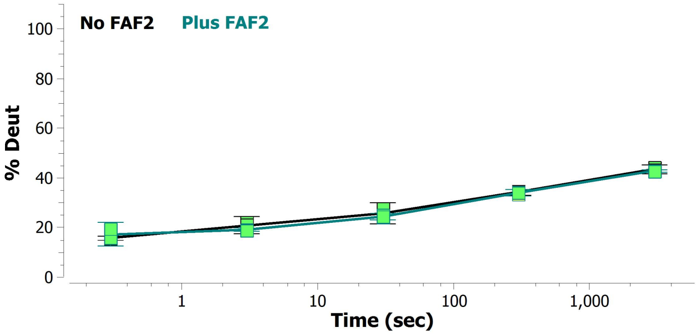

TERA 200-217: EVGYDDIGGCRKQLAQIK (#199)

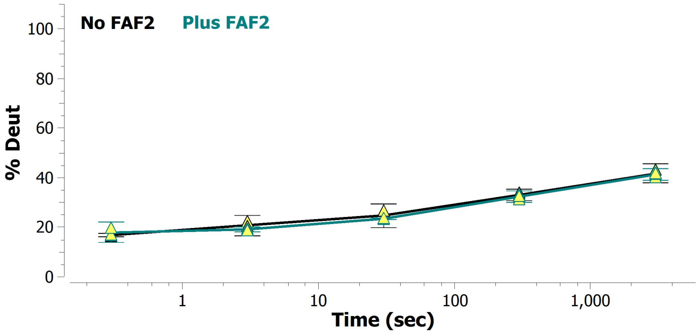

TERA 201-215: VGYDDIGGCRKQLAQ (#200)

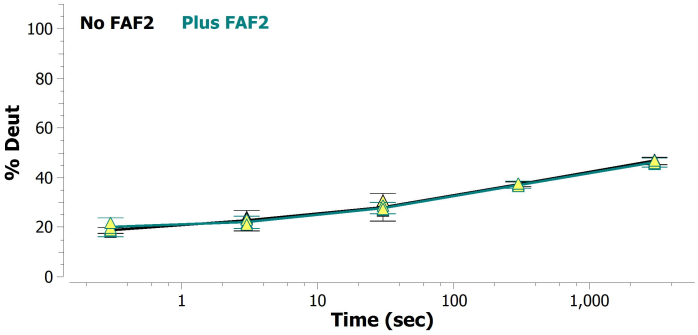

TERA 201-218: VGYDDIGGCRKQLAQIKE (#201)

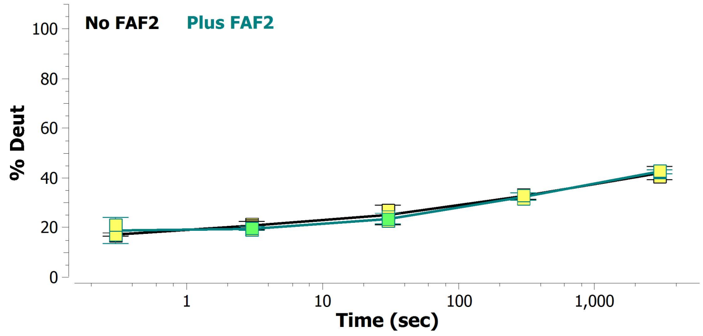

TERA 202-218: GYDDIGGCRKQLAQIKE (#202)

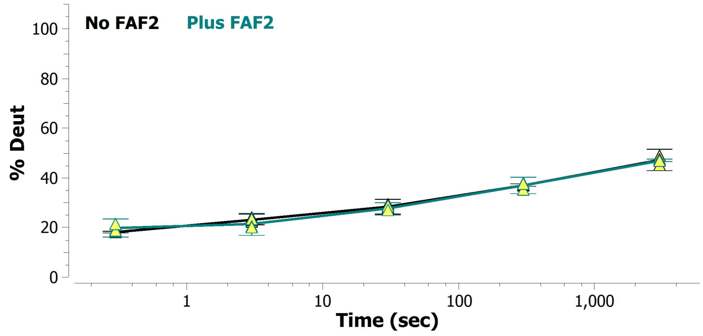

TERA 213-228: LAQIKEMVELPLRHPA (#203)

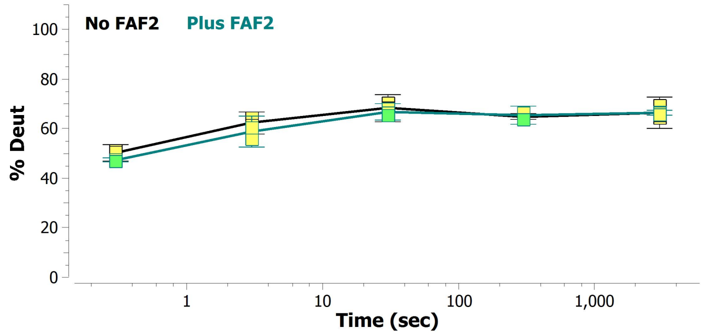

TERA 219-229: MVELPLRHPAL (#204)

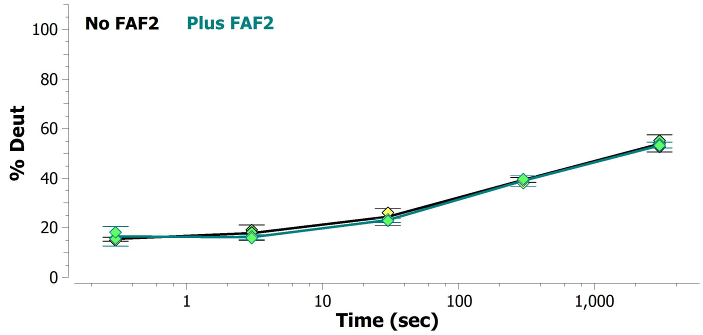

TERA 219-230: MVELPLRHPALF (#205)

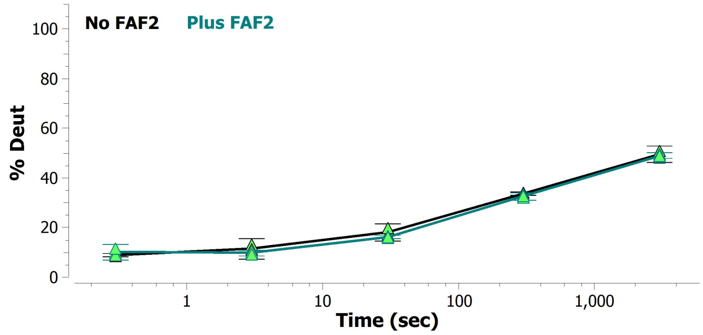

TERA 219-242: MVELPLRHPALFKAIGVKPPRGIL (#206)

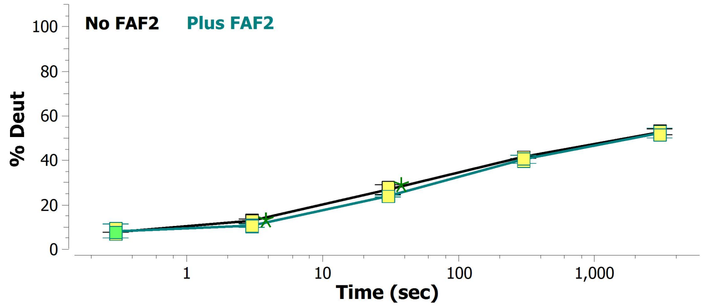

TERA 219-243: MVELPLRHPALFKAIGVKPPRGILL (#207)

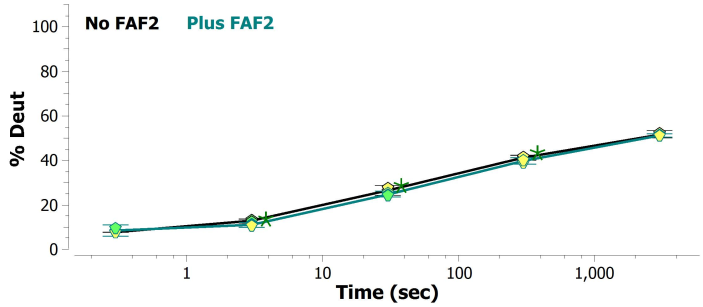

TERA 220-229: VELPLRHPAL (#208)

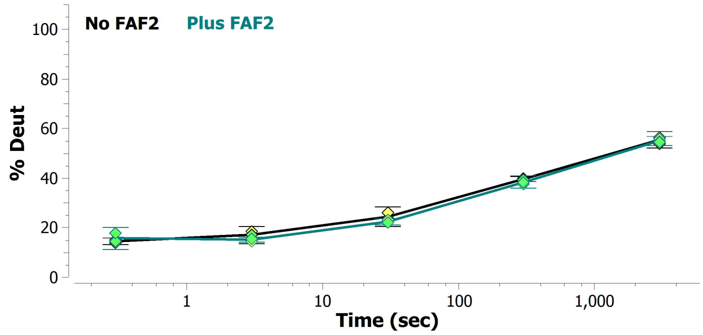

TERA 220-230: VELPLRHPALF (#209)

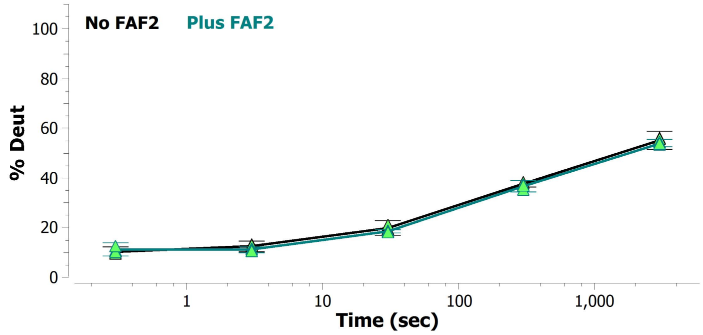

TERA 220-242: VELPLRHPALFKAIGVKPPRGIL (#210)

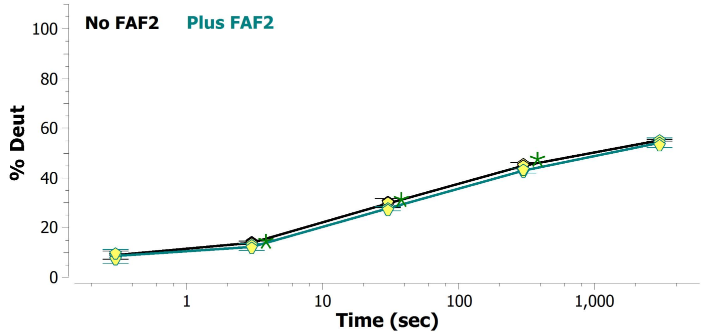

TERA 221-229: ELPLRHPAL (#211)

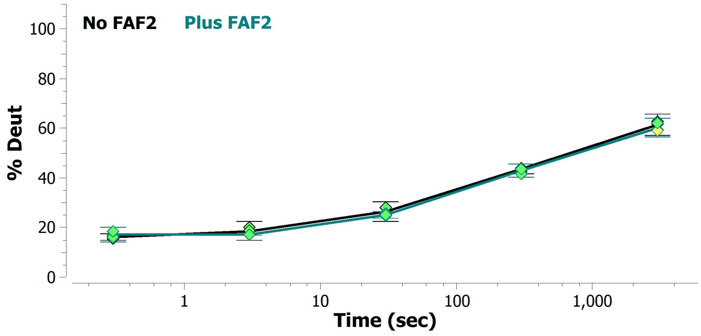

TERA 221-230: ELPLRHPALF (#212)

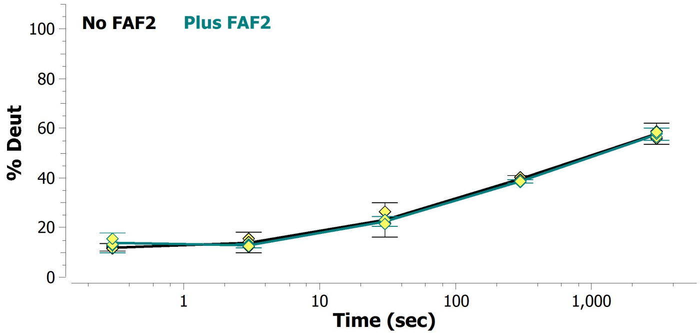

TERA 222-228: LPLRHPA (#213)

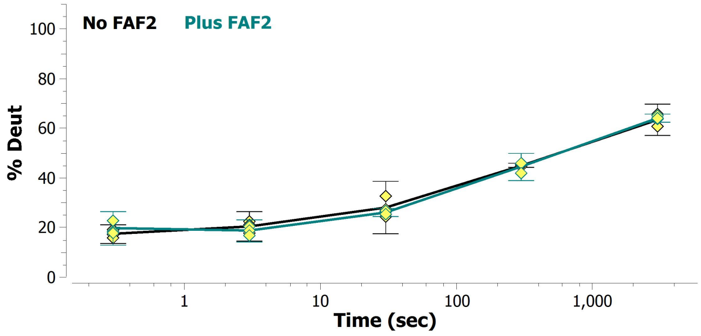

TERA 222-229: LPLRHPAL (#214)

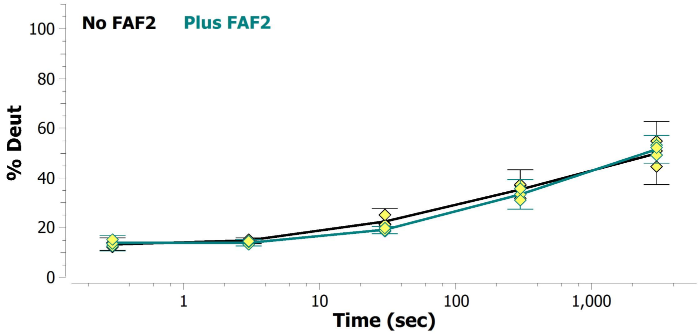

TERA 230-242: FKAIGVKPPRGIL (#215)

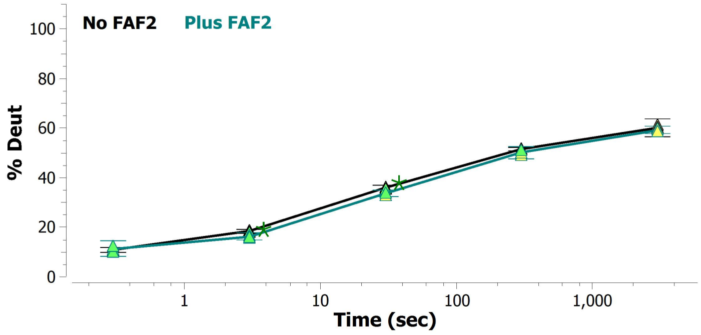

TERA 230-243: FKAIGVKPPRGILL (#216)

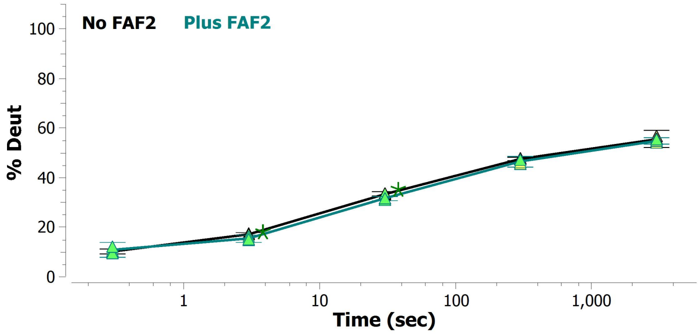

TERA 230-255: FKAIGVKPPRGILLYGPPGTGKTLIA (#217)

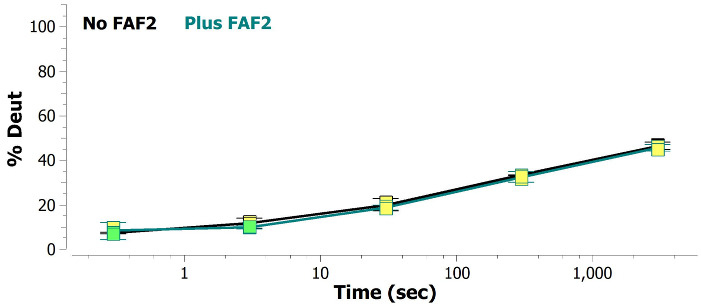

TERA 231-242: KAIGVKPPRGIL (#218)

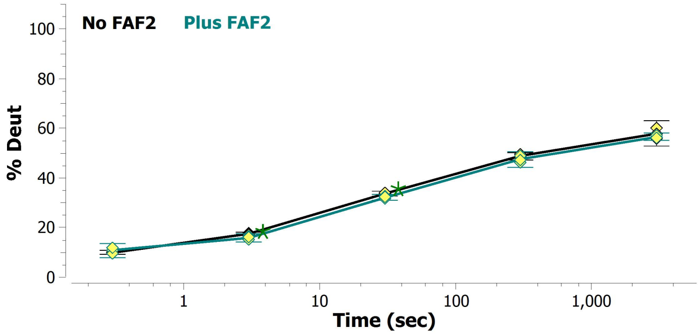

TERA 231-243: KAIGVKPPRGILL (#219)

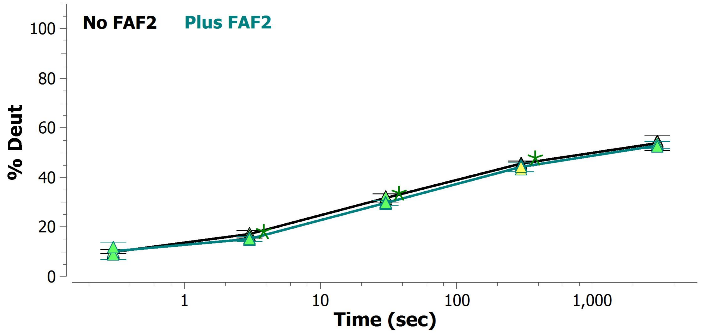

TERA 234-242: GVKPPRGIL (#220)

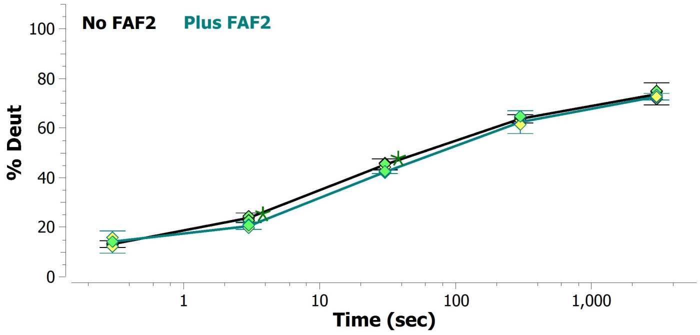

TERA 237-242: PPRGIL (#221)

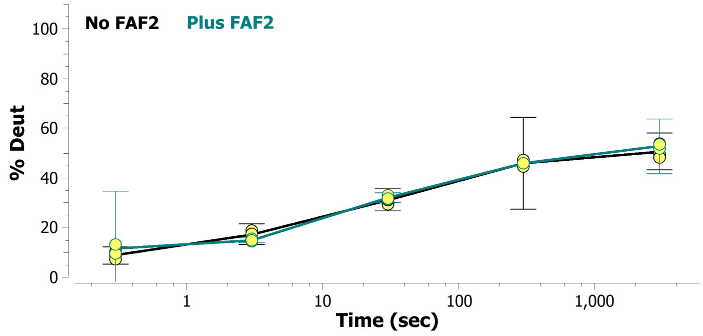

TERA 243-253: LYGPPGTGKTL (#222)

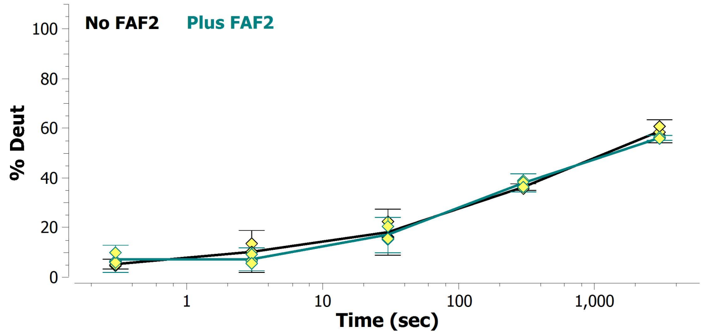

TERA 243-255: LYGPPGTGKTLIA (#223)

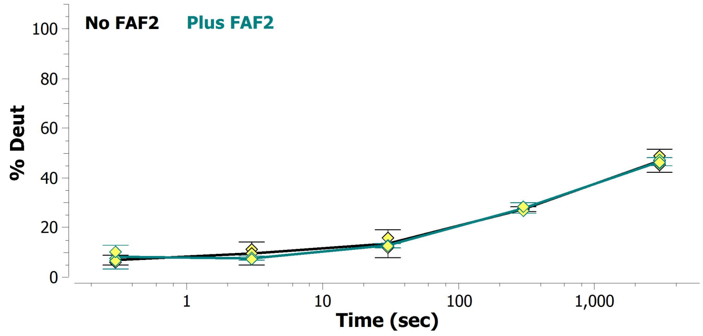

TERA 243-257: LYGPPGTGKTLIARA (#224)

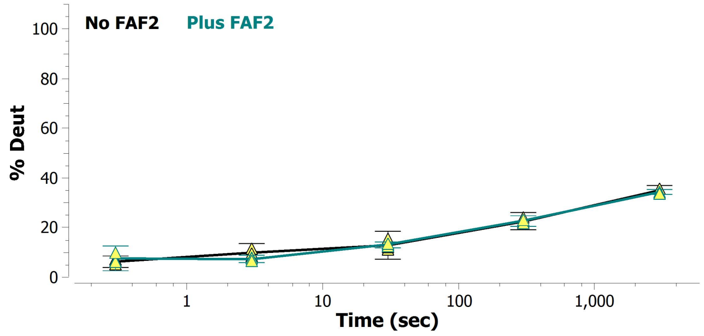

TERA 244-255: YGPPGTGKTLIA (#225)

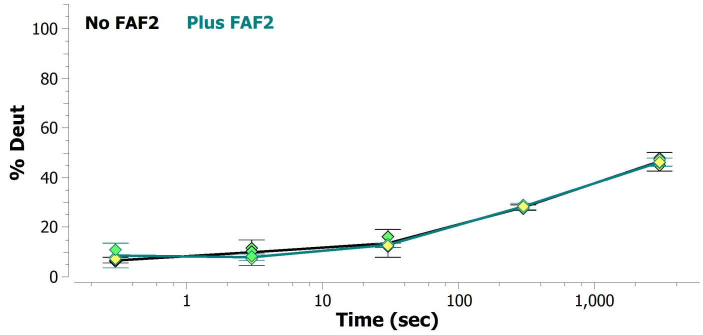

TERA 244-257: YGPPGTGKTLIARA (#226)

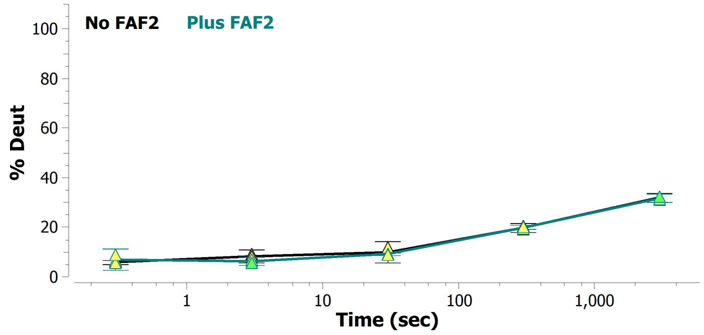

TERA 245-260: GPPGTGKTLIARAVAN (#227)

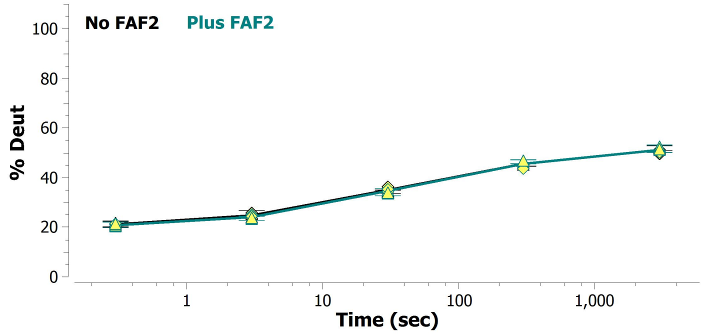

TERA 256-266: RAVANETGAFF (#228)

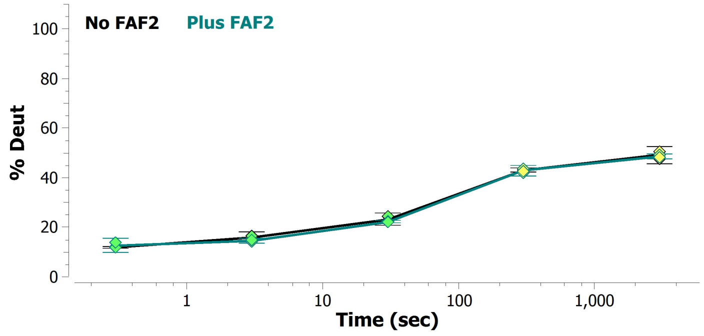

TERA 256-269: RAVANETGAFFFLI (#229)

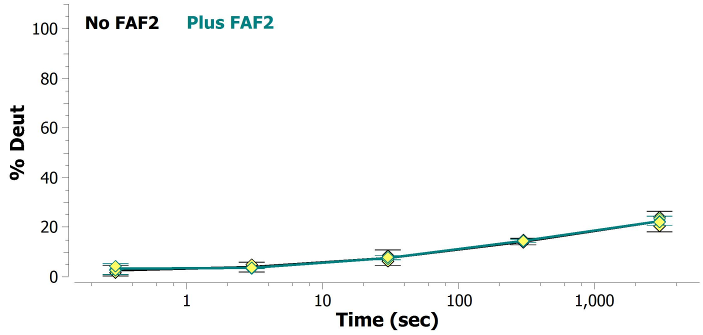

TERA 258-270: VANETGAFFFLIN (#230)

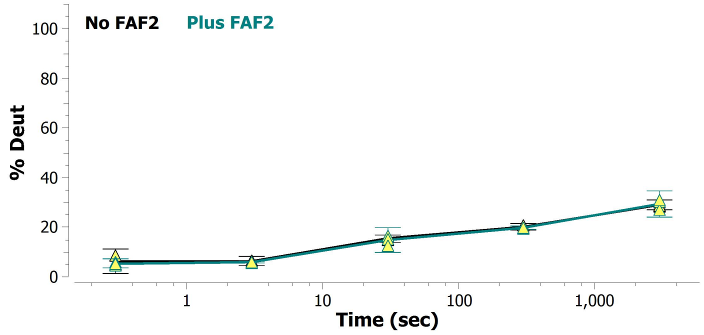

TERA 261-281: ETGAFFFLINGPEIMSKLAGE (#231)

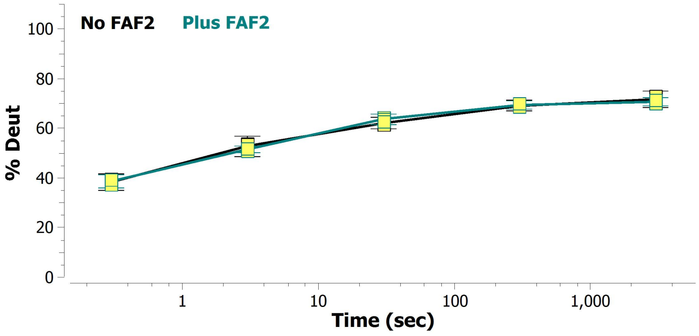

TERA 266-275: FFLINGPEIM (#232)

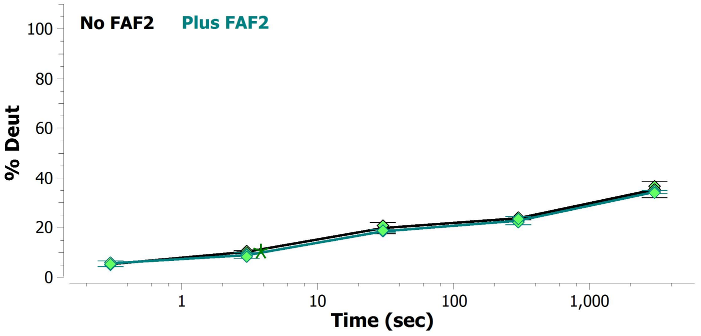

TERA 267-275: FLINGPEIM (#233)

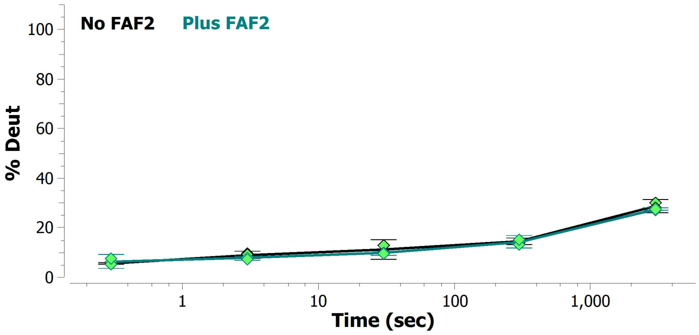

TERA 270-275: NGPEIM (#234)

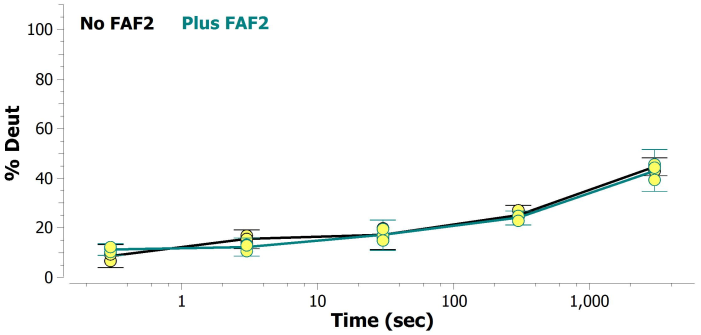

TERA 276-302: SKLAGESESNLRKAFEEAEKNAPAIIF (#235)

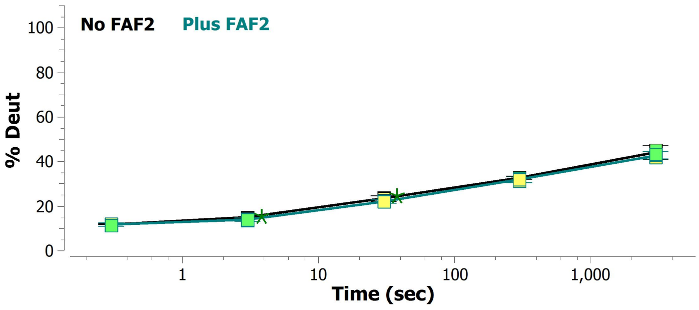

TERA 279-302: AGESESNLRKAFEEAEKNAPAIIF (#236)

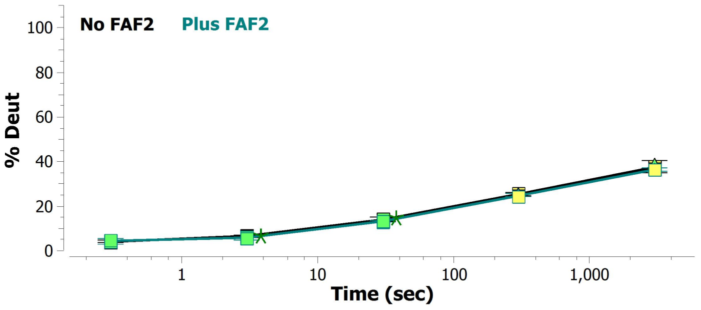

TERA 283-301: ESNLRKAFEEAEKNAPAII (#237)

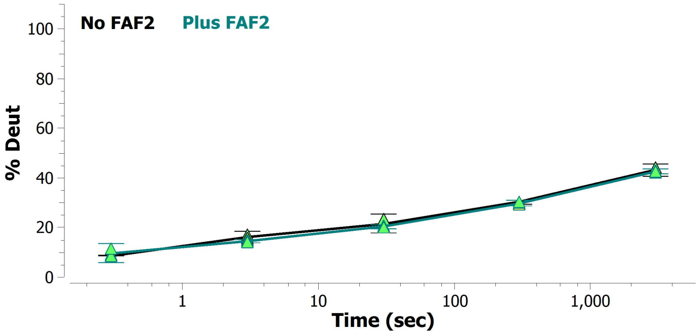

TERA 287-302: RKAFEEAEKNAPAIIF (#238)

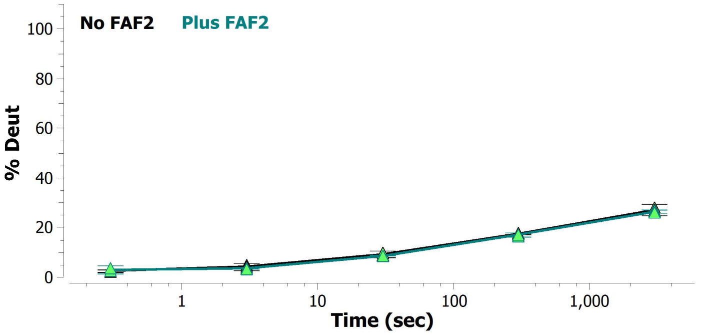

TERA 287-305: RKAFEEAEKNAPAIIFIDE (#239)

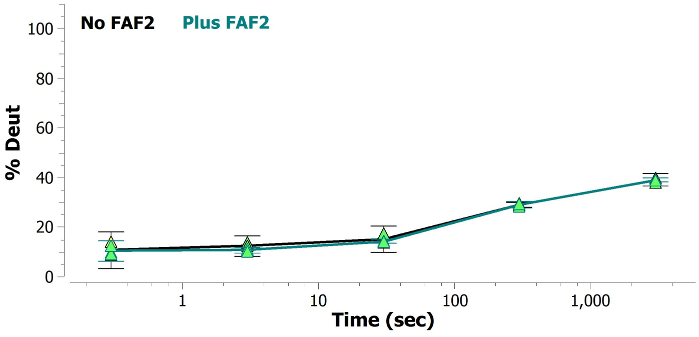

TERA 291-302: EEAEKNAPAIIF (#240)

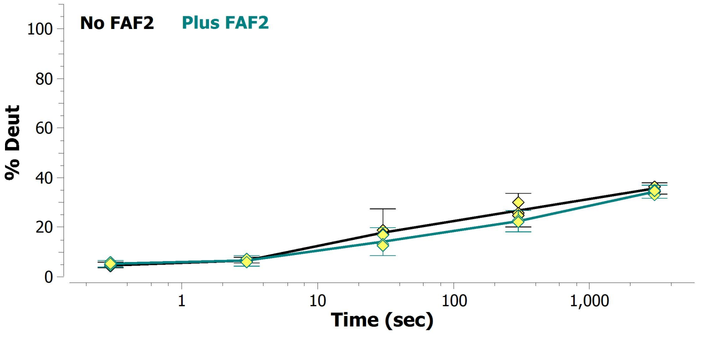

TERA 292-302: EAEKNAPAIIF (#241)

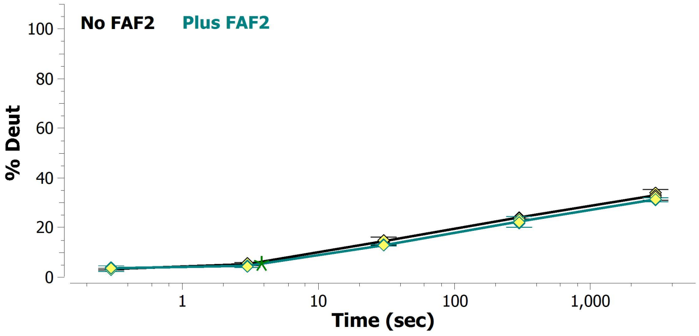

TERA 303-321: IDELDAIAPKREKTHGEVE (#242)

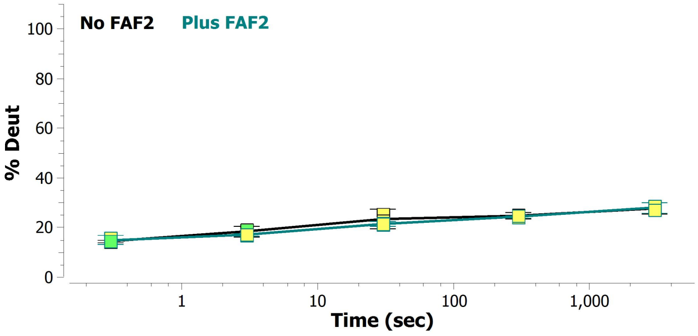

TERA 303-328: IDELDAIAPKREKTHGEVERRIVSQL (#243)

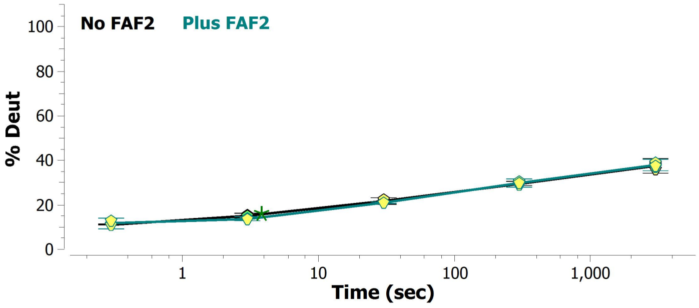

TERA 304-318: DELDAIAPKREKTHG (#244)

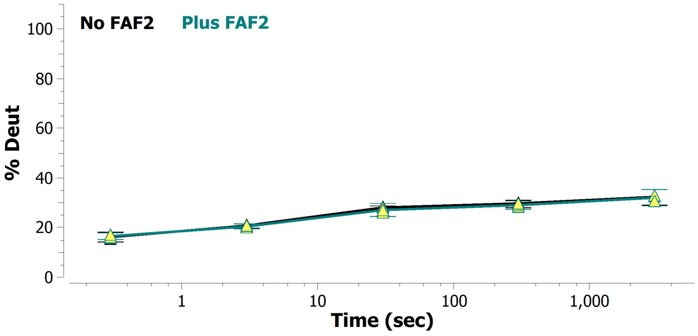

TERA 306-327: LDAIAPKREKTHGEVERRIVSQ (#245)

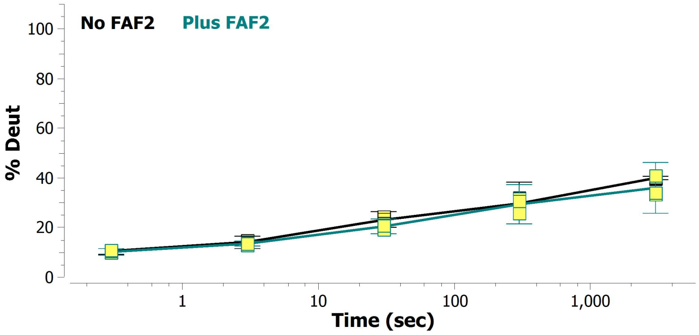

TERA 306-328: LDAIAPKREKTHGEVERRIVSQL (#246)

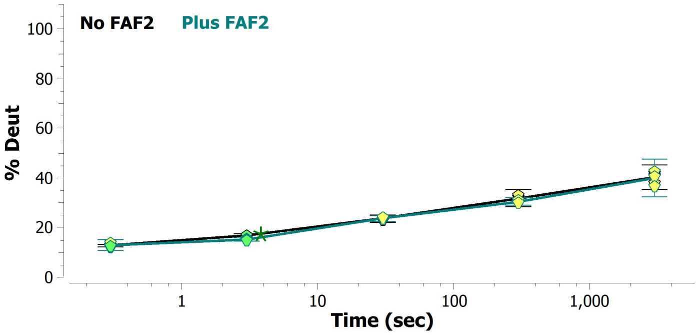

TERA 307-321: DAIAPKREKTHGEVE (#247)

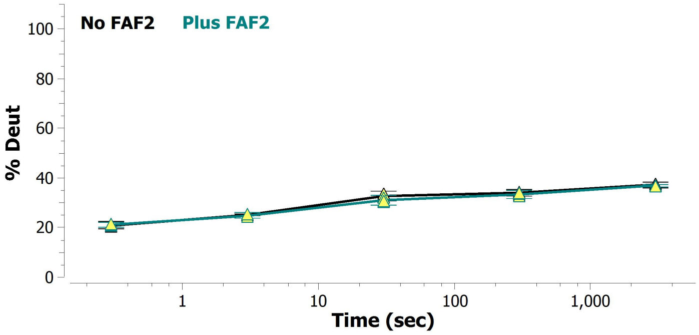

TERA 307-328: DAIAPKREKTHGEVERRIVSQL (#248)

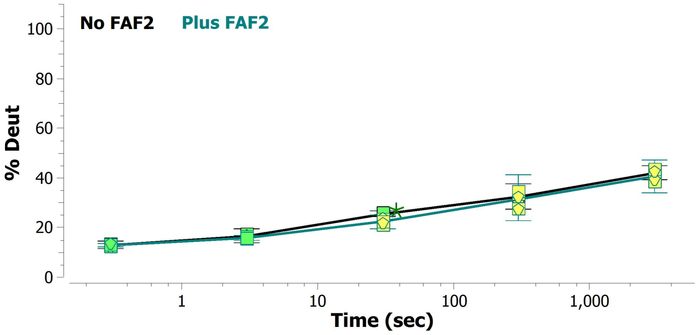

TERA 309-328: IAPKREKTHGEVERRIVSQL (#249)

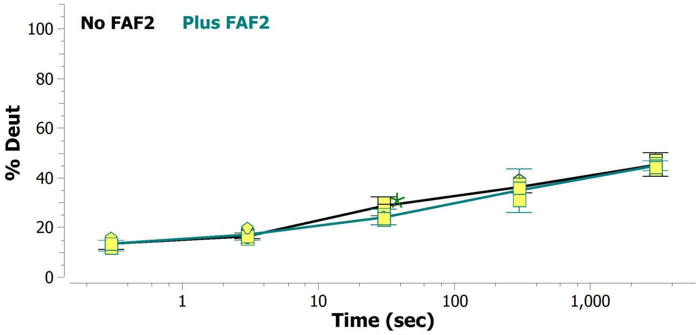

TERA 329-339: LTLMDGLKQRA (#250)

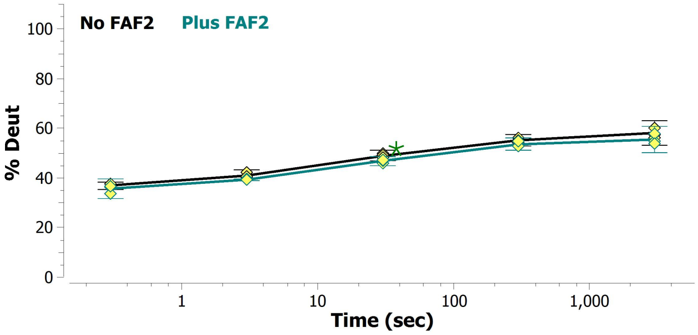

TERA 329-344: LTLMDGLKQRAHVIVM (#251)

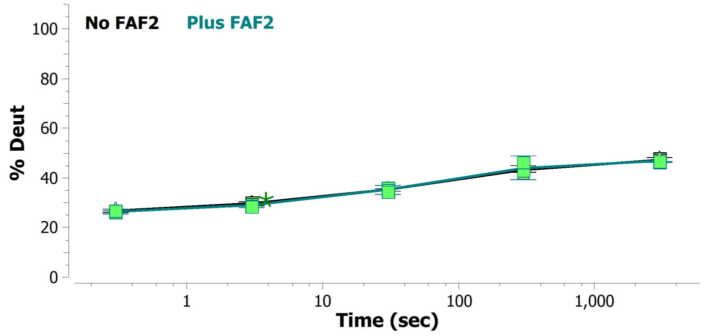

TERA 330-342: TLMDGLKQRAHVI (#252)

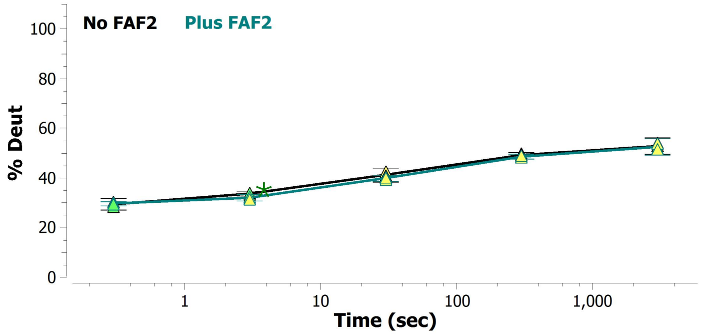

TERA 330-344: TLMDGLKQRAHVIVM (#253)

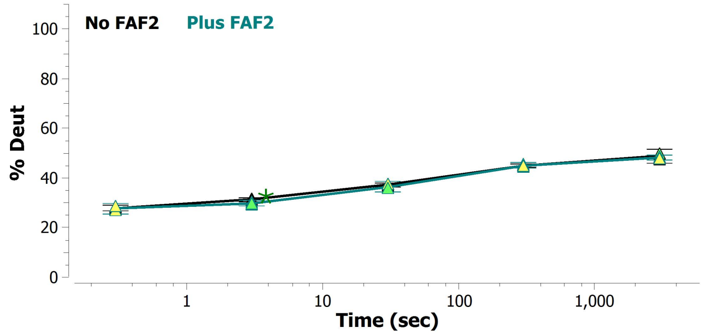

TERA 331-341: LMDGLKQRAHV (#254)

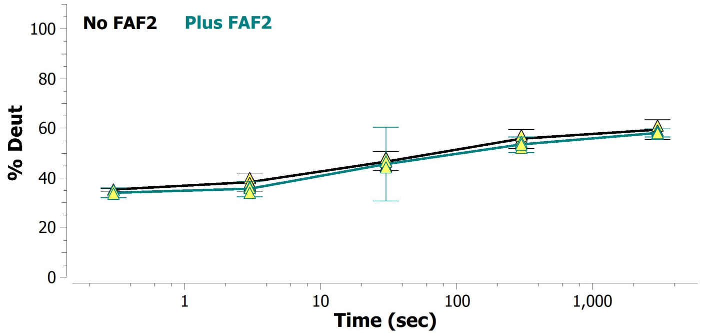

TERA 331-344: LMDGLKQRAHVIVM (#255)

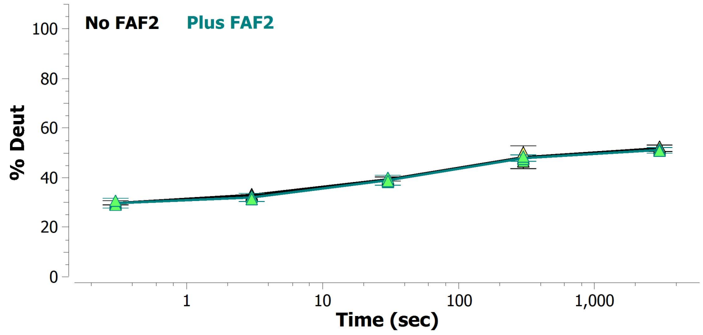

TERA 332-343: MDGLKQRAHVIV (#256)

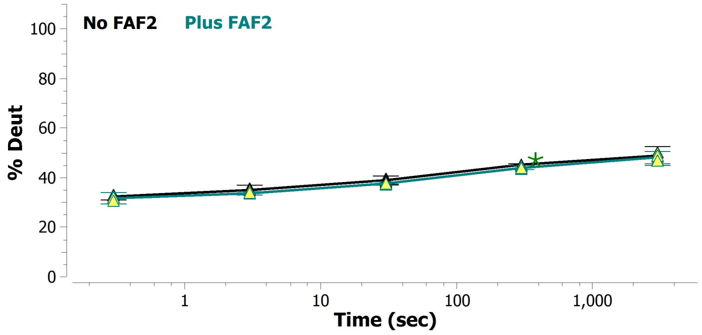

TERA 332-344: MDGLKQRAHVIVM (#257)

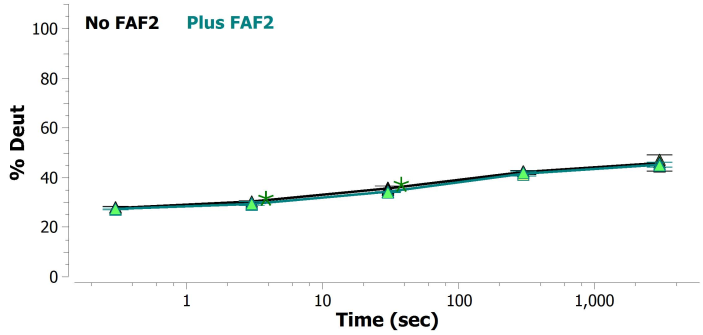

TERA 333-344: DGLKQRAHVIVM (#258)

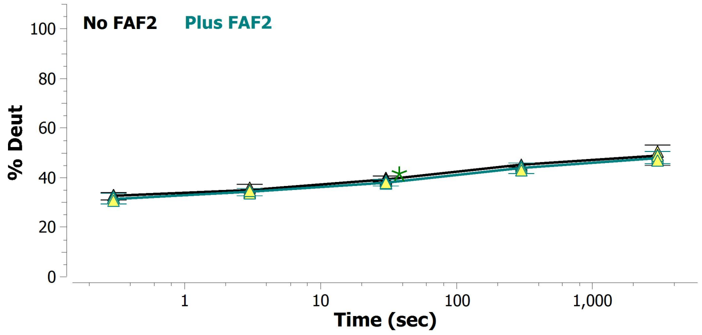

TERA 334-349: GLKQRAHVIVMAATNR (#259)

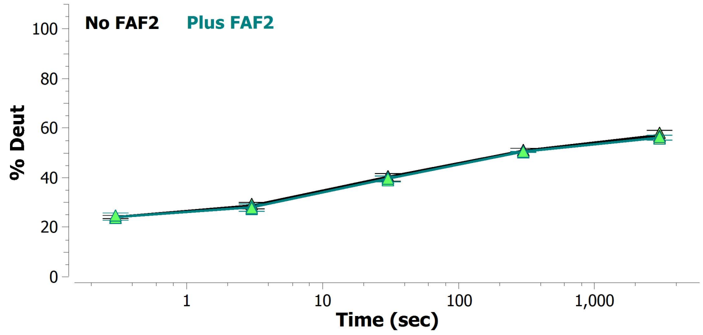

TERA 345-363: AATNRPNSIDPALRRFGRF (#260)

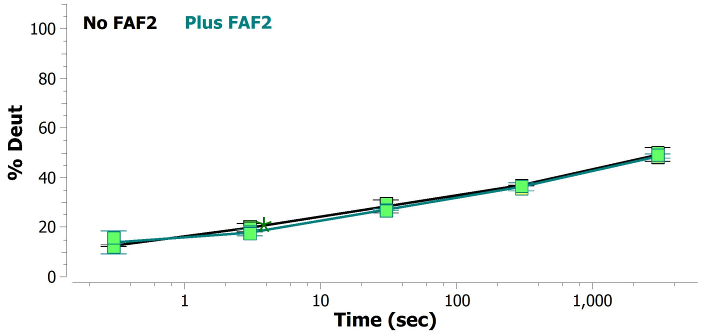

TERA 346-363: ATNRPNSIDPALRRFGRF (#261)

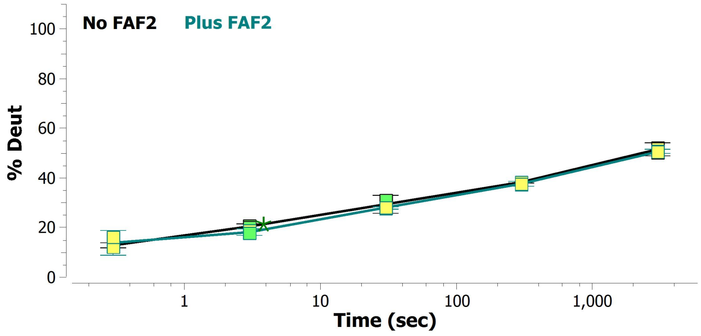

TERA 347-360: TNRPNSIDPALRRF (#262)

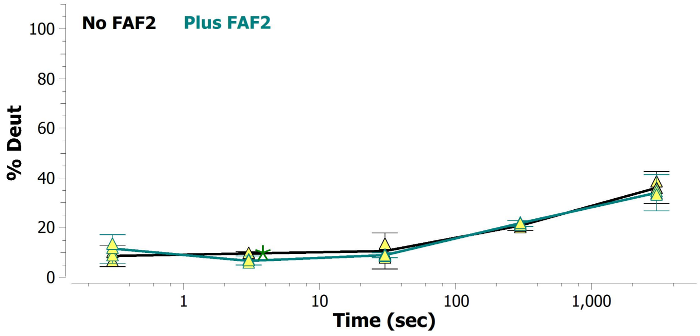

TERA 347-363: TNRPNSIDPALRRFGRF (#263)

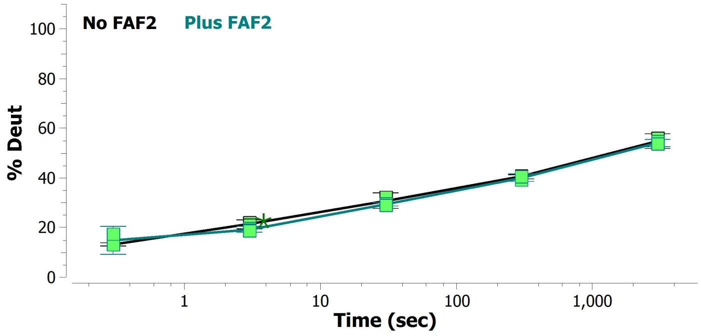

TERA 348-363: NRPNSIDPALRRFGRF (#264)

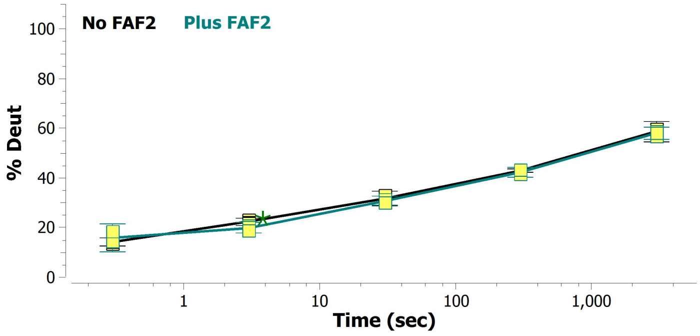

TERA 364-378: DREVDIGIPDATGRL (#265)

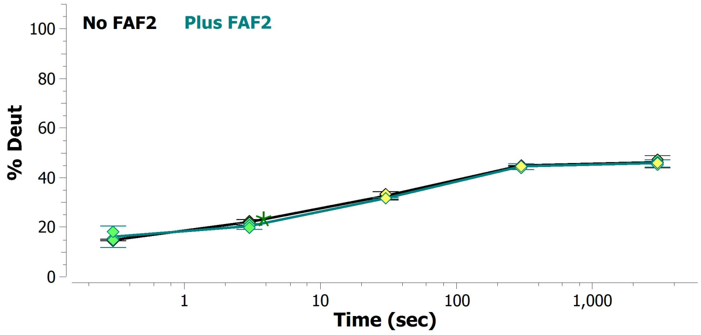

TERA 367-378: VDIGIPDATGRL (#266)

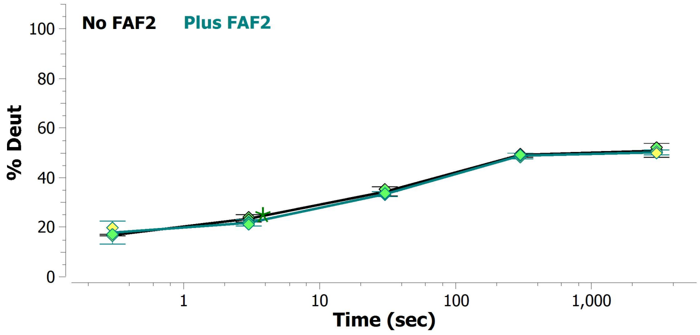

TERA 369-378: IGIPDATGRL (#267)

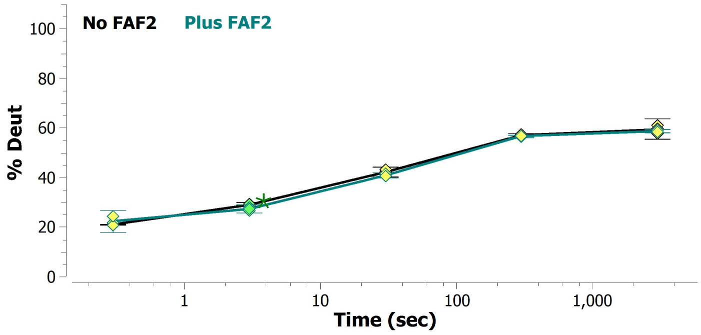

TERA 369-381: IGIPDATGRLEIL (#268)

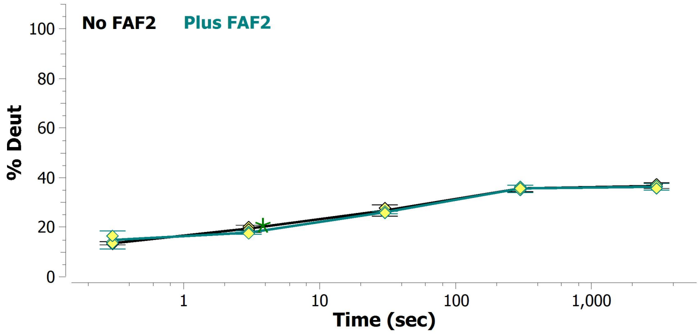

TERA 371-377: IPDATGR (#269)

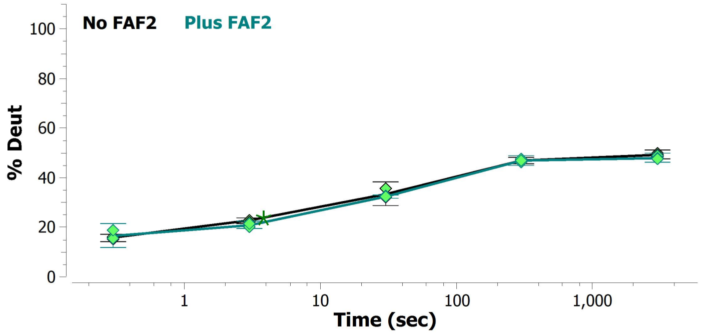

TERA 375-387: TGRLEILQIHTKN (#270)

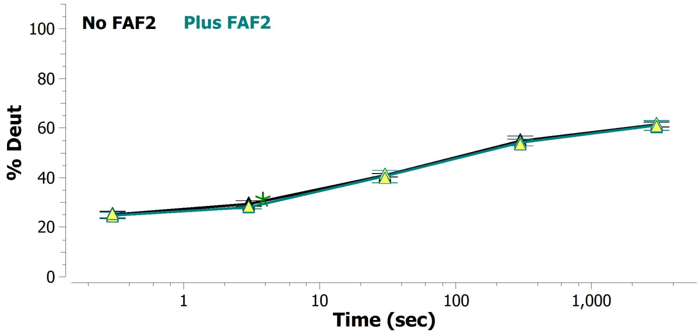

TERA 378-389: LEILQIHTKNMK (#271)

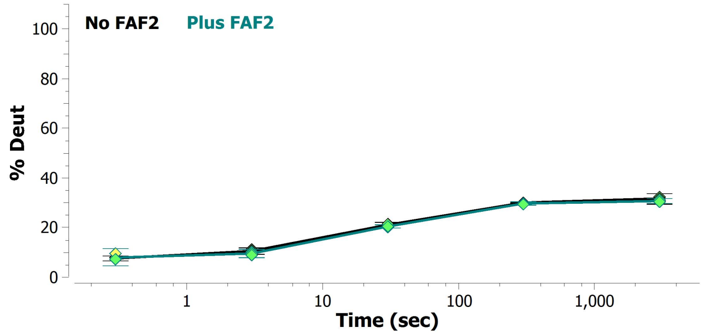

TERA 378-395: LEILQIHTKNMKLADDVD (#272)

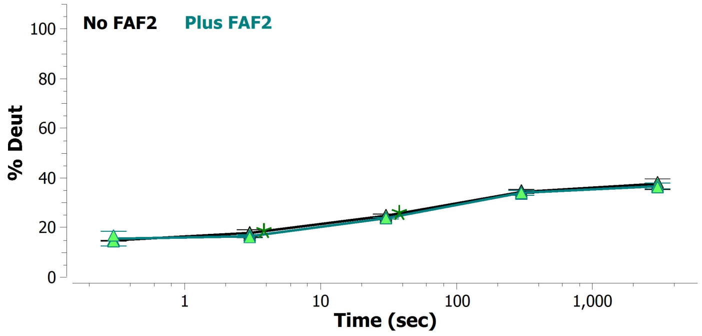

TERA 379-390: EILQIHTKNMKL (#273)

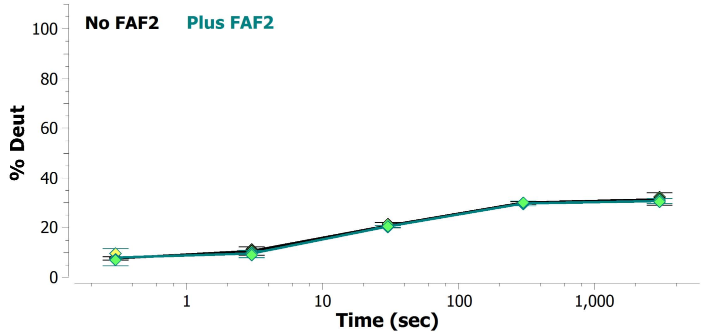

TERA 379-392: EILQIHTKNMKLAD (#274)

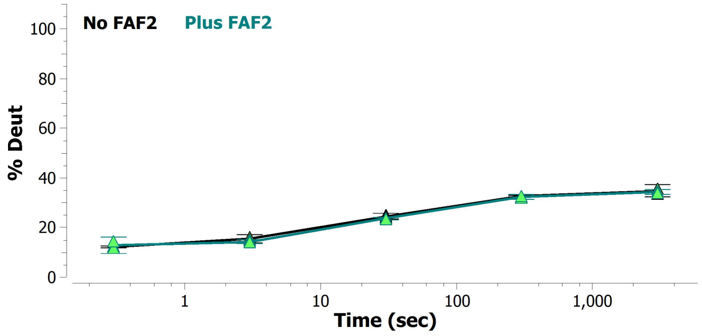

TERA 379-393: EILQIHTKNMKLADD (#275)

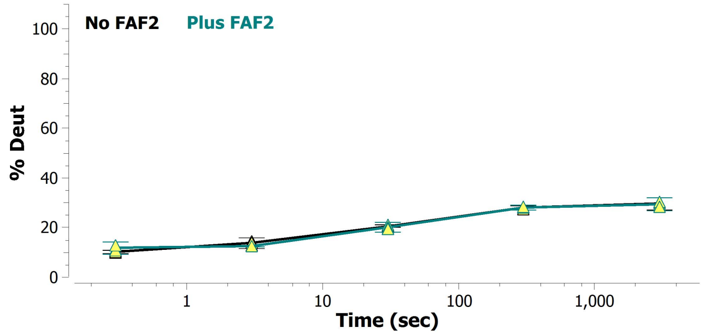

TERA 379-402: EILQIHTKNMKLADDVDLEQVANE (#276)

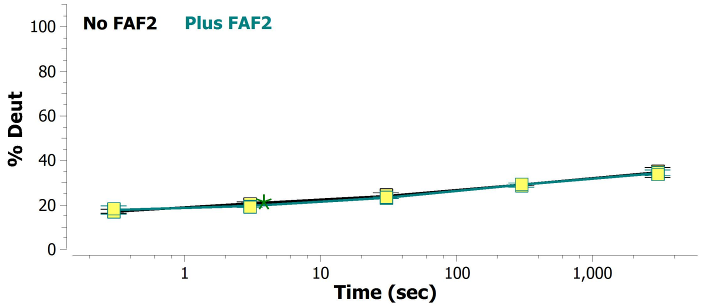

TERA 380-397: ILQIHTKNMKLADDVDLE (#277)

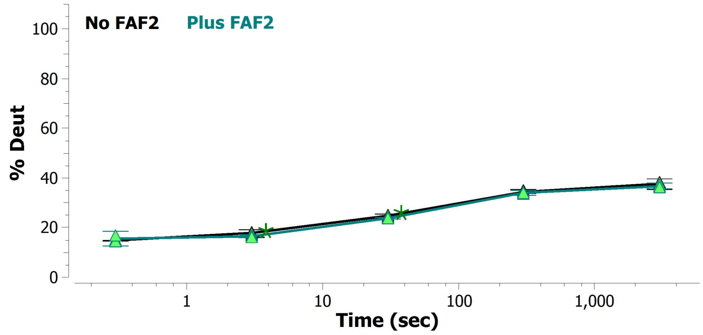

TERA 381-390: LQIHTKNMKL (#278)

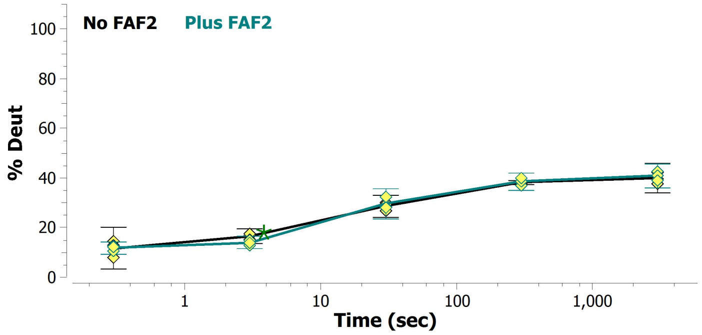

TERA 381-410: LQIHTKNMKLADDVDLEQVANETHGHVGAD (#279)

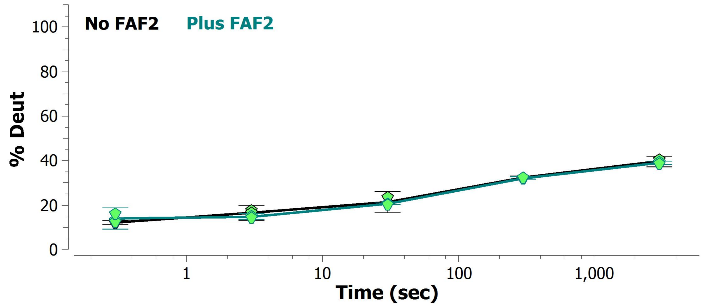

TERA 382-392: QIHTKNMKLAD (#280)

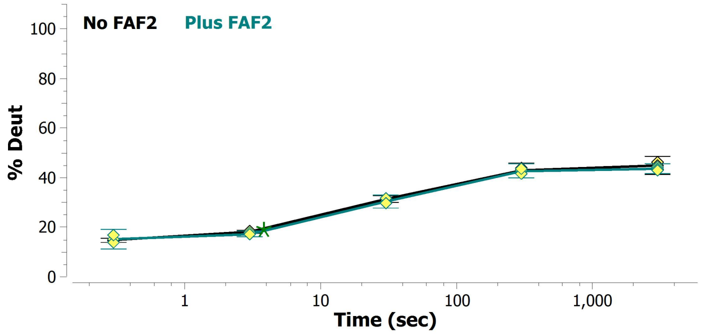

TERA 382-393: QIHTKNMKLADD (#281)

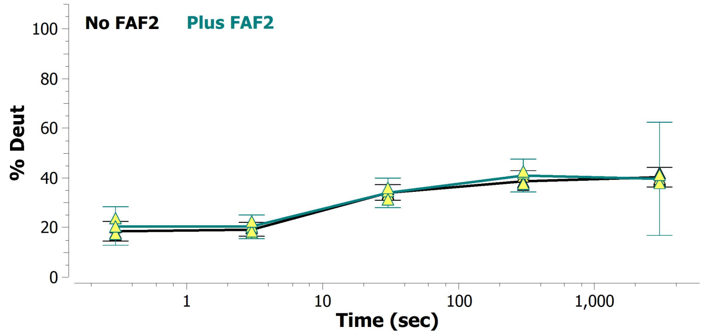

TERA 382-396: QIHTKNMKLADDVDL (#282)

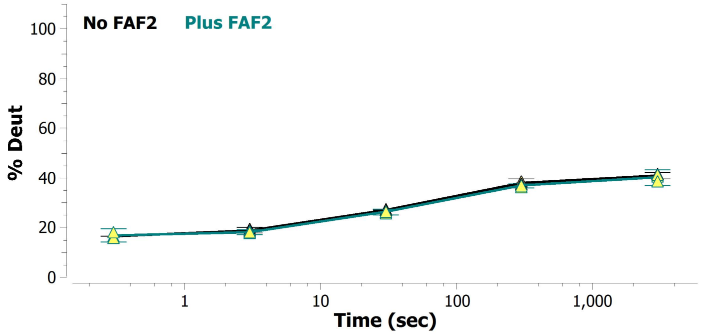

TERA 383-392: IHTKNMKLAD (#283)

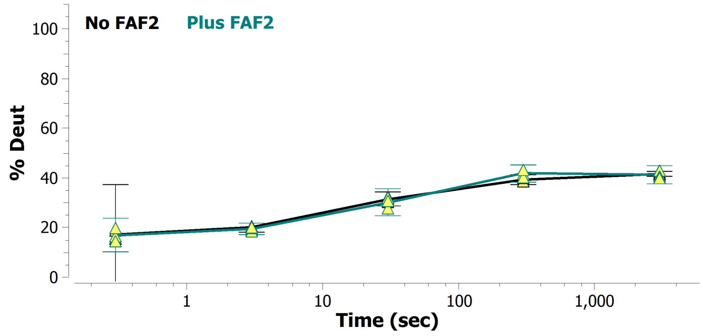

TERA 391-396: ADDVDL (#284)

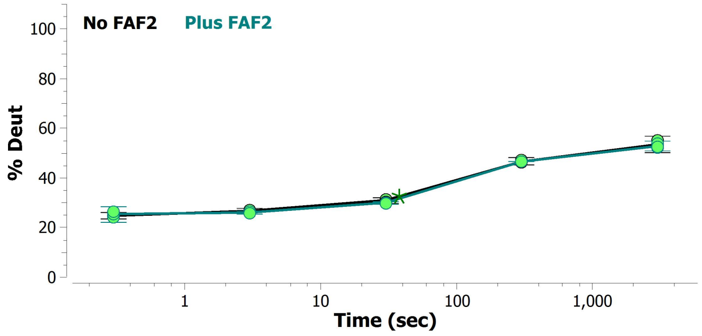

TERA 393-411: DVDLEQVANETHGHVGADL (#285)

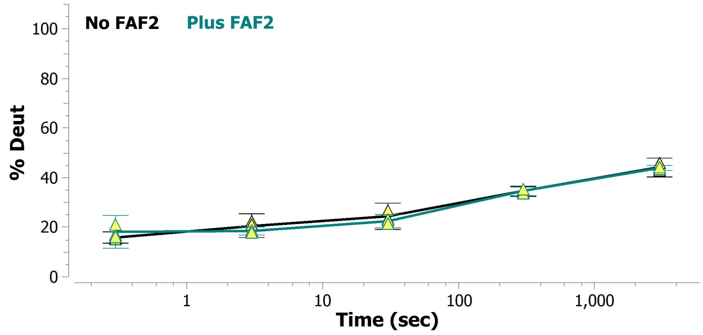

TERA 396-410: LEQVANETHGHVGAD (#286)

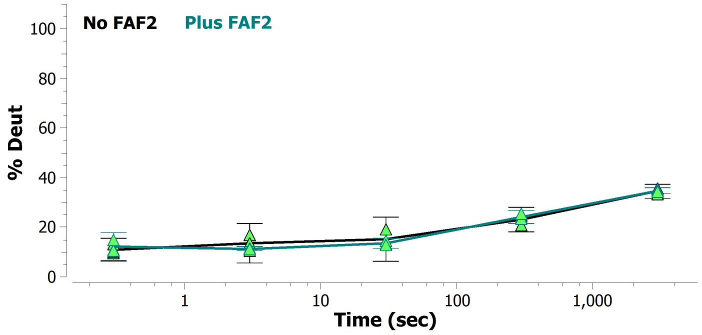

TERA 396-411: LEQVANETHGHVGADL (#287)

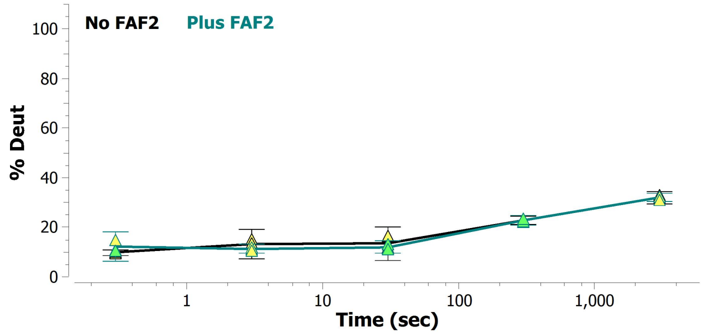

TERA 397-411: EQVANETHGHVGADL (#288)

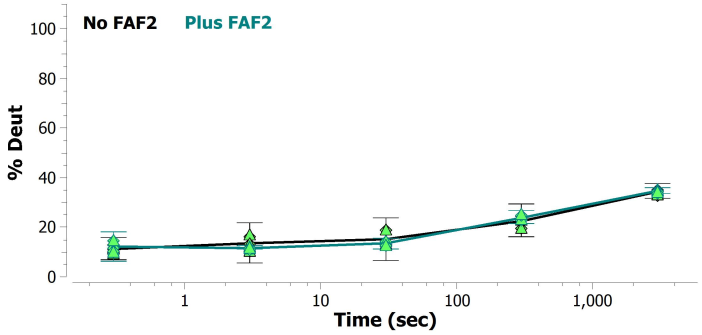

TERA 397-419: EQVANETHGHVGADLAALCSEAA (#289)

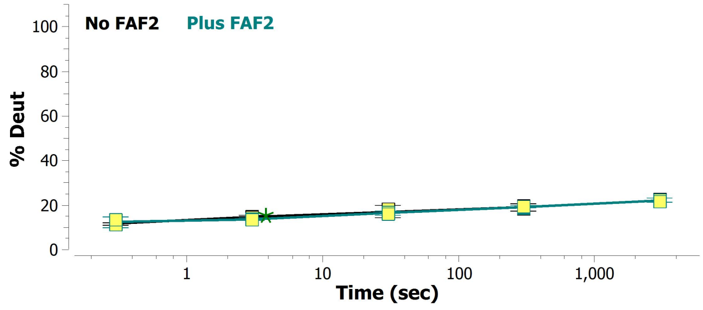

TERA 398-411: QVANETHGHVGADL (#290)

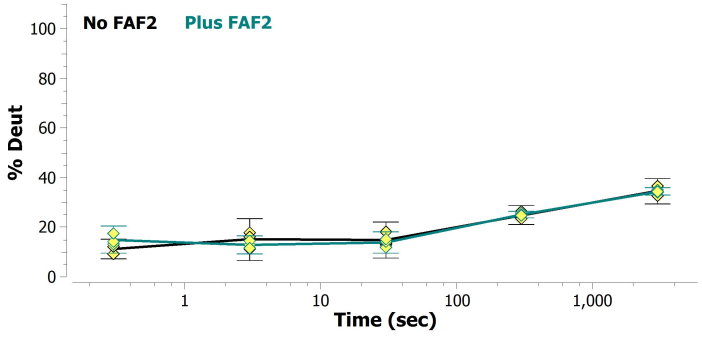

TERA 399-411: VANETHGHVGADL (#291)

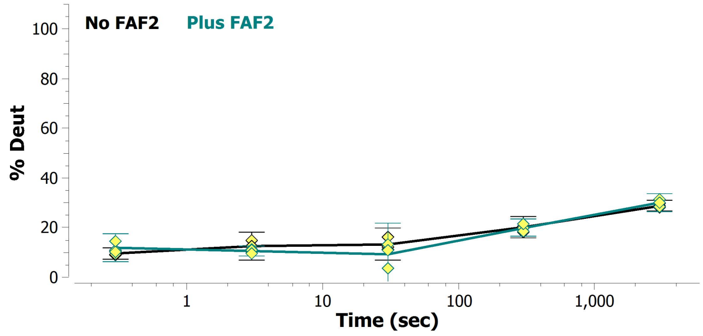

TERA 407-427: VGADLAALCSEAALQAIRKKM (#292)

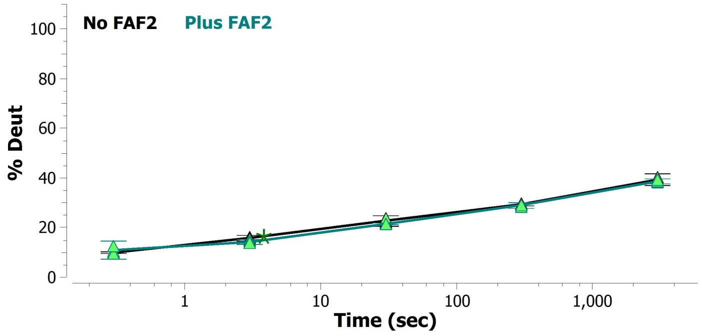

TERA 408-431: GADLAALCSEAALQAIRKKMDLID (#293)

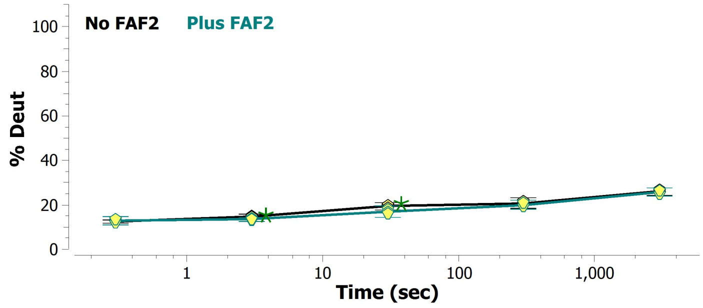

TERA 412-429: AALCSEAALQAIRKKMDL (#294)

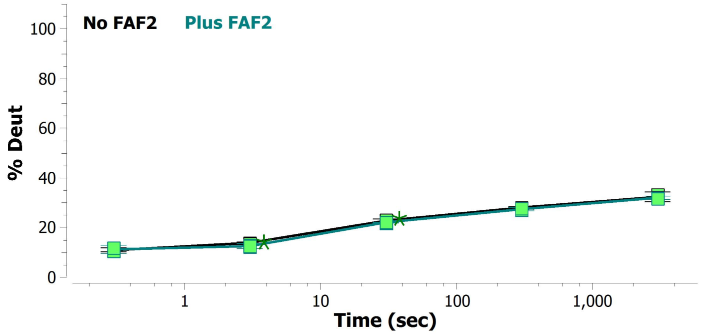

TERA 415-429: CSEAALQAIRKKMDL (#295)

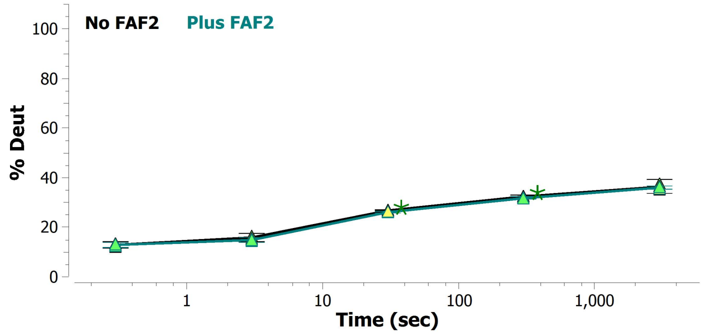

TERA 415-431: CSEAALQAIRKKMDLID (#296)

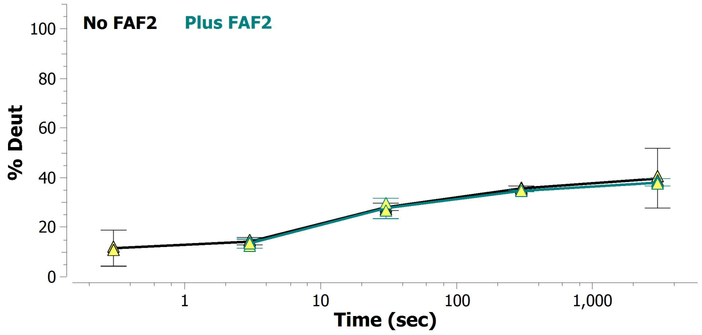

TERA 416-429: SEAALQAIRKKMDL (#297)

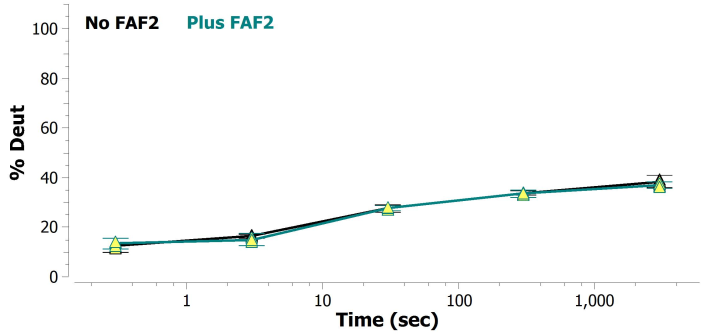

TERA 418-429: AALQAIRKKMDL (#298)

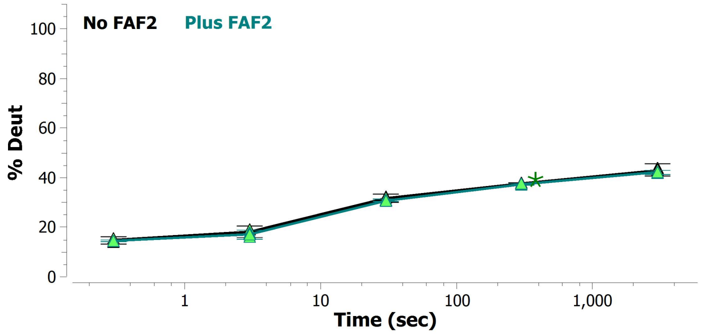

TERA 419-429: ALQAIRKKMDL (#299)

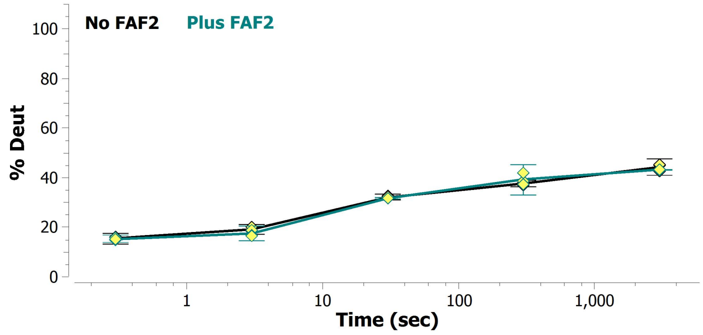

TERA 420-429: LQAIRKKMDL (#300)

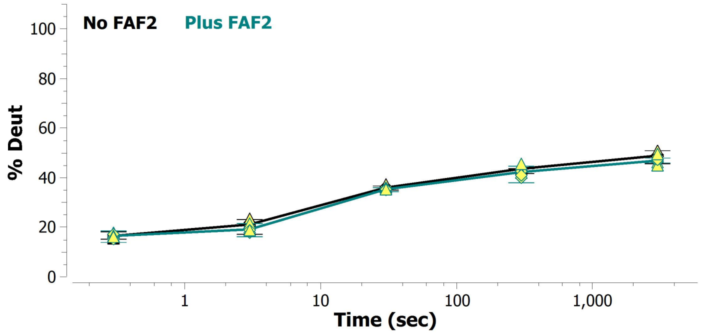

TERA 421-429: QAIRKKMDL (#301)

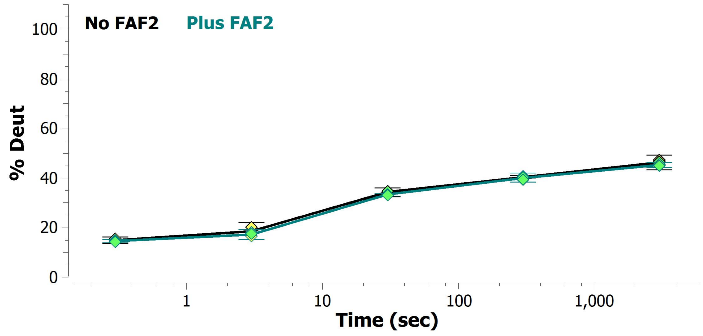

TERA 421-430: QAIRKKMDLI (#302)

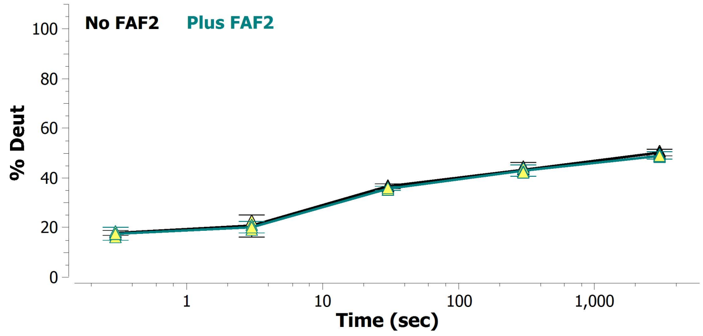

TERA 443-448: NSLAVT (#303)

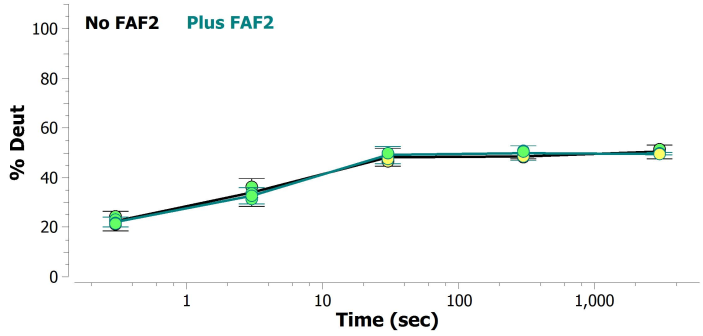

TERA 452-464: FRWALSQSNPSAL (#304)

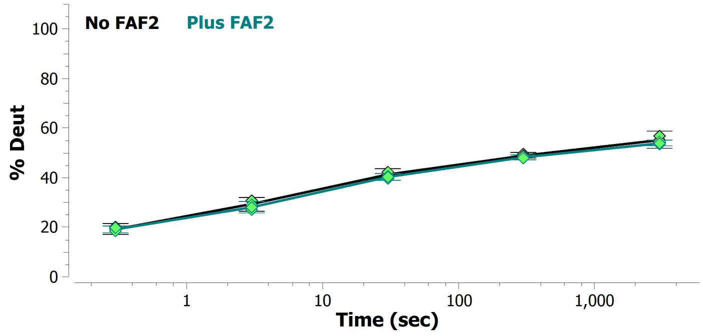

TERA 452-466: FRWALSQSNPSALRE (#305)

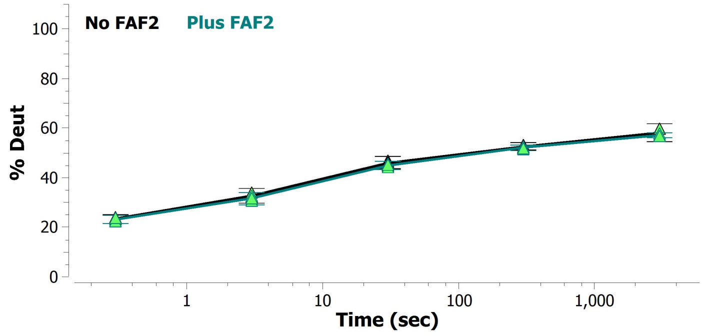

TERA 453-466: RWALSQSNPSALRE (#306)

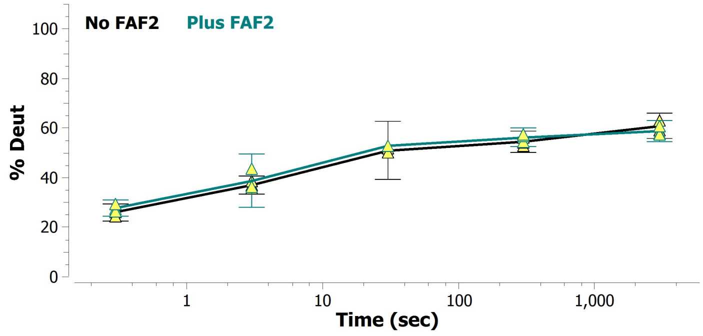

TERA 464-492: LRETVVEVPQVTWEDIGGLEDVKRELQEL (#307)

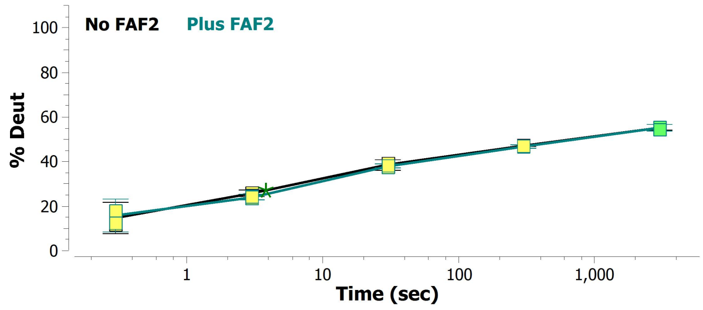

TERA 465-477: RETVVEVPQVTWE (#308)

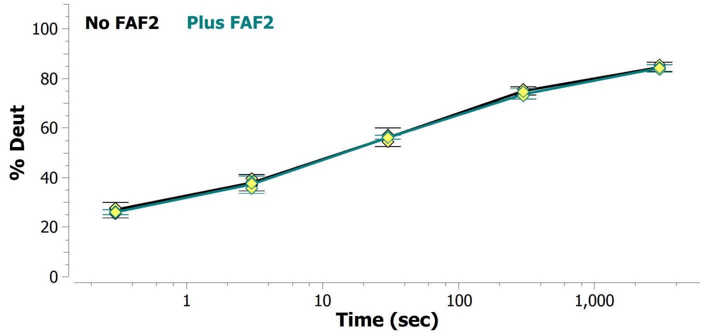

TERA 465-482: RETVVEVPQVTWEDIGGL (#309)

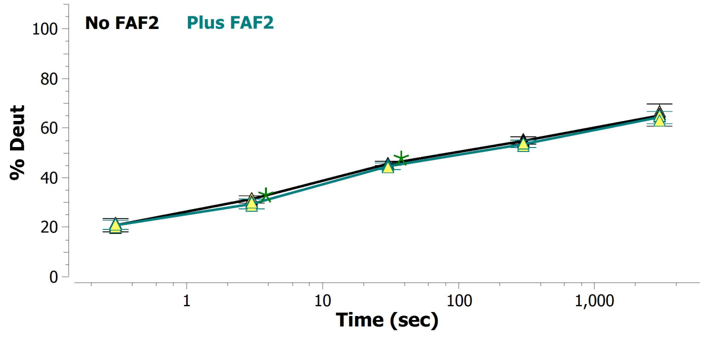

TERA 465-483: RETVVEVPQVTWEDIGGLE (#310)

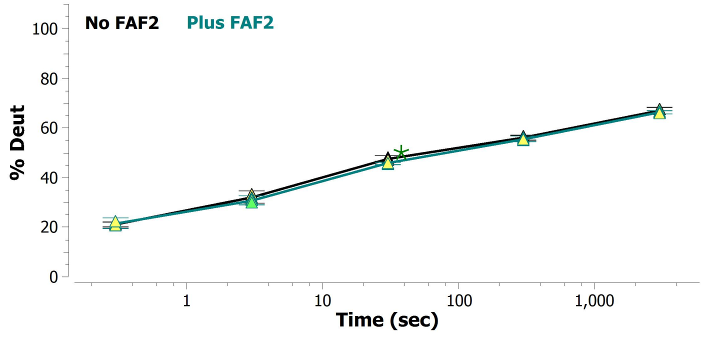

TERA 465-484: RETVVEVPQVTWEDIGGLED (#311)

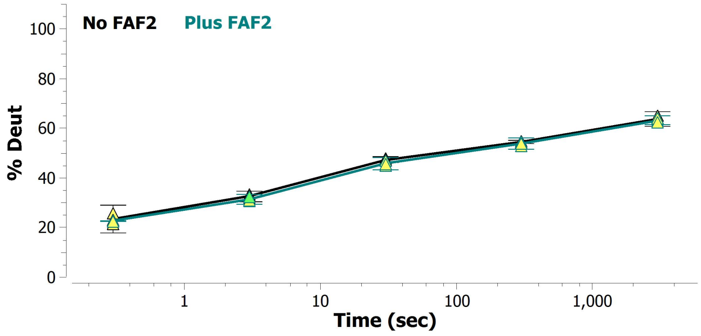

TERA 465-491: RETVVEVPQVTWEDIGGLEDVKRELQE (#312)

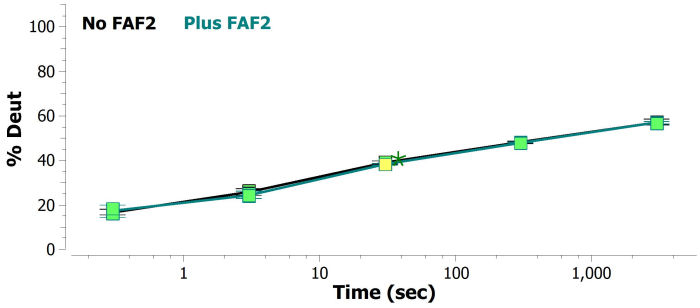

TERA 465-492: RETVVEVPQVTWEDIGGLEDVKRELQEL (#313)

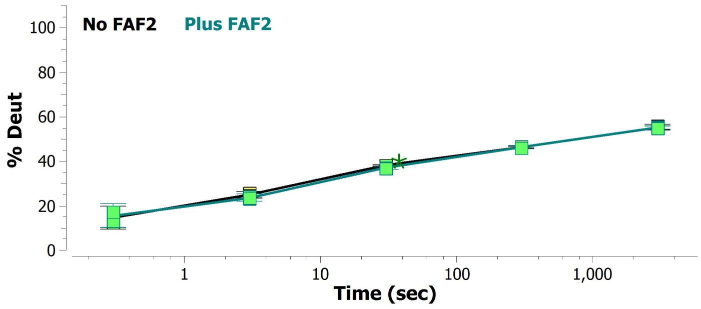

TERA 466-482: ETVVEVPQVTWEDIGGL (#314)

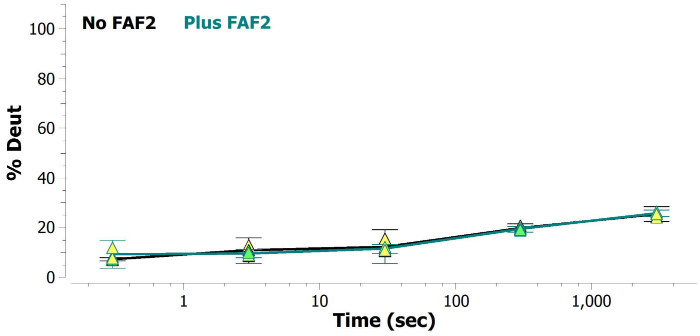

TERA 466-489: ETVVEVPQVTWEDIGGLEDVKREL (#315)

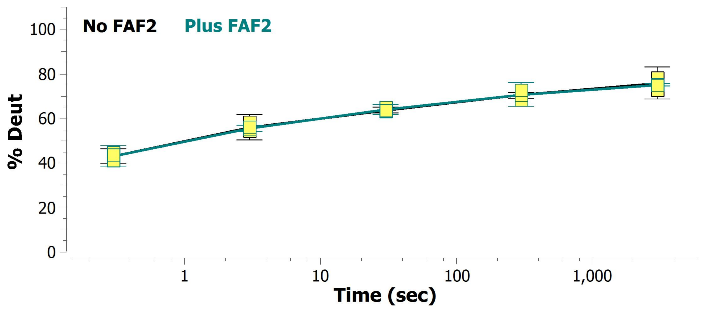

TERA 467-483: TVVEVPQVTWEDIGGLE (#316)

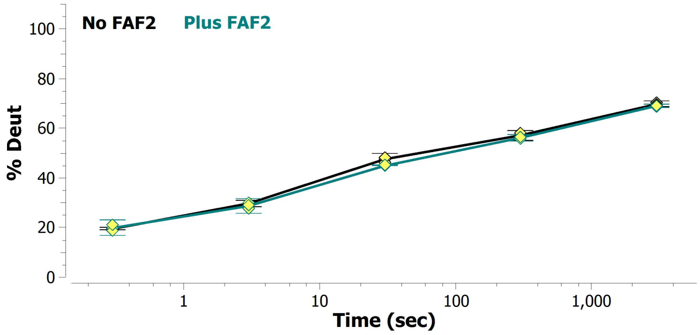

TERA 469-491: VEVPQVTWEDIGGLEDVKRELQE (#317)

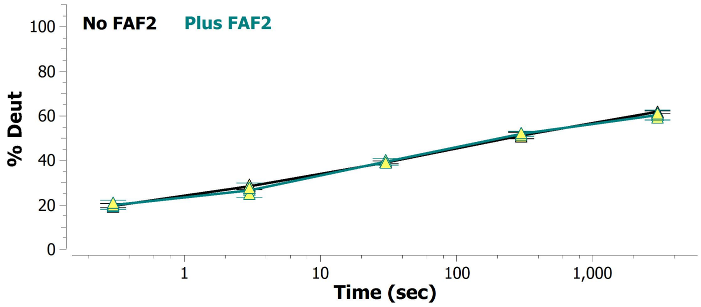

TERA 476-492: WEDIGGLEDVKRELQEL (#318)

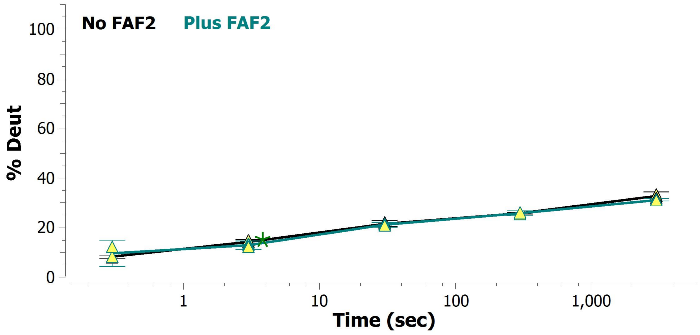

TERA 477-492: EDIGGLEDVKRELQEL (#319)

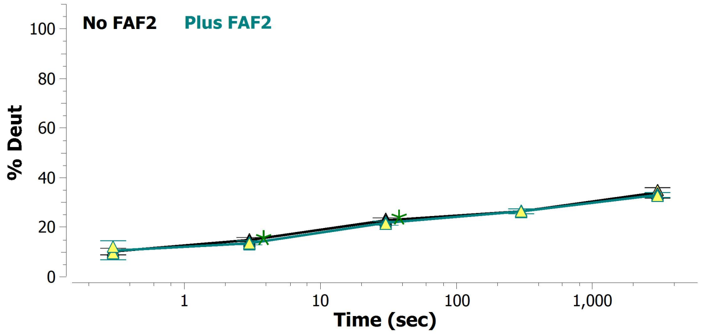

TERA 478-491: DIGGLEDVKRELQE (#320)

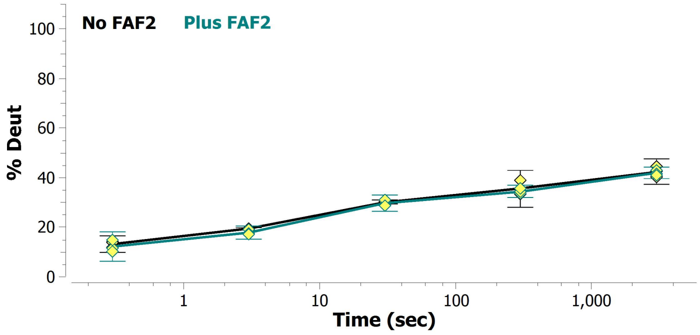

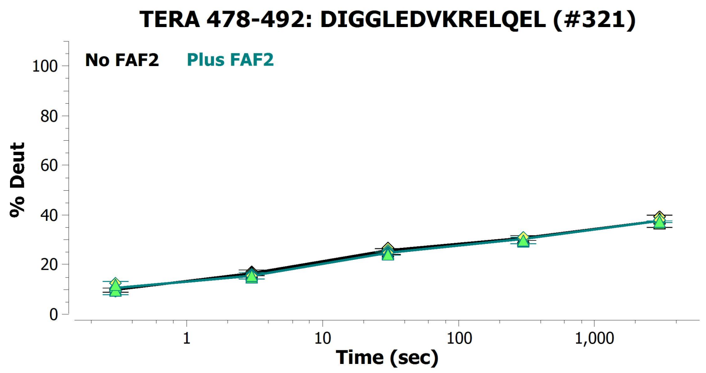
TERA 478-492: DIGGLEDVKRELQEL (#321)
No FAF2
Plus FAF2
% Deut
Time (sec)

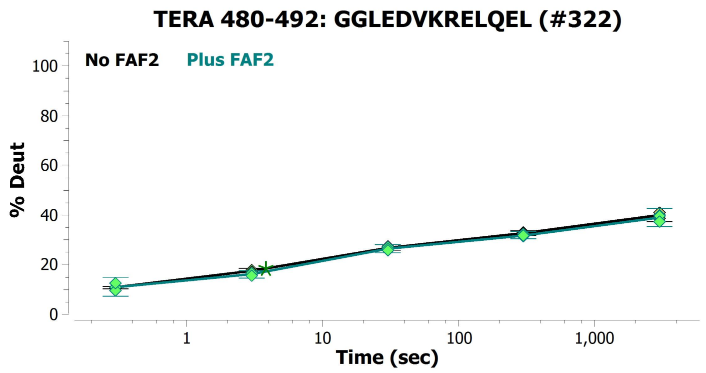
TERA 480-492: GGLEDVKRELQEL (#322)
No FAF2
Plus FAF2
% Deut
Time (sec)

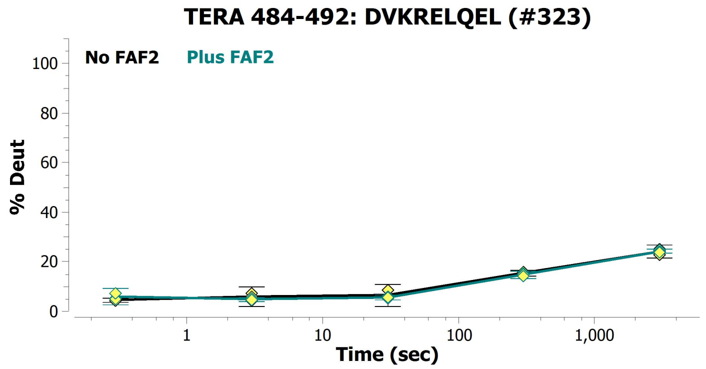
TERA 484-492: DVKRELQEL (#323)
No FAF2
Plus FAF2
% Deut
Time (sec)

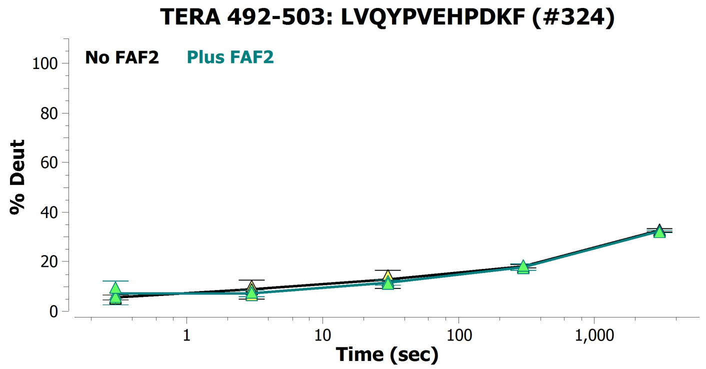
TERA 492-503: LVQYPVEHPDKF (#324)
No FAF2
Plus FAF2
% Deut
Time (sec)

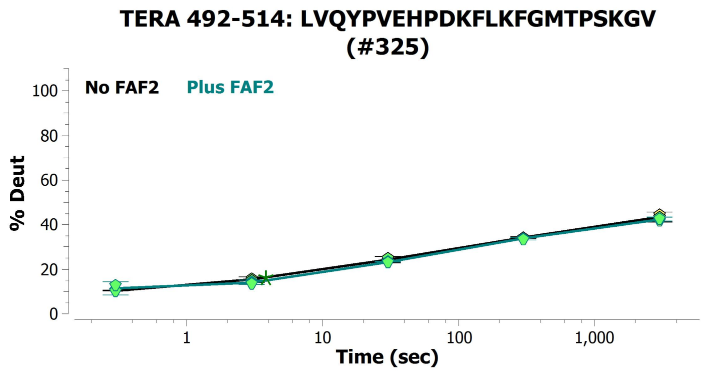
TERA 492-514: LVQYPVEHPDKFLKFGMTPSKGV (#325)
No FAF2
Plus FAF2
% Deut
Time (sec)

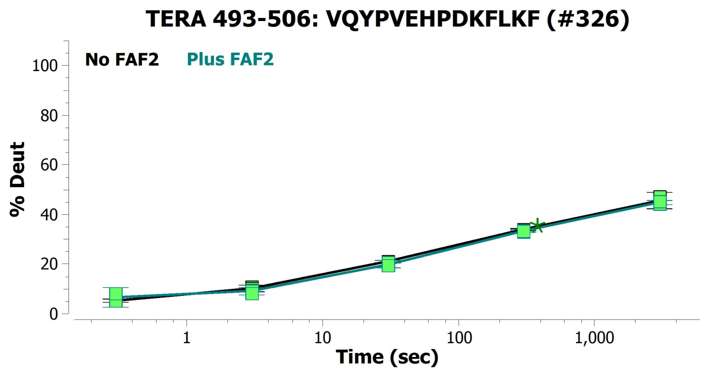
TERA 493-506: VQYPVEHPDKFLKF (#326)
No FAF2
Plus FAF2
% Deut
Time (sec)

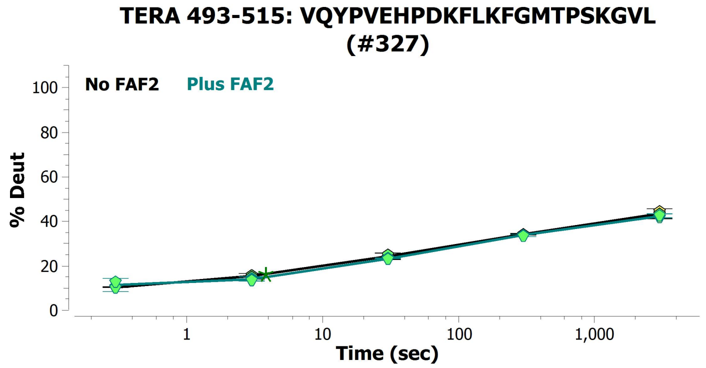
TERA 493-515: VQYPVEHPDKFLKFGMTPSKGVL (#327)
No FAF2
Plus FAF2
% Deut
Time (sec)

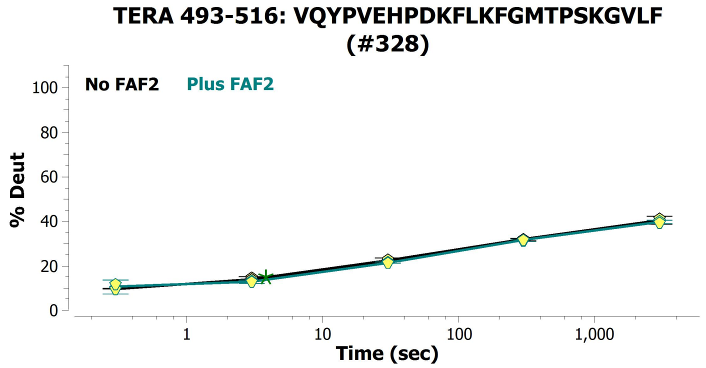
TERA 493-516: VQYPVEHPDKFLKFGMTPSKGVLF (#328)
No FAF2
Plus FAF2
% Deut
Time (sec)

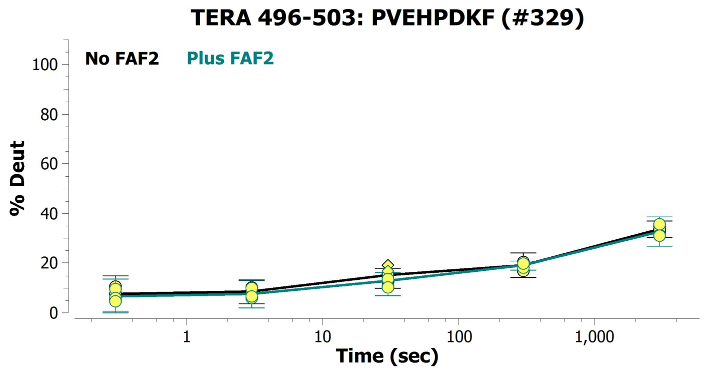
TERA 496-503: PVEHPDKF (#329)
No FAF2
Plus FAF2
% Deut
Time (sec)

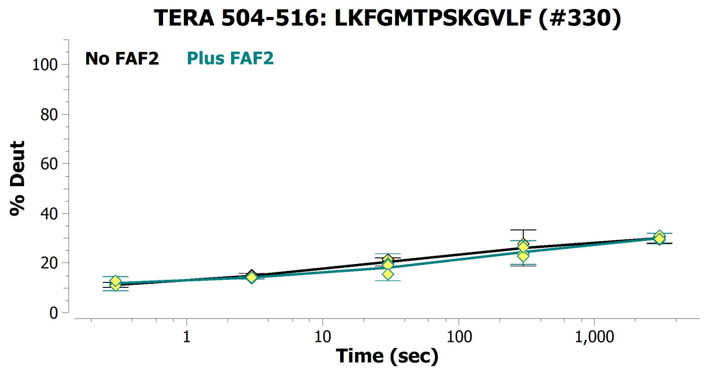
TERA 504-516: LKFGMTPSKGVLF (#330)
No FAF2
Plus FAF2
% Deut
Time (sec)

TERA 504-527: LKFGMTPSKGVLFYGPPGCGKTLL (#331)

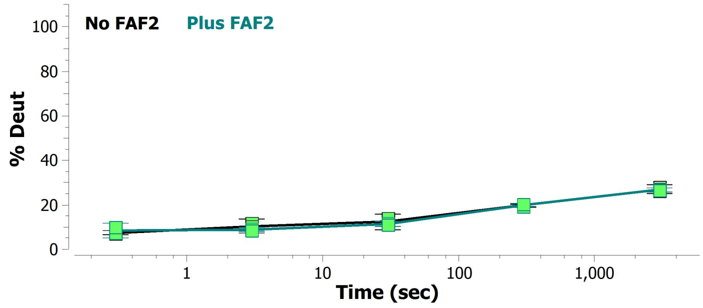

TERA 507-531: GMTPSKGVLFYGPPGCGKTLLAKAI (#332)

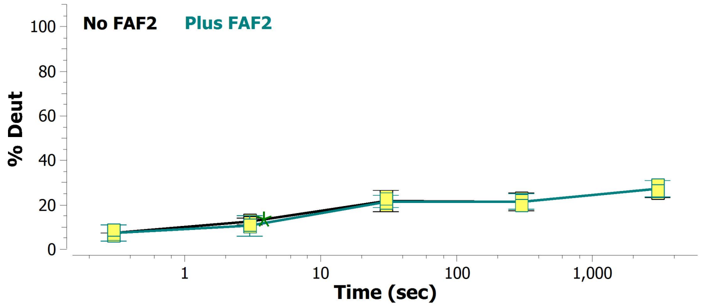

TERA 510-516: PSKGVLF (#333)

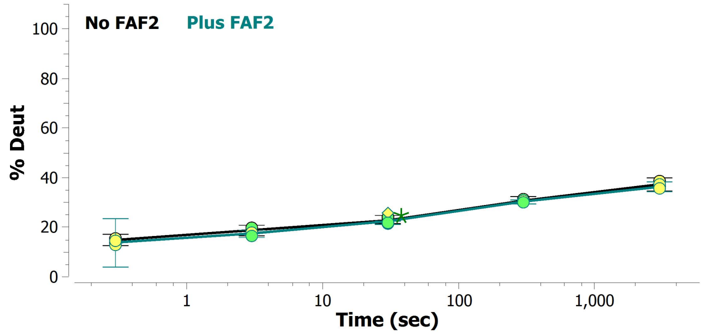

TERA 510-527: PSKGVLFYGPPGCGKTLL (#334)

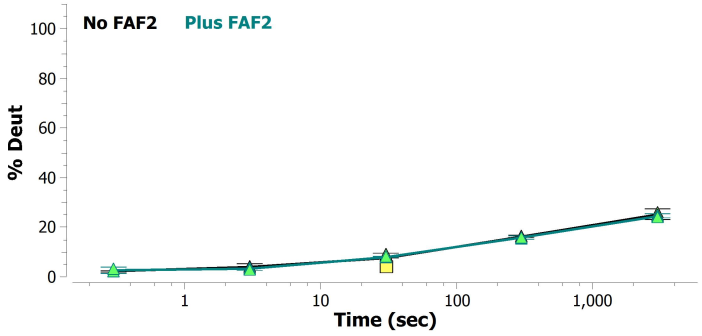

TERA 516-527: FYGPPGCGKTLL (#335)

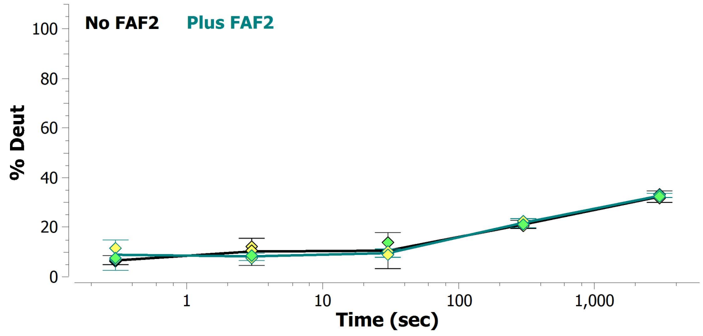

TERA 516-528: FYGPPGCGKTLLA (#336)

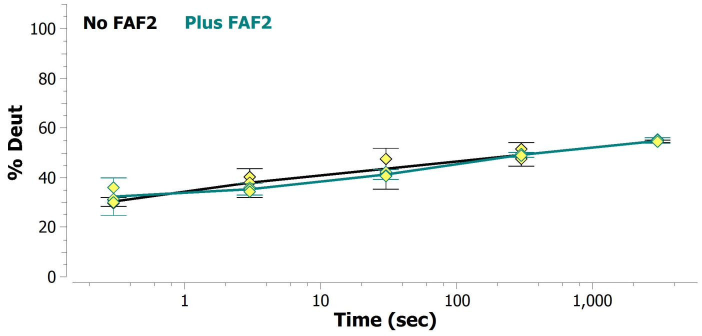

TERA 517-527: YGPPGCGKTLL (#337)

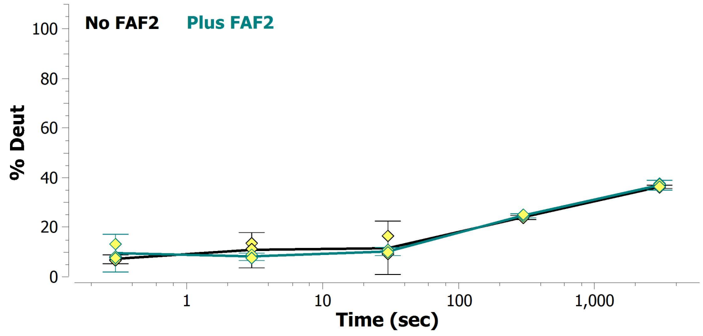

TERA 519-527: PPGCGKTLL (#338)

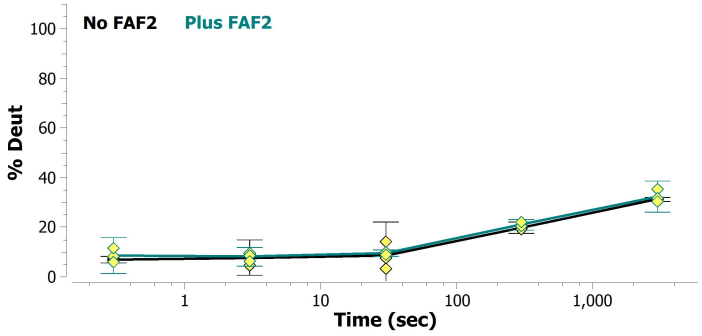

TERA 519-538: PPGCGKTLLAKAIANECQAN (#339)

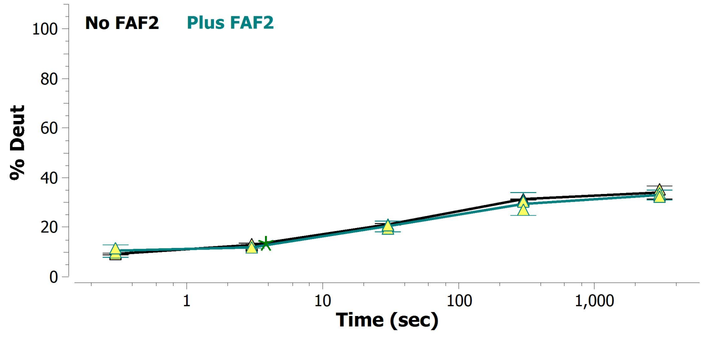

TERA 524-537: KTLLAKAIANECQA (#340)

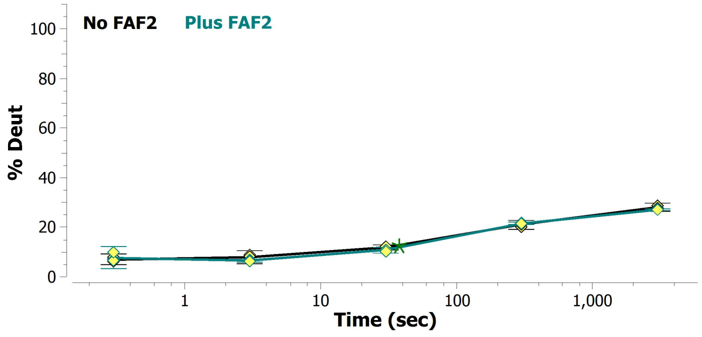

TERA 527-539: LAKAIANECQANF (#341)

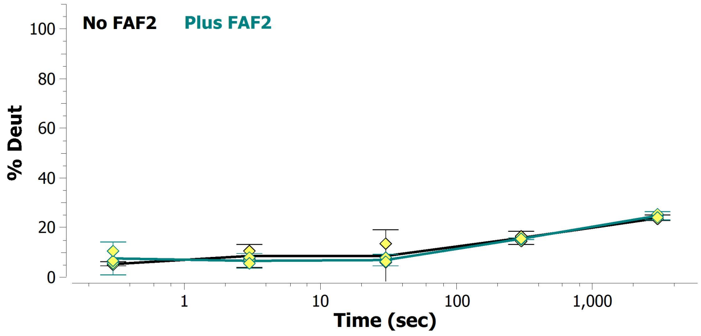

TERA 528-539: AKAIANECQANF (#342)

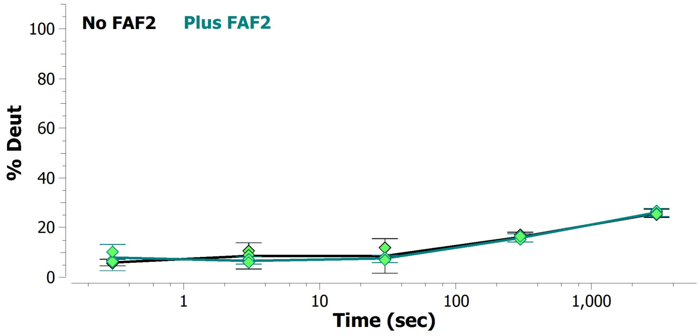

TERA 531-539: IANECQANF (#343)

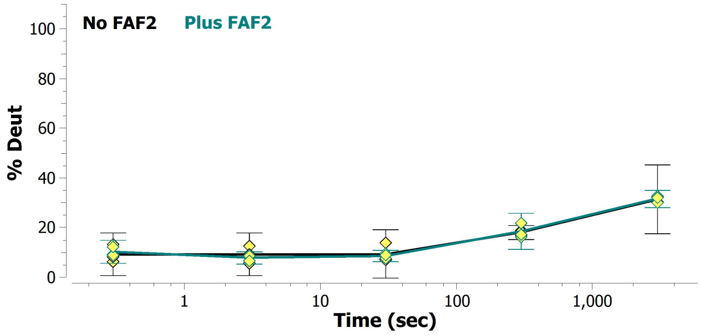

TERA 539-547: FISIKGPEL (#344)

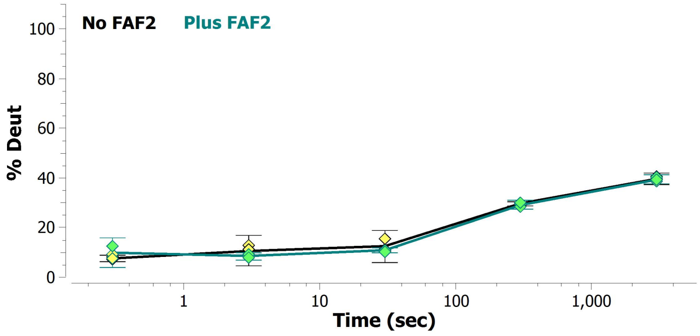

TERA 540-548: ISIKGPELL (#345)

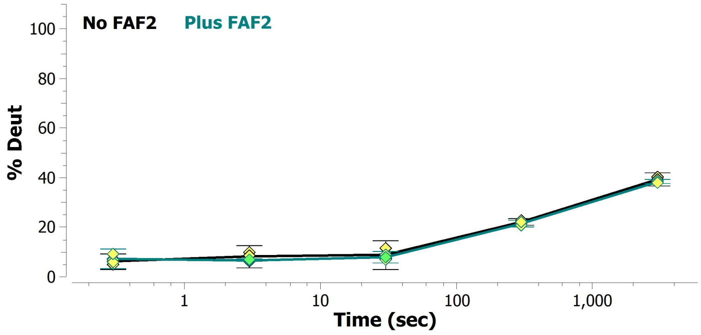

TERA 540-549: ISIKGPELLT (#346)

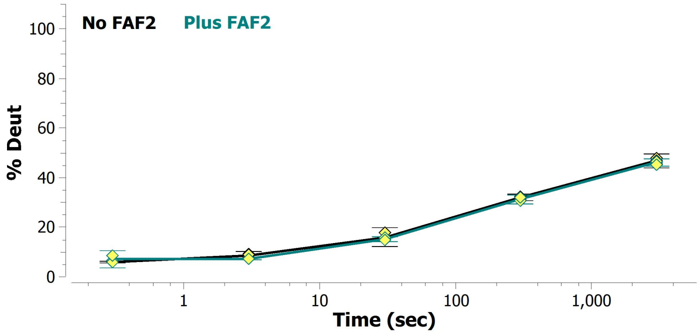

TERA 542-547: IKGPEL (#347)

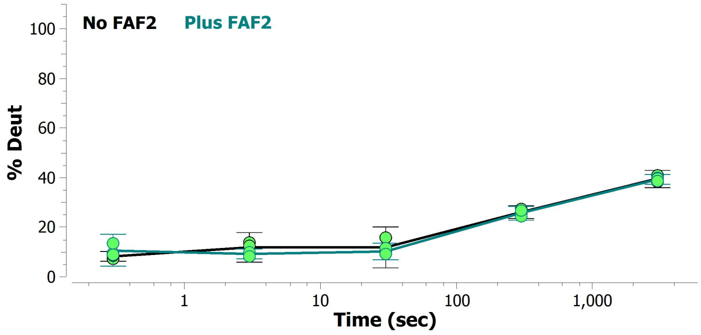

TERA 548-555: LTMWFGES (#348)

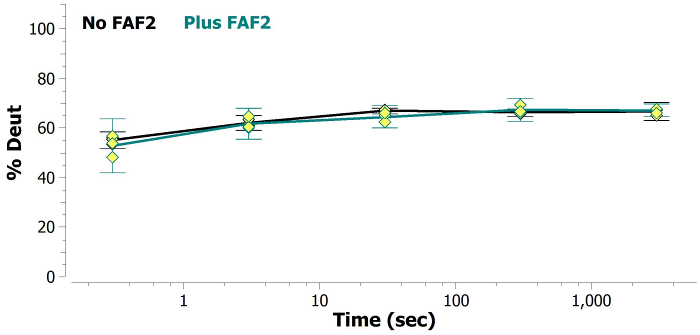

TERA 548-557: LTMWFGESEA (#349)

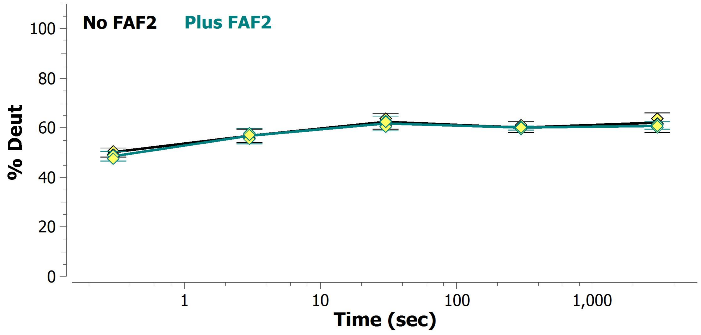

TERA 548-558: LTMWFGESEAN (#350)

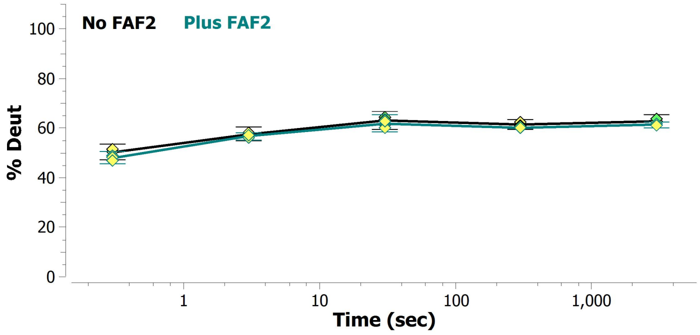

TERA 548-559: LTMWFGESEANV (#351)

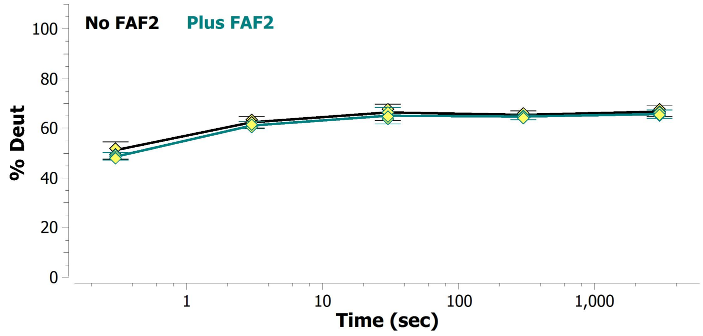

TERA 556-563: EANVREIF (#352)

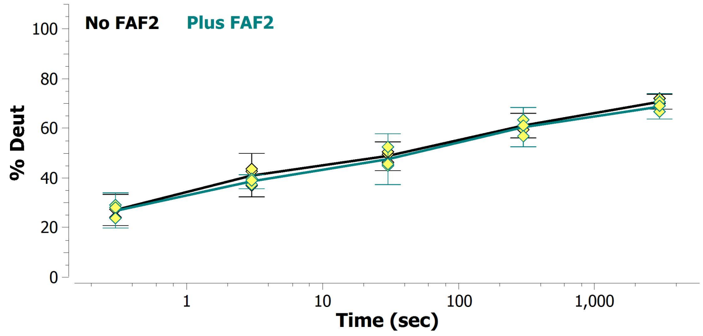

TERA 556-574: EANVREIFDKARQAAPCVL (#353)

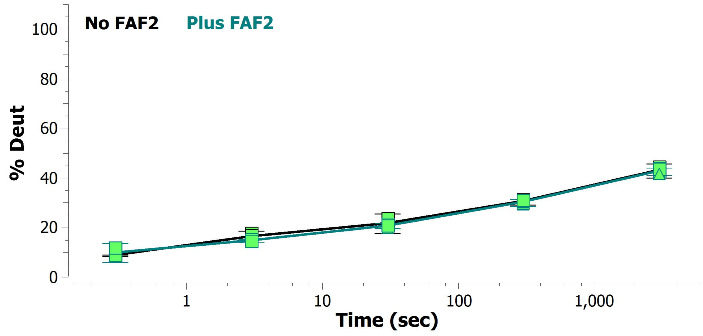

TERA 558-568: NVREIFDKARQ (#354)

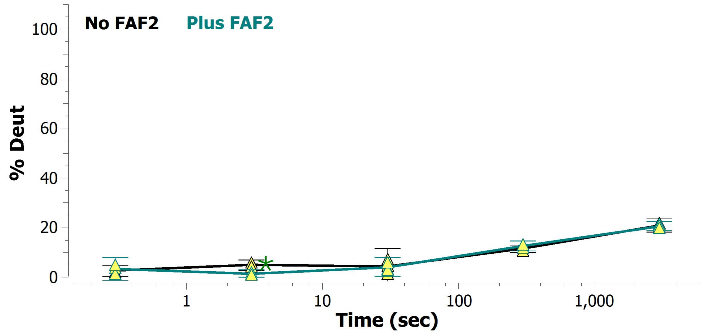

TERA 558-574: NVREIFDKARQAAPCVL (#355)

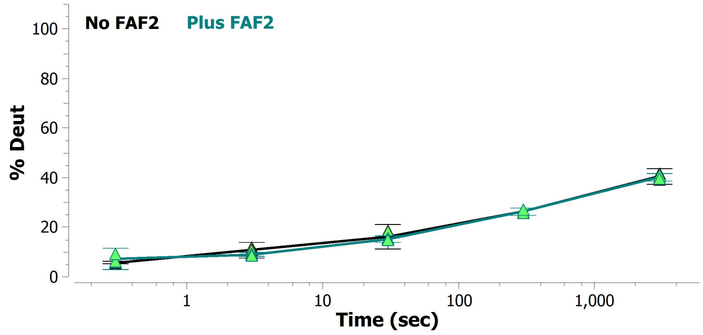

TERA 560-574: REIFDKARQAAPCVL (#356)

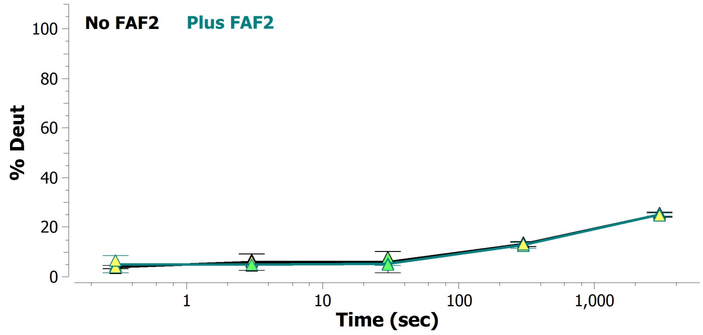

TERA 562-574: IFDKARQAAPCVL (#357)

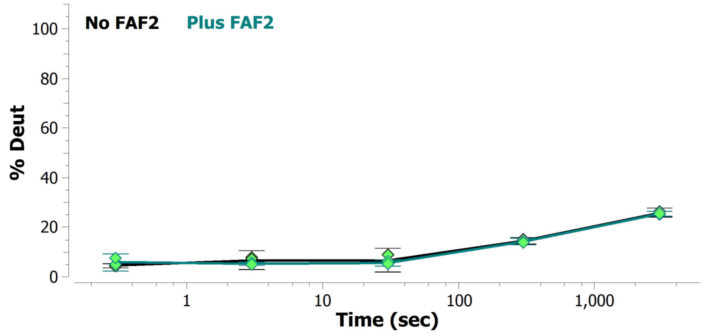

TERA 564-574: DKARQAAPCVL (#358)

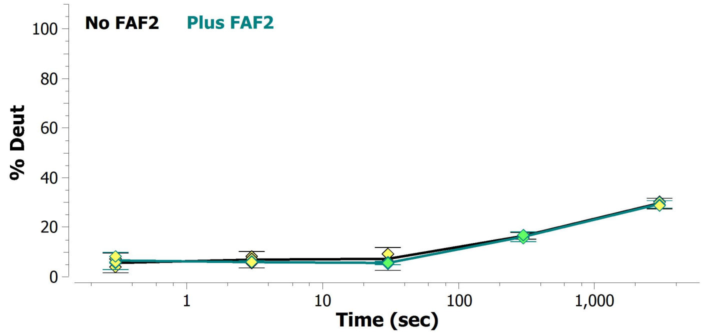

TERA 577-603: DELDSIAKARGGNIGDGGGAADRVINQ (#359)

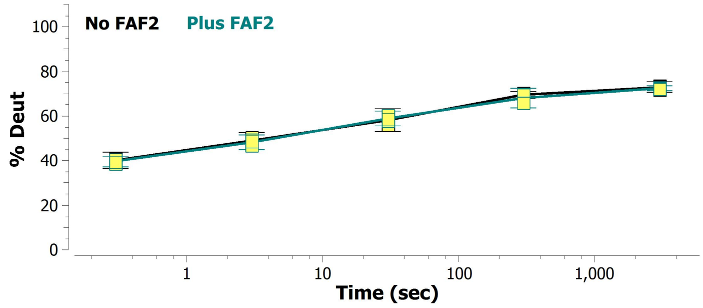

TERA 580-597: DSIAKARGGNIGDGGGAA (#360)

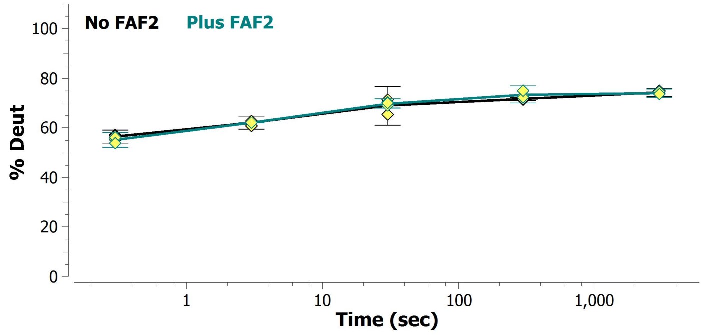

TERA 580-603: DSIAKARGGNIGDGGGAADRVINQ (#361)

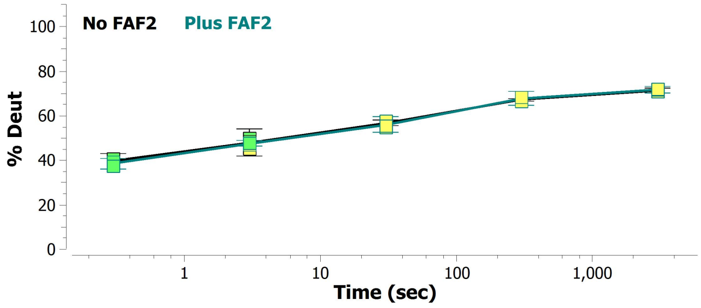

TERA 580-605: DSIAKARGGNIGDGGGAADRVINQIL (#362)

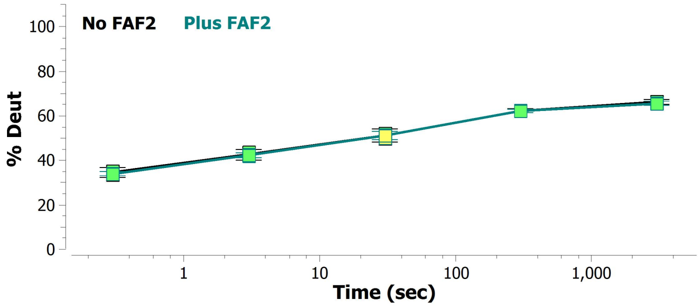

TERA 582-605: IAKARGGNIGDGGGAADRVINQIL (#363)

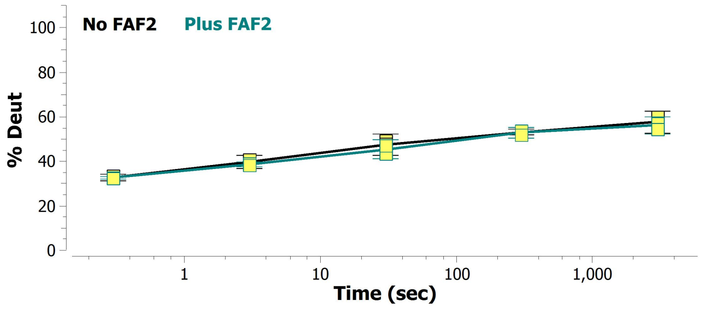

TERA 584-605: KARGGNIGDGGGAADRVINQIL (#364)

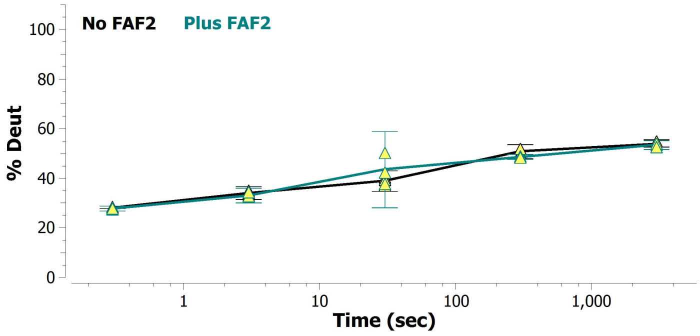

TERA 585-596: ARGGNIGDGGGA (#365)

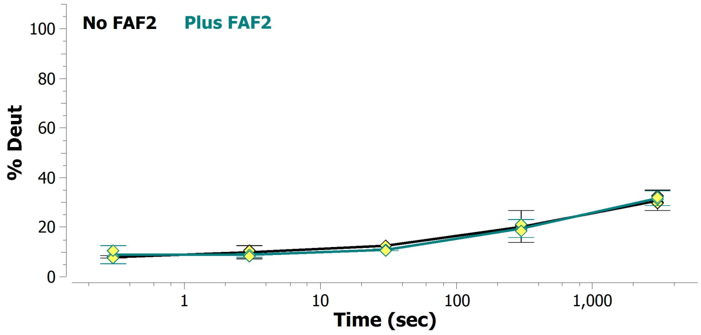

TERA 598-605: DRVINQIL (#366)

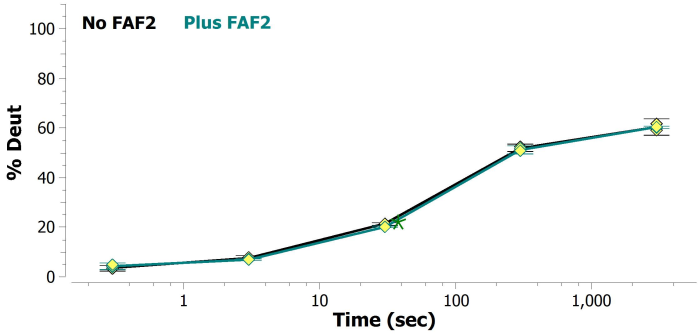

TERA 600-620: VINQILTEMDGMSTKKNVFII (#367)

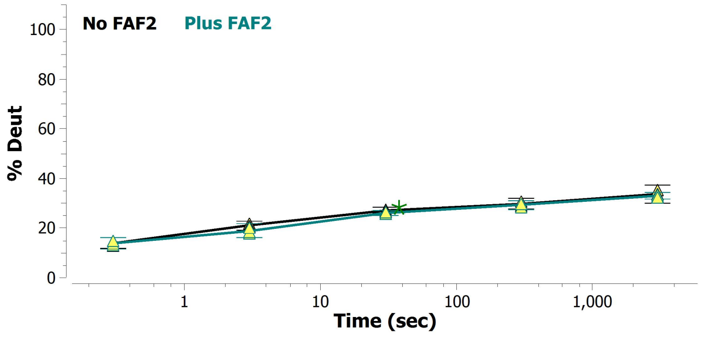

TERA 604-618: ILTEMDGMSTKKNVF (#368)

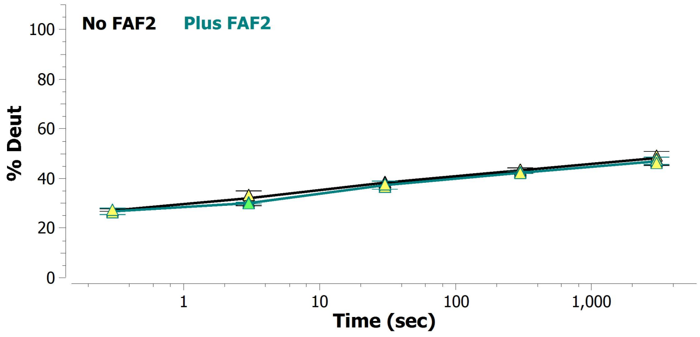

TERA 605-617: LTEMDGMSTKKNV (#369)

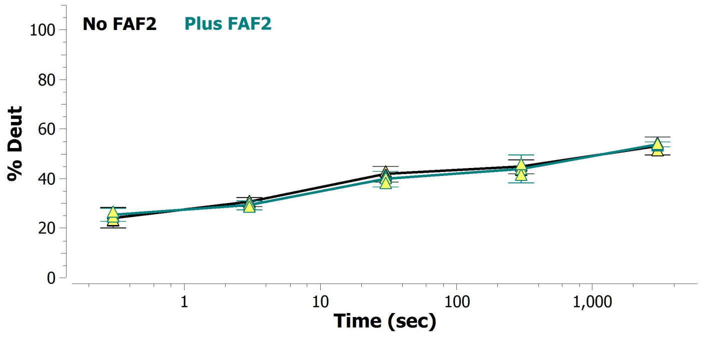

TERA 606-618: TEMDGMSTKKNVF (#370)

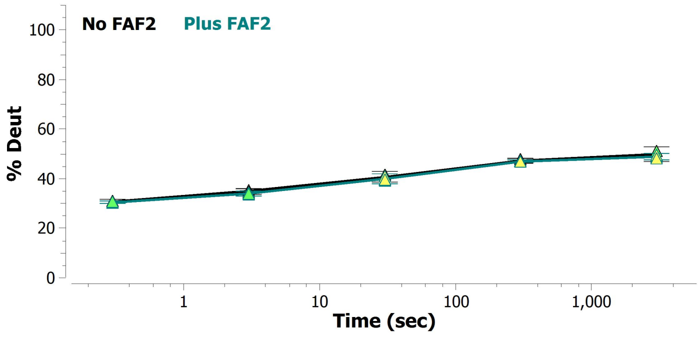

TERA 608-618: MDGMSTKKNVF (#371)

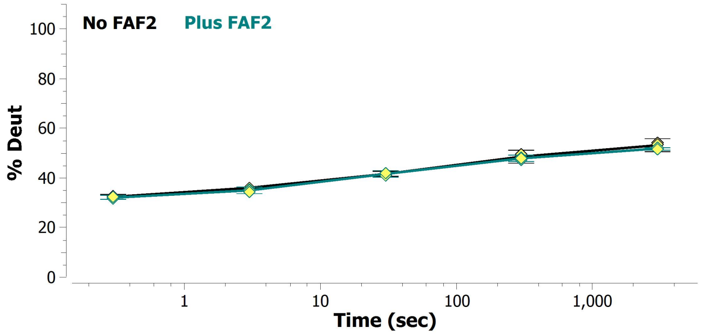

TERA 609-618: DGMSTKKNVF (#372)

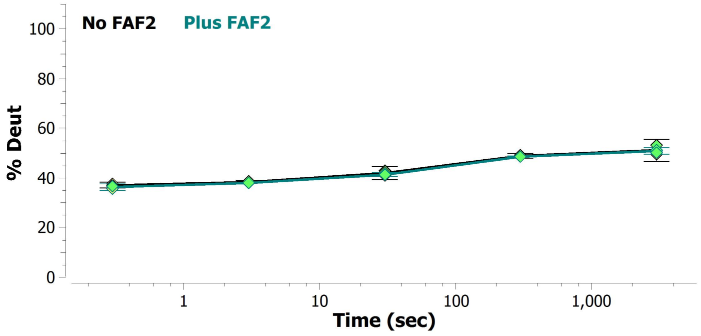

TERA 619-630: IIGATNRPDIID (#373)

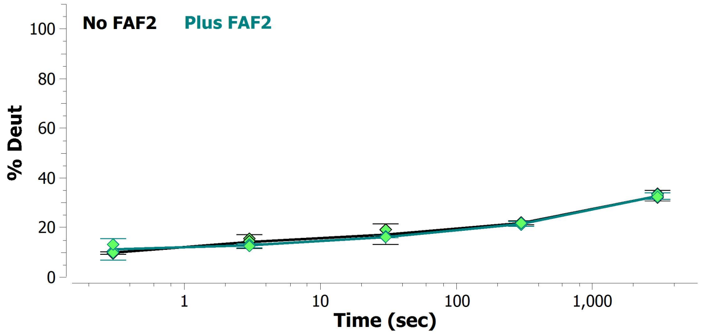

TERA 619-634: IIGATNRPDIIDPAIL (#374)

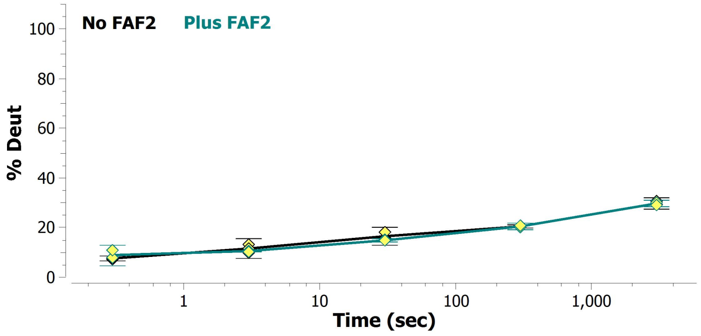

TERA 619-639: IIGATNRPDIIDPAILRPGRL (#375)

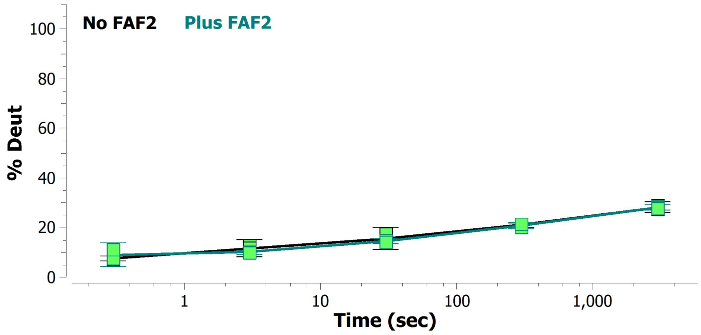

TERA 619-642: IIGATNRPDIIDPAILRPGRLDQL (#376)

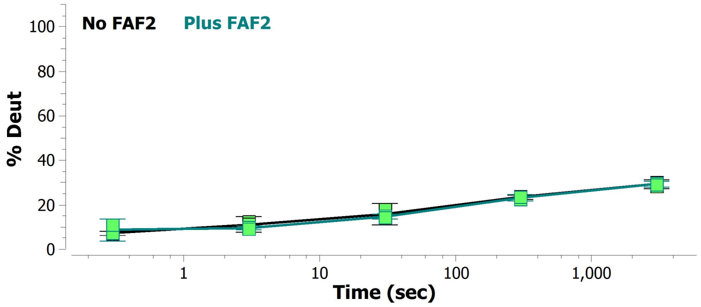

TERA 621-639: GATNRPDIIDPAILRPGRL (#377)

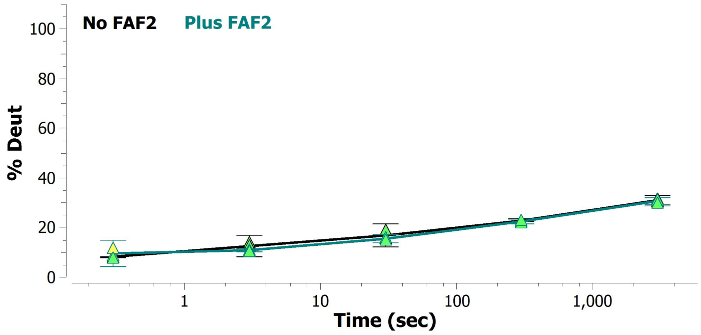

TERA 622-639: ATNRPDIIDPAILRPGRL (#378)

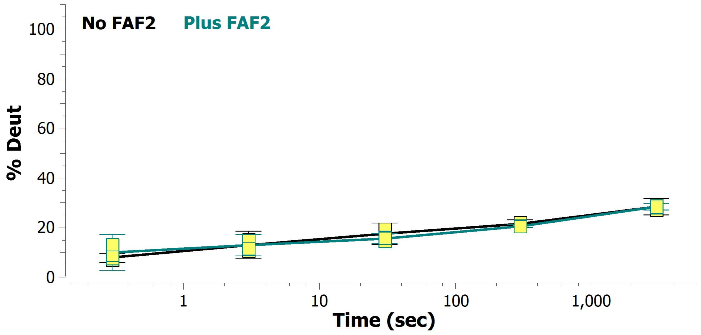

TERA 626-644: PDIIDPAILRPGRLDQLIY (#379)

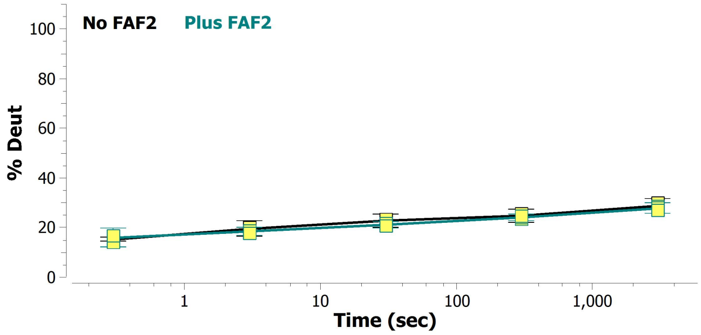

TERA 631-639: PAILRPGRL (#380)

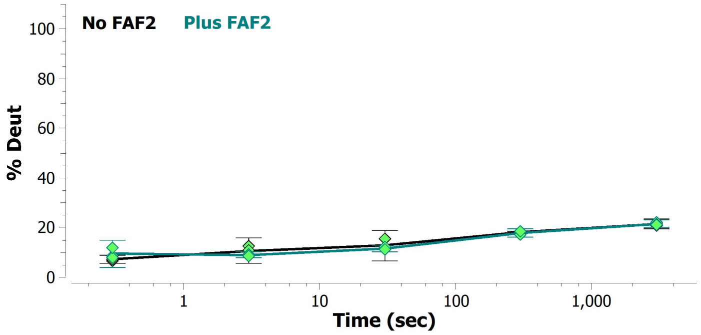

TERA 631-642: PAILRPGRLDQL (#381)

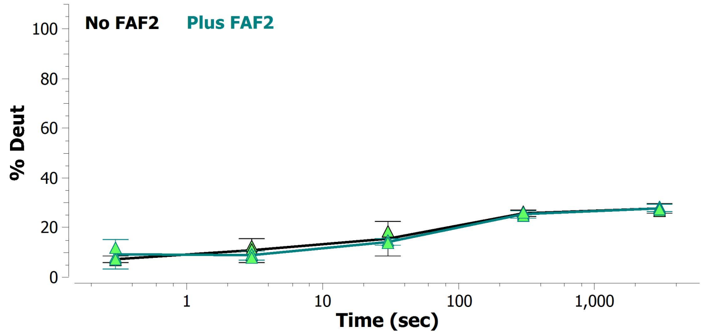

TERA 632-639: AILRPGRL (#382)

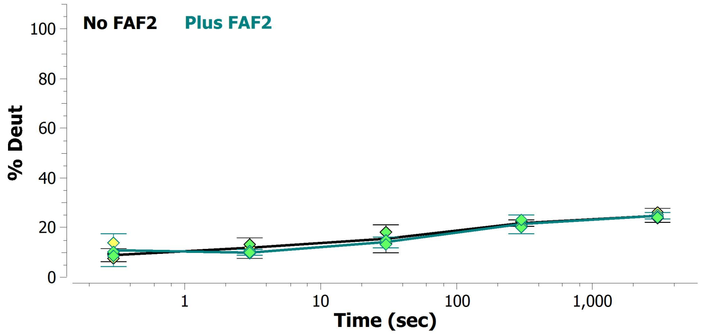

TERA 642-656: LIYIPLPDEKSRVAI (#383)

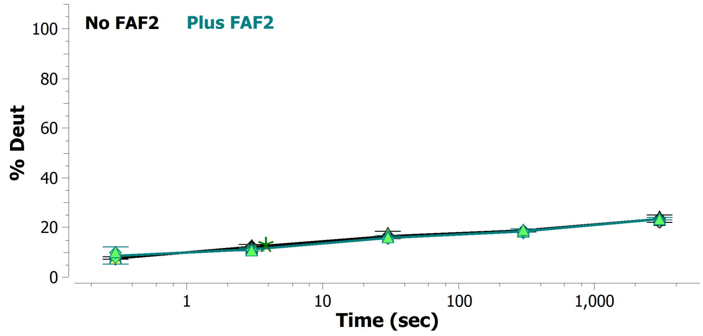

TERA 642-657: LIYIPLPDEKSRVAIL (#384)

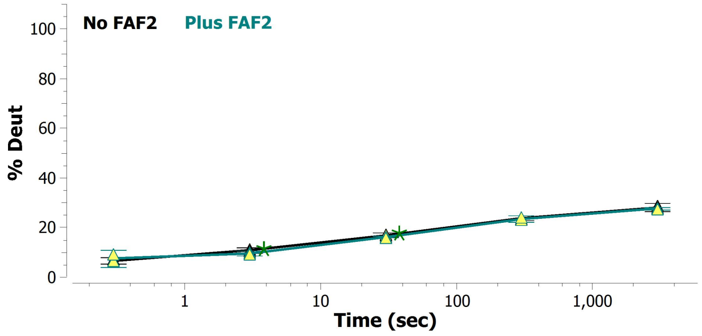

TERA 643-652: IYIPLPDEKS (#385)

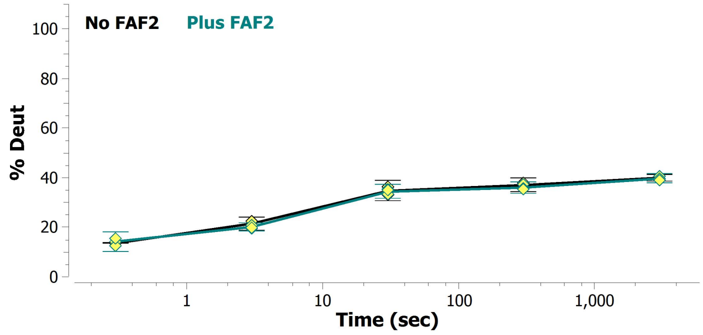

TERA 643-654: IYIPLPDEKSRV (#386)

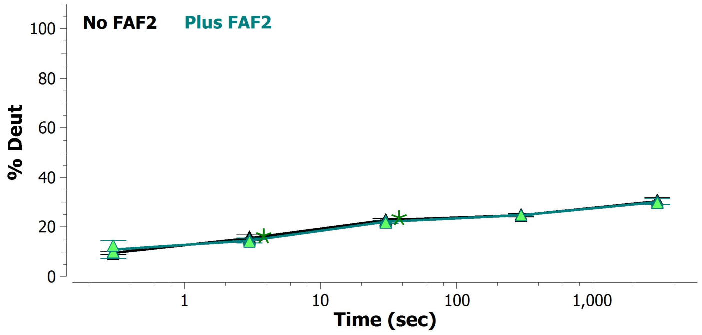

TERA 643-657: IYIPLPDEKSRVAIL (#387)

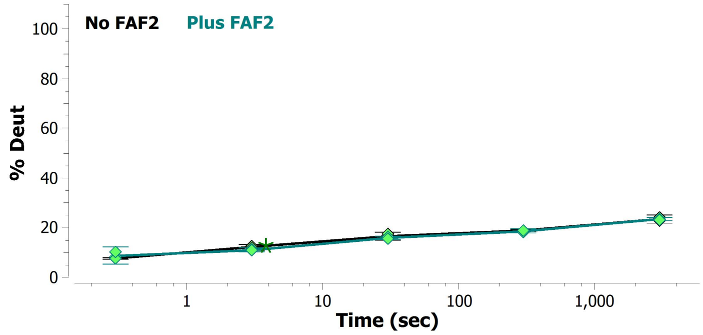

TERA 645-656: IPLPDEKSRVAI (#388)

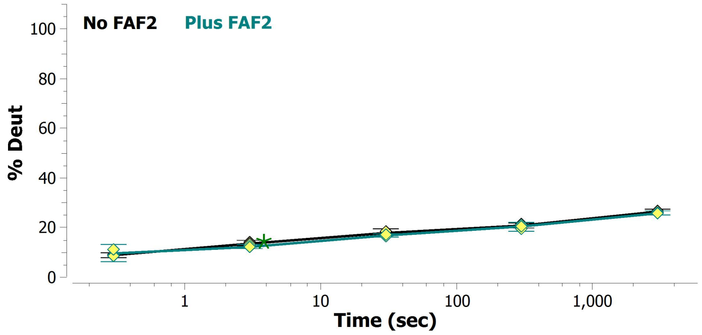

TERA 645-657: IPLPDEKSRVAIL (#389)

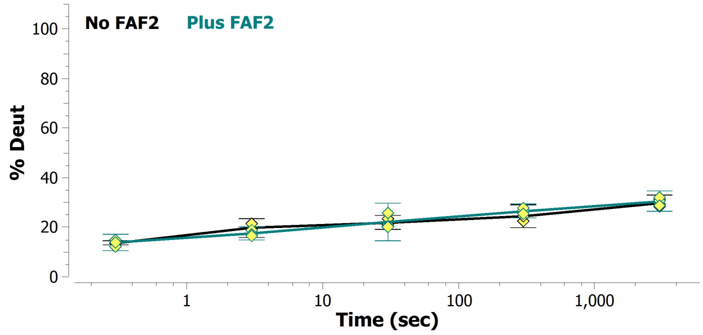

TERA 646-657: PLPDEKSRVAIL (#390)

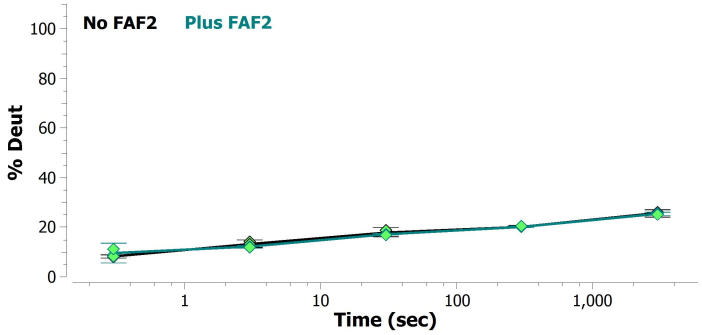

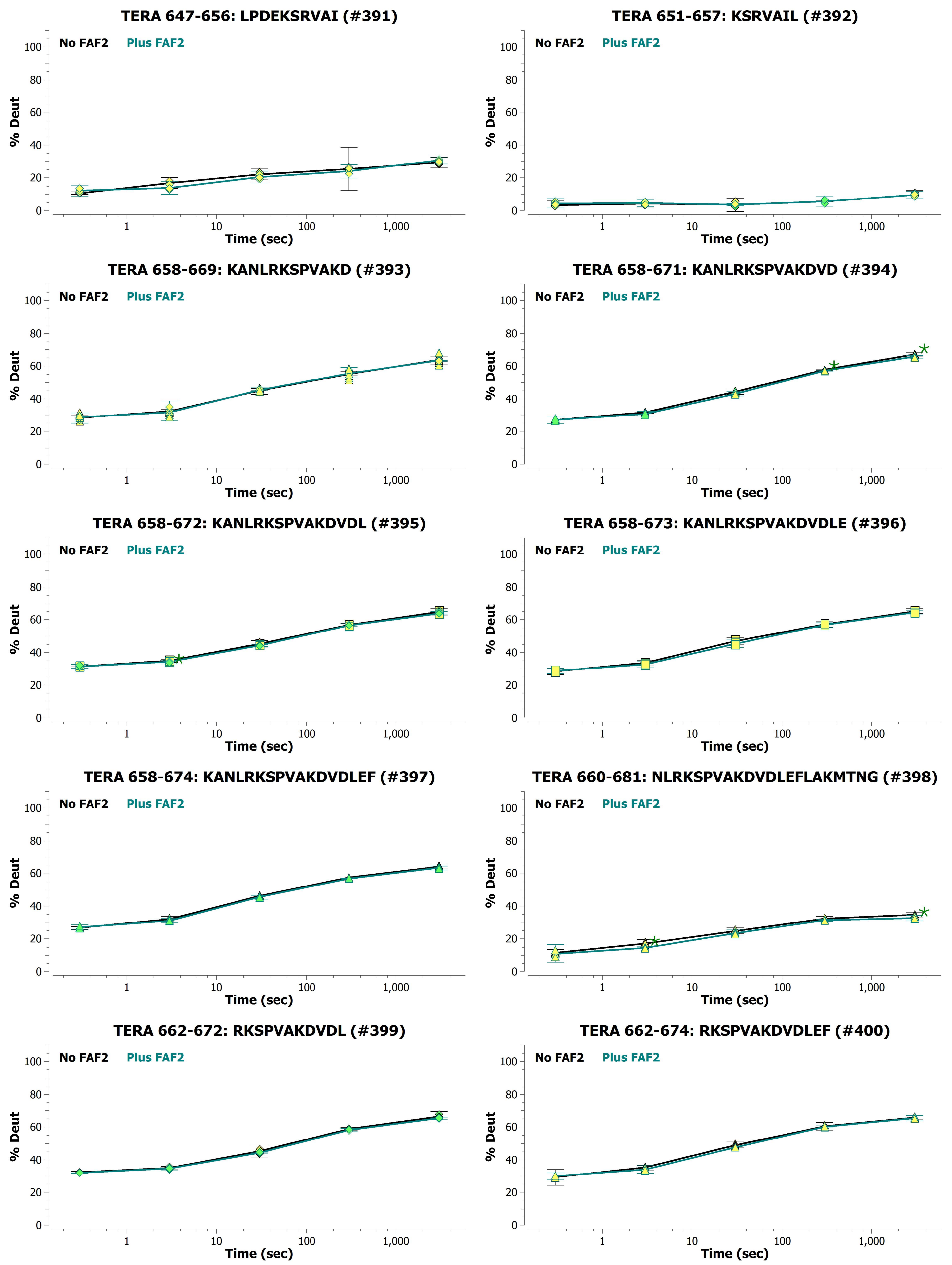

TERA 647-656: LPDEKSRVAI (#391)
TERA 651-657: KSRVAIL (#392)
TERA 658-669: KANLRKSPVAKD (#393)
TERA 658-671: KANLRKSPVAKDVD (#394)
TERA 658-672: KANLRKSPVAKDVDL (#395)
TERA 658-673: KANLRKSPVAKDVDLE (#396)
TERA 658-674: KANLRKSPVAKDVDLEF (#397)
TERA 660-681: NLRKSPVAKDVDLEFLAKMTNG (#398)
TERA 662-672: RKSPVAKDVDL (#399)
TERA 662-674: RKSPVAKDVDLEF (#400)
No FAF2
Plus FAF2
% Deut
Time (sec)
0
20
40
60
80
100
1
10
100
1,000

TERA 666-674: VAKDVDLEF (#401)

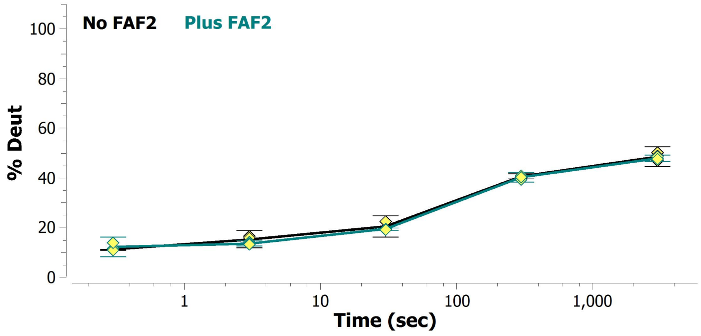

TERA 674-686: FLAKMTNGFSGAD (#402)

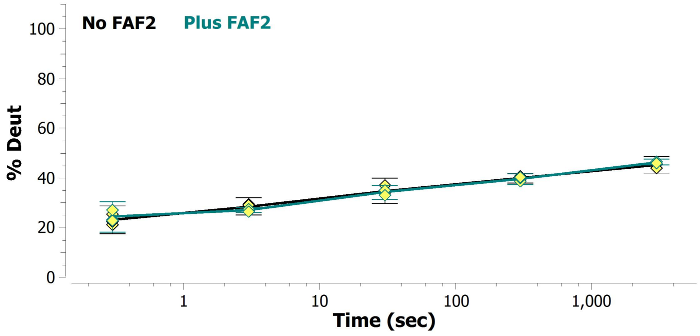

TERA 674-687: FLAKMTNGFSGADL (#403)

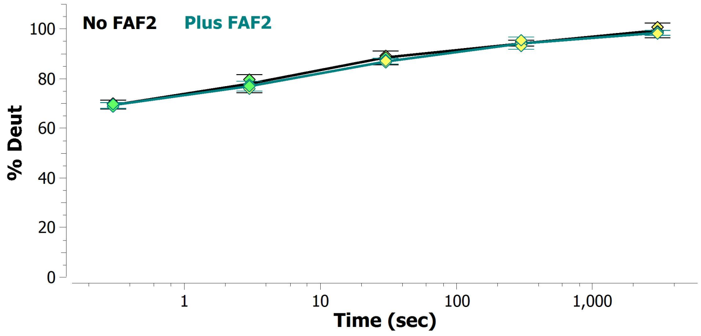

TERA 675-686: LAKMTNGFSGAD (#404)

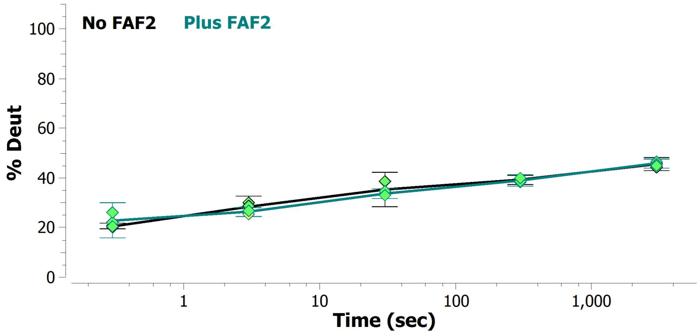

TERA 676-687: AKMTNGFSGADL (#405)

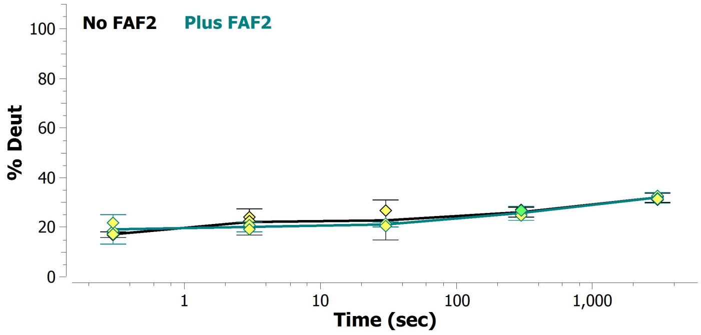

TERA 676-690: AKMTNGFSGADLTEI (#406)

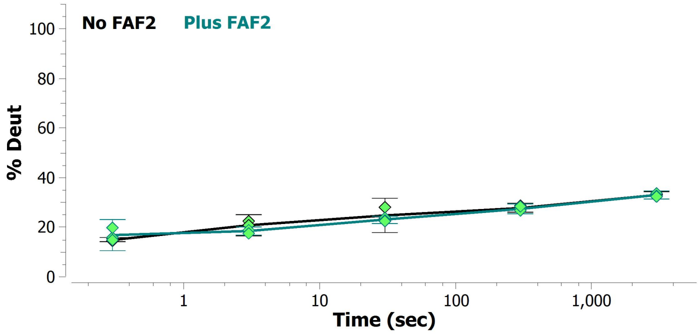

TERA 685-705: ADLTEICQRACKLAIRESIES (#407)

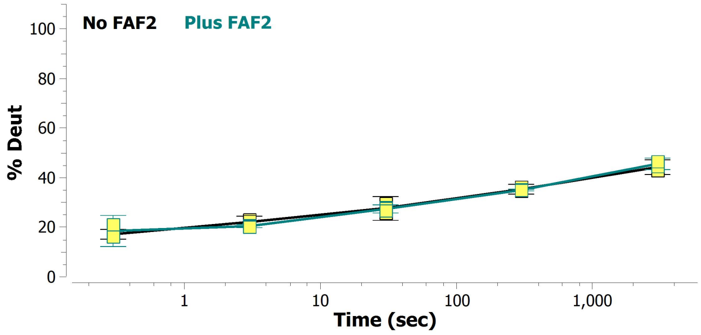

TERA 686-702: DLTEICQRACKLAIRES (#408)

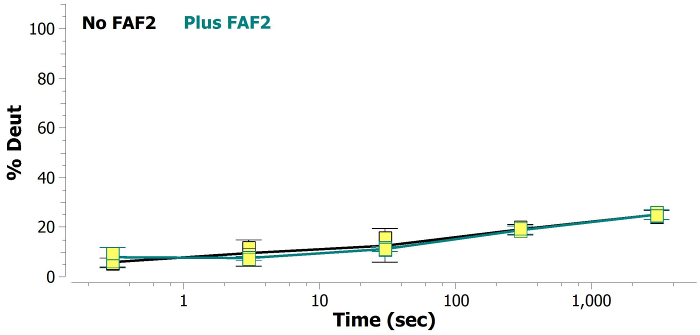

TERA 688-697: TEICQRACKL (#409)

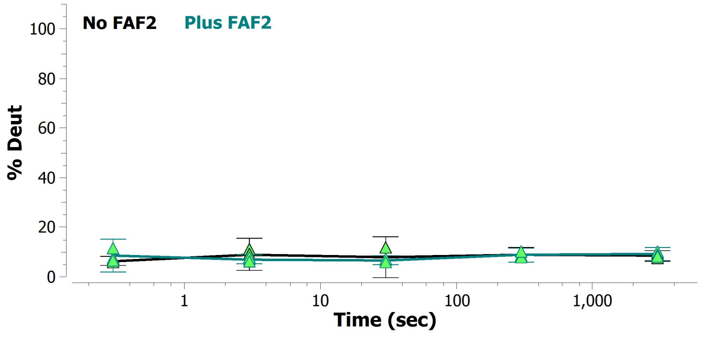

TERA 689-720:
EICQRACKLAIRESIESEIRRERERQTNPSAM (#410)

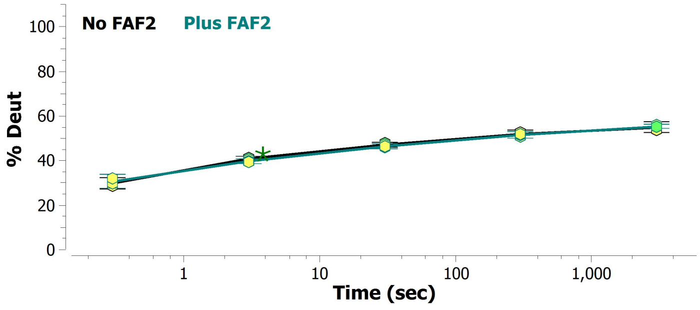

TERA 694-711: ACKLAIRESIESEIRRER (#411)

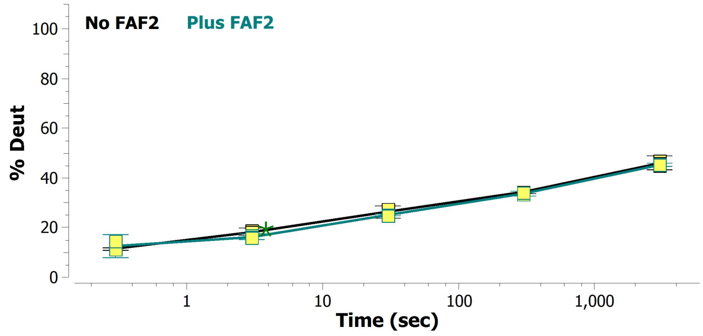

TERA 698-720: AIRESIESEIRRERERQTNPSAM (#412)

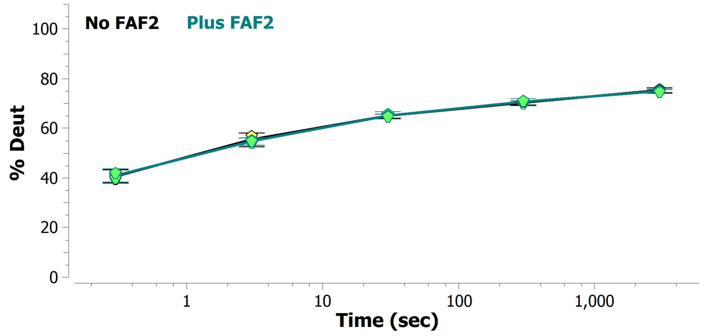

TERA 702-720: SIESEIRRERERQTNPSAM (#413)

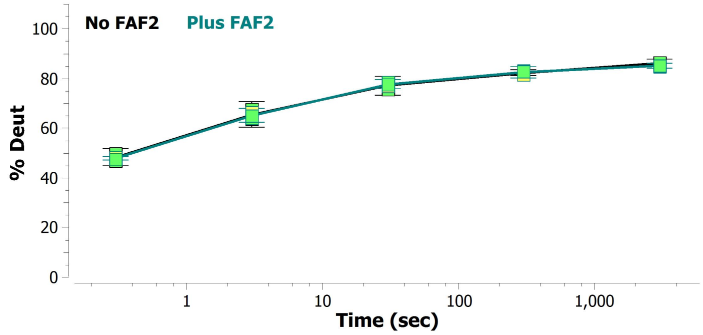

TERA 705-719: SEIRRERERQTNPSA (#414)

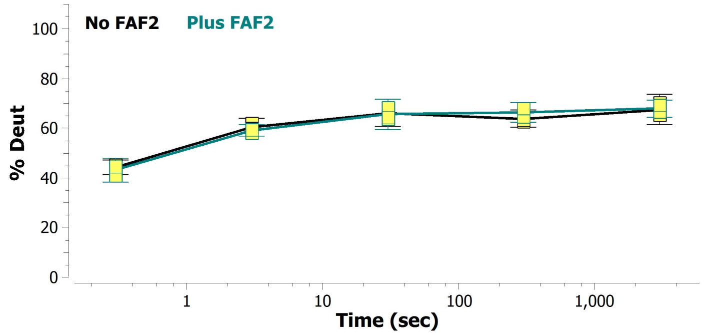

TERA 705-720: SEIRRERERQTNPSAM (#415)

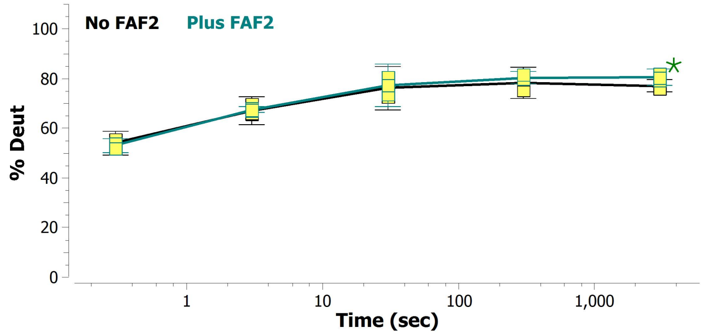

TERA 707-720: IRRERERQTNPSAM (#416)

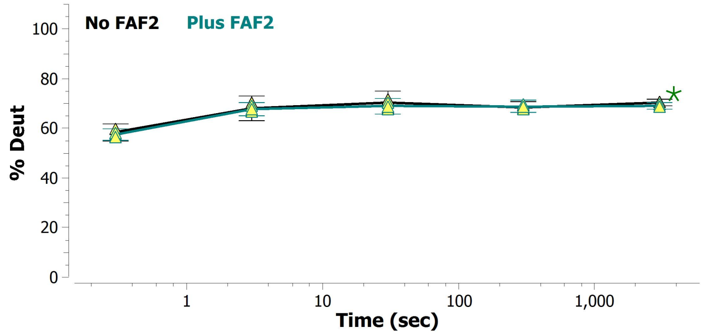

TERA 707-721: IRRERERQTNPSAME (#417)

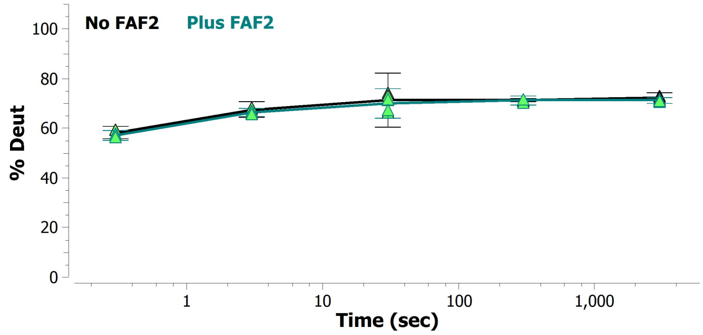

TERA 720-739: MEVEEDDPVPEIRRDHFEEA (#418)

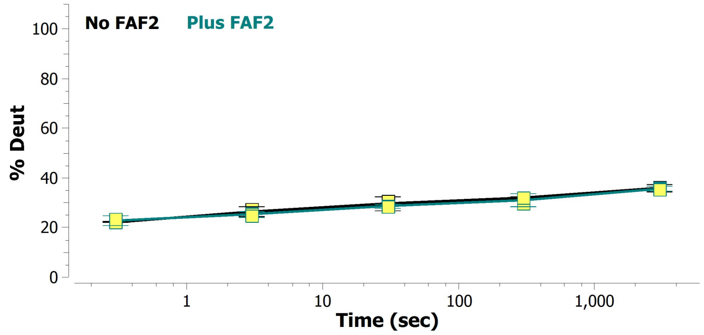

TERA 721-736: EVEEDDPVPEIRRDHF (#419)

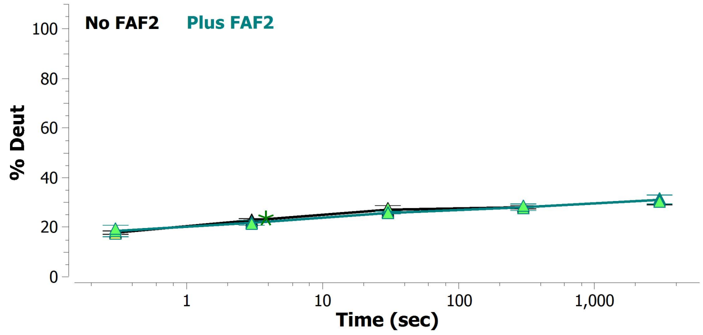

TERA 721-738: EVEEDDPVPEIRRDHFEE (#420)

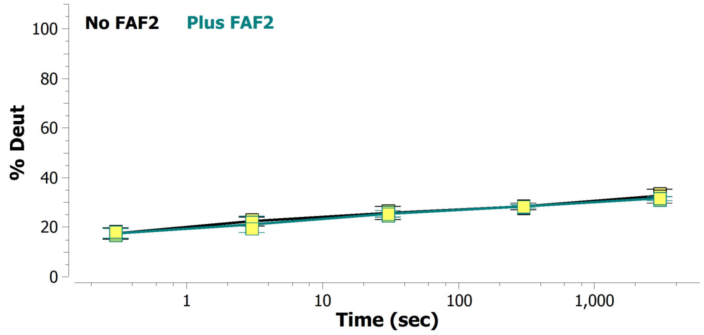

TERA 721-739: EVEEDDPVPEIRRDHFEEA (#421)

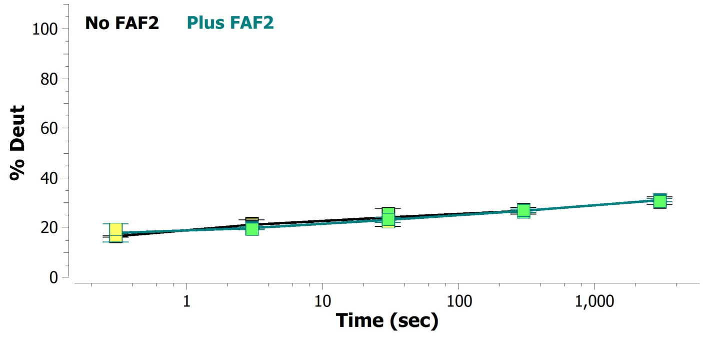

TERA 721-740: EVEEDDPVPEIRRDHFEEAM (#422)

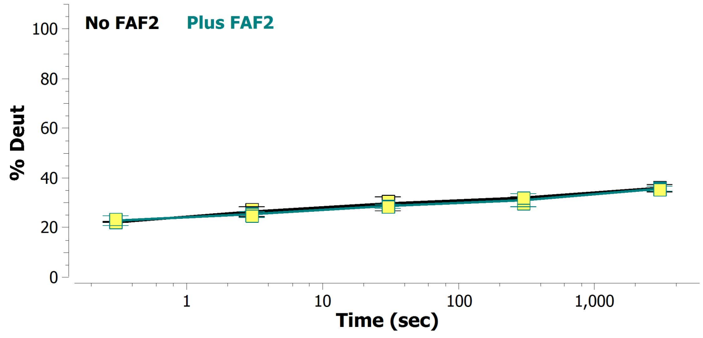

TERA 722-736: VEEDDPVPEIRRDHF (#423)

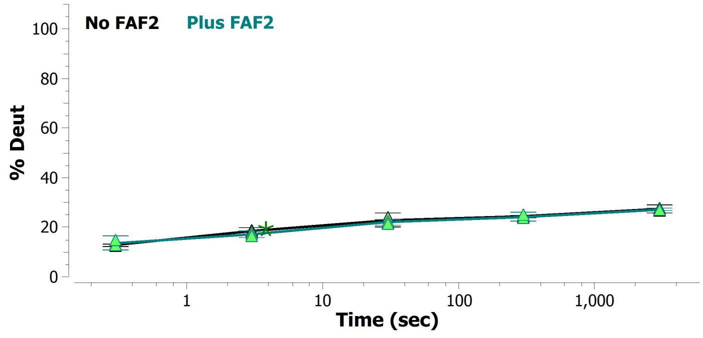

TERA 722-738: VEEDDPVPEIRRDHFEE (#424)

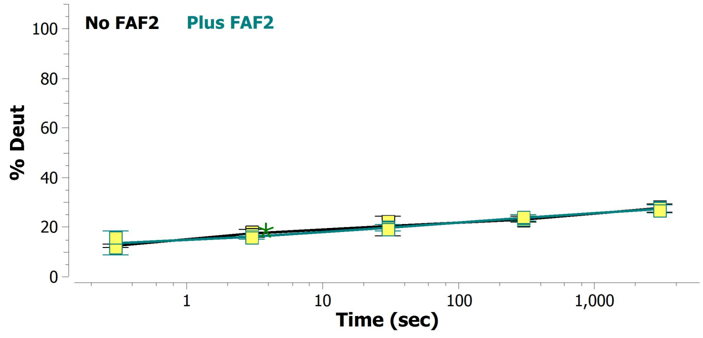

TERA 722-739: VEEDDPVPEIRRDHFEEA (#425)

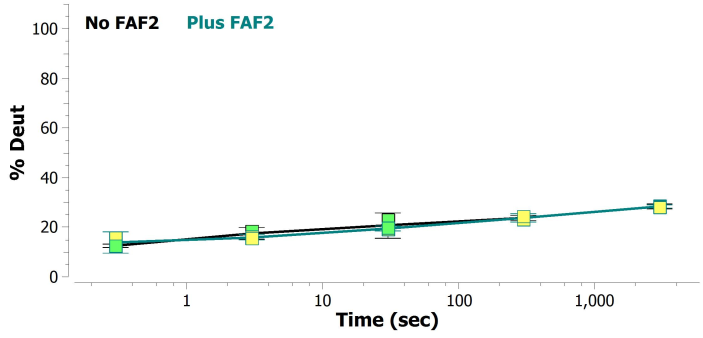

TERA 723-739: EEDDPVPEIRRDHFEEA (#426)

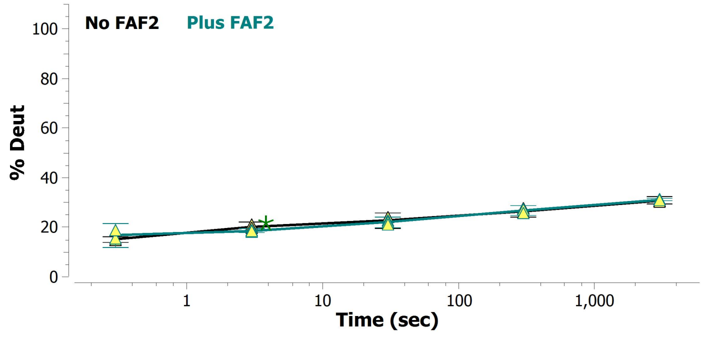

TERA 724-739: EDDPVPEIRRDHFEEA (#427)

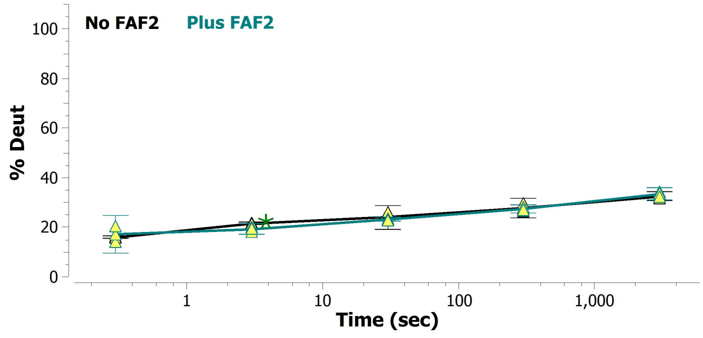

TERA 729-736: PEIRRDHF (#428)

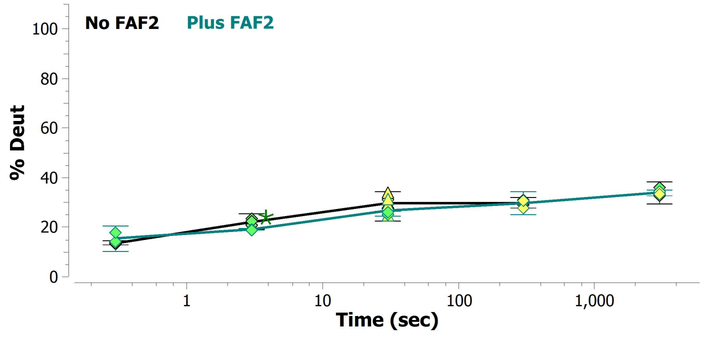

TERA 737-751: EEAMRFARRSVSDND (#429)

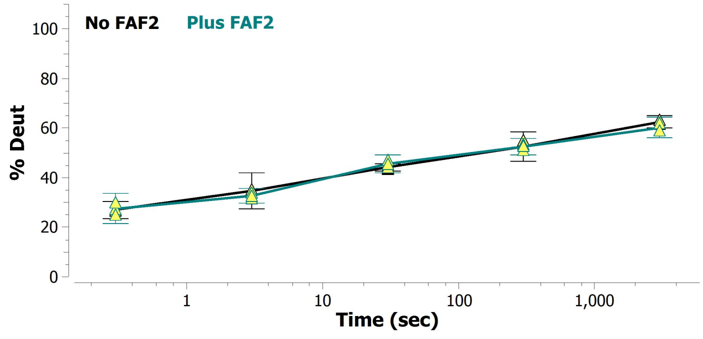

TERA 737-757: EEAMRFARRSVSDNDIRKYEM (#430)

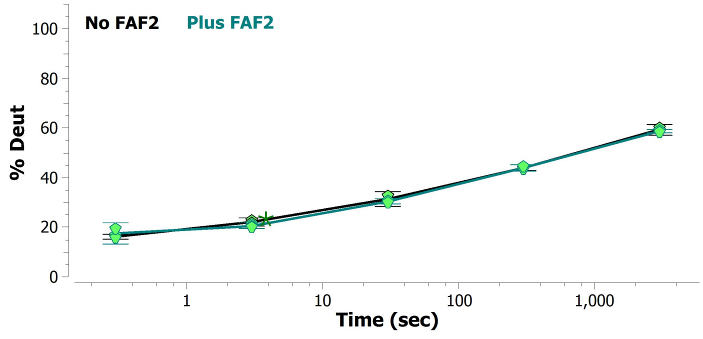

TERA 739-757: AMRFARRSVSDNDIRKYEM (#431)

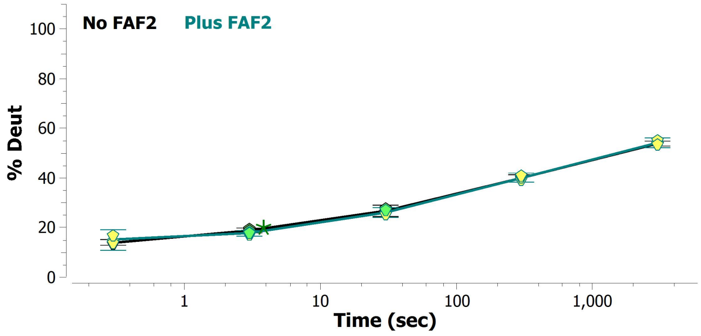

TERA 740-756: MRFARRSVSDNDIRKYE (#432)

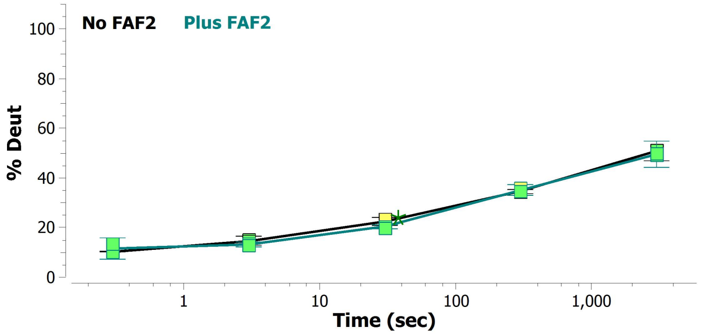

TERA 740-757: MRFARRSVSDNDIRKYEM (#433)

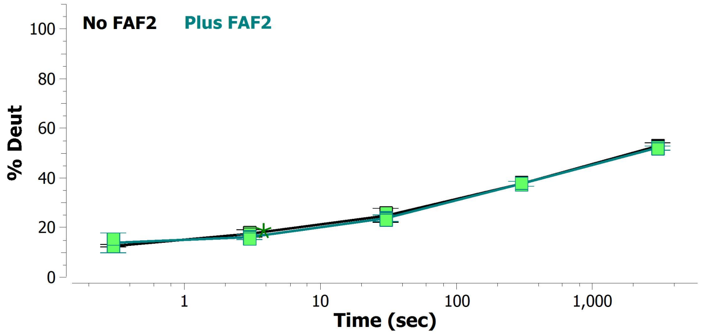

TERA 740-758: MRFARRSVSDNDIRKYEMF (#434)

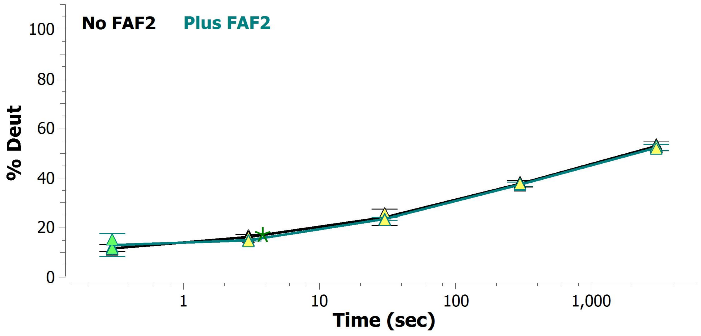

TERA 741-757: RFARRSVSDNDIRKYEM (#435)

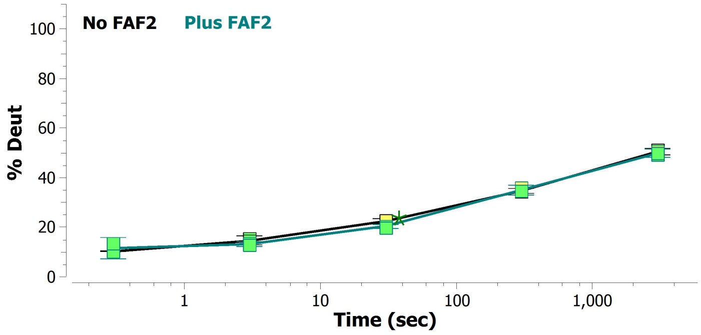

TERA 743-751: ARRSVSDND (#436)

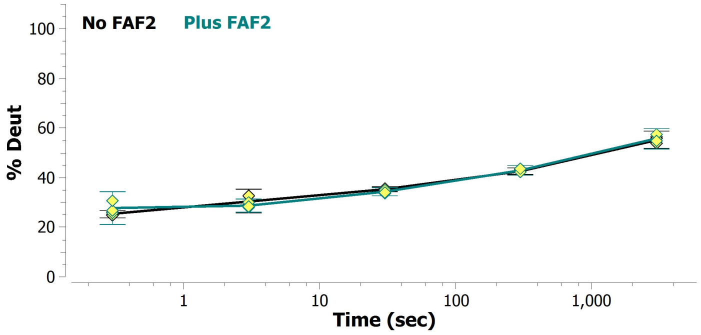

TERA 743-757: ARRSVSDNDIRKYEM (#437)

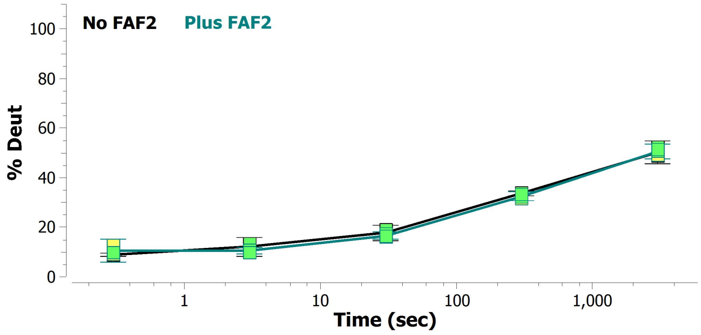

TERA 743-758: ARRSVSDNDIRKYEMF (#438)

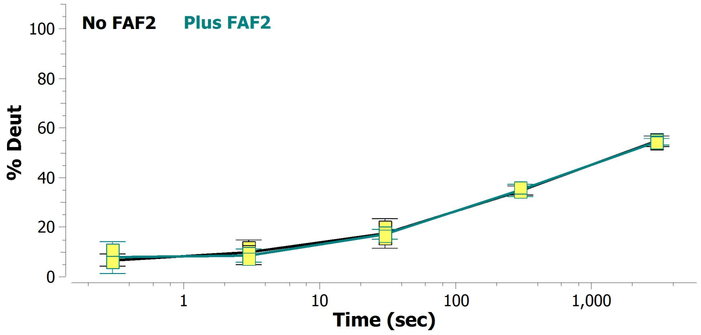

TERA 751-757: DIRKYEM (#439)

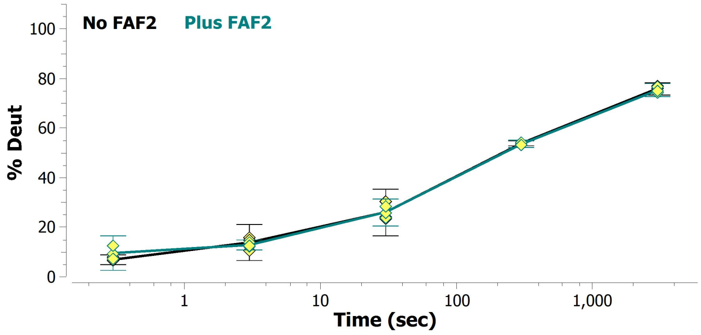

TERA 752-758: IRKYEMF (#440)

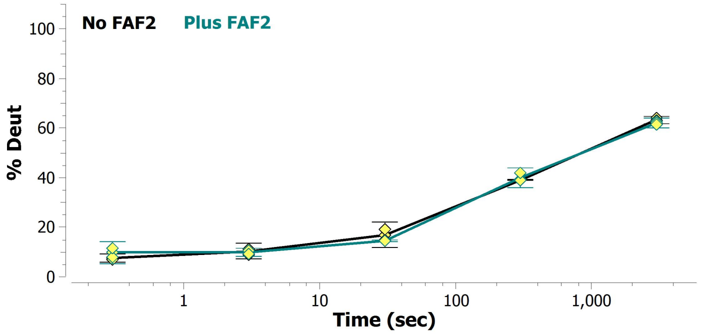

TERA 758-770: FAQTLQQSRGFGS (#441)

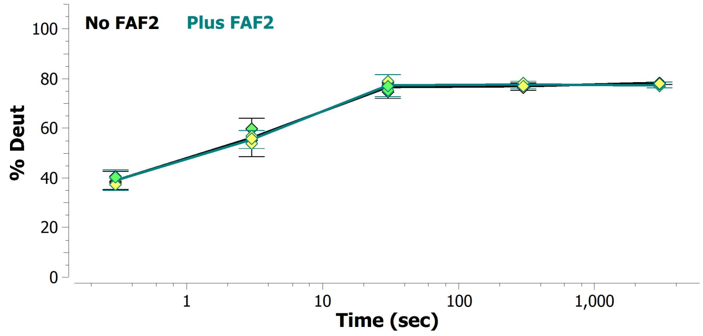

TERA 765-795: SRGFGSFRFPSGNQGGAGPSQGSGGGTGGSV (#442)

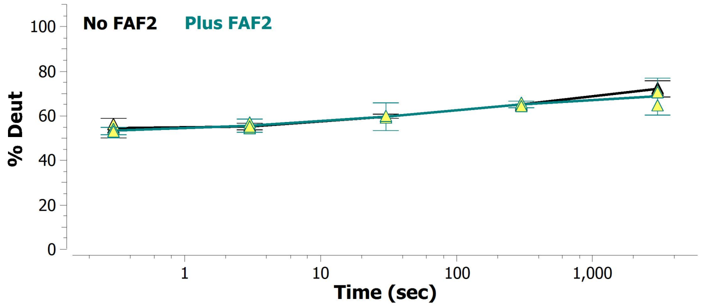

TERA 771-796: FRFPSGNQGGAGPSQGSGGGTGGSVY (#443)

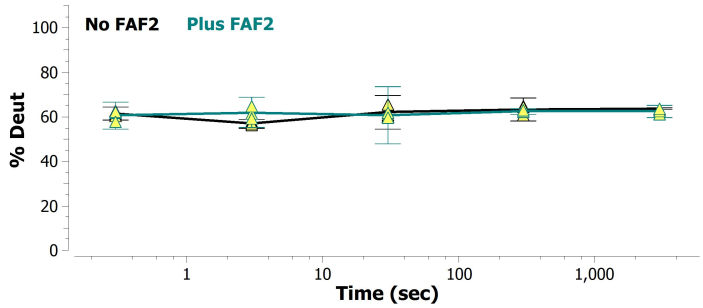

TERA 784-804: SQGSGGGTGGSVYTEDNDDDL (#444)

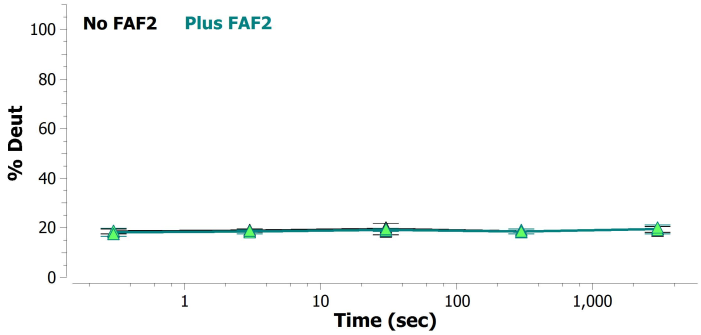

TERA 797-804: TEDNDDDL (#445)

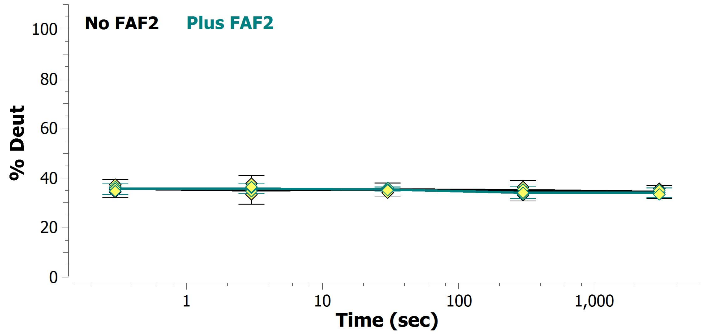

Supplement: Supplementary file 11 [file 44318_2026_894_MOESM6_ESM.zip › Figure 4/Fig 4C/p97 Complex uptake plots.pdf]
