# Supplementary material for: FAF1 and FAF2 enhance unfolding by p97-UFD1-NPL4 complex enabling rational design of p97 activators
Source: EMBO J. 2026 Aug 15;45(18):6595–629. doi: 10.1038/s44318-026-00894-x (PMC13578712; doi:10.1038/s44318-026-00894-x)
Supplement: Supplementary file 11 [file 44318_2026_894_MOESM6_ESM.zip › Figure 4/Fig 4E/FAF2 uptake plots.pdf]

Q96CS3 1-12: MAAPEERDLTQE (#1)

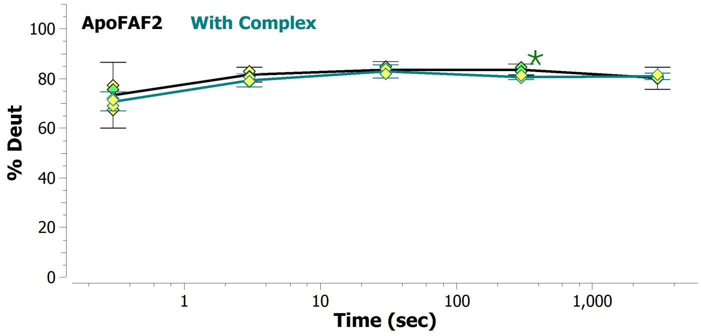

Q96CS3 1-17: MAAPEERDLTQEQTEKL (#2)

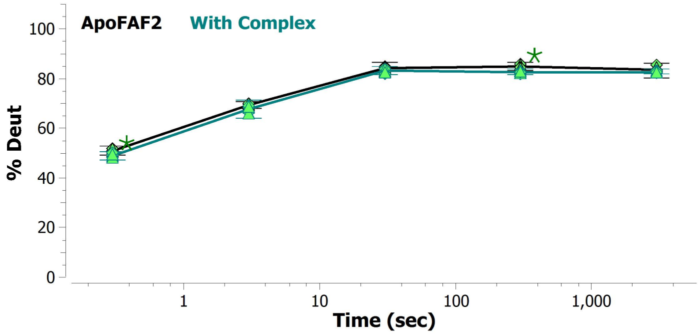

Q96CS3 1-19: MAAPEERDLTQEQTEKLLQ (#3)

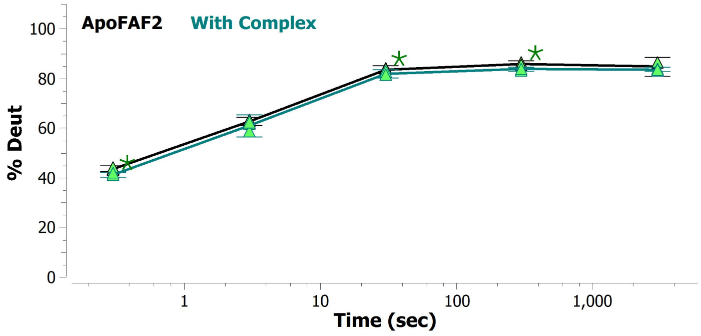

Q96CS3 1-20: MAAPEERDLTQEQTEKLLQF (#4)

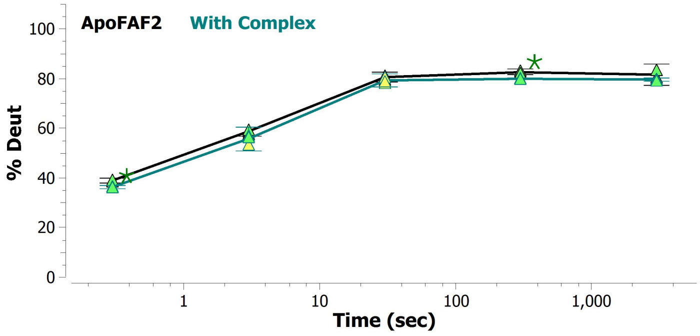

Q96CS3 1-23: MAAPEERDLTQEQTEKLLQFQDL (#5)

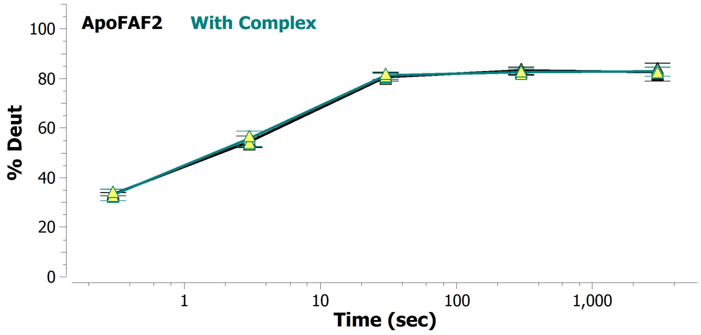

Q96CS3 1-29: MAAPEERDLTQEQTEKLLQFQDLTGIESM (#6)

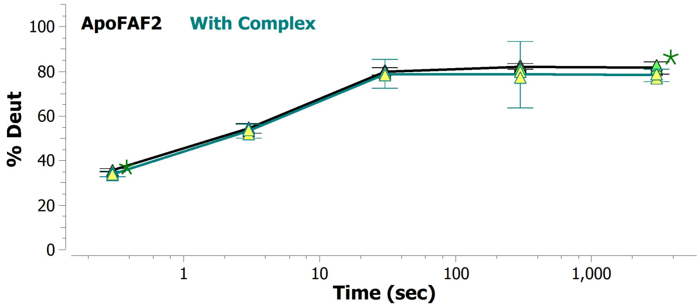

Q96CS3 1-44: [illegible]APEERDLTQEQTEKLLQFQDLTGIESMDQCRHTLEQHNWN (#7)

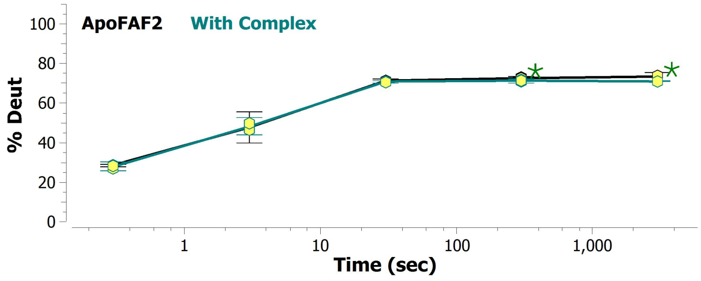

Q96CS3 3-17: APEERDLTQEQTEKL (#8)

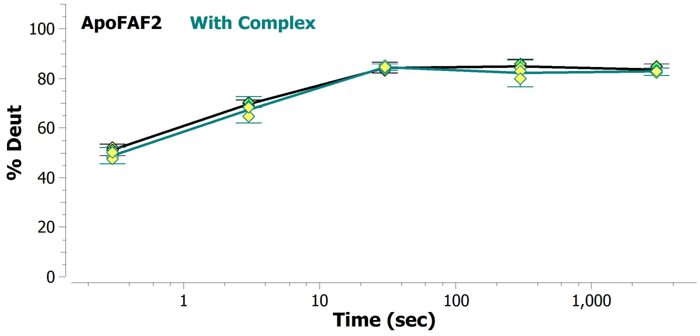

Q96CS3 4-17: PEERDLTQEQTEKL (#9)

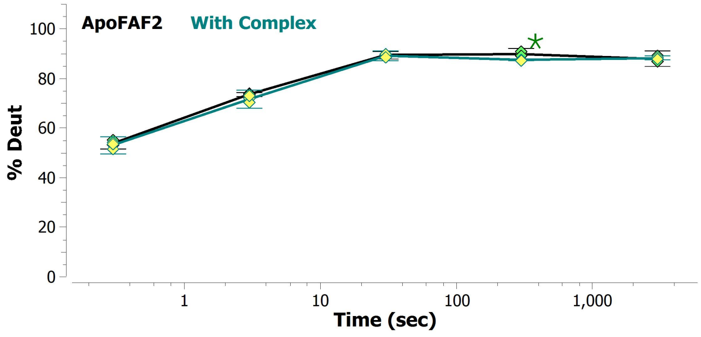

Q96CS3 18-29: LQFQDLTGIESM (#10)

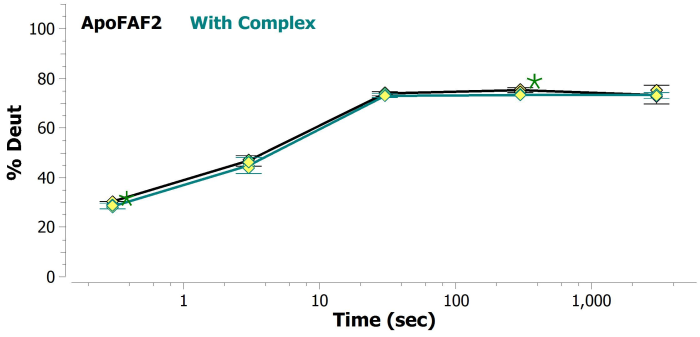

Q96CS3 18-44: LQFQDLTGIESMDQCRHTLEQHNWNIE (#11)

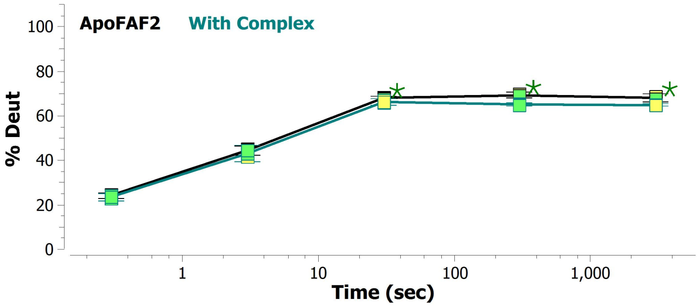

Q96CS3 18-45: LQFQDLTGIESMDQCRHTLEQHNWNIEA (#12)

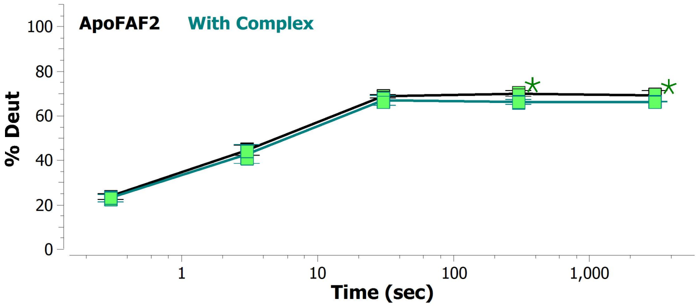

Q96CS3 20-44: FQDLTGIESMDQCRHTLEQHNWNIE (#13)

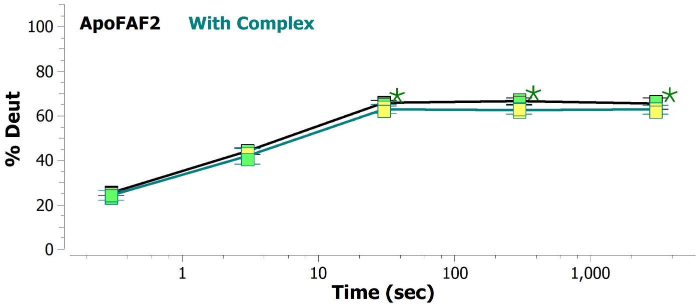

Q96CS3 21-45: QDLTGIESMDQCRHTLEQHNWNIEA (#14)

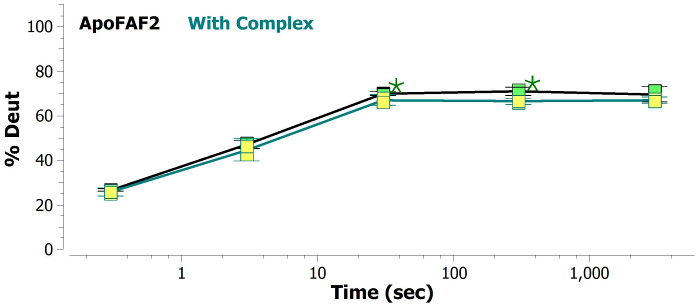

Q96CS3 24-44: TGIESMDQCRHTLEQHNWNIE (#15)

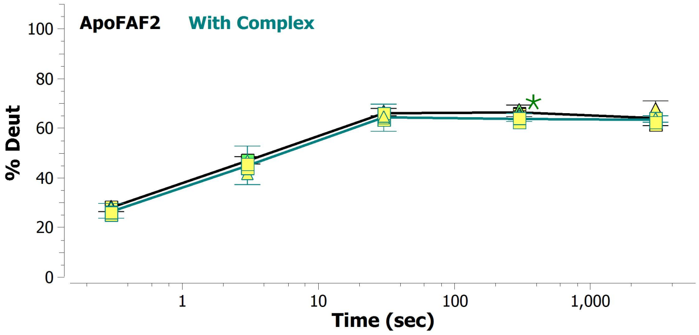

Q96CS3 26-45: IESMDQCRHTLEQHNWNIEA (#16)

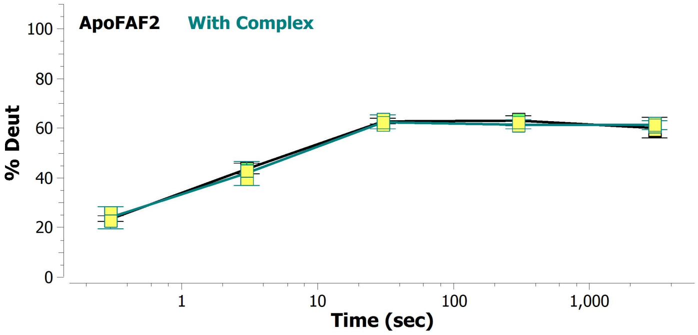

Q96CS3 30-44: DQCRHTLEQHNWNIE (#17)

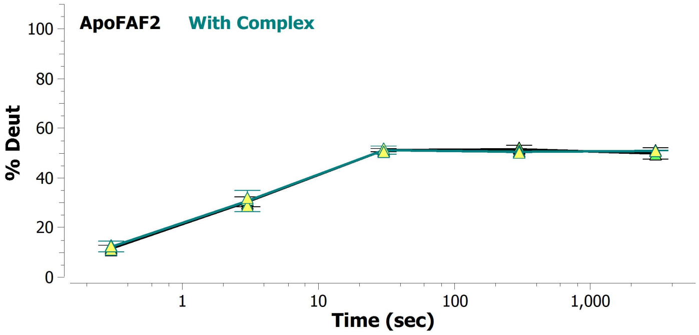

Q96CS3 30-45: DQCRHTLEQHNWNIEA (#18)

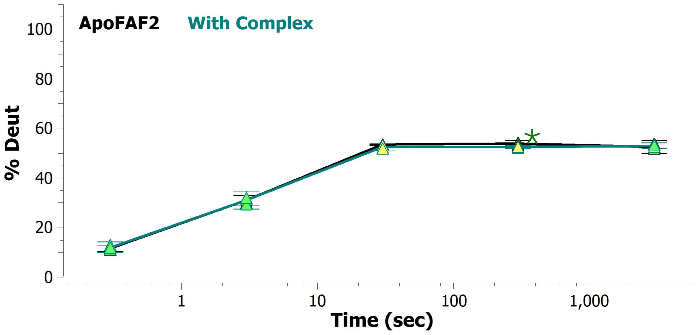

Q96CS3 30-46: DQCRHTLEQHNWNIEAA (#19)

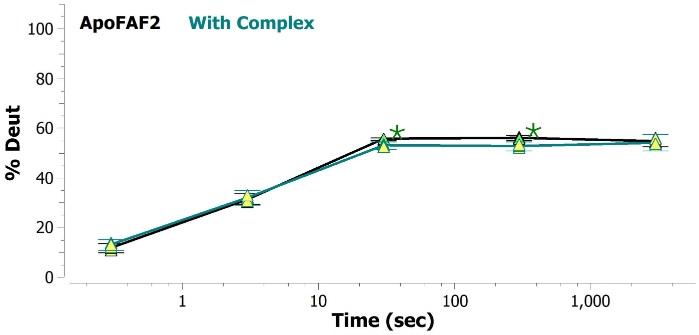

Q96CS3 45-80: AAVQDRLNEQEGVPSVFNPPPSRPLQVNTADHRIYS (#20)

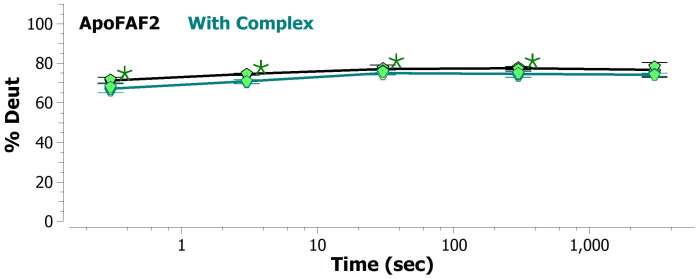

Q96CS3 45-81: AAVQDRLNEQEGVPSVFNPPPSRPLQVNTADHRIYSY (#21)

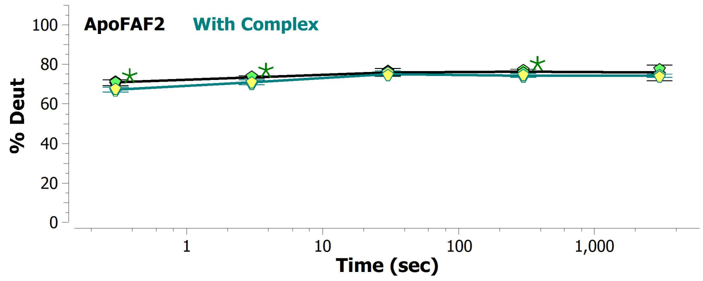

Q96CS3 46-80: AVQDRLNEQEGVPSVFNPPPSRPLQVNTADHRIYS (#22)

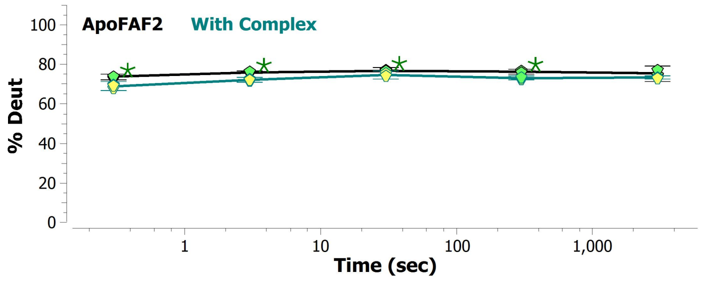

Q96CS3 52-80: NEQEGVPSVFNPPPSRPLQVNTADHRIYS (#23)

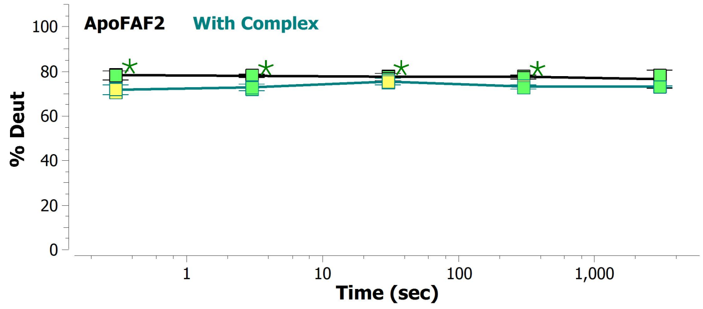

Q96CS3 139-153: FMHSFEEKYGRAHPV (#24)

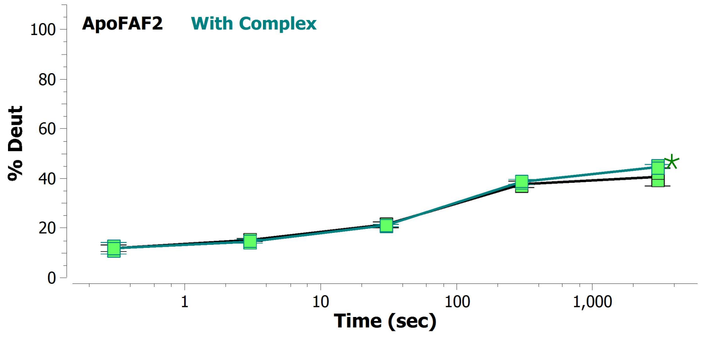

Q96CS3 140-154: MHSFEEKYGRAHPVF (#25)

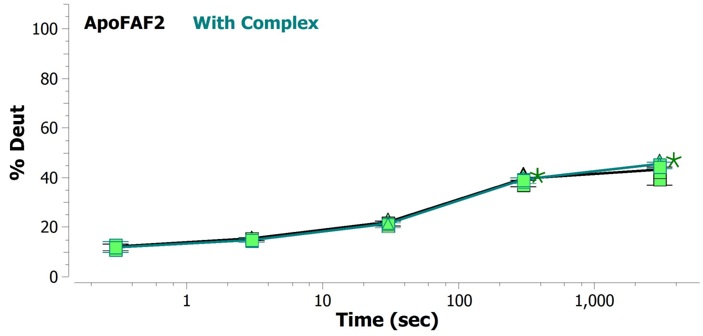

Q96CS3 140-174: MHSFEEKYGRAHPVFYQGTYSQALNDAKRELRFLL (#26)

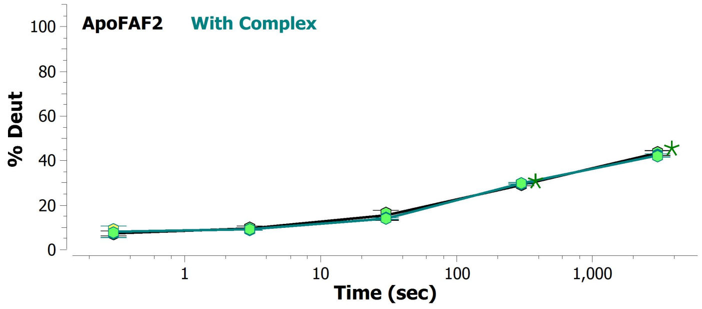

Q96CS3 141-154: HSFEEKYGRAHPVF (#27)

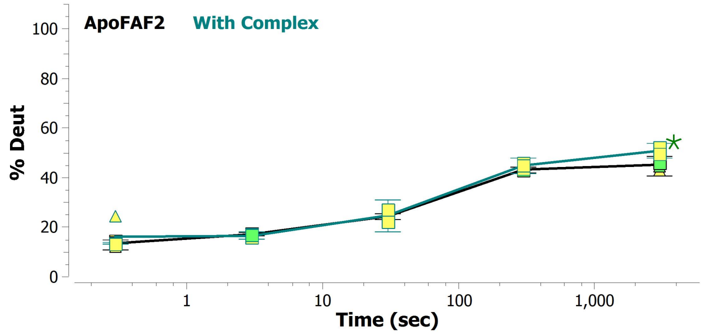

Q96CS3 143-154: FEEKYGRAHPVF (#28)

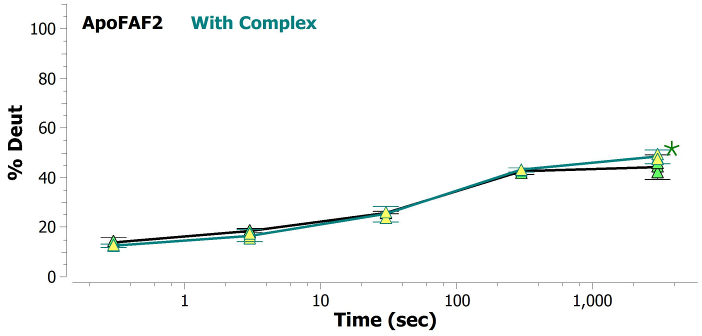

Q96CS3 144-154: EEKYGRAHPVF (#29)

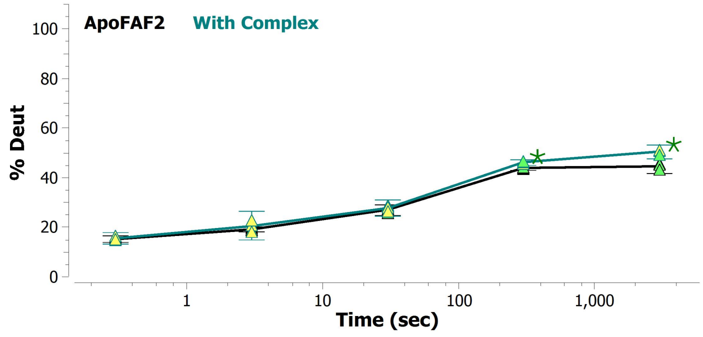

Q96CS3 155-172: YQGTYSQALNDAKRELRF (#30)

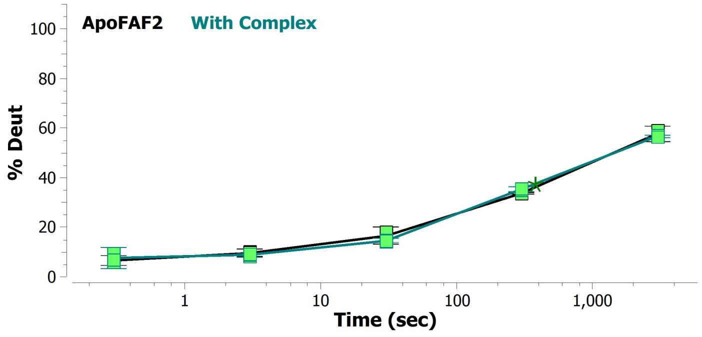

Q96CS3 155-174: YQGTYSQALNDAKRELRFLL (#31)

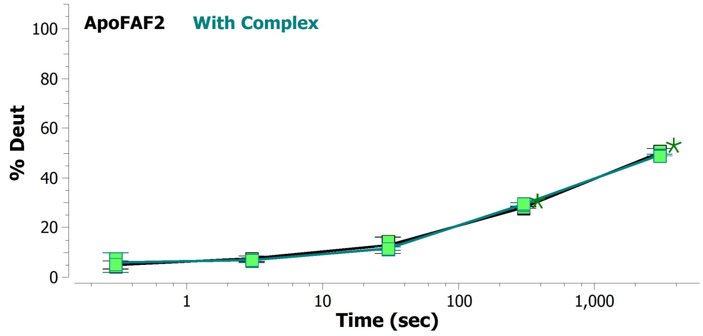

Q96CS3 155-175: YQGTYSQALNDAKRELRFLLV (#32)

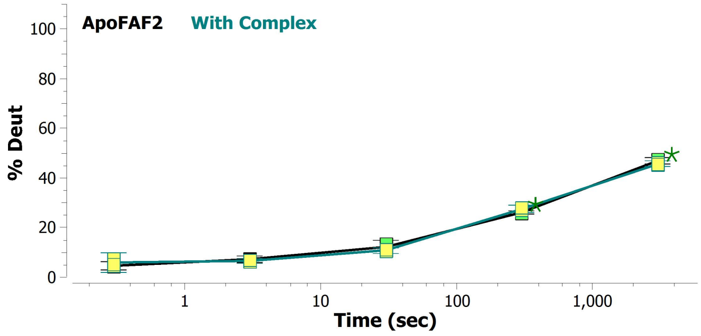

Q96CS3 163-174: LNDAKRELRFLL (#33)

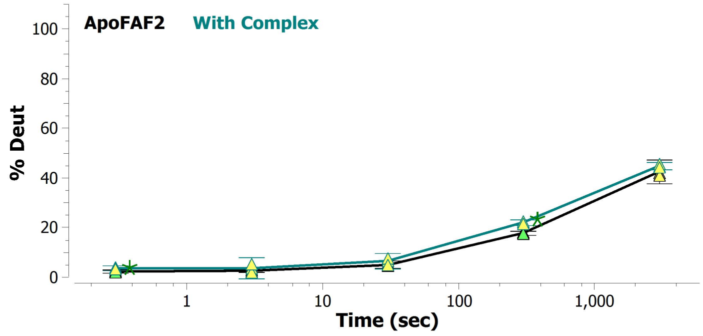

Q96CS3 164-174: NDAKRELRFLL (#34)

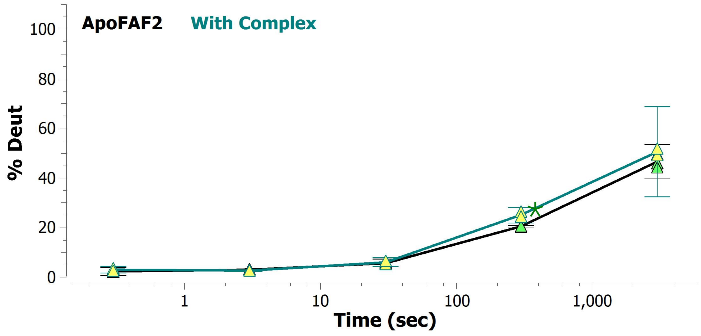

Q96CS3 166-174: AKRELRFLL (#35)

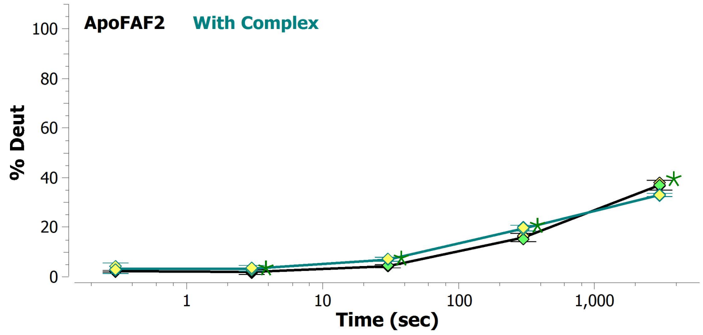

Q96CS3 173-187: LLVYLHGDDHQDSDE (#36)

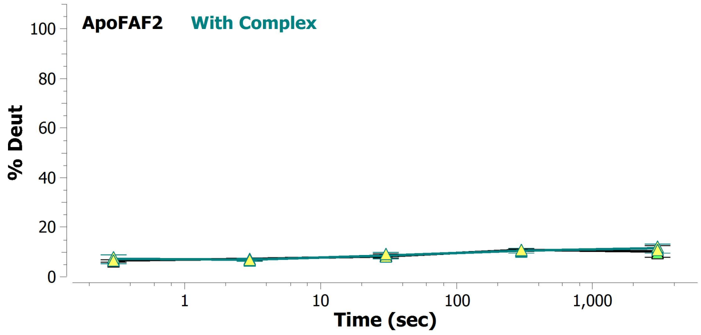

Q96CS3 173-188: LLVYLHGDDHQDSDEF (#37)

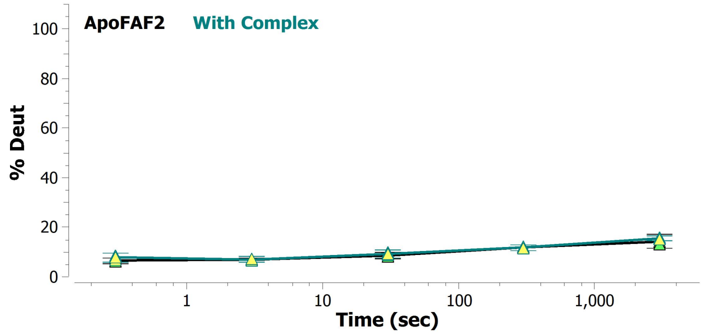

Q96CS3 175-187: VYLHGDDHQDSDE (#38)

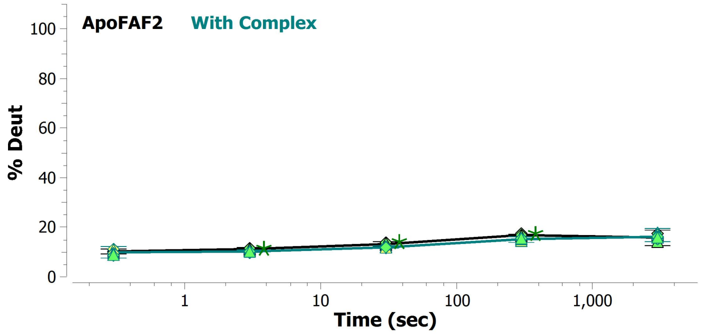

Q96CS3 175-188: VYLHGDDHQDSDEF (#39)

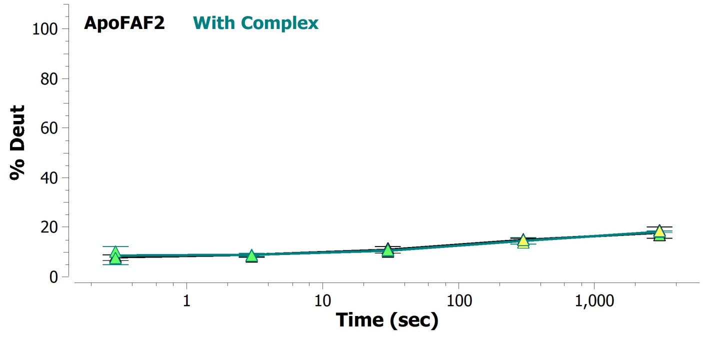

Q96CS3 175-201: VYLHGDDHQDSDEFCRNTLCAPEVISL (#40)

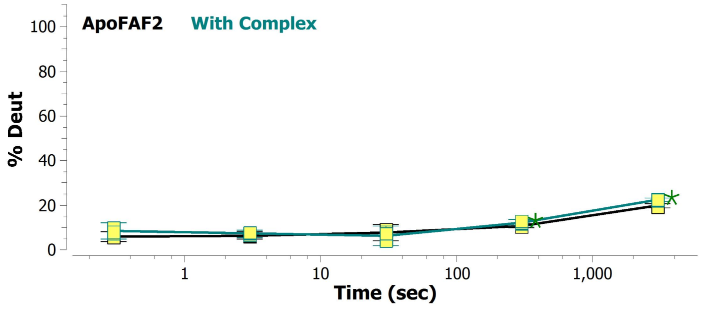

Q96CS3 176-187: YLHGDDHQDSDE (#41)

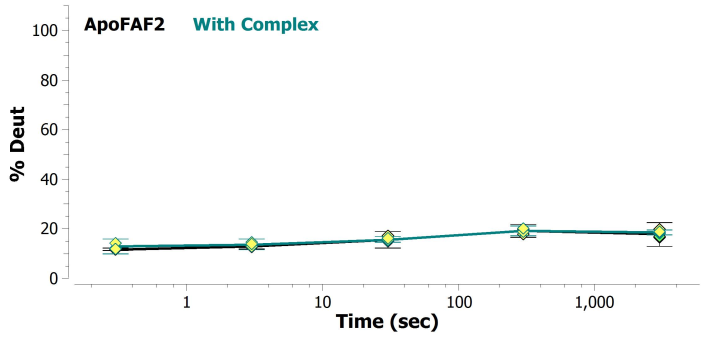

Q96CS3 176-188: YLHGDDHQDSDEF (#42)

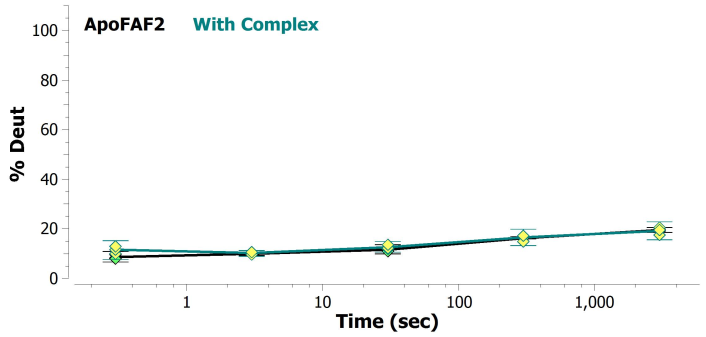

Q96CS3 187-211: EFCRNTLCAPEVISLINTRMLFWAC (#43)

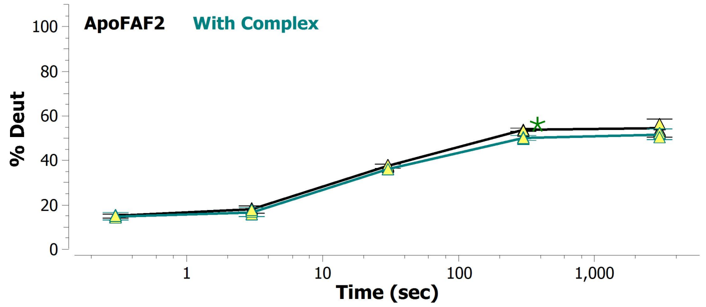

Q96CS3 188-195: FCRNTLCA (#44)

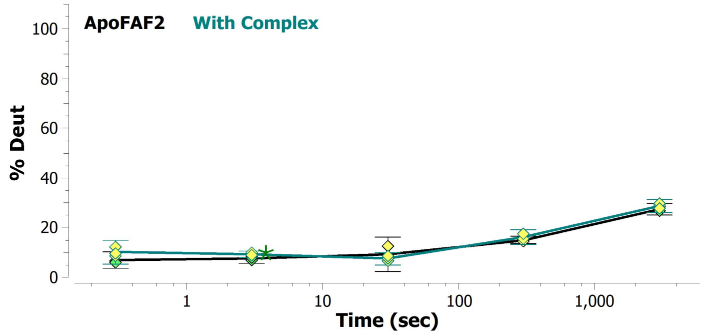

Q96CS3 188-198: FCRNTLCAPEV (#45)

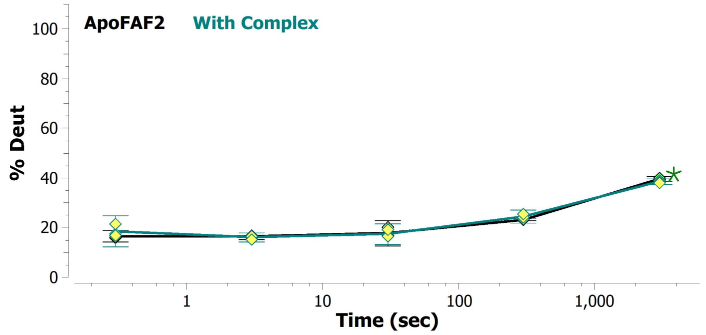

Q96CS3 188-200: FCRNTLCAPEVIS (#46)

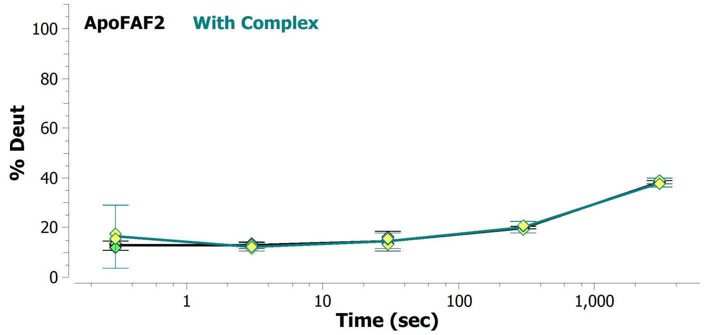

Q96CS3 188-201: FCRNTLCAPEVISL (#47)

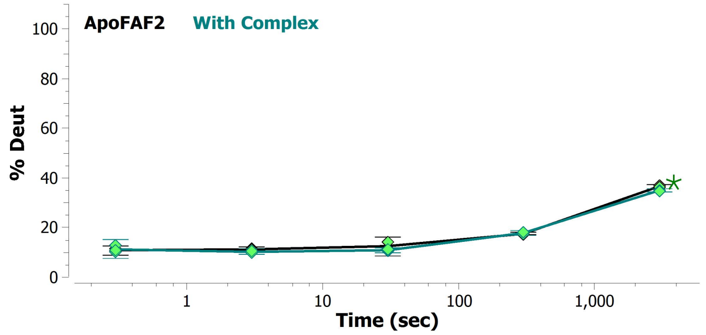

Q96CS3 189-201: CRNTLCAPEVISL (#48)

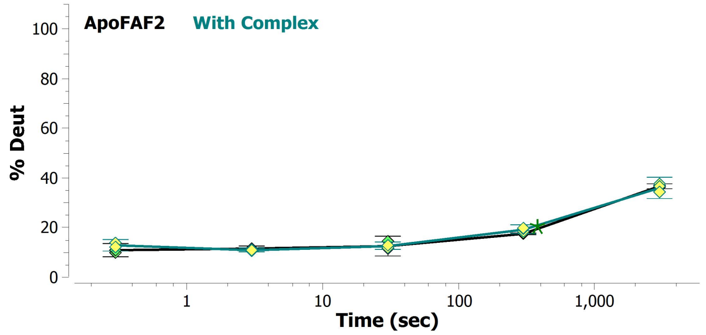

Q96CS3 201-208: LINTRMLF (#49)

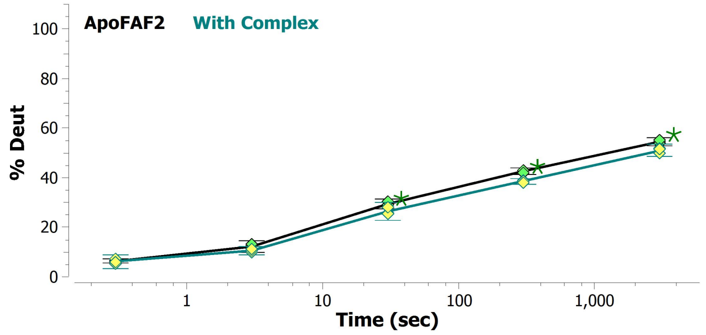

Q96CS3 202-208: INTRMLF (#50)

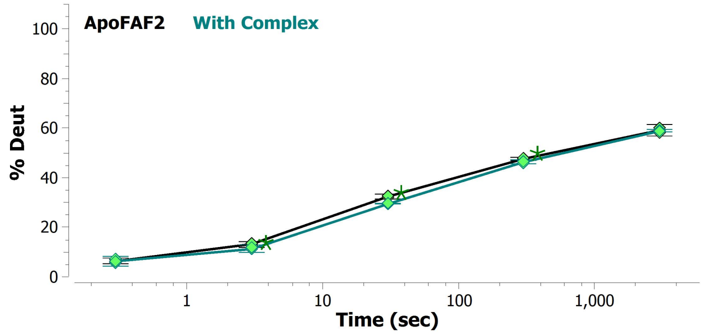

Q96CS3 203-208: NTRMLF (#51)

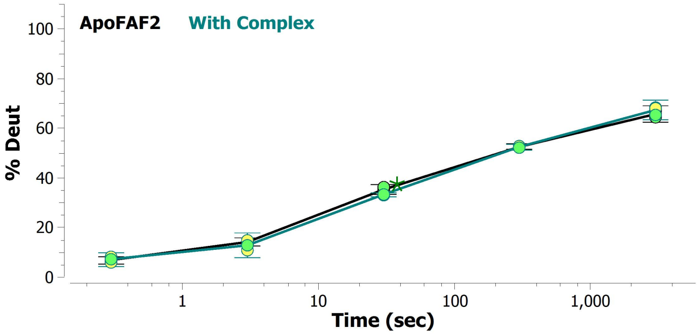

Q96CS3 207-233: LFWACSTNKPEGYRVSQALRENTYPFL (#52)

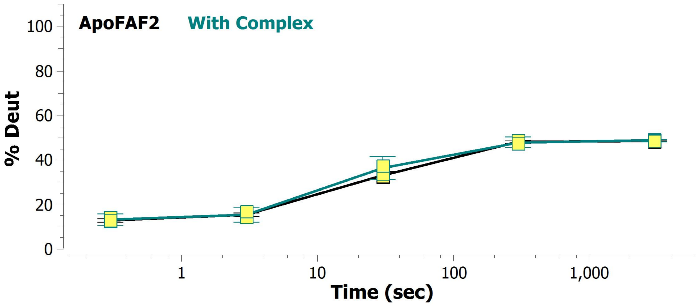

Q96CS3 207-235: LFWACSTNKPEGYRVSQALRENTYPFLAM (#53)

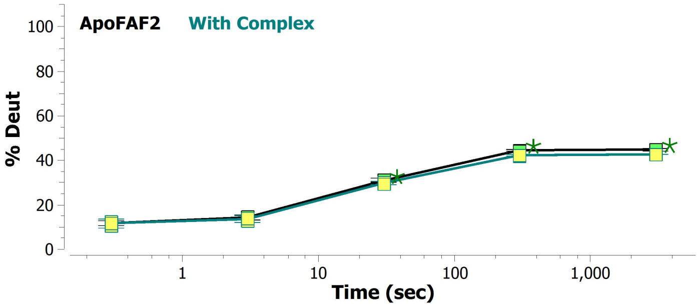

Q96CS3 209-223: WACSTNKPEGYRVSQ (#54)

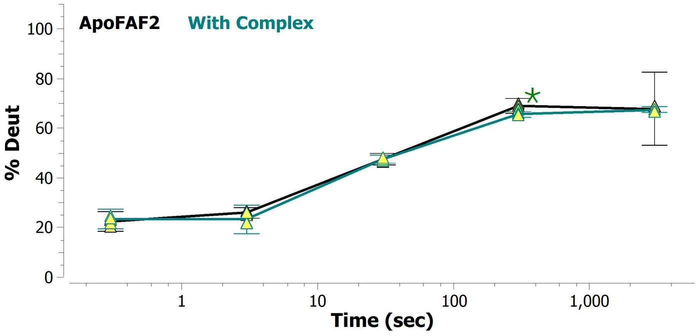

Q96CS3 209-224: WACSTNKPEGYRVSQA (#55)

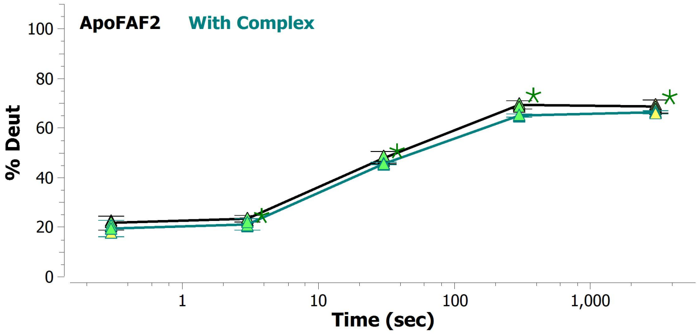

Q96CS3 209-225: WACSTNKPEGYRVSQAL (#56)

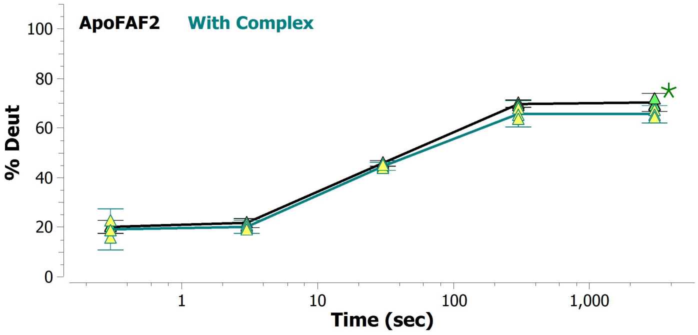

Q96CS3 209-227: WACSTNKPEGYRVSQALRE (#57)

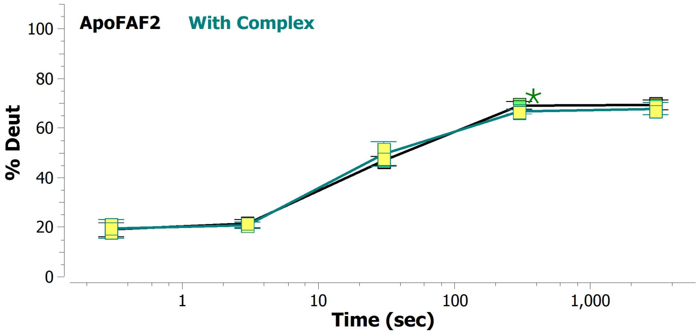

Q96CS3 209-235: WACSTNKPEGYRVSQALRENTYPFLAM (#58)

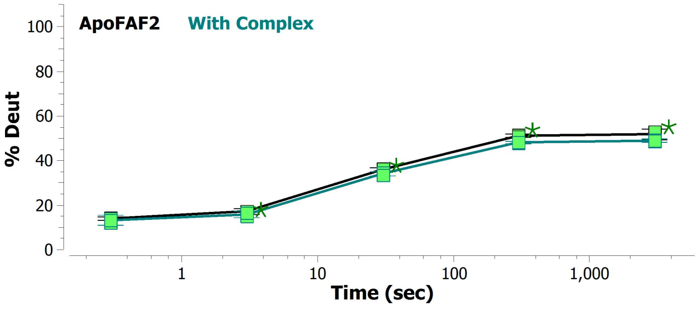

Q96CS3 219-235: YRVSQALRENTYPFLAM (#59)

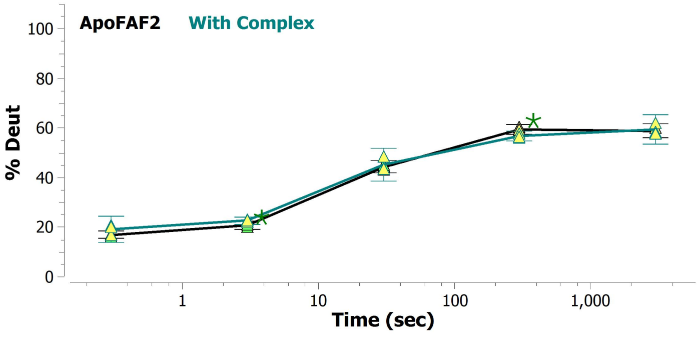

Q96CS3 224-235: ALRENTYPFLAM (#60)

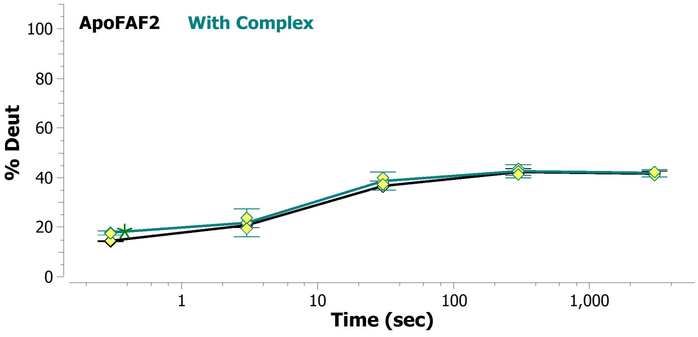

Q96CS3 225-235: LRENTYPFLAM (#61)

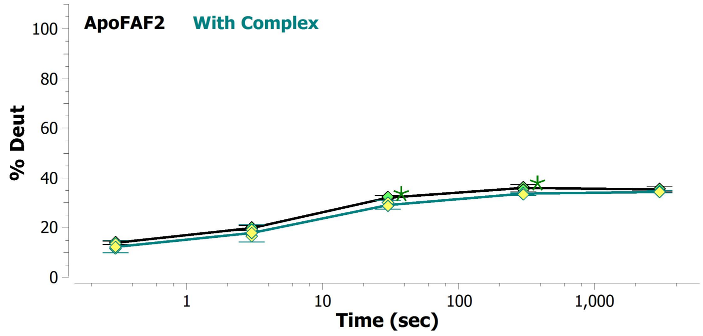

Q96CS3 226-236: RENTYPFLAMI (#62)

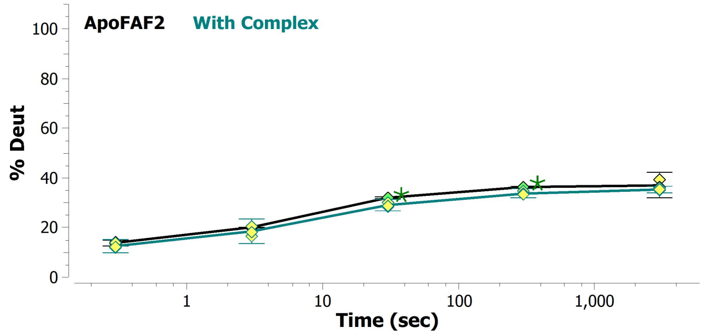

Q96CS3 235-258: MIMLKDRRMTVVGRLEGLIQPDDL (#63)

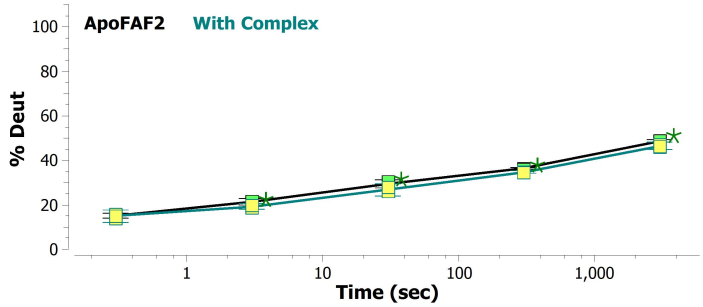

Q96CS3 236-249: IMLKDRRMTVVGRL (#64)

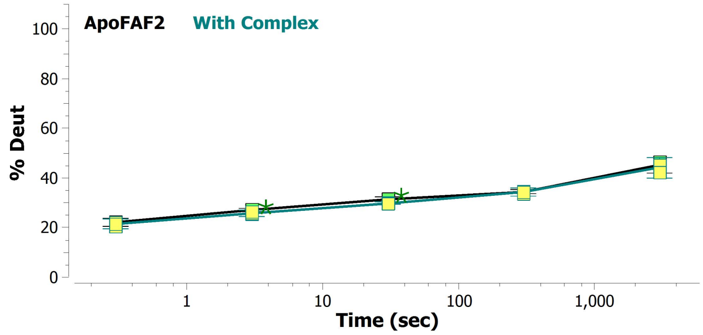

Q96CS3 236-251: IMLKDRRMTVVGRLEG (#65)

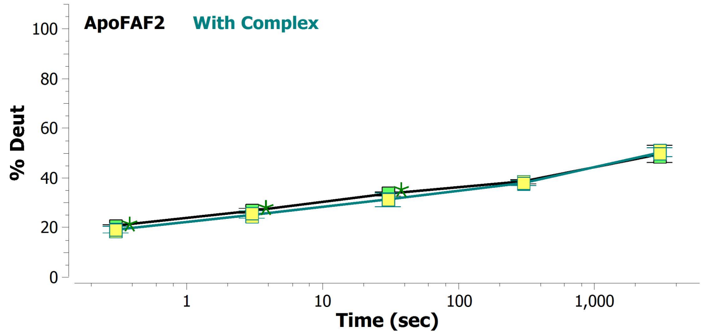

Q96CS3 236-258: IMLKDRRMTVVGRLEGLIQPDDL (#66)

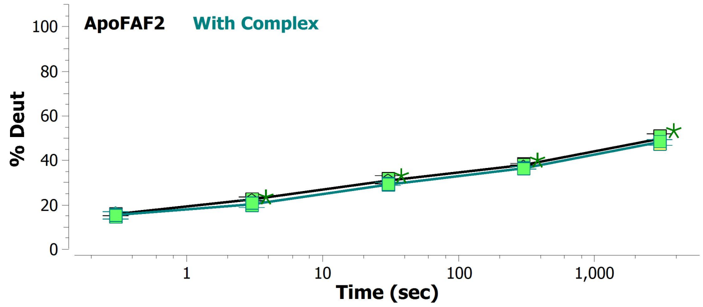

Q96CS3 236-261: IMLKDRRMTVVGRLEGLIQPDDLINQ (#67)

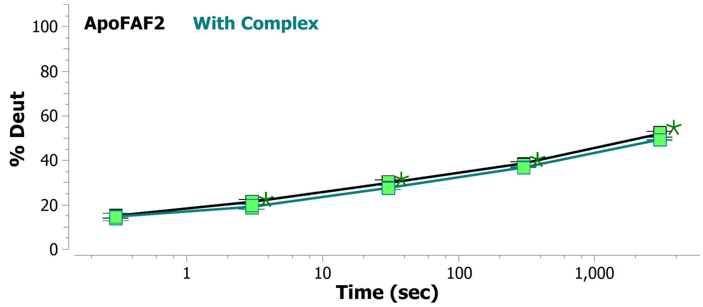

Q96CS3 236-263: IMLKDRRMTVVGRLEGLIQPDDLINQLT (#68)

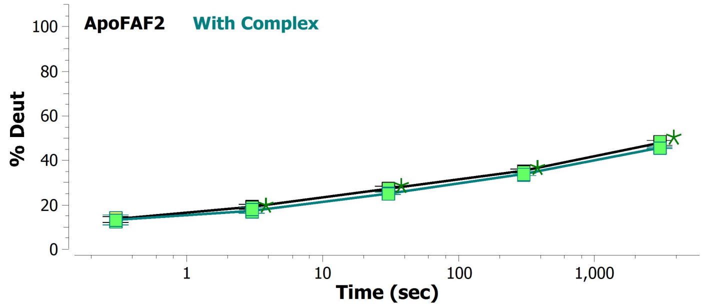

Q96CS3 236-264: IMLKDRRMTVVGRLEGLIQPDDLINQLTF (#69)

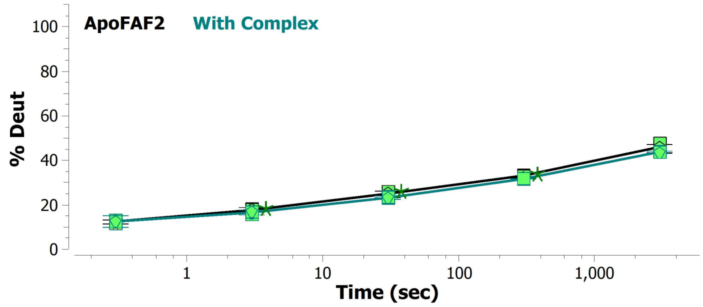

Q96CS3 237-265: MLKDRRMTVVGRLEGLIQPDDLINQLTFI (#70)

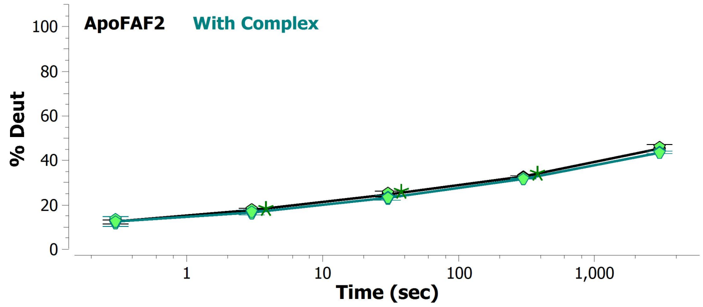

Q96CS3 238-258: LKDRRMTVVGRLEGLIQPDDL (#71)

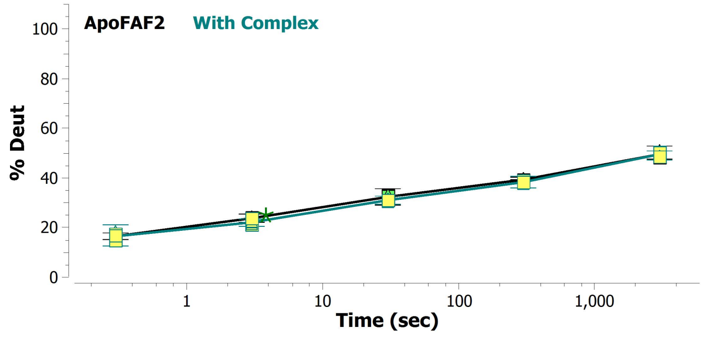

Q96CS3 238-261: LKDRRMTVVGRLEGLIQPDDLINQ (#72)

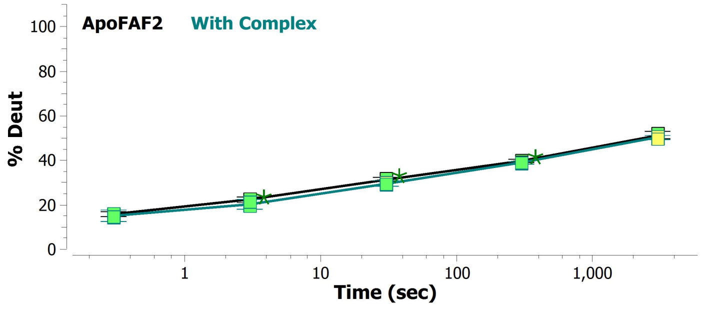

Q96CS3 238-263: LKDRRMTVVGRLEGLIQPDDLINQLT (#73)

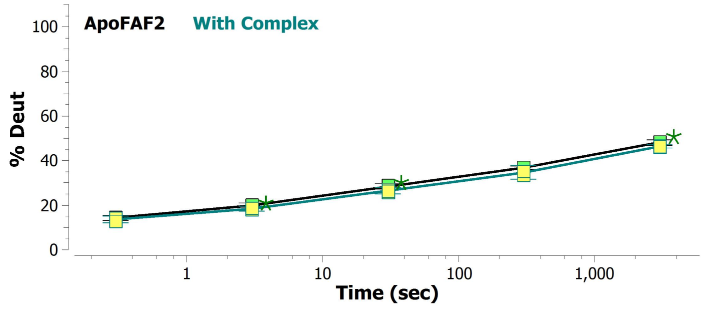

Q96CS3 238-264: LKDRRMTVVGRLEGLIQPDDLINQLTF (#74)

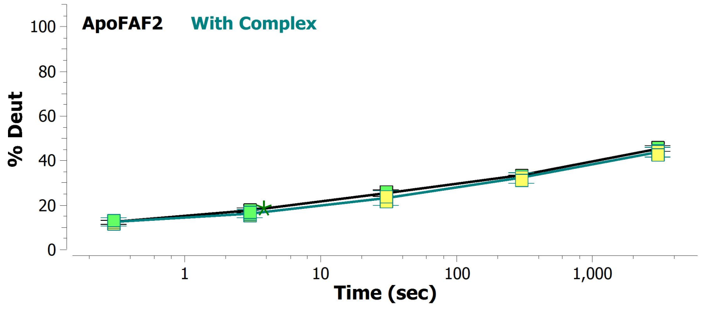

Q96CS3 239-259: KDRRMTVVGRLEGLIQPDDLI (#75)

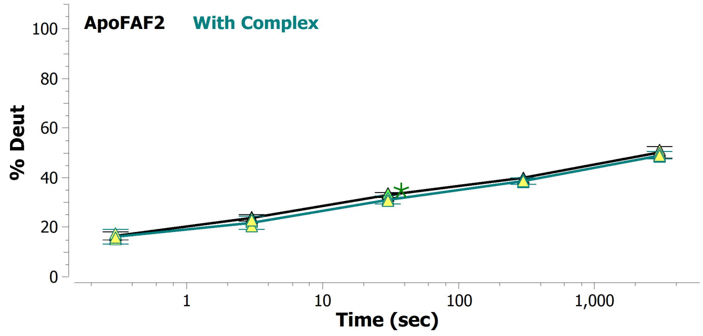

Q96CS3 239-264: KDRRMTVVGRLEGLIQPDDLINQLTF (#76)

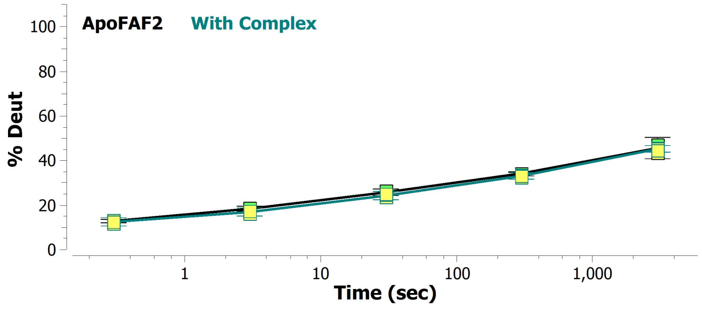

Q96CS3 243-284: MTVVGRLEGLIQPDDLINQLTFIMDANQTYLVSERLEREERN (#77)

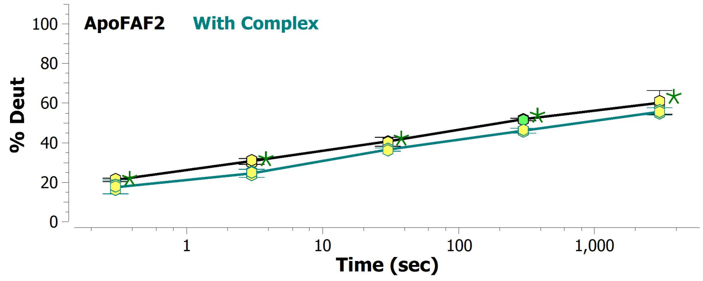

Q96CS3 261-283: QLTFIMDANQTYLVSERLEREER (#78)

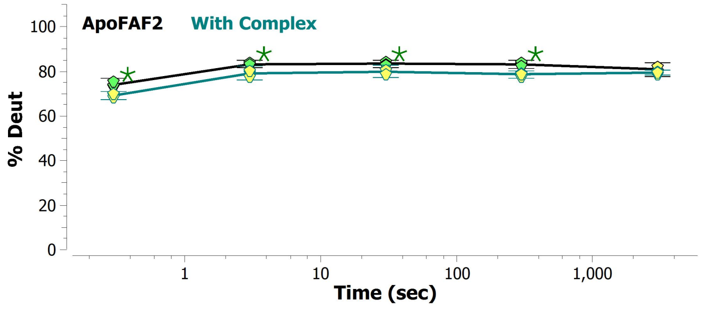

Q96CS3 264-273: FIMDANQTYL (#79)

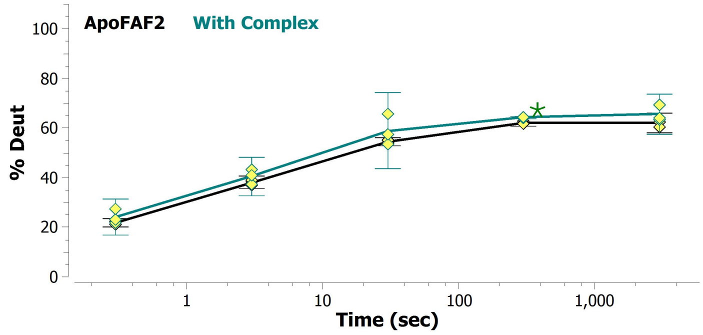

Q96CS3 265-273: IMDANQTYL (#80)

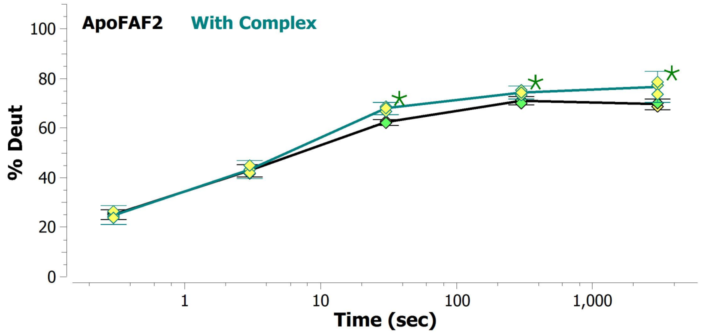

Q96CS3 265-289: IMDANQTYLVSERLEREERNQTQVL (#81)

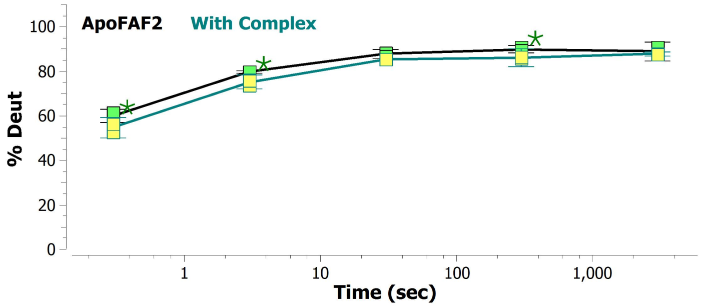

Q96CS3 270-292: QTYLVSERLEREERNQTQVLRQQ (#82)

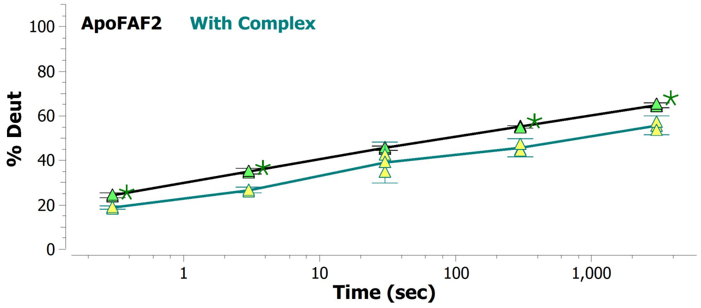

Q96CS3 274-296: VSERLEREERNQTQVLRQQQDEA (#83)

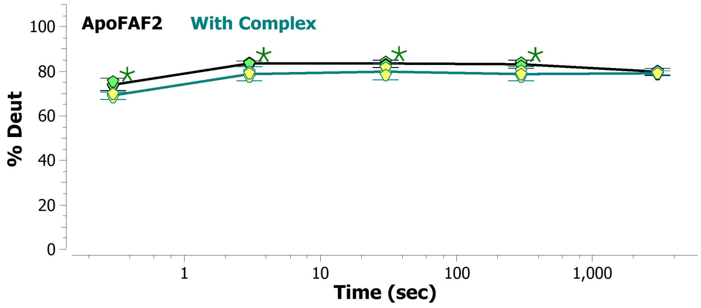

Q96CS3 274-298: VSERLEREERNQTQVLRQQQDEAYL (#84)

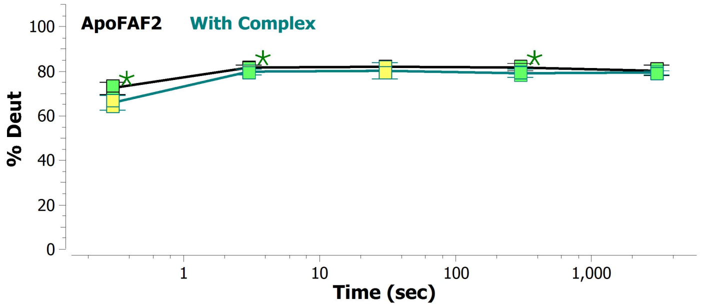

Q96CS3 276-298: ERLEREERNQTQVLRQQQDEAYL (#85)

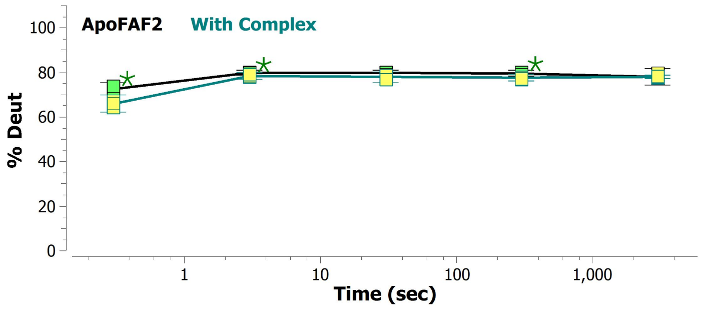

Q96CS3 277-298: RLEREERNQTQVLRQQQDEAYL (#86)

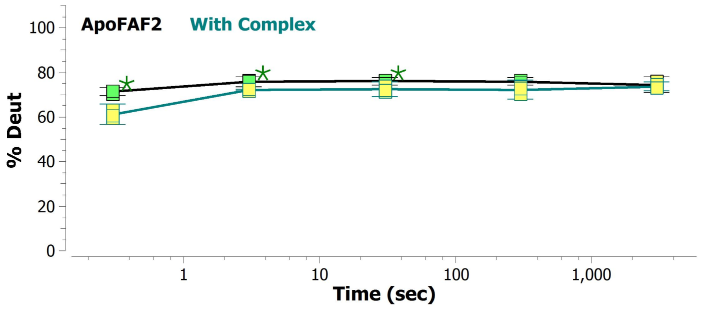

Q96CS3 279-298: EREERNQTQVLRQQQDEAYL (#87)

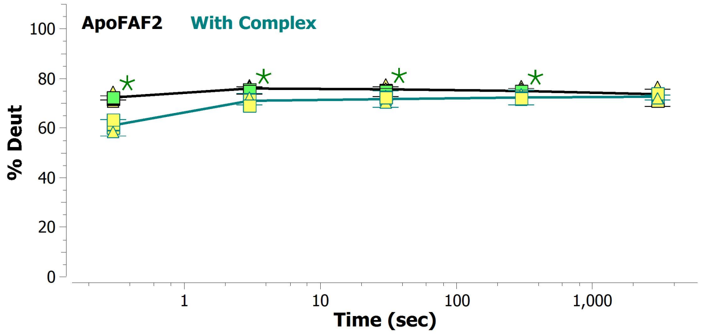

Q96CS3 324-347: EVQQQKLAEERRRQNLQEEKERKL (#88)

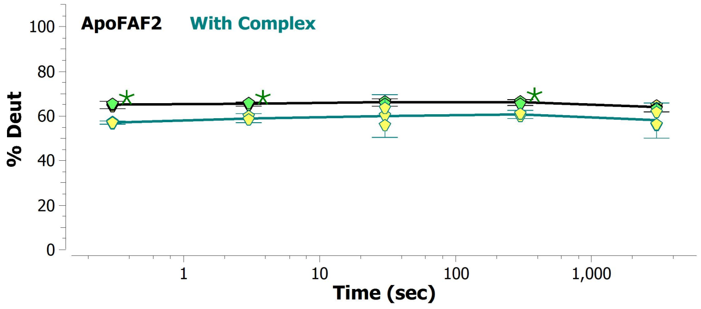

Q96CS3 339-364: LQEEKERKLECLPPEPSPDDPESVKI (#89)

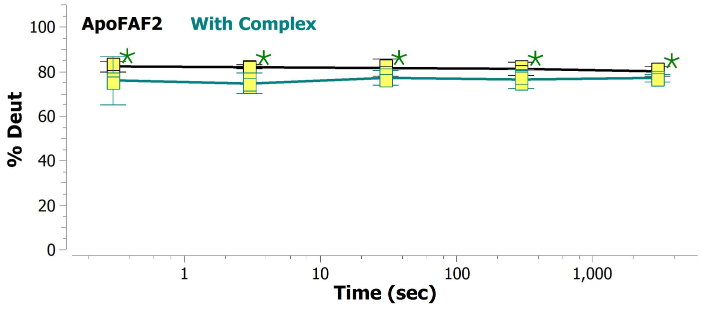

Q96CS3 342-384: EKERKLECLPPEPSPDDPESVKIIFKLPNDSRVERRFHFSQSL (#90)

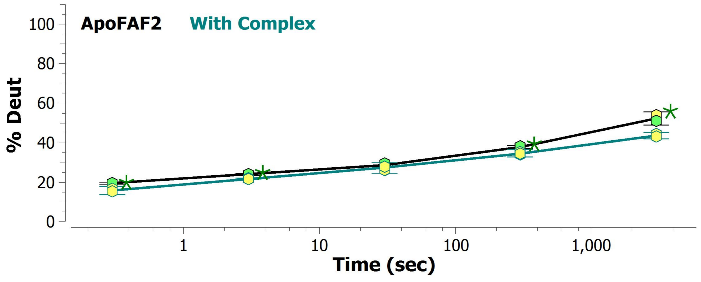

Q96CS3 356-384: PDDPESVKIIFKLPNDSRVERRFHFSQSL (#91)

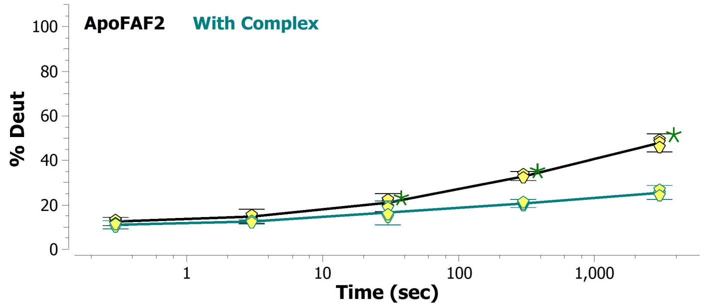

Q96CS3 362-384: VKIIFKLPNDSRVERRFHFSQSL (#92)

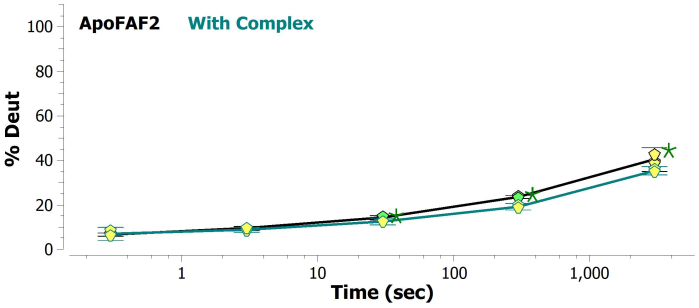

Q96CS3 363-374: KIIFKLPNDSRV (#93)

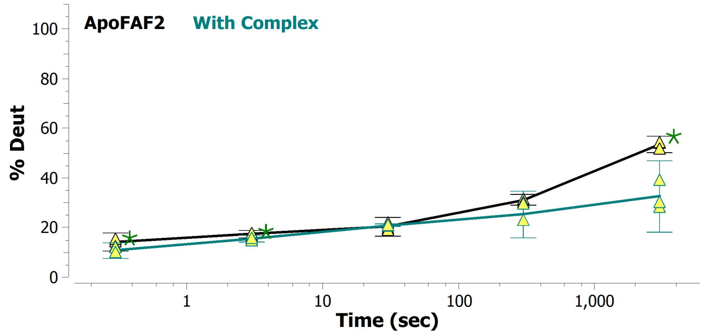

Q96CS3 384-391: LTVIHDFL (#94)

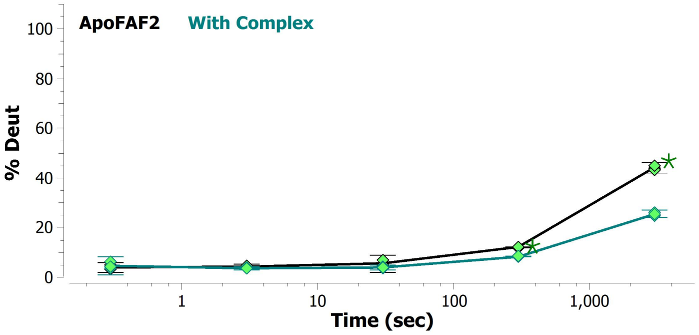

Q96CS3 385-391: TVIHDFL (#95)

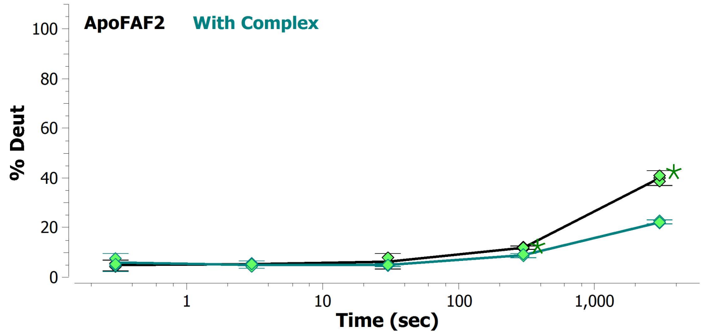

Q96CS3 385-392: TVIHDFLF (#96)

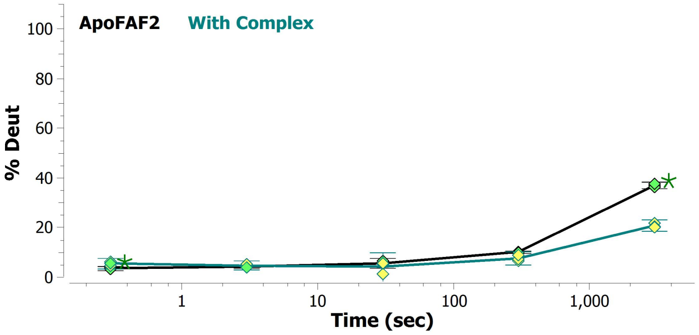

Q96CS3 386-391: VIHDFL (#97)

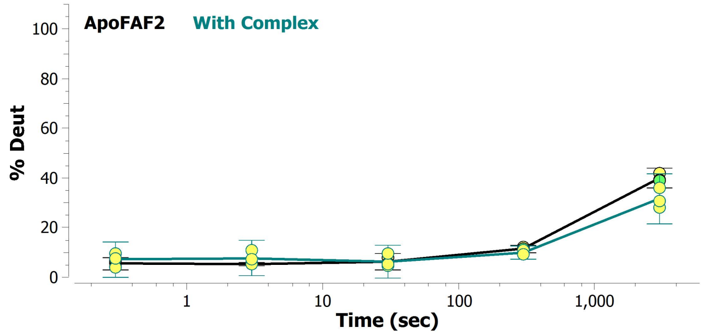

Q96CS3 391-425: LFSLKESPEKFQIEANFPRRVLPCIPSEEWPNPPT (#98)

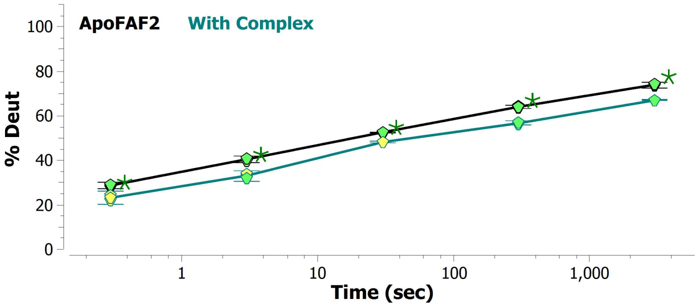

Q96CS3 391-430: LFSLKESPEKFQIEANFPRRVLPCIPSEEWPNPPTLQEAG (#99)

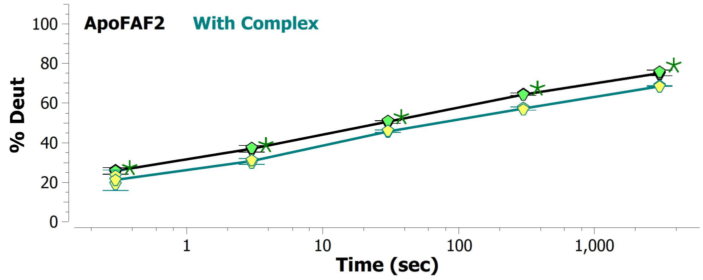

Q96CS3 392-401: FSLKESPEKF (#100)

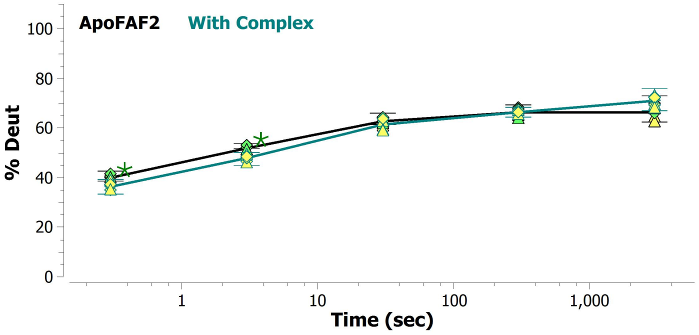

Q96CS3 392-404: FSLKESPEKFQIE (#101)

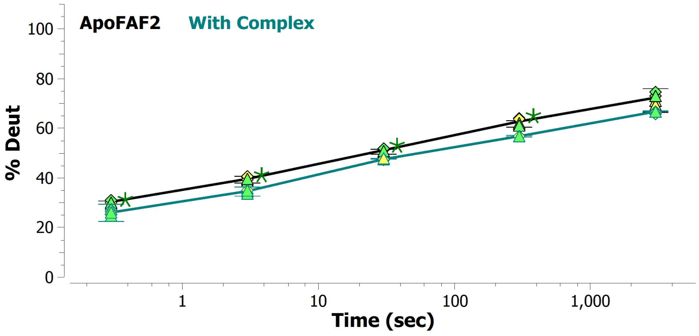

Q96CS3 392-407: FSLKESPEKFQIEANF (#102)

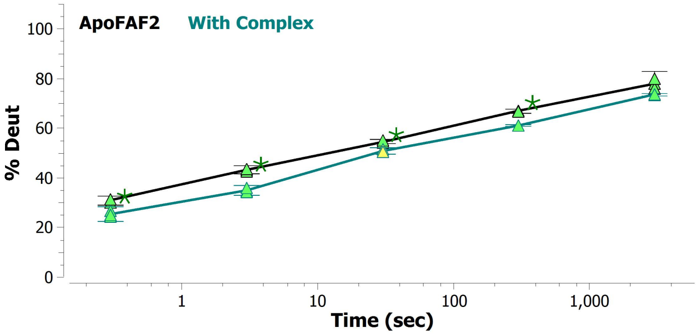

Q96CS3 392-426: FSLKESPEKFQIEANFPRRVLPCIPSEEWPNPPTL (#103)

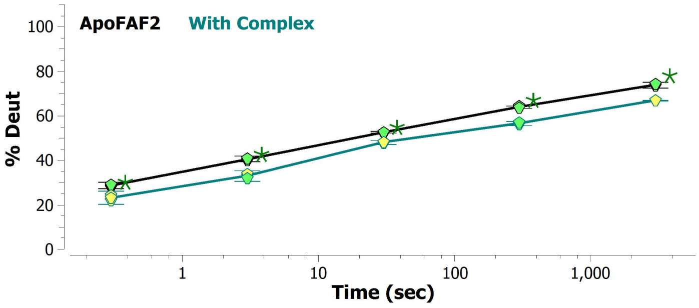

Q96CS3 392-427: FSLKESPEKFQIEANFPRRVLPCIPSEEWPNPPTLQ (#104)

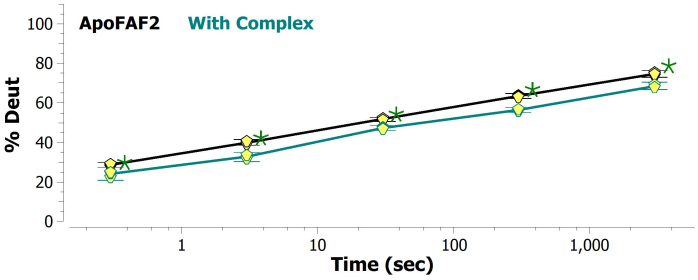

Q96CS3 392-431: FSLKESPEKFQIEANFPRRVLPCIPSEEWPNPPTLQEAGL (#105)

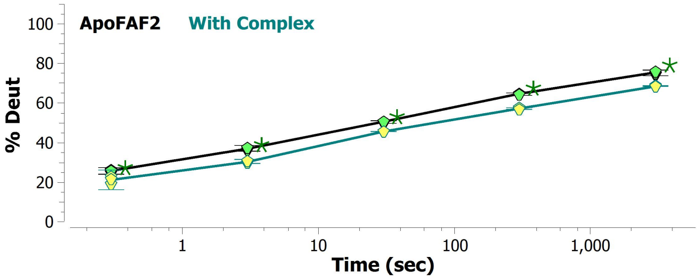

Q96CS3 392-437: LKESPEKFQIEANFPRRVLPCIPSEEWPNPPTLQEAGLSHTE (#106)

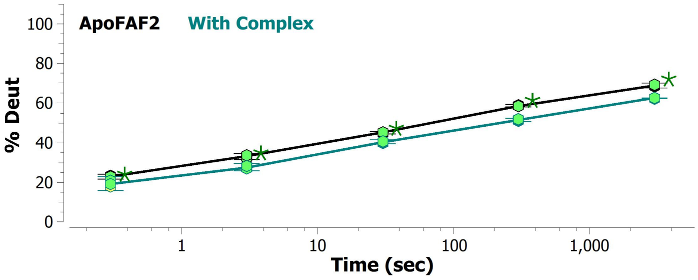

Q96CS3 393-438: KESPEKFQIEANFPRRVLPCIPSEEWPNPPTLQEAGLSHTEV (#107)

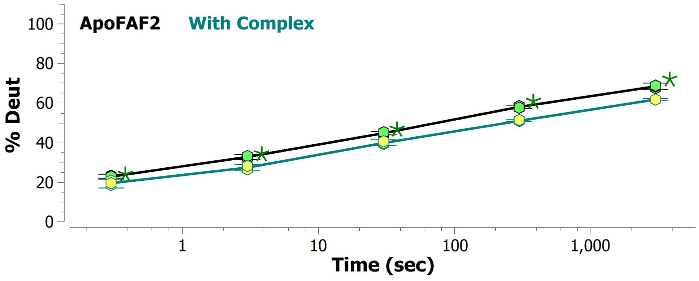

Q96CS3 396-420: ESPEKFQIEANFPRRVLPCIPSEEW (#108)

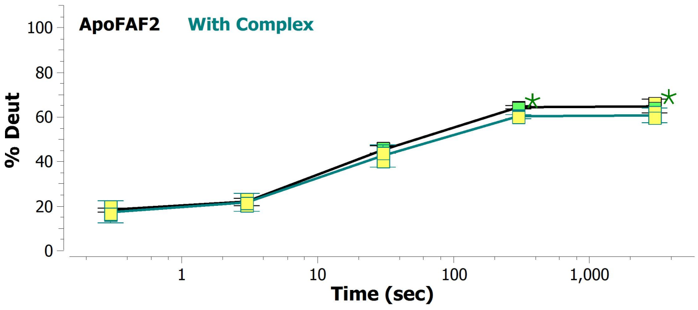

Q96CS3 400-434: KFQIEANFPRRVLPCIPSEEWPNPPTLQEAGLSHT (#109)

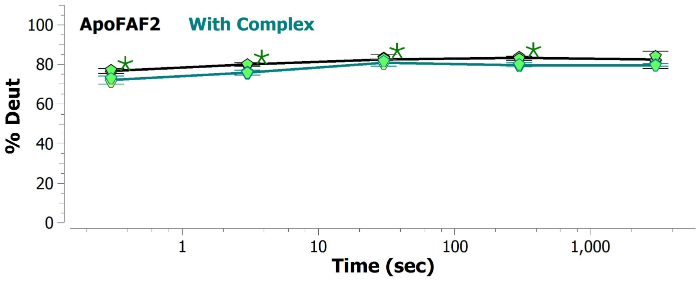

Q96CS3 402-428: QIEANFPRRVLPCIPSEEWPNPPTLQE (#110)

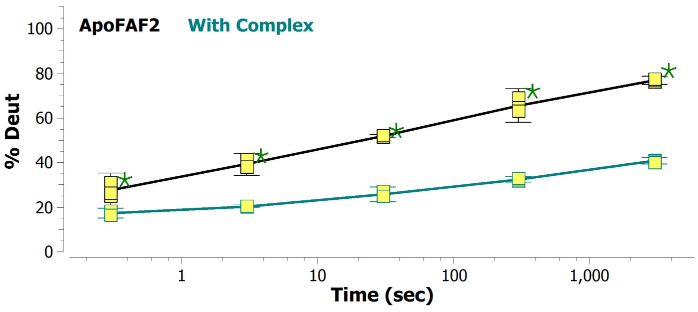

Q96CS3 403-427: IEANFPRRVLPCIPSEEWPNPPTLQ (#111)

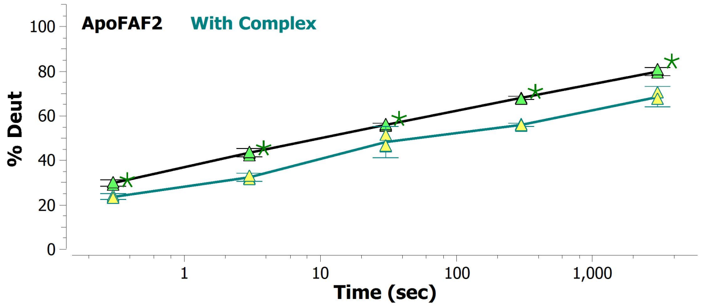

Q96CS3 405-426: ANFPRRVLPCIPSEEWPNPPTL (#112)

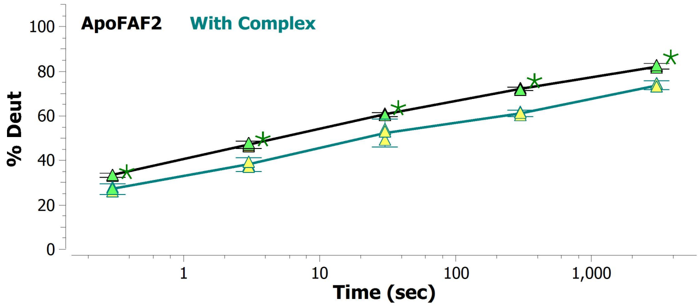

Q96CS3 405-431: ANFPRRVLPCIPSEEWPNPPTLQEAGL (#113)

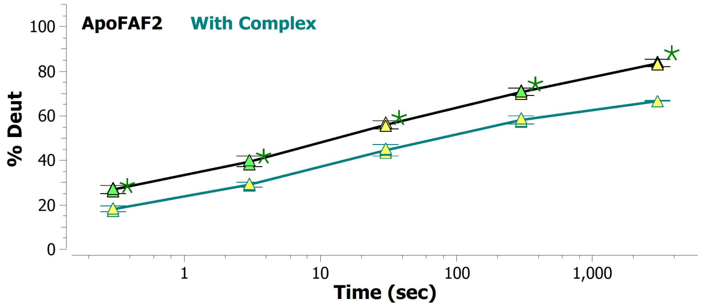

Q96CS3 405-437: ANFPRRVLPCIPSEEWPNPPTLQEAGLSHTEVL (#114)

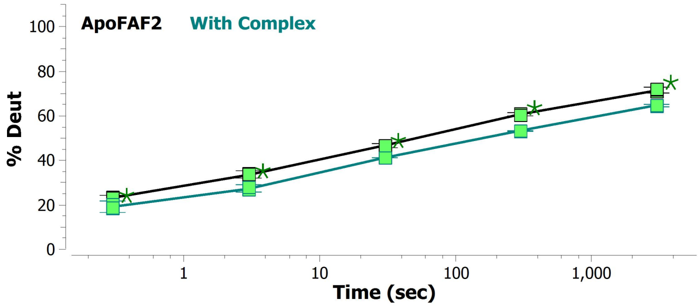

Q96CS3 405-438: ANFPRRVLPCIPSEEWPNPPTLQEAGLSHTEVLF (#115)

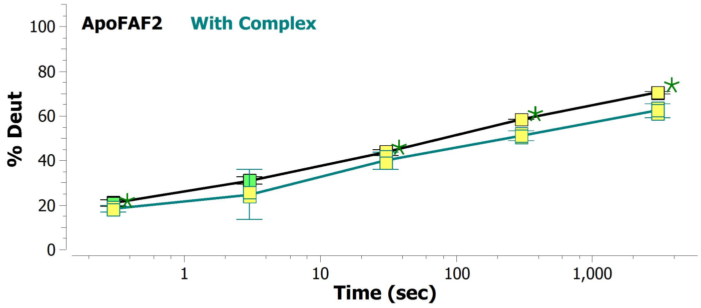

Q96CS3 408-426: PRRVLPCIPSEEWPNPPTL (#116)

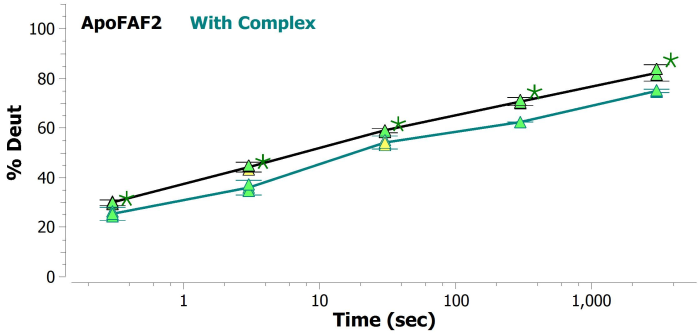

Q96CS3 408-437: PRRVLPCIPSEEWPNPPTLQEAGLSHTEVL (#117)

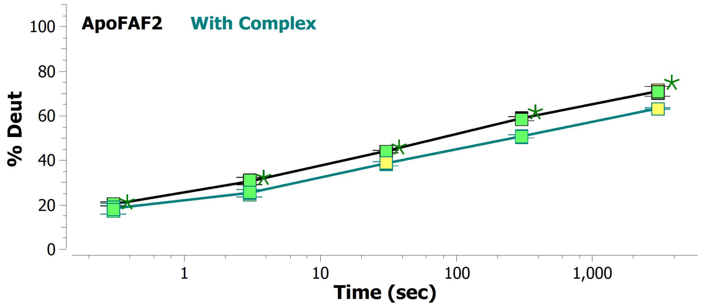

Q96CS3 411-432: VLPCIPSEEWPNPPTLQEAGLS (#118)

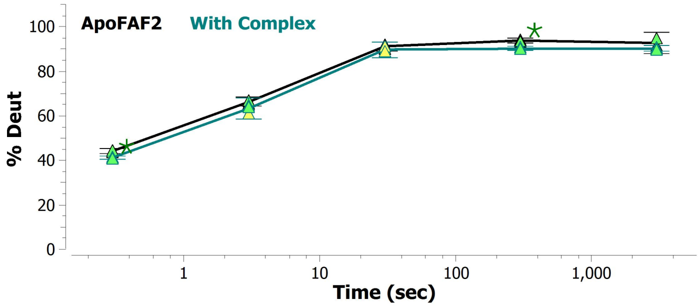

Q96CS3 427-437: QEAGLSHTEVL (#119)

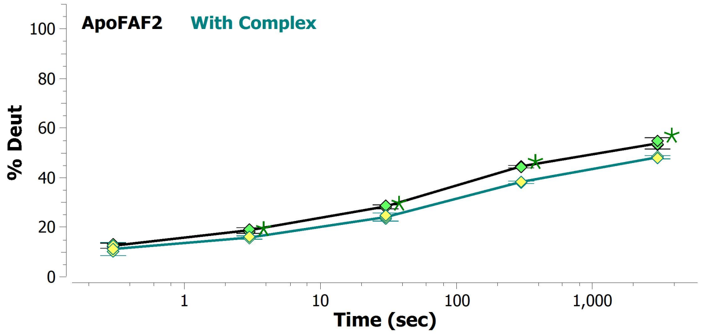

Q96CS3 427-438: QEAGLSHTEVLF (#120)

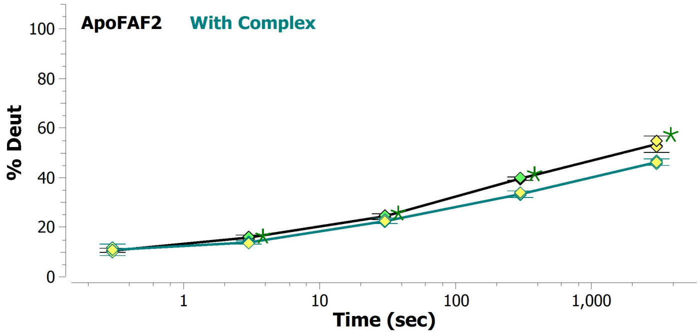

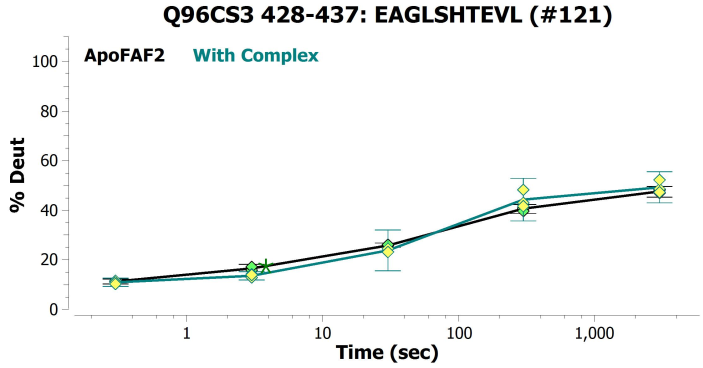
Q96CS3 428-437: EAGLSHTEVL (#121)
ApoFAF2
With Complex
% Deut
Time (sec)
0
20
40
60
80
100
1
10
100
1,000

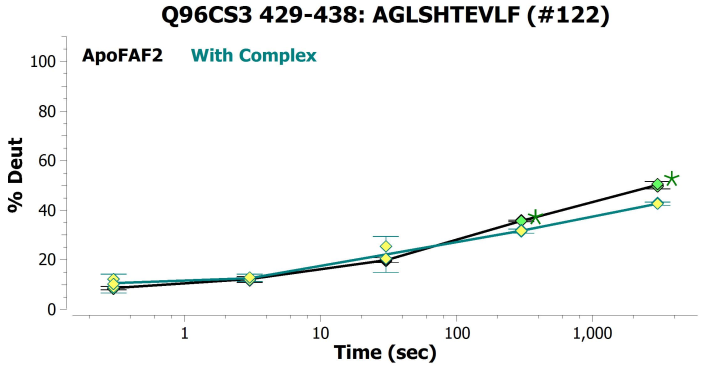
Q96CS3 429-438: AGLSHTEVLF (#122)
ApoFAF2
With Complex
% Deut
Time (sec)
0
20
40
60
80
100
1
10
100
1,000

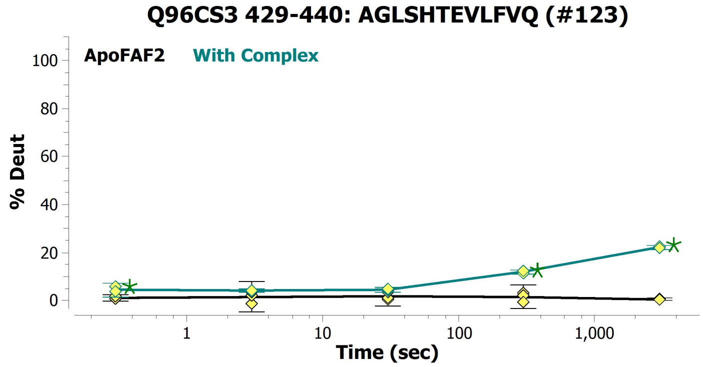
Q96CS3 429-440: AGLSHTEVLFVQ (#123)
ApoFAF2
With Complex
% Deut
Time (sec)
0
20
40
60
80
100
1
10
100
1,000
