# Supplementary material for: FAF1 and FAF2 enhance unfolding by p97-UFD1-NPL4 complex enabling rational design of p97 activators
Source: EMBO J. 2026 Aug 15;45(18):6595–629. doi: 10.1038/s44318-026-00894-x (PMC13578712; doi:10.1038/s44318-026-00894-x)
Supplement: Supplementary file 11 [file 44318_2026_894_MOESM6_ESM.zip › Figure 4/Fig 4E/faf2_coverage.pdf]

Q96CS3

```
  1 MAAPEERDLTQEQTEKLLQFQDLTGIESMDQCRHTLEQHNWNIEAAVQDR
 51 LNEQEGVPSVFNPPPSRPLQVNTADHRIYSYVVSRPQPRGLLGWGYYLIM
101 LPFRFTYYTILDIFRFALRFIRPDPRSRVTDPVGDIVSFMHSFEEKYGRA
151 HPVFYQGTYSQALNDAKRELRFLLVYLHGDDHQDSDEFCRNTLCAPEVIS
201 LINTRMLFWACSTNKPEGYRVSQALRENTYPFLAMIMLKDRRMTVVGRLE
251 GLIQPDDLINQLTFIMDANQTYLVSERLEREERNQTQVLRQQQDEAYLAS
301 LRADQEKERKKREERERKRRKEEEVQQQKLAEERRRQNLQEEKERKLECL
351 PPEPSPDDPESVKIIFKLPNDSRVERRFHFSQSLTVIHDFLFSLKESPEK
401 FQIEANFPRRVLPCIPSEEWPNPPTLQEAGLSHTEVLFVQDLTDE
```
