# Supplementary material for: FAF1 and FAF2 enhance unfolding by p97-UFD1-NPL4 complex enabling rational design of p97 activators
Source: EMBO J. 2026 Aug 15;45(18):6595–629. doi: 10.1038/s44318-026-00894-x (PMC13578712; doi:10.1038/s44318-026-00894-x)
Supplement: Supplementary file 11 [file 44318_2026_894_MOESM6_ESM.zip › Figure 4/Fig 4A/p97-UN complex coverage.pdf]

**UFD1**

1 MFSFNMFDHPIPRVFQNRFSTQYRCFSVSMLAGPNDRSDVEKGGKIIMPP

51 SALDQLSRLNITYPMLFKLTNKNSDRMTHCGVLEFVADEGICYLPHWMMQ

101 NLLLEEGGLVQVESVNLQVATYSKFQPQSPDFLDITNPKAVLENALRNFA

151 CLTTGDVIAINYNEKIYELRVMETKPDKAVSIIECDMNVDFDAPLGYKEP

201 ERQVQHEESTEGEADHSGYAGELGFRAFSGSGNRLDGKKKGVEPSPSPIK

251 PGDIKRGIPNYEFKLGKITFIRNSRPLVKKVEEDEAGGRFVAFSGEGQSL

301 RKKGRKP

**NPL4**

1 MAESIIIRVQSPDGVKRITATKRETAATFLKKVAKEFGFQNNGFSVYINR

51 NKTGEITASSNKSLNLLKIKHGDLLFLFPSSLAGPSSEMETSVPPGFKVF

101 GAPNVVEDEIDQYLSKQDGKIYRSRDPQLCRHGPLGKCVHCVPLEPFDED

151 YLNHLEPPVKHMSFHAYIRKLTGGADKGKFVALENISCKIKSGCEGHLPW

201 PNGICTKCQPSAITLNRQKYRHVDNIMFENHTVADRFLDFWRKTGNQHFG

251 YLYGRYTEHKDIPLGIRAEVAAIYEPPQIGTQNSLELLEDPKAEVVDEIA

301 AKLGLRKVGWIFTDLVSEDTRKGTVRYSRNKDTYFLSSEECITAGDFQNK

351 HPNMCRLSPDGHFGSKFVTAVATGGPDNQVHFEGYQVSNQCMALVRDECL

401 LPCKDAPELGYAKESSSEQYVPDVFYKDVDKFGNEITQLARPLPVEYLII

451 DITTTFPKDPVYTFSISQNPFPIENRDVLGETQDFHSLATYLSQNTSSVF

501 LDTISDFHLLLFLVTNEVMPLQDSISLLLEAVRTRNEELAQTWKRSEQWA

551 TIEQLCSTVGGQLPGLHEYGAVGGSTHTATAAMWACQHCTFMNQPGTGHC

601 EMCSLPRT

**TERA**

-6 MHHHHHHMASGADSKGDDLSTAILKQKNRPNRLIVDEAINEDNSVVSLSQ

44 PKMDELQLFRGDTVLLKGKKRREAVCIVLSDDTCSDEKIRMNRVVRNNLR

94 VRLGDVISIQPCPDVKYGKRIHVLPIDDTVEGITGNLFEVYLKPYFLEAY

144 RPIRKGDIFLVRGGMRAVEFKVVETDPSPYCIVAPDTVIHCEGEPIKRED

194 EEESLNEVGYDDIGGCRKQLAQIKEMVELPLRHPALFKAIGVKPPRGILL

244 YGPPGTGKTLIARAVANETGAFFFLINGPEIMSKLAGESESNLRKAFEEA

294 EKNAPAIIFIDELDAIAPKREKTHGEVERRIVSQLLTLMDGLKQRAHVIV

344 MAATNRPNSIDPALRRFGRFDREVDIGIPDATGRLEILQIHTKNMKLADD

394 VDLEQVANETHGHVGADLAALCSEAALQAIRKKMDLIDLEDETIDAEVMN

444 SLAVTMDDFRWALSQSNPSALRETVVEVPQVTWEDIGGLEDVKRELQELV

494 QYPVEHPDKFLKFGMTPSKGVLFYGPPGCGKTLLAKAIANECQANFISIK

544 GPELLTMWFGESEANVREIFDKARQAAPCVLFFDELDSIAKARGGNIGDG

594 GGAADRVINQILTEMDGMSTKKNVFIIGATNRPDIIDPAILRPGRLDQLI

644 YIPLPDEKSRVAILKANLRKSPVAKDVDLEFLAKMTNGFSGADLTEICQR

694 ACKLAIRESIESEIRRERERQTNPSAMEVEEDDPVPEIRRDHFEEAMRFA

744 RRSVSDNDIRKYEMFAQTLQQSRGFGSFRFPSGNQGGAGPSQGSGGGTGG

794 SVYTEDNDDDLYG
